# Inhibition of DYRK1A, via histone modification, promotes cardiomyocyte cell cycle activation and cardiac repair after myocardial infarction

**DOI:** 10.1016/j.ebiom.2022.104139

**Published:** 2022-07-08

**Authors:** Cong Lan, Caiyu Chen, Shuang Qu, Nian Cao, Hao Luo, Cheng Yu, Na Wang, Yuanzheng Xue, Xuewei Xia, Chao Fan, Hongmei Ren, Yongjian Yang, Pedro A. Jose, Zaicheng Xu, Gengze Wu, Chunyu Zeng

**Affiliations:** a Department of Cardiology, Daping Hospital, The Third Military Medical University, Chongqing, PR China; b Department of Cardiology, General Hospital of Western Theater Command, Chengdu, PR China; c Chongqing Key Laboratory for Hypertension Research, Chongqing Cardiovascular Clinical Research Center, Chongqing Institute of Cardiology, Chongqing, PR China; d Department of Cardiology, the Sixth Medical Centre, Chinese PLA General Hospital, Beijing, PR China; e Department of Internal Medicine, the 519th Hospital of Chinese PLA, Xichang, PR China; f Department of Cancer Center, Second Affiliated Hospital, Chongqing Medical University, Chongqing, China; g State Key Laboratory of Trauma, Burns and Combined Injury, Daping Hospital, The Third Military Medical University, Chongqing, PR China; h Cardiovascular Research Center of Chongqing College, Department of Cardiology of Chongqing General Hospital, University of Chinese Academy of Sciences, Chongqing, PR China; i Division of Renal Diseases & Hypertension, Department of Medicine and Department of Physiology/Pharmacology, The George Washington University School of Medicine & Health Sciences, Washington DC, United States

**Keywords:** Cardiomyocyte cell cycle activation, Cardiac repair, DYRK1A, H3K27ac, H3K4me3

## Abstract

**Background:**

While the adult mammalian heart undergoes only modest renewal through cardiomyocyte proliferation, boosting this process is considered a promising therapeutic strategy to repair cardiac injury. This study explored the role and mechanism of dual-specificity tyrosine regulated kinase 1A (DYRK1A) in regulating cardiomyocyte cell cycle activation and cardiac repair after myocardial infarction (MI).

**Methods:**

DYRK1A-knockout mice and DYRK1A inhibitors were used to investigate the role of DYRK1A in cardiomyocyte cell cycle activation and cardiac repair following MI. Additionally, we explored the underlying mechanisms by combining genome-wide transcriptomic, epigenomic, and proteomic analyses.

**Findings:**

In adult mice subjected to MI, both conditional deletion and pharmacological inhibition of DYRK1A induced cardiomyocyte cell cycle activation and cardiac repair with improved cardiac function. Combining genome-wide transcriptomic and epigenomic analyses revealed that DYRK1A knockdown resulted in robust cardiomyocyte cell cycle activation (shown by the enhanced expression of many genes governing cell proliferation) associated with increased deposition of trimethylated histone 3 Lys4 (H3K4me3) and acetylated histone 3 Lys27 (H3K27ac) on the promoter regions of these genes. Mechanistically, via unbiased mass spectrometry, we discovered that WD repeat-containing protein 82 and lysine acetyltransferase 6A were key mediators in the epigenetic modification of H3K4me3 and H3K27ac and subsequent pro-proliferative transcriptome and cardiomyocyte cell cycle activation.

**Interpretation:**

Our results reveal a significant role of DYRK1A in cardiac repair and suggest a drug target with translational potential for treating cardiomyopathy.

**Funding:**

This study was supported in part by grants from the National Natural Science Foundation of China (81930008, 82022005, 82070296, 82102834), National Key R&D Program of China (2018YFC1312700), Program of Innovative Research Team by the National Natural Science Foundation (81721001), and National Institutes of Health (5R01DK039308-31, 7R37HL023081-37, 5P01HL074940-11).

**Research in context**

**Evidence before this study**

The adult mammalian heart is endowed with regenerative capacity, albeit limited, via proliferation of pre-existing cardiomyocytes. Boosting cardiomyocyte cell cycle activity is a promising therapeutic strategy in repairing cardiac injury. Dual-specificity tyrosine regulated kinase 1A (DYRK1A), an evolutionarily conserved protein kinase, pleiotropically regulates the proliferation of tumor cells, neuronal progenitor cells, pancreatic β-cells, and cardiomyocytes in neonatal mice. However, the mechanisms by which DYRK1A regulates adult cardiomyocyte cell cycle activity and cardiac repair remain unknown.

**Added value of this study**

Our study demonstrated that either conditional deletion or pharmacological inhibition of DYRK1A induced cardiomyocyte cell cycle activation along with improved cardiac function in adult mice subjected to MI. We also found that DYRK1A phosphorylates WD repeat-containing protein 82 and lysine acetyltransferase 6A to reduce the deposition of trimethylated histone 3 Lys4 and acetylated histone 3 Lys27 on the promoters of cell cycle regulators, thus limiting their transcriptional activation and inhibiting cardiomyocyte cell cycle activity.

**Implications of all the available evidence**

Our findings suggest a significant role of DYRK1A in cardiac repair and as a drug target with translational potential for the treatment of cardiomyopathy.

Alt-text: Unlabelled box

## Introduction

Heart failure is one of the major causes of death, with more than 25 million people affected globally.^1^ The inability of the adult heart to replenish lost or damaged myocardium is a common pathophysiological basis of heart failure. Recently, an emerging paradigm-breaking concept reveals that the adult heart is still endowed with regenerative capacity via the proliferation of pre-existing cardiomyocytes,^2^ albeit significantly limited compared to that of perinatal mammals^3^ or lower vertebrates such as the zebrafish.^4^ At present, the molecular mechanisms underlying the poor regenerative capacity of the adult heart remain largely unknown, despite intensive investigative efforts, thus hampering the development of drug-based therapies for cardiac regeneration.

Dual-specificity tyrosine regulated kinase 1A (DYRK1A) is an evolutionarily conserved protein kinase mapped to the Down syndrome-critical region in human chromosome 21 and is involved in many cellular functions, including cell proliferation, survival, and differentiation.^5^ It is a pleiotropic kinase with an inconsistent role in regulating tumor cell proliferation; however, it most commonly shows a pro-proliferative effect,^6^^,^^7^ thus representing a promising therapeutic target for anti-tumor drugs. DYRK1A also plays an essential role in controlling neuronal progenitor cell division via regulation of epidermal growth factor receptor-based signaling during early development.^8^ Other studies further revealed that inhibition of DYRK1A stimulates pancreatic β-cell proliferation, which increases islet mass and improves glycemic control.^9^ Intriguingly, overexpression of DYRK1A impairs cardiomyocyte cell cycle progression, leading to dilated cardiomyopathy associated with congestive heart failure and premature death in postnatal mice.^10^ These observations imply that DYRK1A may be an extremely powerful regulator of cell proliferation, even if the cell type is resistant to stimulation of cell division or prone to excessive proliferation.

We identified DYRK1A as a key target in regulating cardiomyocyte cell cycle activation and cardiac repair in the adult heart after MI. Mechanistically, DYRK1A phosphorylates WDR82 and KAT6A to reduce the deposition of H3K4me3 and H3K27ac on the promoters of cell cycle regulators, thus limiting their transcriptional activation and inhibiting cardiomyocyte cell cycle activity. This study revealed a significant role of DYRK1A in adult cardiomyocyte cycling and identified it as an effective therapeutic target for cardiac repair after myocardial injury.

## Methods

### Animal

All experimental procedures were approved by the Animal Care and Use Committee of the Third Military Medical University (AMUWEC202113554) and conformed to the guidelines from the Directive 2010/63/EU of the European Parliament on the protection of animals used for scientific purposes. All experiments were performed using age-matched mice. C57BL/6J mice (RRID: IMSR_JAX:000664) were purchased from the Experimental Animal Center of the Daping Hospital. α-MHC^MerCreMer^ mice (RRID: IMSR_JAX:005657) were obtained from the Jackson Laboratory (Bar Harbor, Maine, USA). The mice were housed in standard cages with a 12-h light/12-h dark cycle at 22 to 24°C and *ad libitum* access to food and water.

### Generation of DYRK1A-knockout mice

DYRK1A^flox/flox^ mice were generated using the Cas9 nickase method. Two single-guide RNAs (sgRNA1 and sgRNA2) targeting DYRK1A introns 1 and 2 were designed to flank the second exon of DYRK1A with loxP sites. The donor plasmid containing DYRK1A exon 2 flanked by two loxP sites, sgRNA1 and sgRNA2, and Cas9 mRNAs was co-injected into one-cell-stage fertilized embryos to obtain mice with DYRK1A exon 2 flanked by two loxP sites on one allele. Homozygous DYEK1A-flox (DYRK1A^flox/flox^) mice were obtained, and α-MHC^MerCreMer^/DYRK1A^flox/flox^ mice were then generated by crossing DYRK1A^flox/flox^ mice with α-MHC^MerCreMer^ mice. Cardiomyocyte-specific deletion of DYRK1A (DYRK1A CKO) was cyclic recombinase (Cre)-induced by thrice intraperitoneal injection of tamoxifen (50 mg/kg), every other day in adult mice.

### Myocardial infarction (MI)

As previously described, MI was surgically induced.^11^ Mice were anesthetized by inhalation of isoflurane (2%), and ventilation was provided through tracheal intubation, connected to a small-animal anesthesia ventilator. After left thoracotomy through the fourth intercostal space, the left anterior descending coronary artery was visualized and permanently ligated using a 7-0 nylon suture under an operating microscope. After the chest wall was closed, the mice were allowed to recover with free access to food and water. Sham-operated mice underwent a similar surgical procedure without arterial ligation.

### Echocardiography

Cardiac function of the mice was measured at the indicated time points by echocardiography using a small-animal high-resolution ultrasound imaging system (Vevo 2100, Visual Sonics, Canada). Two-dimensional guided M-mode measurements at the parasternal long-axis plane were performed for at least three beats and then averaged. All measurements were performed by a technician blinded to the experimental groups. The left ventricular internal diameter at end-diastole (LVIDd) and left ventricular internal diameter at end-systole (LVIDs) were measured to determine cardiac function, while the left ventricular ejection fraction (LVEF) was calculated as 100% × (LVIDd^3^ − LVIDs^3^) / LVIDd^3^.

### Cell culture

Neonatal cardiomyocytes were isolated from the hearts of 1–2-day-old Sprague-Dawley rats using the previously described enzymatic digestion method.^12^ The hearts were extracted, minced into pieces smaller than 1 mm^3^, and subsequently subjected to enzymatic digestion with 1.25 mg/mL trypsin for 3 min and 0.8 mg/mL collagenase II for 30 min at 37°C. The digestion was halted by adding Dulbecco's modified Eagle's medium (DMEM), supplemented with 10% fetal bovine serum (FBS). After centrifugation at 300 g for 5 min, the cell pellet was resuspended and incubated at 37°C for 90 min for differential attachment. Subsequently, the cells in the suspension were plated and cultured in DMEM with 10% FBS at 37°C in an atmosphere with 5% CO_2_ for 24 h.

Small interfering RNAs (siRNAs), targeting the genes of interest, were transfected into cardiomyocytes using the lipofectamine 3000 transfection reagent (L3000015, Thermo Fisher Scientific, Waltham, MA) and cultured for 48 h before subsequent analysis. For DYRK1A overexpression experiments, the coding sequence of rat DYRK1A was cloned into the pcDNA3.1 vector under the control of the cytomegalovirus promoter. Recombinant plasmids were then generated and cardiomyocytes transfected for 48 h.

We also constructed plasmids encoding hemagglutinin (HA)-tagged human full-length DYRK1A, mutated DYRK1A with deletion of the N, K, or C terminus, and FLAG-tagged full-length WDR82 or KAT6A. Co-transfection of these plasmids was performed in HEK293T cells (validated by short tandem repeat, RRID: CVCL_0063) using the lipofectamine 3000 transfection reagent. After co-transfection for 48 h, the cells were lysed for co-immunoprecipitation (IP) experiments to determine the binding domain of DYRK1A with WDR82 and KAT6A.

### Determining the number of cardiomyocytes per heart

Adult cardiomyocytes were isolated from mice at day 35 post-MI, according to a published protocol.^13^ The cells were fixed with 4% paraformaldehyde for one hour at room temperature and immunostained with cardiac troponin T to label cardiomyocytes in a 1.5-mL Eppendorf tube. The cell pellet was fully resuspended, 1-μL cell suspension was dropped and cover-slipped on a slide, and cardiomyocytes were counted under a fluorescence microscope (Nikon, Japan). The total cardiomyocyte number per heart was calculated as the average cell number per µL suspension × the total volume of cell suspension.

### Time-lapse imaging assay

To investigate whether loss of DYRK1A promotes cardiomyocyte cytokinesis *in vitro*, we performed a time-lapse imaging assay using an Olympus IX83 inverted microscope with a humidified cell culture chamber in the presence of 5% CO_2_ at 37°C. Initially, control (αMHC^MerCreMer^) and α-MHC^MerCreMer^/DYRK1A^flox/flox^ mice (1–3 days after birth) were infected with a single intraperitoneal injection of AAV9-GFP under the control of the cardiac troponin T promoter. GFP-labeled adult cardiomyocytes were isolated from eight-week old mice, according to a published protocol^13^ and co-cultured with primary neonatal rat cardiomyocytes in the cell culture chamber. More than 100 random fields of 20X objective lens were selected for time-lapse imaging at intervals of 1 h for 7 days. Only the division events with completed cytokinesis were counted.

### Culture of human induced pluripotent stem cell (iPSC)-derived cardiomyocytes (hiPSC-CMs)

Commercial hiPSC-CMs (validated by the commercial vendor) were purchased from Saibei Biotechnology (Beijing, China), and were seeded in a plating medium (Saibei Biotechnology, CA2020008) into fibronectin-coated plates. The hiPSC-CMs were routinely tested for mycoplasma contamination and cultured for one week prior to experimentation in a maintenance medium (Saibei Biotechnology, CA2015002).

### RNA isolation and real-time quantitative polymerase chain reaction (qPCR)

Total RNA was isolated from cells or tissues using TRIzol™ reagent (15596026, Thermo Fisher Scientific) according to the manufacturer's protocol. The total RNA concentration was measured using a Nanodrop 8000 spectrophotometer (Thermo Fisher Scientific). Additionally, 1-μg of total RNA was used to synthesize cDNA with the iScript Reverse Transcription Supermix (Bio-Rad, Hercules, CA, USA), as per the manufacturer's protocol. The generated cDNA was used as a template to perform real-time qPCR with a quantitative SYBR Green PCR mix on a QuantStudio 6 Flex Real-Time PCR system (Bio-Rad). Additionally, the relative expression of an individual gene was calculated using the 2−∆∆CT method, and glyceraldehyde 3-phosphate dehydrogenase (GAPDH) was used as the endogenous control.

### RNA-sequencing and data analysis

Neonatal cardiomyocytes in primary culture were transfected with siRNA against DYRK1A (si-DYRK1A) or scramble siRNA (si-NC) using lipofectamine 3000 transfection reagent and cultured for 48 h. RNA from cells transfected with scramble or DYRK1A siRNA was extracted using TRIzol™ reagent, according to the manufacturer's protocol. Additionally, RNA-sequencing (RNA-seq) libraries were prepared using the NEBNext Ultra™ RNA Library Prep Kit for the Illumina system, according to the manufacturer's instructions, and paired-end (150 bp) sequencing was performed using the HiSeq 3000 sequencer from the Novogene Bioinformatics Institute (Beijing, China). RNA-seq reads were mapped to Rnor_6.0 using HISAT2 (v2.0.5) with default settings, and fragments per kilobase of exon per million mapped reads were used to analyze differentially expressed transcripts between groups. Heatmaps of gene expression, gene ontology (GO) and Kyoto encyclopedia of genes and genomes (KEGG) pathway analyses were performed using the OmicShare tools, a free online platform for data analysis (http://www.omicshare.com/tools). RNA-seq data were deposited in the gene expression omnibus (GEO) database under the accession number GSE202169.

### Antibodies

The antibodies used for the chromatin immunoprecipitation (ChIP) assays were rabbit anti-H3K27ac (ab4729, Abcam, London, England, RRID: AB_2118291) and "rabbit anti-H3K4me3 (ab8580, Abcam, RRID: AB_306649). The following antibodies were used for immunofluorescence staining: mouse anti-cardiac troponin T (cTnT, MA5-12960, Thermo Fisher Scientific, RRID: AB_11000742), rabbit anti-Ki67 (PA5-19462, Thermo Fisher Scientific, RRID: AB_10981523), rabbit anti-phospho-histone H3 (pH3, PA5-17869, Thermo Fisher Scientific, RRID: AB_10984484), and rabbit anti-Aurkb (ab2254, Abcam, RRID: AB_302923). In addition, the following antibodies were used for IP: mouse anti-DYRKA (PAB19417, Abnova, Walnut, CA, RRID: AB_10904391), rabbit anti-phospho-Ser/Thr (ab117253, Abcam, RRID: AB_10903259), and rabbit anti-HA tag (ab9110, Abcam, RRID: AB_307019). The following antibodies were used for immunoblotting: mouse anti-DYRKA (PAB19417, Abnova, RRID: AB_10904391), rabbit anti-Ccna1 (bs-5739R, Bioss, Beijing, RRID: AB_11118565), rabbit anti-Ccnb1 (bsm-52044R, Bioss), rabbit anti-Ccnd1 (bs-20596R, Bioss), rabbit anti-Ccnd2 (bs-1148R, Bioss, RRID: AB_10857746), rabbit anti-Ccne1 (bs-0573R, Bioss, RRID: AB_10858063), rabbit anti-Cdc20 (ab183479,Abcam), rabbit anti-Cdc25a (bs-2758R, Bioss, RRID: AB_10855140), rabbit anti-Cdk1 (bsm-52026R, Bioss), mouse anti-Cdk4 (bsm-52028M, Bioss), rabbit anti-Pcna (bs-2006R, Bioss, RRID: AB_10855815), rabbit anti-Pole2 (bs-14356R, Bioss), rabbit anti-Plk1 (bs-3535R, Bioss, RRID: AB_10857021), rabbit anti-FoxM1 (ab180710, Abcam, RRID: AB_2893324), rabbit anti-KAT6A (PAB8745, Abnova), rabbit anti-WDR82 (PA5-110580, Thermo Fisher Scientific, RRID: AB_2855991), rabbit anti-H3K27ac (ab4729, Abcam, RRID: AB_2118291), rabbit anti-H3K4me3 (ab8580, Abcam, RRID: AB_306649), rabbit anti-H3K9me3 (ab8898, Abcam, RRID: AB_306848), mouse anti-H3K27me3 (ab6002, Abcam, RRID: AB_305237), rabbit anti-H4k20me3 (ab177190, Abcam, RRID: AB_2713955), rabbit anti-H4K5ac (ab124636, Abcam, RRID: AB_10976331), rabbit anti-H4K8ac (ab45166, Abcam, RRID: AB_732937), rabbit anti-histone H3 (ab1791, Abcam, RRID: AB_302613), mouse anti-FLAG tag (bsm-33346M, Bioss), and mouse anti-GAPDH (ab8245, Abcam, RRID: AB_2107448). All the antibodies were validated and used.

### ChIP assays

ChIP assays were performed using chromatin IP assay kits (17-371, Millipore, Milford, MA), as per the manufacturer's protocol. Briefly, rat neonatal cardiomyocytes were transfected with scramble or DYRK1A siRNA using lipofectamine 3000 transfection reagent (Thermo Fisher Scientific) and cultured for 48 h. The cells (about 1×10^7^ cells for each ChIP experiment) were then cross-linked with 1% formaldehyde for 10 min at room temperature, and the reaction was stopped with glycine (0.125 M) for 5 min. The cross-linked cells were harvested and lysed in a ChIP lysis buffer and chromatin was fragmented to 180–360 bp using an EZ-Zyme™ kit (17-295, Millipore), as per the manufacturer's protocols. Additionally, fragmented chromatin was immunoprecipitated with anti-H3K27ac and anti-H3K4me3 antibodies, or a negative control rabbit IgG. Furthermore, immunocomplexes were captured with protein G agarose, and crosslinking was reversed by incubating the samples in the presence of 200 mM NaCl at 65°C overnight. After treatment with RNase A and proteinase K, free DNA was purified using spin columns. The purified DNA fragments were analyzed by quantitative PCR using primers for the target gene promoter (within 2000bp upstream of the transcription start site) or subjected to ChIP-sequencing (ChIP-seq). The ChIP-qPCR results are expressed as enrichment relative to input.^14^ Furthermore, ChIP-seq libraries were constructed using the NEBNext ChIP-seq Library Prep Master Mix Set for the Illumina system, and sequencing was performed using a HiSeq 3000 sequencer (Guangzhou Epibiotek, Guangzhou, China). ChIP-seq reads were then mapped to rat genome assembly rn6. ChIP-seq peak calling was conducted using MACS 1.4.2 (Model based analysis of ChIP-seq) with default settings to profile binding regions, and peaks were visualized using integrative genomics viewer software. ChIP-seq heatmaps of the binding strength were drawn using the ggplot2 library. *De novo* motif analysis was performed within a 200-bp region around the peak centers using Homer software. Additionally, GO and KEGG pathway analyses were performed using the OmicShare tools, a free online platform for data analysis (http://www.omicshare.com/tools). ChIP-seq data were deposited in the GEO database under the accession number GSE202168.

### Immunoprecipitation

IP was performed to investigate protein interactions and phosphorylation. Briefly, rat neonatal cardiomyocytes in primary culture were lysed using ice-cold lysis buffer (P0013C, Beyotime Biotechnology) containing a protease inhibitor cocktail (P8340, Sigma, St Louis, MO, USA) and PhosStop phosphatase inhibitor (4906845001, Sigma). The lysates were cleared by centrifugation, and proteins were sequentially incubated with the indicated antibodies at 4°C overnight and protein A/G agarose (20421, Thermo Fisher Scientific) at room temperature for 2 h. The beads were washed thrice with the lysis buffer and centrifuged to obtain the indicated protein complexes, which were then subjected to immunoblotting or mass spectrometry.

### Immunoblot analysis

We prepared the cell or tissue lysates using an ice-cold lysis buffer for immunoblotting and IP (P0013, Beyotime Biotechnology) containing a protease inhibitor cocktail (P8340, Sigma) and protein concentrations were measured using the Quick Start Bradford Protein Assay Kit (Bio-Rad). Proteins were separated using sodium dodecyl-sulfate polyacrylamide gel electrophoresis and electrotransferred onto polyvinylidene difluoride membranes, followed by blocking for 2 h with Tris-buffered saline, 0.1% tween-20 detergent containing 5% non-fat dry milk. The membranes were then subsequently incubated with the indicated primary antibodies (1:1000 dilution) at 4°C overnight, and the corresponding secondary antibodies at room temperature for 2 h. The protein bands were then visualized using the Odyssey Infrared Imaging System (Li-Cor Biosciences, Lincoln, NE), and the intensity of bands was analyzed using Quantity One image analysis software. Additionally, GAPDH served as an internal control to normalize densitometric intensity. All uncropped blots are shown in Supplementary Data.

### Mass spectrometry analysis

Mass spectrometric analysis was performed at the Shanghai Applied Protein Technology Co, Ltd. (Shanghai, China). To profile DYRK1A-interacting effectors, DYRK1A complexes were immunoprecipitated, as described in the IP assay. The DYRK1A complexes obtained were then subjected to enzymatic digestion in trypsin solution (Promega, Madison, WI). The peptides were later extracted, dried in a speed-vac, and reconstituted in high-performance liquid chromatography (HPLC) solvent A (2.5% acetonitrile and 0.1% formic acid), and were subjected to standard tandem mass tag labeling using a commercial kit, as per the manufacturer's instructions (Thermo Fisher Scientific). Labeled peptides were fractionated with a high pH, reversed phase fractionation kit (Thermo Fisher Scientific), and an HPLC system AKTA Purifier 100 (General Electric, Fairfield, CT), as per the manufacturer's instructions. The peptides were further separated using an EASY-nLC 1200 systems coupled with a Thermo Q exactive mass spectrometer (Thermo Fisher Scientific). A full high-resolution scan was performed at 70,000 resolutions (300-1800 m/z), followed by 20 low-resolution MS/MS scans in the ion trap. Mascot2.2 and Proteome Discoverer 1.4 software were used for database searching, peptide mass fingerprinting, and peptide sequence tagging, thus achieving protein identification.

### Immunofluorescence staining

The cells or tissues were fixed with 4% paraformaldehyde and 4-μm sections of paraffin-embedded tissues were obtained. After dewaxing and antigen retrieval, fixed cells or tissue sections were permeabilized with Triton (0.1%) for 10 min and blocked in immunostaining blocking buffer containing 5% bovine serum albumin for 1 h at 37°C. The samples were subsequently incubated with the indicated primary antibodies (1:100 dilution) at 4°C overnight and corresponding secondary antibodies (conjugated with Alexa Fluor Plus 488 or 546) at 37°C for 2 h. After nuclear visualization after 10 min of DAPI staining, the samples were mounted, and immunofluorescence images were obtained under a laser confocal microscope (Olympus, Tokyo) and were analyzed using the Olympus Fluoview FV300 version 3C Acquisition Software.

### Masson's trichrome staining

Heart tissues were fixed in 4% paraformaldehyde and cut into five transverse slices from the apex to the site of occlusion. The left ventricular scars were then visualized using a modified Masson's trichrome staining kit (G1346, Solarbio Life Science, Beijing), as per the manufacturer's instructions. The scar size was quantified using Image J software and calculated as the circumference of fibrotic tissue divided by that of the total left ventricle from consecutive myocardial slices, as previously described.^15^

### Statistical analysis

All data are expressed as mean ± Standard Deviation (SD), and statistical calculations were performed using SPSS software (version 19.0). Two-tailed unpaired Student's t test was performed to compare means between two groups, and one-way analysis of variance (ANOVA), followed by Tukey's multiple-comparison test, was used for comparisons among three or more groups. Statistical significance was set at P < 0.05, and no outliers were excluded from the final statistical analysis.

### Role of the funding sources

No funding sources were involved in the study design; the collection, analysis, interpretation of data; the writing of the report, or the decision to submit the manuscript for publication.

## Results

### Loss of DYRK1A promotes cardiomyocyte cell cycle reentry and improves cardiac repair following MI in the adult heart

**Figure 1:**
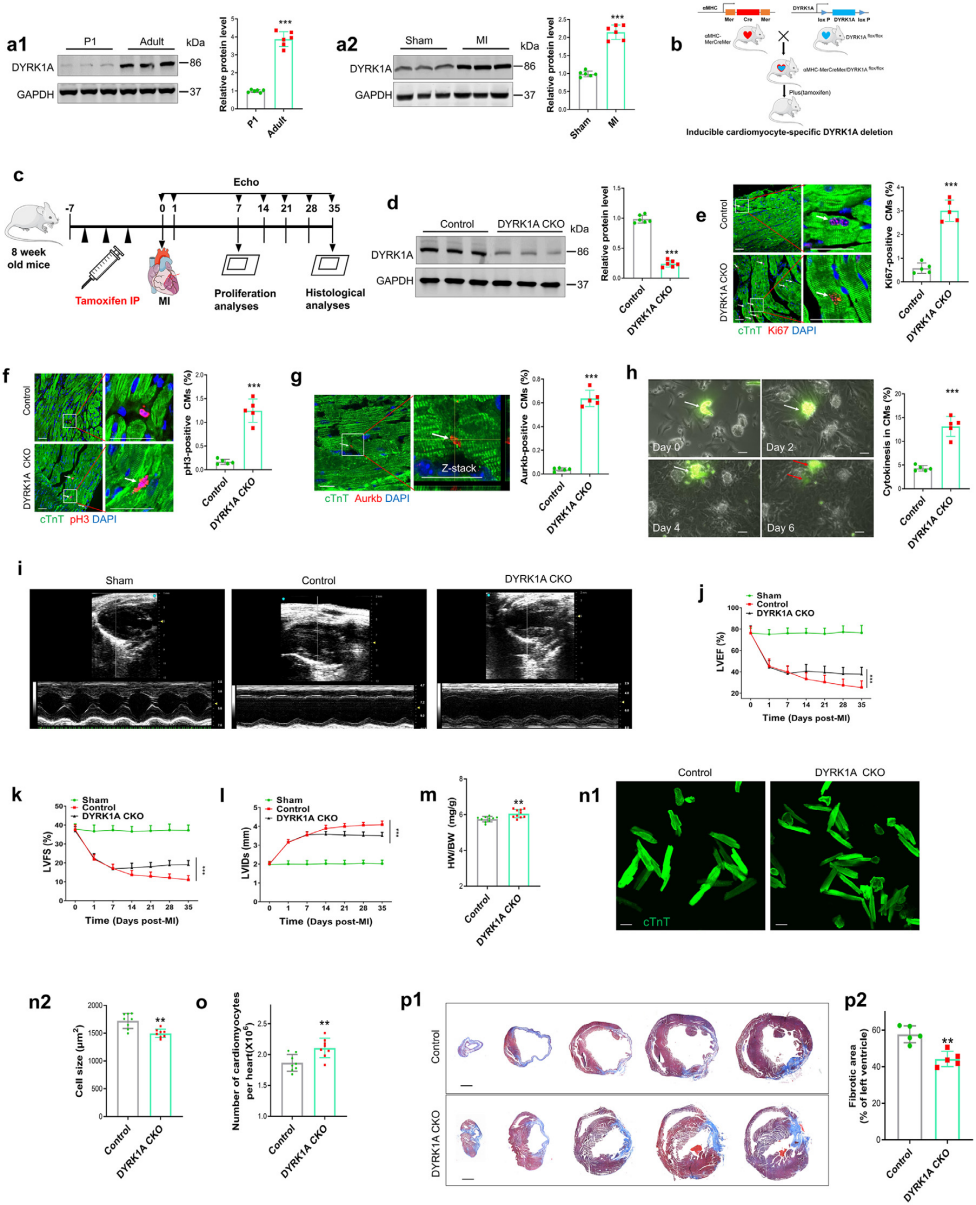
**Loss of DYRK1A promotes cardiomyocyte cell cycle reentry and improves cardiac repair following MI in adult hearts.** (**a**) DYRK1A expression in mouse heart during development and post-MI in adult mouse. (**a1**) Immunoblotting was used to detect DYRK1A expression in postnatal day 1 (P1) and adult mouse hearts (n=6 mice per group). Representative immunoblots (left) and quantification of protein levels (right) are shown. ^⁎⁎⁎^*P*<0.001 versus P1 group. (**a2**) Adult mice were subjected to MI or sham-operation, and protein expression of DYRK1A in the heart was determined at day 7 post-MI (*n*=6 mice per group). Representative immunoblots (left) and quantification of protein levels (right) are shown. ^⁎⁎⁎^*P*<0.001 versus sham group. (**b**) Schematic diagram of cardiomyocyte-specific deletion of DYRK1A (DYRK1A CKO). DYRK1A^flox/flox^ mice were crossed with α-MHC^MerCreMer^ mice to generate tamoxifen-inducible DYRK1A CKO mice. (**c**) Overview of the experimental setup. After the intraperitoneal injection (IP) of tamoxifen every other day thrice, control (αMHC^MerCreMer^) and α-MHC^MerCreMer^/DYRK1A^flox/flox^ mice (DYRK1A CKO) were subjected to MI. Echocardiography was performed every 7 days until removal of the heart, while proliferation and histological analyses were performed at 7 and 35 days, respectively. (**d**) Validation of deletion efficiency of DYRK1A in hearts of α-MHC^MerCreMer^/DYRK1A^flox/flox^ mice (*n*=6 mice per group). Representative immunoblots (left) and quantification of protein levels (right) are shown. ^⁎⁎⁎^*P*<0.001 versus control group. (**e–g**) Cardiomyocyte cell cycle activity regulated by DYRK1A *in vivo*. Immunofluorescence staining (left) and quantification (right) of Ki67- (**e**), pH3- (**f**) and Aurkb- (**g**) positive cardiomyocytes in the peri-infarct area in control or DYRK1A CKO mice are shown. Ki67-, pH3-, and Aurkb-positive cardiomyocytes are indicated by arrows. Scale bar=40 μm in **e** and **f**; scale bar=20 μm in **g**. CMs: cardiomyocytes (>10000 cardiomyocytes from five mice per group). ^⁎⁎⁎^*P*<0.001 versus control group. (**h**) Cardiomyocyte cytokinesis regulated by DYRK1A *in vitro*. Time-lapse microscopy imaging of live cells was performed after adult cardiomyocytes were isolated from 8-week-old control (αMHC^MerCreMer^) and α-MHC^MerCreMer^/DYRK1A^flox/flox^ mice (cardiomyocyte expression of GFP induced by infection of AAV9-GFP at neonatal stage) and co-cultured with primary neonatal rat cardiomyocytes for 7 days. Only division events with completed cytokinesis were counted. A representative image and quantification of cardiomyocyte cytokinesis are shown. Scale bars are 50 µm. CMs: cardiomyocytes (>3000 adult cardiomyocytes from five mice per group were traced). ^⁎⁎⁎^P<0.001 versus control group. (**i-l**) Echocardiographic analysis of cardiac function of adult control or DYRK1A CKO mice. Representative M-mode tracing images (day 35 post-MI) and quantitative analysis are shown. LVEF: left ventricular ejection fraction; LVFS: left ventricular fractional shortening; LVIDs: left ventricular internal dimension at systole (*n*=8 mice per group). ^⁎⁎⁎^*P*<0.001 versus control group. (**m**) Heart weight to body weight (HW/BW) ratio at day 35 post-MI (n=10). ^⁎⁎^*P*<0.01 versus control group. (**n**) Representative images (**n1**) and quantification (**n2**) of the cell size of cardiomyocytes dissociated at day 35 post-MI. Eighty to one hundred cells were randomly selected per heart (*n*=8). Scale bars are 50 µm. ^⁎⁎^*P*<0.01 versus control group. (**o**) Total number of dissociated cardiomyocytes per heart were determined at day 35 post-MI (*n*=8). ^⁎⁎^*P*<0.01 versus control group. (**p**) Fibrotic area of MI in hearts from control and DYRK1A CKO mice. Masson's trichrome staining (**p1**) and quantification (**p2**) of fibrotic scars of hearts were performed at day 35 post-MI (*n*=5 mice per group). Serial transverse sectioning was performed at 500 μm intervals from heart apex to the site of arterial occlusion. Scale bar=1 mm. ^⁎⁎^*P*<0.01 versus control group. All data are expressed as mean ± SD. The Two-tailed unpaired Student's t test was used for all statistical analysis except in **j**–**l**, where one-way ANOVA, followed by Tukey's multiple-comparison test were used.

To determine the relationship between DYRK1A and cardiomyocyte cell cycle activity, we quantified DYRK1A expression in mouse hearts during development and post-MI. We found that DYRK1A protein expression was higher in the adult mouse heart than that in the neonatal mouse heart, and was further increased post-MI in the adult mouse (Figure 1a). We further observed that DYRK1A was more extensively expressed in cardiomyocytes than that in cardiac fibroblasts, and was localized to a greater extent in the nucleus than that in the cytoplasm (Supplementary Fig. 1). The role of DYRK1A in cardiac repair was studied in mice with inducible cardiomyocyte-specific deletion of DYRK1A (DYRK1A CKO), generated by crossing DYRK1A^flox/flox^ with α-MHC^MerCreMer^ mice (Figure 1b). Cre was induced by tamoxifen in 8-week-old adult α-MHC^MerCreMer^/DYRK1A^flox/flox^ mice to delete DYRK1A from cardiomyocytes. Mice with cardiomyocyte-selective deletion of DYRK1A were then subjected to MI by permanent ligation of the left anterior descending coronary artery (Figure 1c–d). At day 7 post-MI, cardiomyocyte cell cycle activity, determined by measuring percentages of the cell cycling marker Ki67, G2-M progression marker phospho-histone H3 (pH3), and cleavage furrow-localized cytokinesis marker Aurora kinase B (*Aurkb*)-positive cardiomyocytes, in the peri-infarcted area, was significantly higher in DYRK1A CKO mice than that in control mice (Figure 1e–g). Time-lapse live-cell imaging of adult cardiomyocytes in culture further showed a higher cytokinesis rate of cardiomyocytes isolated from DYRK1A CKO mice than those from control mice (Figure 1h). Cardiac function was measured weekly by echocardiography. As shown in Figure 1i–l, MI-induced cardiac dysfunction, evidenced by decreased LVEF and fractional shortening (LVFS) and increased left ventricular internal dimension at systole (LVIDs), was improved in DYRK1A CKO mice, as compared with that in control mice. The improvement in cardiac function in DYRK1A CKO mice, relative to control mice, was accompanied by an increase in the heart weight to body weight ratio, cardiomyocyte cell cycle activity, and cardiomyocyte number per heart, and a decrease in cardiomyocyte cell size and fibrotic area in DYRK1A CKO mice (Figure 1m–p, Supplementary Fig. 2). These findings indicate that the loss of DYRK1A promotes adult cardiomyocyte cell cycle reentry, and suggest that DYRK1A could be a target to promote cardiac repair post-MI.

### DYRK1A knockdown induces transcriptional reprogramming of networks that govern cardiomyocyte cell cycle activation

**Figure 2:**
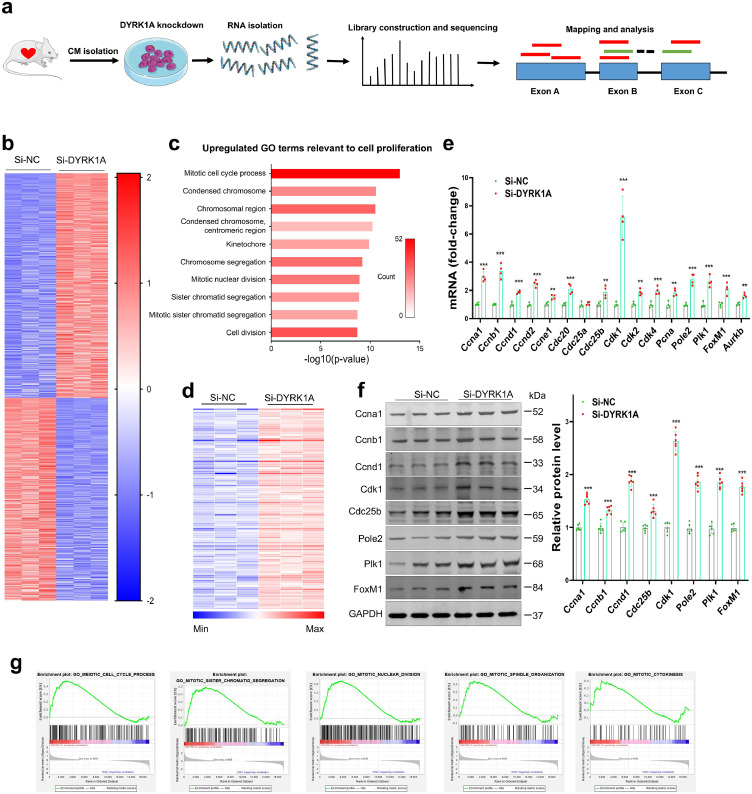
**DYRK1A knockdown induces transcriptional reprogramming of networks that govern cardiomyocyte cell cycle activation.** (**a**) Schematic workflow of transcriptome analysis. (**b**) Heatmap profile of RNA-sequencing (RNA-seq) analysis to show differentially expressed genes in primary neonatal cardiomyocytes transfected with scramble (si-NC) or DYRK1A siRNA (si-DYRK1A) (*n*=3 samples per group). (**c**) Gene ontology (GO) analysis of upregulated genes in si-DYRK1A cardiomyocytes (relative to si-NC group) show multiple, significantly enriched GO terms relevant to cell proliferation. (**d**) Heatmap profile of upregulated cell cycle regulators (relative to si-NC group). (**e–f**) Validation of selected cell cycle regulators from RNA-seq by qRT-PCR and immunoblotting (*n*=4 samples per group in **e**; *n*=6 samples per group in **f**). Representative immunoblots (left) and quantification of protein levels (right) are shown in **f**. (^⁎⁎^*P*<0.01, ^⁎⁎⁎^*P*<0.001) versus si-NC group. (**g**) The dynamic bias of differentially expressed genes in si-NC and si-DYRK1A cardiomyocytes. Gene set enrichment analysis was used to analyze clusters of genes that regulate cell cycle. For **e–f**, all bars express mean ± SD and data are analyzed using the two-tailed unpaired Student's t test.

Consistent with *in vivo* studies, knockdown of DYRK1A expression by siRNA increased cell cycle activity in both human iPS-derived cardiomyocytes and rat neonatal cardiomyocytes (Supplementary Figs. 3–4). To explore how DYRK1A affects cardiomyocyte cell cycle reentry, we performed transcriptome analysis via RNA-seq, using total RNAs from rat neonatal cardiomyocytes in primary cultures transfected with si-DYRK1A or si-NC (Figure 2a). Heatmap and volcano plot analyses showed that the expressions of 1548 genes were altered, with 814 genes upregulated and 734 genes downregulated in si-DYRK1A-transfected cardiomyocytes (Figure 2b, Supplementary Fig. 5a, Supplementary Table 1). The box plots of RNA-seq read counts in each sample showed that the samples were comparable in their overall variability, which helped to ensure that the differences detected were not the effect of skewed data (Supplementary Fig. 5b). GO and KEGG analyses showed that DYRK1A knockdown-induced the transcriptional reprogramming of networks related to cell proliferation, and activated genes involved in DNA replication, mitotic cell cycle process, chromosome segregation, mitotic nuclear division, and cell division (Figure 2c, Supplementary Figs. 5c–d). Among the 814 upregulated genes, 151 enriched in cell proliferation-related GO terms were screened, while some representative genes and proteins were further validated by qRT-PCR and immunoblotting, respectively (Figure 2d–f). Gene set enrichment analyses further indicated that DYRK1A knockdown in cardiomyocytes leads to a dynamic bias toward positive regulation of cell cycle activation (Figure 2g, Supplementary Fig. 5e). These results suggests that DYRK1A regulates cardiomyocyte cycling by triggering transcriptional reprogramming of a large number of genes that drive cardiomyocyte cell cycle activation.

### DYRK1A knockdown increases the deposition of H3K27ac and H3K4me3 on promoters of cell cycle genes in cardiomyocytes

**Figure 3:**
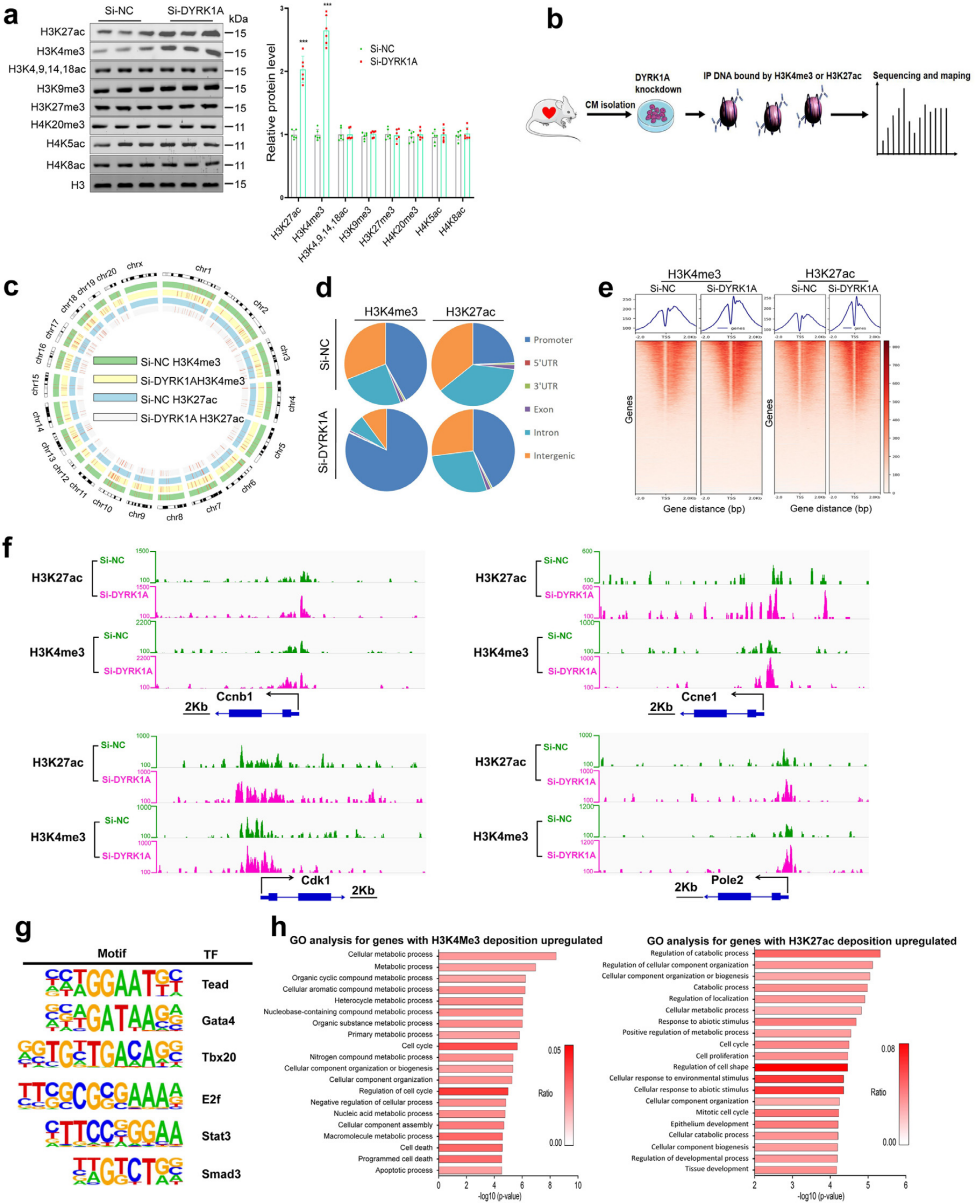
**DYRK1A knockdown increases deposition of H3K27ac and H3K4me3 on promoters of cell cycle genes in cardiomyocytes.** (**a**) Changes of histone modifications induced by DYRK1A knockdown *in vitro*. Immunoblotting was performed to show global changes of histone modifications, using proteins extracted from cardiomyocytes in primary culture transfected with scramble (si-NC) or DYRK1A siRNA (si-DYRK1A) for 48 h (*n*=6 samples per group). Representative immunoblots (left) and quantification of protein level (right) are shown. ^⁎⁎⁎^P<0.001 versus si-NC group. (**b**) Schematic workflow of H3K4me3 and H3K27ac ChIP-seq. (**c**) Distribution of H3K4me3 or H3K27ac ChIP-seq peaks in chromosomes. The positions of H3K4me3 and H3K27ac enrichment were aligned, according to chromosome position in the outermost circle. (**d**) Pie chart of the genomic distribution of H3K4me3 or H3K27ac binding peaks, including promoters, 5′ UTR, 3′ UTR, exons, introns, and intergenic regions. (**e**) ChIP-seq density heatmaps in cardiomyocytes transfected with si-NC or si-DYRK1A. Signals were ranked by H3K4me3 and H3K27ac read intensity within ±2k bp of peak from the transcription start sites. (**f**) Visualization of H3K4me3 or H3K27ac ChIP-seq data tracks. Cell cycle genes were selected, and integrative genomics viewer screen shots were used to show representative peaks. (**g**) Motif analysis showed binding motifs for representative transcriptional factors functioning in activating cell cycle activity and cardiomyocyte proliferation. (**h**) GO analysis for genes with increased H3K4me3 (left) and H3K27ac (right) deposition on promoter in si-DYRK1A-treated cardiomyocytes. For **a**, all bars express mean ± SD and data are analyzed using two-tailed unpaired Student's t test.

While a large number of genes were regulated by DYRK1A, few signaling pathways regulating the cell cycle were activated by DYRK1A knockdown (Supplementary Fig. 5d). We thus hypothesized that DYRK1A regulation of cardiomyocyte cell cycle activation may occur via epigenetic mechanisms such as regulation of histone modification. Our screening studies show that DYRK1A knockdown increased H3K27ac and H3K4me3 levels (Figure 3a). This leads to open and accessible chromatin, which facilitates the binding of transcription factors to gene promoters and activation of transcription. Therefore, we performed chromatin IP followed by sequencing (ChIP-seq) using DNA fragments captured by H3K4me3 or H3K27ac antibodies in si-NC- and si-DYRK1A-transfected cardiomyocytes (Figure 3b). As expected, H3K4me3 and H3K27ac occupied the chromatin globally (Figure 3c), and DYRK1A knockdown markedly enhanced the deposition of H3K4me3 and H3K27ac in promoter regions (Figure 3d). Further analysis of the profiles of ChIP-seq peaks in the vicinity of all transcriptional start sites (TSSs) revealed an increase in enrichment of both H3K4me3 and H3K27ac surrounding TSSs in DYRK1A knockdown cardiomyocytes (Figure 3e).

Interestingly, inhibition of DYRK1A led to the enrichment of H3K27ac and/or H3K4me3 in the promoters of cell cycle genes, including cyclin A1 (*Ccna1*), cyclin B1 (*Ccnb1*), cyclin D1 (*Ccnd1*), cyclin E1 (*Ccne1*), cyclin-dependent kinase 1 (*Cdk1*), DNA polymerase epsilon 2 (*Pole2*), *Aurkb*, and proliferating cell nuclear antigen (*Pcna*) (Figure 3f, Supplementary Fig. 6), which is consistent with the increased gene expression shown in Figure 2. Motif analyses of the H3K4me3 and H3K27ac ChIP-seq peaks further showed binding motifs for transcription factors involved in promoting cardiomyocyte cell cycle activation, including TEA domain transcription factor,^16^ GATA binding protein 4,^17^ and T-Box Transcription Factor 20^18^ (Figure 3g), indicating that DYRK1A loss-of-function in cardiomyocytes leads to open and accessible chromatin to facilitate the transcription of these transcription factors. In line with the transcriptomic results, GO analysis of genes with increased occupation of H3K4me3 and H3K27ac in the promoter region revealed enrichment of categories, including cell proliferation, cell cycle, and mitotic cell cycle (Figure 3h), indicating that the change in the H3K4me3 and H3K27ac epigenetic landscape may be the cause of the transcriptional activation of cell cycle genes and cell cycle regulators induced by DYRK1A knockdown.

### DYRK1A inhibition decreases KAT6A and WDR82 phosphorylation to induce H3K27ac and H3K4me3 modification in cardiomyocytes

**Figure 4:**
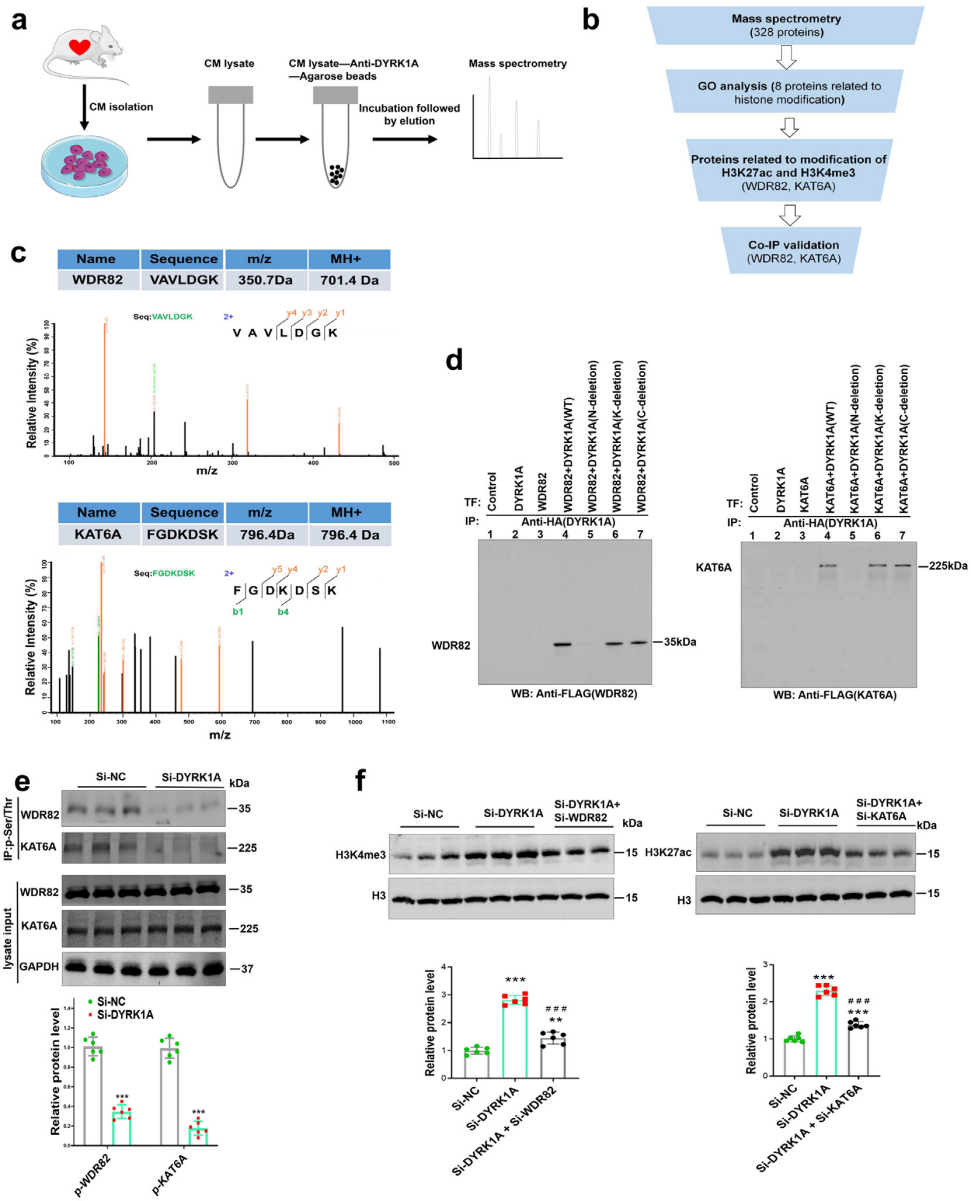

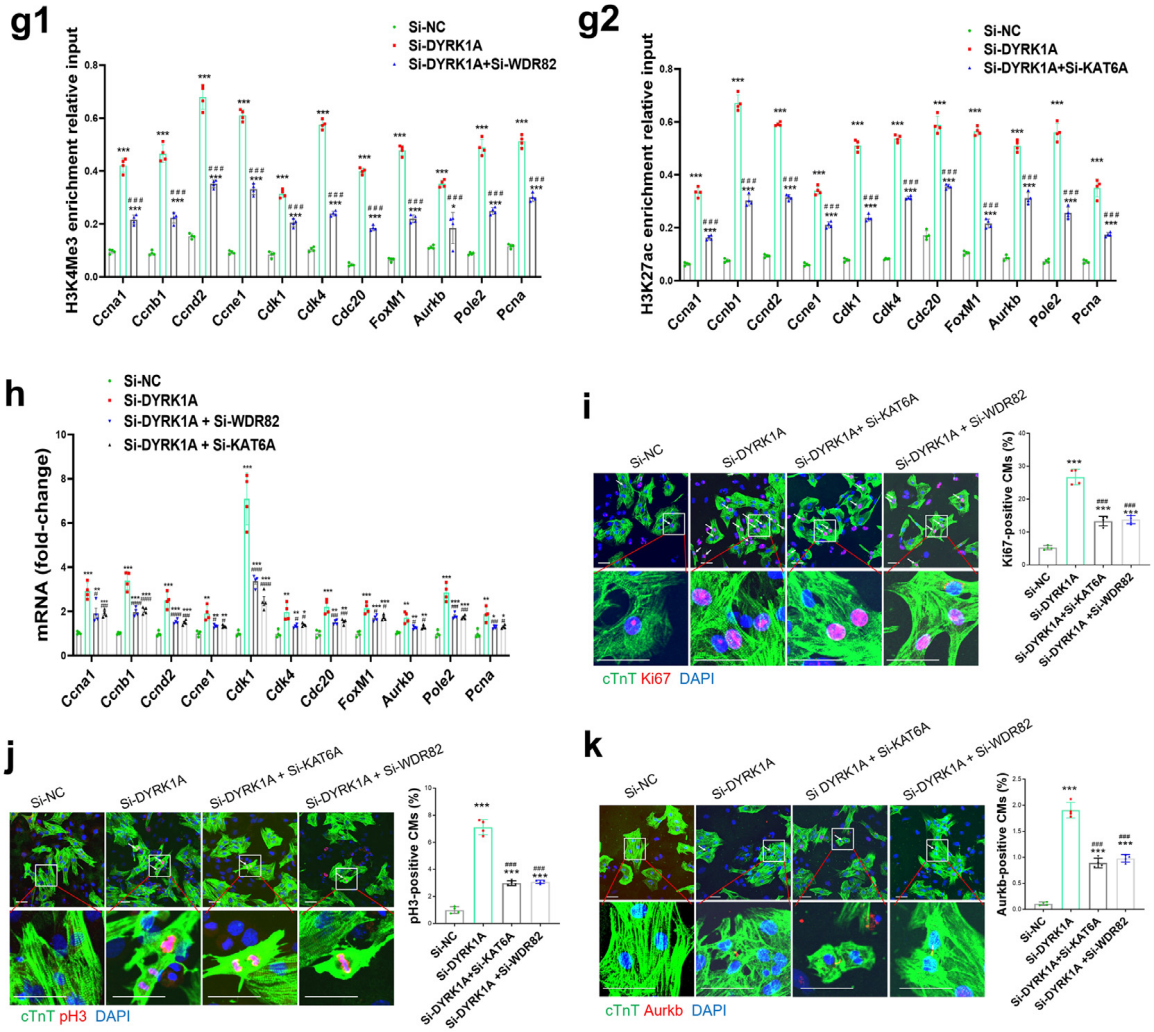
**Inhibition of DYRK1A decreases KAT6A and WDR82 phosphorylation to induce H3K27ac and H3K4me3 modification in cardiomyocytes.** (**a**) Schematic workflow of immunoprecipitation (IP)-mass spectrometry to identify binding partners of DYRK1A in cardiomyocytes. (**b**) Selection strategy of candidate effectors for DYRK1A in regulating H3K4me3 and H3K27ac enrichments from mass spectrometry profiling. (**c**) Mass spectrogram profile of WDR82 (upper figure) and KAT6A (lower figure). MS2 spectra of WDR 82 and KAT6A peptides are shown, and the intensity of peptide fragments is plotted against the mass-to-charge ratio (m/z). (**d**) Validation of the interaction of DYRK1A with WDR82 or KAT6A, and determination of DYRK1A domain responsible for the binding. The binding of HA-tagged full-length DYRK1A (lane 4), or its deletion mutants (N-terminal domain deletion, lane 5; kinase catalytic domain deletion, lane 6; C-terminal domain deletion, lane 7) with FLAG-tagged WDR82 or KAT6A was examined by co-transfection/co-IP experiments. Lane 1, control without transfection; lane 2, control with only DYRK1A transfection. Lane 3 control with only WDR82 or KAT6A transfection. TF: transfection. IP: immunoprecipitation. WB: western blot. (**e**) Phosphorylation of WDR82 and KAT6A regulated by DYRK1A. Co-IP assays were used to show the effect of DYRK1A knockdown on the phosphorylation of WDR82 and KAT6A. Total phosphorylation (Ser and Thr) antibody was used for IP and WDR82 or KAT6A antibody was used for immunoblotting. Co-IP was performed 48 h after scramble (si-NC) or DYRK1A siRNA (si-DYRK1A) transfection (*n*=6 samples per group). Representative immunoblots (upper) and quantification of protein levels (lower) are shown. IP: immunoprecipitation. ^⁎⁎⁎^*P*<0.001 versus si-NC group. (**f**) Effect of WDR82 and KAT6A on the regulatory role of DYRK1A on global changes of H3K4me3 and H3K27ac measured by immunoblotting (*n*=6 samples per group). Additionally, representative immunoblots (upper) and quantification of protein levels (lower) are shown. (^⁎⁎^*P*<0.01, ^⁎⁎⁎^*P*<0.001) versus si-NC group; ^###^*P*<0.001 versus si-DYRK1A group. (**g**) Effect of WDR82 and KAT6A on regulatory role of DYRK1A in H3K4me3 (**g1**) and H3K27ac (**g2**) deposition on promoters of cell cycle regulatory genes. ChIP-qPCR assays were performed (*n*=4 samples per group). (**h**) Effect of WDR82 and KAT6A on the regulatory role of DYRK1A in the expression of cell cycle regulatory genes. qPCR assays were performed (*n*=4 samples per group). (**i–k**) Immunofluorescence staining and quantification of Ki67- (**i**), pH3- (**j**) and Aurkb- (**k**) positive cardiomyocytes to show the effects of WDR82 or KAT6A knockdown on the regulatory role of DYRK1A in cardiomyocyte cell cycle activation. CMs: cardiomyocytes (more than 10,000 cardiomyocytes from four independent experiments per group). (*P<0.05, ^⁎⁎⁎^*P*<0.01, ^⁎⁎⁎^*P*<0.001) versus si-NC group; (^#^*P*<0.05, ^##^*P*<0.01, ^###^*P*<0.001) versus si-DYRK1A group. All data are expressed as mean ± SD. Two-tailed unpaired Student's t test was used for all statistical analysis in **e** and one-way ANOVA followed by Tukey's multiple-comparison test was used in **f–k**.

While the preceding data showed that DYRK1A can regulate H3K27ac and H3K4me3 modifications, DYRK1A, a serine/threonine kinase, cannot directly regulate lysine methylation or acetylation. We deduced that DYRK1A may interact with other molecules to affect H3K27ac and H3K4me3 modifications. To search for potential DYRK1A-interacting molecules, unbiased mass spectrometry analysis was performed on the co-immunoprecipitated DYRK1A complexes in cardiomyocytes (Figure 4a–b, Supplementary Table 2). Through procedural filtering, we found two potential DYRK1A-interacting proteins: WDR82, a component of the H3K4me3 methyltransferase complex, and KAT6A, a member of histone acetyltransferases (Figure 4c). DYRK1A is composed of three major domains: C-terminal tail (C), kinase catalytic domain (K), and N-terminal domain (N). To validate the interaction of DYRK1A with WDR82 and KAT6A and determine the DYRK1A domain responsible for the binding, we constructed plasmids encoding HA-tagged full-length DYRK1A and mutated DYRK1A with deletion of the N, K, or C termini and FLAG-tagged full-length WDR82 or KAT6A. Co-expression/co-IP assay validated the interaction of DYRK1A with WDR82 and KAT6A, and the N-terminal deletion mutation of DYRK1A abolished its interactions with KAT6A and WDR82, while the K domain or C-terminal tail deletion exerted no effect on the interactions (Figure 4d), indicating the essential role of the N-terminal domain of DYRK1A in the KAT6A and WDR68 binding. Although the expression of WDR82 and KAT6A in the hearts of mice with MI was not different from that in sham mice (Supplementary Fig. 7), we found that DYRK1A knockdown decreased WDR82 and KAT6A phosphorylation *in vitro* (Figure 4e), a modification that may increase their activity in neonatal cardiomyocytes. Conversely, wild-type DYRK1A overexpression decreased cardiomyocyte cell cycle activity, increased WDR82 and KAT6A phosphorylation, and inhibited H3K4me3 and H3K27ac occupancy on the promoters of cell cycle genes as well as the expression of these genes, whereas overexpression of kinase-dead DYRK1A-K188R, with a mutation in its adenosine-triphosphate (ATP)-binding site to make it enzymatically inactive, exerted no effect (Supplementary Fig. 8). Thus, this indicates that kinase activity was required for the DYRK1A-mediated regulation of cardiomyocyte cycling. Moreover, knockdown of WDR82 or KAT6A by siRNA blocked the DYRK1A deficiency-induced increase in H3K4me3 and H3K27ac expressions and occupancy on the promoters of cell cycle genes (Figure 4f–g, Supplementary Fig. 9). Consequently, it blocked DYRK1A deficiency-induced cardiomyocyte cell cycle activation, as determined by checking cell cycle gene expression and the percentages of Ki67-, pH3-, and *Aurkb*-positive cardiomyocytes (Figure 4h–k). Taken together, these results indicate that WDR82 and KAT6A are indispensable for DYRK1A in regulating H3K4me3 and H3K27ac, and subsequent cardiomyocyte cell cycle activation.

### Pharmacological inhibition of DYRK1A promotes cardiac repair post-MI in adult mice

**Figure 5:**
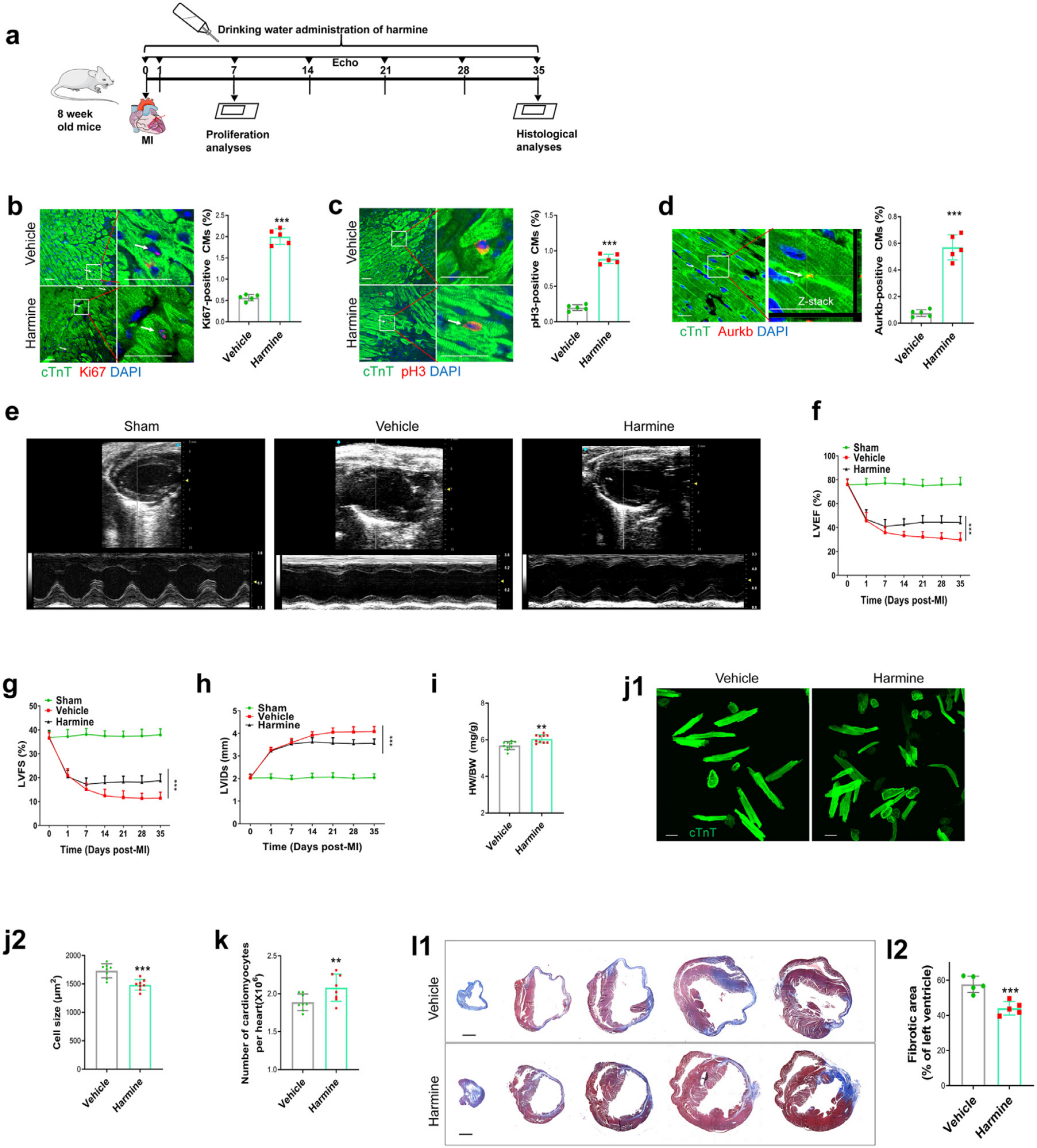
**Pharmacological inhibition of DYRK1A promotes cardiac repair after MI in adult hearts.** (**a**) Schematic diagram of the experimental design. Harmine was dissolved in drinking water at a concentration of 0.05 mg/ml. After MI or sham surgery, echocardiography was performed every 7 days until the removal of the hearts. Proliferation and histological analyses were performed at 7 and 35 days, respectively. (**b–d**) Cardiomyocyte cell cycle activity induced by harmine *in vivo*. Immunofluorescence staining (left) and quantification (right) of Ki67- (**b**), pH3- (**c**), and Aurkb- (**d**) positive cardiomyocytes in the peri-infarct area in vehicle- or harmine-treated mice. Ki67-, pH3-, and Aurkb-positive cardiomyocytes are indicated by arrows. Scale bar=40 μm in **b** and **c**; scale bar=20 μm in **d**. CMs: cardiomyocytes (>10000 cardiomyocytes from five mice per group). ^⁎⁎⁎^*P*<0.001 versus vehicle group. (**e–h**) Echocardiographic analysis of cardiac function from vehicle- or harmine-treated adult mice. Representative M-mode tracing images (day 35 post-MI) and quantitative analysis are shown. LVEF: left ventricular ejection fraction; LVFS: left ventricular fractional shortening; LVIDs: left ventricular internal dimension at systole (*n*=8 mice per group). ^⁎⁎⁎^*P*<0.001 versus vehicle group. (**i**) Heart weight to body weight (HW/BW) ratio were calculated at day 35 post-MI (*n*=10). ^⁎⁎^*P*<0.01 versus vehicle group. (**j**) Representative images (**j1**) and quantification (**j2**) of the cell size of cardiomyocytes dissociated at day 35 post-MI. Eighty to one hundred cells were randomly selected per heart (*n*=8). Scale bars are 50 µm. ^⁎⁎⁎^*P*<0.001 versus vehicle group. (**k**) The total number of dissociated cardiomyocytes per heart were determined at day 35 post-MI (*n*=8). ^⁎⁎⁎^*P*<0.01 versus vehicle group. (**l**) Fibrotic area of infarcted hearts from vehicle- or harmine-treated mice. Masson's trichrome staining (**l1**) and quantification (**l2**) of fibrotic scar of hearts at day 35 post-MI. Serial transverse sectioning was performed at 500 μm intervals from the heart apex to the site of arterial occlusion. Scale bar=1 mm (*n*=5 mice per group). ^⁎⁎⁎^*P*<0.001 versus vehicle group. All data are expressed as the mean ± SD. The two-tailed unpaired Student's t test was used for all statistical analysis except in **f**–**h**, where one-way ANOVA followed by Tukey's multiple-comparison test were used.

Given that DYRK1A deletion showed a beneficial effect on cardiomyocyte cell cycle reentry and post-injury repair, we determined the clinical relevance of targeting DYRK1A via pharmacological inhibition of its activity using harmine, ID-8, and INDY, three commonly used small-molecule inhibitors of DYRK1A kinase activity. We found that the stimulatory effect of the three DYRK1A inhibitors on cardiomyocyte cell cycle activation was concentration-dependent, with their effect peaking at 5 μM in rat primary neonatal cardiomyocytes (Supplementary Fig. 10). We selected harmine as the DYRK1A inhibitor in subsequent studies because it exerted the strongest cycling-promoting effect among the three. Similar to DYRK1A knockdown, harmine reduced WDR82 and KAT6A phosphorylation in neonatal cardiomyocytes (Supplementary Fig. 11). We then determined if the *in vitro* experiments could be replicated *in vivo* by treating adult mice for one week with harmine dissolved in drinking water at various concentrations (0.01 to 0.1 mg/ml). Given that the mice weighed approximately 20 g and the daily water consumption was approximately 5 ml, the estimated final daily harmine dosage ranged from 2.5 to 25 mg/kg body weight. The cardiomyocyte cycling-promoting effect of harmine was detected at 0.01 mg/ml and peaked at 0.05 mg/ml, without exerting an effect on cardiac function in the basal state (Supplementary Fig. 12). Therefore, in subsequent studies, we chose 0.05 mg/ml harmine as the concentration for treatment, with an estimated dose of 12.5 mg/kg/d. After administering harmine for one week in mice with MI, cardiomyocyte cell cycle reentry was observed, indicated by higher percentages of Ki67, pH3, and Aurkb-positive cardiomyocytes (Figure 5a–d). We also observed an improvement in cardiac function after the administration of harmine for 5 weeks, as seen in an increase in LVEF and LVFS and decrease in LVIDs (Figure 5e–h). Furthermore, the improvement in cardiac function in harmine-treated mice was also accompanied by an increase in heart weight to body weight ratio, cardiomyocyte cell cycle activity, and cardiomyocyte number per heart, and decrease in cardiomyocyte cell size and fibrotic area (Figure 5i-l, Supplementary Fig. 13). These results demonstrate that DYRK1A may be an important therapeutic target in adult hearts with MI.

## Discussion

Considerable advances have been made in understanding the regulatory mechanisms of cardiomyocyte proliferation, and many beneficial targets, including the extracellular matrix proteins Agrin, neuregulin-1, and Hoxb13, have been identified.^19^, ^20^, ^21^ However, the clinical application of these findings requires genetic intervention or the delivery of recombinant proteins, which is not yet clinically feasible because of low organ specificity, deleterious immunogenic effects, and other drawbacks, including gene toxicity and tumorigenesis.^22^^,^^23^ Therefore, factors that have high efficacy in regulating cardiac repair and pharmacological targetability for drug development are urgently needed. Our study, identified a key role played by DYRK1A in the regulation of cardiomyocyte cell cycle reentry and showed that either pharmacological inhibition or genetic deletion of DYRK1A enhanced cardiac repair in adult mouse hearts with MI. Thus, targeting DYRK1A may represent a therapeutic strategy for injured hearts, with promising clinical potential.

The regulation of cardiomyocyte cycling is a complex process. An increasing number of experiments show that multiple cell cycle genes participate in this process, with key cell cycle regulatory genes inactivating during mammalian heart development, especially in the adult stage. The activation of silent genes in adult heart is an ongoing area of investigation. Viral vector-mediated overexpression of specific combinations of multiple cell cycle genes, including CDK1, CDK4, cyclin B1, and cyclin D1, can efficiently unleash the proliferative potential of adult cardiomyocytes.^24^ However, owing to the obvious limitation of the use of viruses in clinical situations, focusing on epigenetic modifications to simultaneously modulate the expression of various genes may be preferable.

Many studies have demonstrated the critical role of histone modifications in regulating diverse cellular processes, especially cell proliferation.^25^ Distinct modes of H3K4me3, H3K27ac, and H3K27me3 in juvenile and adult human β cells are associated with the transcriptional regulation of age-dependent genes governing proliferation.^26^ In the heart, expression of H3K4me3 and H3K27ac (associated with active transcription) decreases, while that of H3K27me3 (present in inactive or silenced genomic loci) increases during development.^27^ Therefore, increasing chromatin accessibility through remodeling of these histone markers may represent a useful strategy to regain the active expression of cell cycle regulators driving cardiomyocyte cell cycle reentry. In support of this hypothesis, we observed an increase in expression of H3K4me3 and H3K27ac in the promoters of cell cycle regulators, which may explain the increase in the expression of these genes and cardiomyocyte cell cycle activation induced by DYRK1A knockdown. Thus, discovering more histone codes may shed new mechanistic light on the DYRK1A-mediated regulation of cardiomyocyte cycling and cardiac repair.

As a Down syndrome-associated kinase, DYRK1A functions by interacting with and phosphorylating its substrates, including NFAT,^9^^,^^28^ RNA polymerase II,^29^ and the MuvB-like protein LIN52.^30^ In our report, using unbiased mass spectrometry analysis, we found two previously unreported DYRK1A-interacting proteins, WDR82 and KAT6A. WDR82 is a specific component of the SETD1A histone H3-Lys4 methyltransferase complex that recruits the complex to transcription start sites; hence, a defect in WDR82 causes SETD1A dysfunction and loss of H3K4me3.^31^ KAT6A belongs to the MYST family of acetyltransferases, that acylate both histone H3 and non-histone proteins.^32^ The essential roles of KAT6A and WDR82 in regulating cell proliferation, via modification of histone lysine residues, have been reported in some cell types, such as B-cell progenitors and hematopoietic cells.^32^, ^33^, ^34^ Importantly, DYRK1A regulates the phosphorylation of WDR82 and KAT6A. Intriguingly, silencing these two effectors markedly blunted the DYRK1A knockdown-induced elevation in H3K4me3 and H3K27ac deposition on gene promoters of cell cycle regulators, thus abolishing cardiomyocyte cell cycle activation. In contrast to this epigenomic mechanism by which DYRK1A regulates cardiomyocyte cycling, DYRK1A regulates pancreatic β-cell proliferation mainly via calcineurin-NFAT signaling.^9^

Harmine, a β-carboline alkaloid, is the most commonly used DYRK1A inhibitor. It decreases the kinase activity of DYRK1A via the competitive inhibition of ATP-binding to the DYRK1A kinase pocket. Many studies show broad-spectrum beneficial effects of harmine such as induction of regeneration of adult human β cells,^9^^,^^35^ inhibition of glioblastoma growth,^7^ amelioration of cognitive impairment,^36^ and alleviation of cardiovascular diseases, including atherosclerosis,^37^ and cardiac hypertrophy.^38^ In our study, harmine induced substantial cardiomyocyte cycling and cardiac repair post-MI in adult mice. Although harmine has a high affinity for DYRK1A, it also inhibits other kinases at higher concentrations, including other members of the DYRK family, cdc-like kinases, and monoamine oxidase A.^39^ This may explain the observation that a high concentration of harmine can reduce the stimulatory effect on cardiomyocyte cycling *in vitro*. However, these drawbacks can be overcome via organ-selective targeting nanoparticles as drug carrier^40^ or synthesizing non-toxic harmine analogs.^41^ Indeed, the robust action of DYRK1A in multiple diseases, especially in pancreatic β cell regeneration, is an incentive to develop an increasing number of potentially therapeutic DYRK1A inhibitors with better target specificity and reduced toxicity.^42^^,^^43^ Thus, clinical applications of the cardiac repair effect of DYRK1A is a possibility in the near future.

**Figure 6:**
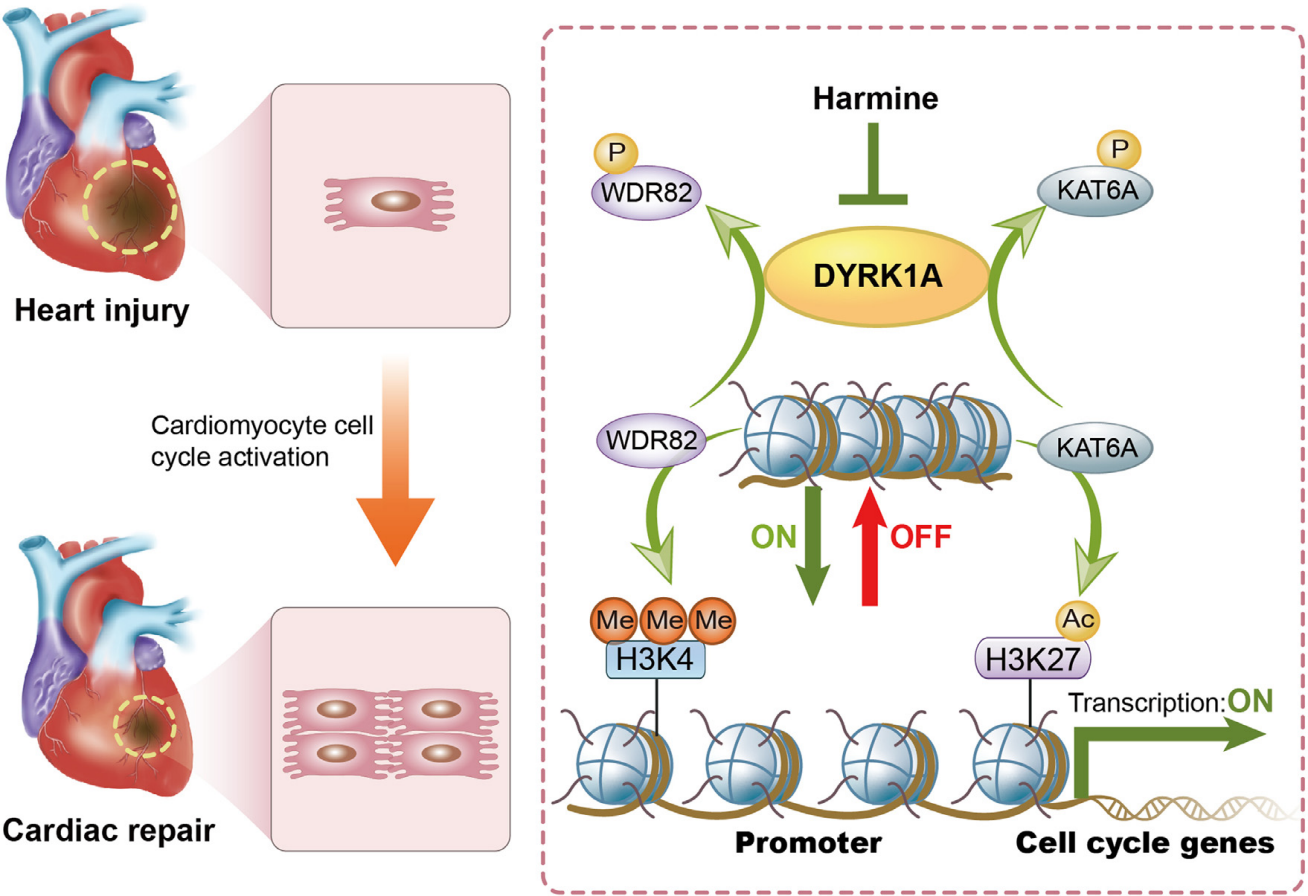
**Graphic abstract**. DYRK1A phosphorylates WDR82 and KAT6A to reduce deposition of H3K4me3 and H3K27ac on the promoters of cell cycle genes, thereby limiting their transcriptional activation and inhibiting cardiomyocyte cell cycle activity. Either pharmacological inhibition or genetic deletion of DYRK1A promotes cardiomyocyte cell cycle activation and cardiac repair in adult heart post-MI.

As summarized in Figure 6, our study provides evidence that DYRK1A is a promising target for the promotion of cardiomyocyte cell cycle reentry and cardiac repair after heart injury, especially MI. We also uncovered the underlying mechanisms, that is, DYRK1A, via phosphorylation of WDR82 and KAT6A, reduces the deposition of H3K4me3 and H3K27ac on promoters of cell cycle regulators, limiting their transcriptional activation and repressing cardiomyocyte cell cycle activation. This finding has translational significance because pharmacological inhibition of DYRK1A by harmine promotes cardiac repair after MI. However, rescue experiments with phospho-WDR82 or phospho-KAT6A inhibitors are needed to confirm the effect of WDR82 and KAT6A phosphorylation on the DYRK1A-mediated regulation of cardiomyocyte cycling and cardiac repair. In addition, because of the concentration-dependent and possible off-target effects of harmine, further studies on the optimal timing, dosage, and tissue-specific delivery of harmine and the development of safe and selective DYRK1A inhibitors are warranted.

## Contributors

Chunyu Zeng, Zaicheng Xu, Gengze Wu, and Cong Lan conceived the project and designed the experiments. Cong Lan, Caiyu Chen, Shuang Qu, Nian Cao, Hao Luo, Cheng Yu, Na Wang, Yuanzheng Xue, Xuewei Xia, Chao Fan and Hongmei Ren conducted experiments. Cong Lan wrote the manuscript. Cong Lan, Pedro A. Jose, Chunyu Zeng, Zaicheng Xu, and Gengze Wu analyzed and discussed the results. Pedro A. Jose, Chunyu Zeng, Zaicheng Xu, and Gengze Wu revised the manuscript. All authors read and verified the underlying data and approved the final manuscript.

## Data sharing statement

The data supporting the findings of this study are available from the corresponding author upon reasonable request.

## Declaration of interests

The authors declare no competing interests.

## Appendix Supplementary materials

Image, application 1

Image, application 2

Image, application 3

Image, application 4

Image, application 5

Image, application 6
