# Supplementary material for: Inhibition of DYRK1A, via histone modification, promotes cardiomyocyte cell cycle activation and cardiac repair after myocardial infarction
Source: eBioMedicine. 2022 Jul 8;82:104139. doi: 10.1016/j.ebiom.2022.104139 (PMC9278077; doi:10.1016/j.ebiom.2022.104139)
Supplement: Supplementary file 1 [file mmc1.docx]

**Captions for supplementary materials**

**Supplementary figures**

Supplementary Fig. 1: DYRK1A expression in cardiomyocytes and cardiac fibroblasts.

Supplementary Fig. 2: Loss of DYRK1A promotes cardiomyocyte cell cycle activation following MI in adult hearts.

Supplementary Fig. 3: DYRK1A knockdown promotes cell cycle activation of human induced pluripotent stem cell (iPS)-derived cardiomyocytes *in vitro*.

Supplementary Fig. 4: DYRK1A knockdown promotes primary neonatal cardiomyocyte cell cycle activation *in vitro*.

Supplementary Fig. 5: RNA-sequencing analysis reveals that DYRK1A knockdown leads to activation of cardiomyocyte cell cycle activity.

Supplementary Fig. 6: Distribution of H3K4me3 and H3K27ac on representative genes (cell cycle genes and regulators).

Supplementary Fig. 7: WDR82 and KAT6A expression in hearts with MI.

Supplementary Fig. 8: DYRK1A regulation of cardiomyocyte cell cycle activity is dependent on its kinase activity.

Supplementary Fig. 9: Knockdown of WDR82 and KAT6A with the corresponding siRNA in cardiomyocytes.

Supplementary Fig. 10: Inhibition of DYRK1A promotes cardiomyocyte cell cycle activation *in vitro*.

Supplementary Fig. 11: Inhibition of DYRK1A by harmine reduces WDR82 and KAT6A phosphorylation.

Supplementary Fig. 12: Harmine promotes cell cycle activation of cardiomyocyte in adult mice in basal state.

Supplementary Fig. 13: Pharmacological inhibition of DYRK1A promotes cardiomyocyte cell cycle activation following MI in adult hearts.

**Supplementary Tables**

Supplementary Table 1: The list of differentially expressed genes after DYKR1A knockdown in cardiomyocytes

Supplementary Table 2. DYRK1A-interacting proteins detected by mass spectrometry analysis.

**Full Western Blots**
