# Supplementary material for: Inhibition of DYRK1A, via histone modification, promotes cardiomyocyte cell cycle activation and cardiac repair after myocardial infarction
Source: eBioMedicine. 2022 Jul 8;82:104139. doi: 10.1016/j.ebiom.2022.104139 (PMC9278077; doi:10.1016/j.ebiom.2022.104139)
Supplement: Supplementary file 2 [file mmc2.docx]

**Supplemental Figures**


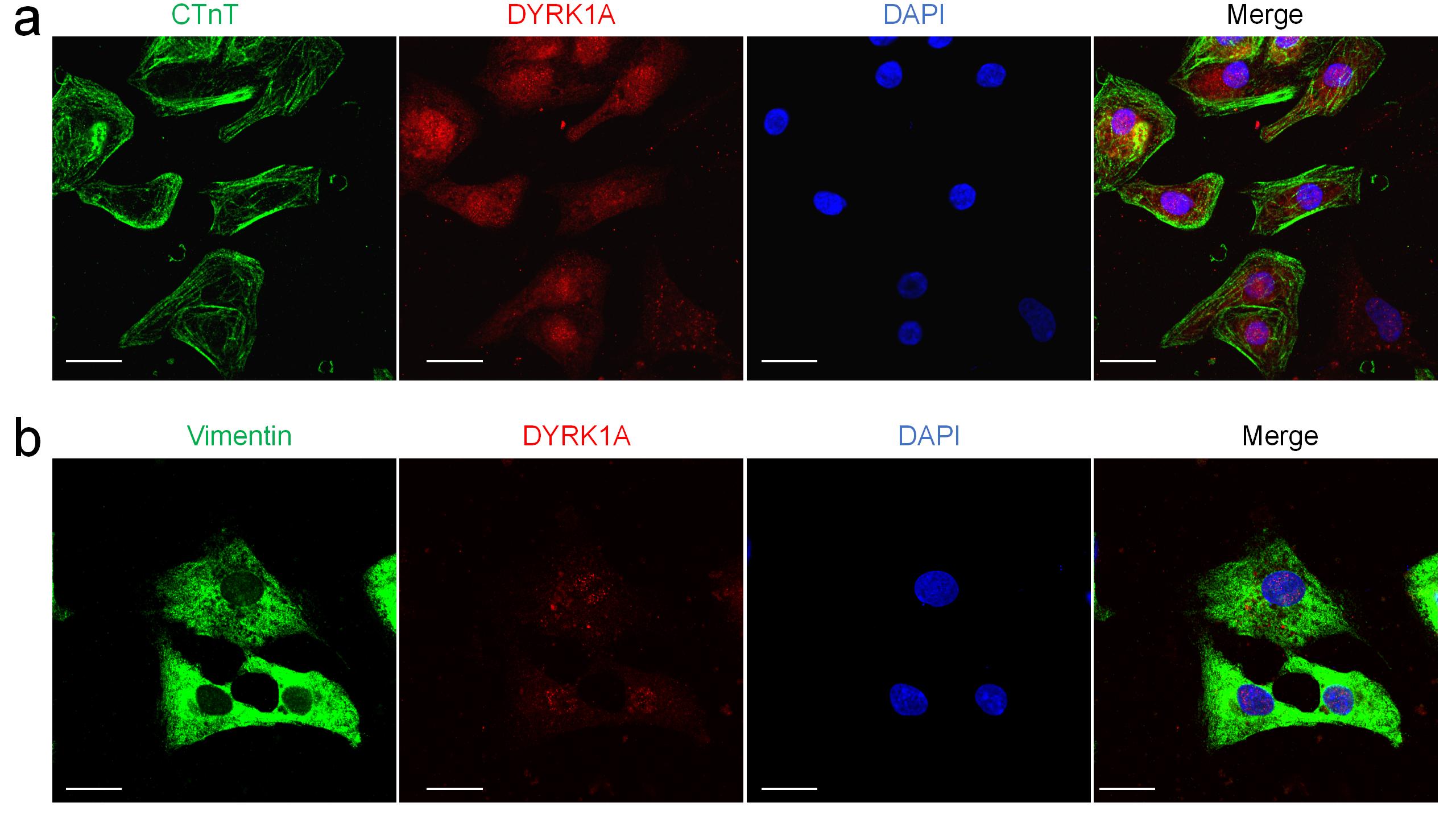


**Supplementary Figure 1. DYRK1A expression in cardiomyocytes and cardiac fibroblasts.** (**a**) DYRK1A expression in cardiomyocytes. Neonatal cardiomyocytes in primary culture were stained for cTnT (green), DYRK1A (red), and DAPI (blue). Scale bar=20 μm. (**b**) DYRK1A expression in cardiac fibroblasts. Cardiac fibroblasts in primary culture were stained for vimentin (green), DYRK1A (red), and DAPI (blue). Scale bar=20 μm.


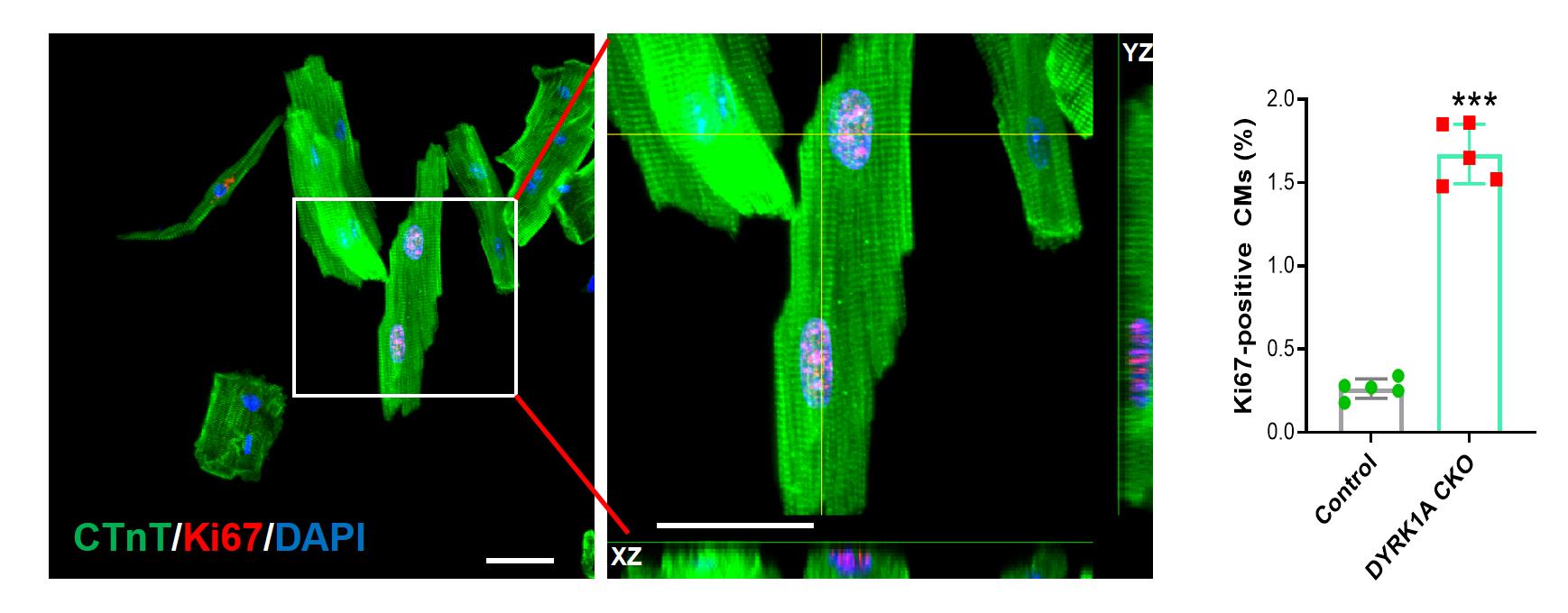


**Supplementary Figure 2. Loss of DYRK1A promotes cardiomyocyte cell cycle activation following MI in adult hearts.** Control (αMHC^MerCreMer^) and α-MHC^MerCreMer^/DYRK1A^flox/flox^ mice (DYRK1A CKO) were subjected to MI. The hearts were harvested and the cardiomyocytes were dissociated on day 35 post-MI, followed by immunostaining with Ki67 to measure cardiomyocyte cell cycle activity. Representative images with z-stacking (left) and quantification of the percentage of Ki67-positive cardiomyocytes (right) are shown. CMs: cardiomyocytes (>10,000 cardiomyocytes from five mice were analyzed in each group). Scale bar=40 µm. All data are expressed as mean ± SD and significant differences were analyzed using the two-tailed unpaired Student’s t test. ^***^P<0.001 versus the control group.


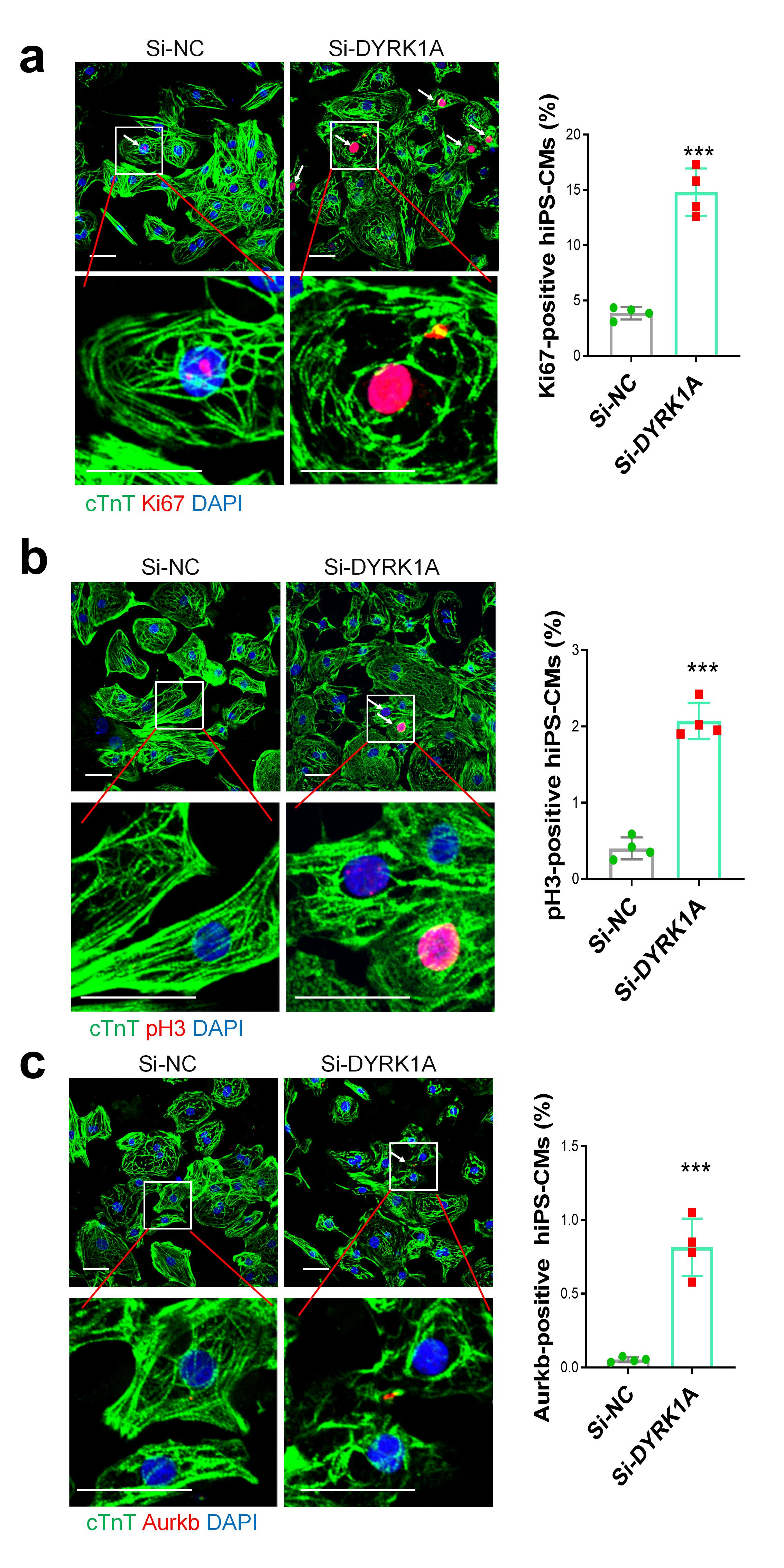


**Supplementary Figure 3. DYRK1A knockdown promotes cell cycle activation of human induced pluripotent stem cell (iPS)-derived cardiomyocytes *in vitro*.** Human iPS-derived cardiomyocytes were transfected with scramble (si-NC) or DYRK1A siRNA (si-DYRK1A), and cell cycle activity was identified by immunoblotting 48 h after transfection. (**a-c**) Immunofluorescence staining (left) and quantification (right) of Ki67- (**a**), pH3- (**b**), and Aurkb- (**c**) positive cardiomyocytes were performed to determine cell cycle activity. Ki67-, pH3-, or Aurkb-positive cardiomyocytes are indicated by white arrows. Scale bar=40 μm. An enlarged view of cardiomyocytes, indicated by white squares, is shown. hiPS-CMs indicates human iPS-derived cardiomyocytes (>10,000 cardiomyocytes from four independent experiments per group were analyzed). All data are expressed as mean ± SD and significant differences were analyzed using the two-tailed unpaired Student’s t test. ^***^P<0.001 versus the si-NC group.


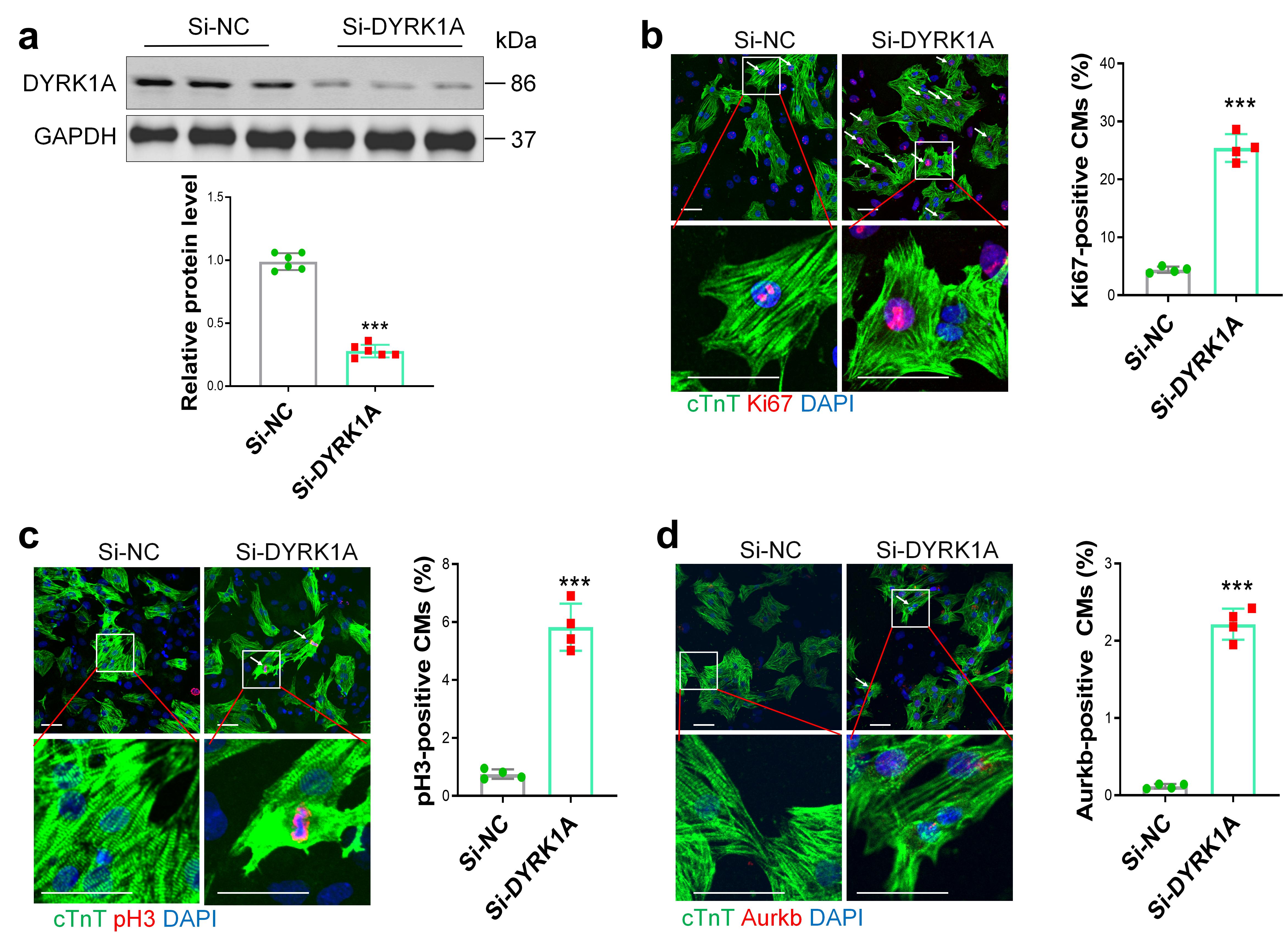


**Supplementary Figure 4. DYRK1A knockdown promotes primary neonatal cardiomyocyte cell cycle activation *in vitro*.** (**a**) DYRK1A knockdown efficiency in primary neonatal cardiomyocytes *in vitro*. Protein expression of DYRK1A in cardiomyocytes, transfected with scramble (si-NC) or DYRK1A siRNA (si-DYRK1A), was detected by immunoblotting 48 h after transfection (n=6 samples per group). Representative immunoblots (upper) and quantification of protein level (lower) are shown. (**b-d**) Effect of DYRK1A knockdown on primary neonatal cardiomyocyte cell cycle activity. Immunofluorescence staining (left) and quantification (right) of Ki67- (**b**), pH3- (**c**), and Aurkb- (**d**) positive cardiomyocytes were performed 48 h after si-NC or si-DYRK1A transfection. Ki67-, pH3-, and Aurkb-positive cardiomyocytes are indicated by white arrows. Scale bar=40 μm. Enlarged views of cardiomyocytes, indicated by white squares, are shown. CMs: cardiomyocytes (>10,000 cardiomyocytes from four independent experiments per group were analyzed). All data are expressed as mean ± SD and significant differences were analyzed using the two-tailed unpaired Student’s t test. ^***^P<0.001 versus the si-NC group.


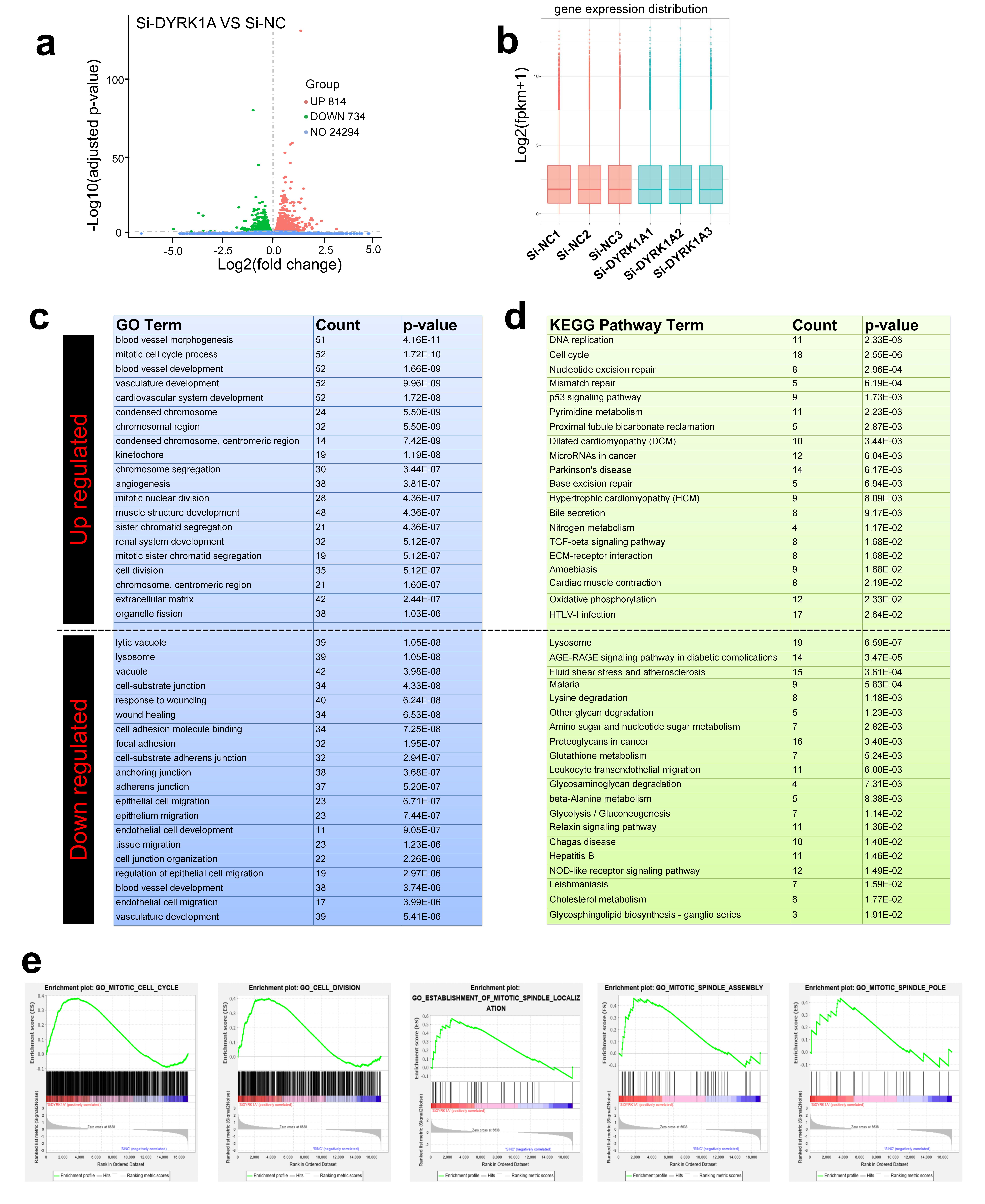


**Supplementary Figure 5. RNA-sequencing analysis reveals that DYRK1A knockdown leads to activation of cardiomyocyte cell cycle activity.** RNA-sequencing (RNA-seq) analysis was performed in primary neonatal cardiomyocytes transfected with scramble (si-NC) or DYRK1A siRNA (si-DYRK1A). (**a**) The volcano plot of RNA-seq shows changes in gene expression induced by DYRK1A knockdown, relative to si-NC. The green dots indicate significantly downregulated genes; red, significantly upregulated genes (adjusted p value < 0.05); and blue, represent genes with no changes in expression. (**b**) Box plots of the RNA-seq read counts (log2(FPKM+1)) show gene expression distribution in replicates of si-DYRK1A- and si-NC-treated cardiomyocytes. (**c**) GO analysis of upregulated and downregulated genes in si-DYRK1A cardiomyocytes (the top 20 most significantly affected terms are shown). (**d**) KEGG pathway analysis of upregulated and downregulated genes in si-DYRK1A cardiomyocytes (the top 20 most significantly affected terms are shown). (**e**) GSEA analysis of selected gene sets (GO terms relevant to cell proliferation) shows a correlation between DYRK1A knockdown and cardiomyocyte cell cycle activation.





**Supplementary Figure 6. Distribution of H3K4me3 and H3K27ac on representative genes (cell cycle genes and regulators).** H3K4me3 and H3K27ac chromatin immunoprecipitation with sequencing (ChIP-seq) was performed in scramble (si-NC)- and DYRK1A siRNA (si-DYRK1A)-transfected cardiomyocytes. Visualization of H3K4me3 or H3K27ac ChIP-seq data track is shown. Cell cycle genes were selected and IGV screenshots were used to show representative peaks.


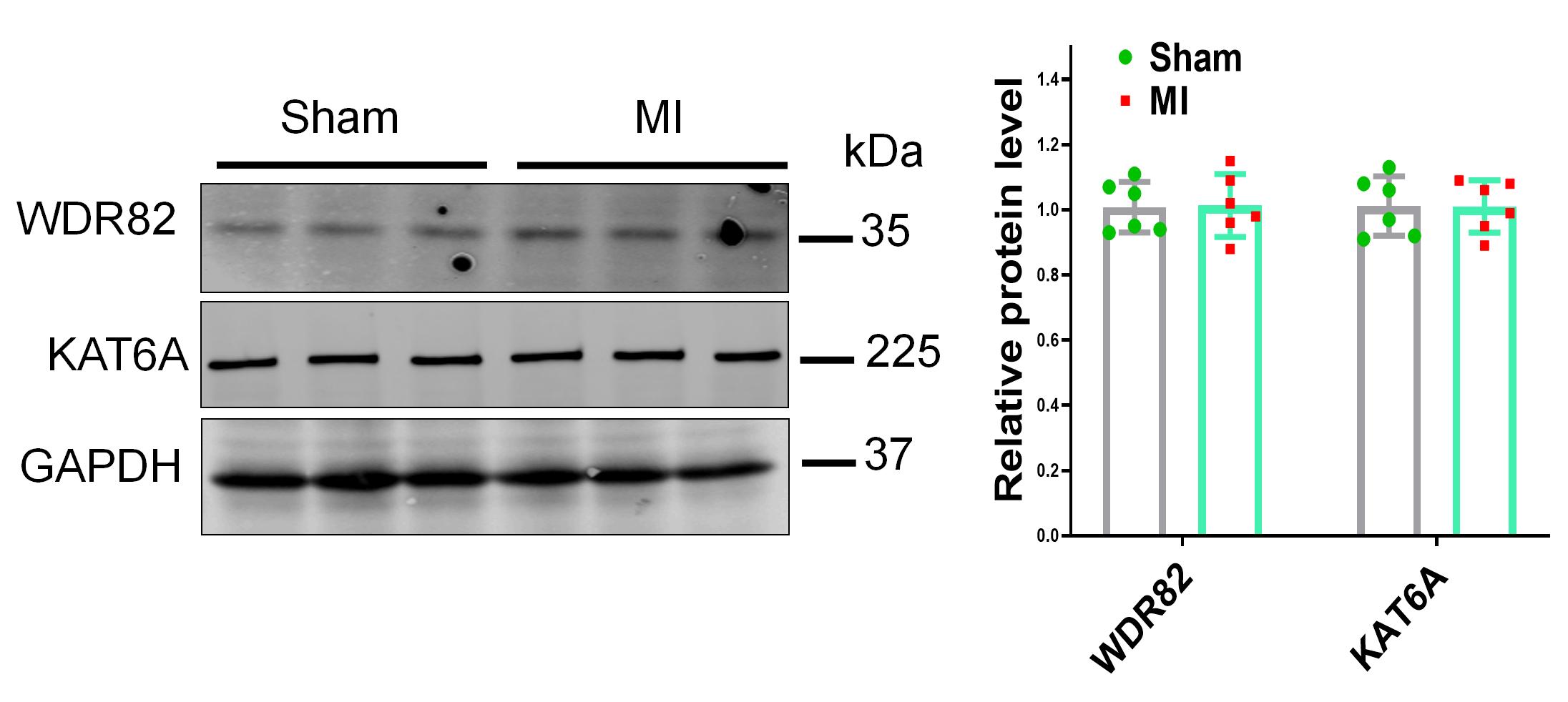


**Supplementary Figure 7. WDR82 and KAT6A expression in hearts with MI.** Adult mice were subjected to MI or sham operation, and protein expressions of WDR82 and KAT6A in the heart were determined at day 35 post-MI (n=6 mice per group). Representative immunoblots (left) and quantifications of protein levels (right) are shown. All data are expressed as mean ± SD and significant differences were analyzed using the two-tailed unpaired Student’s t test.


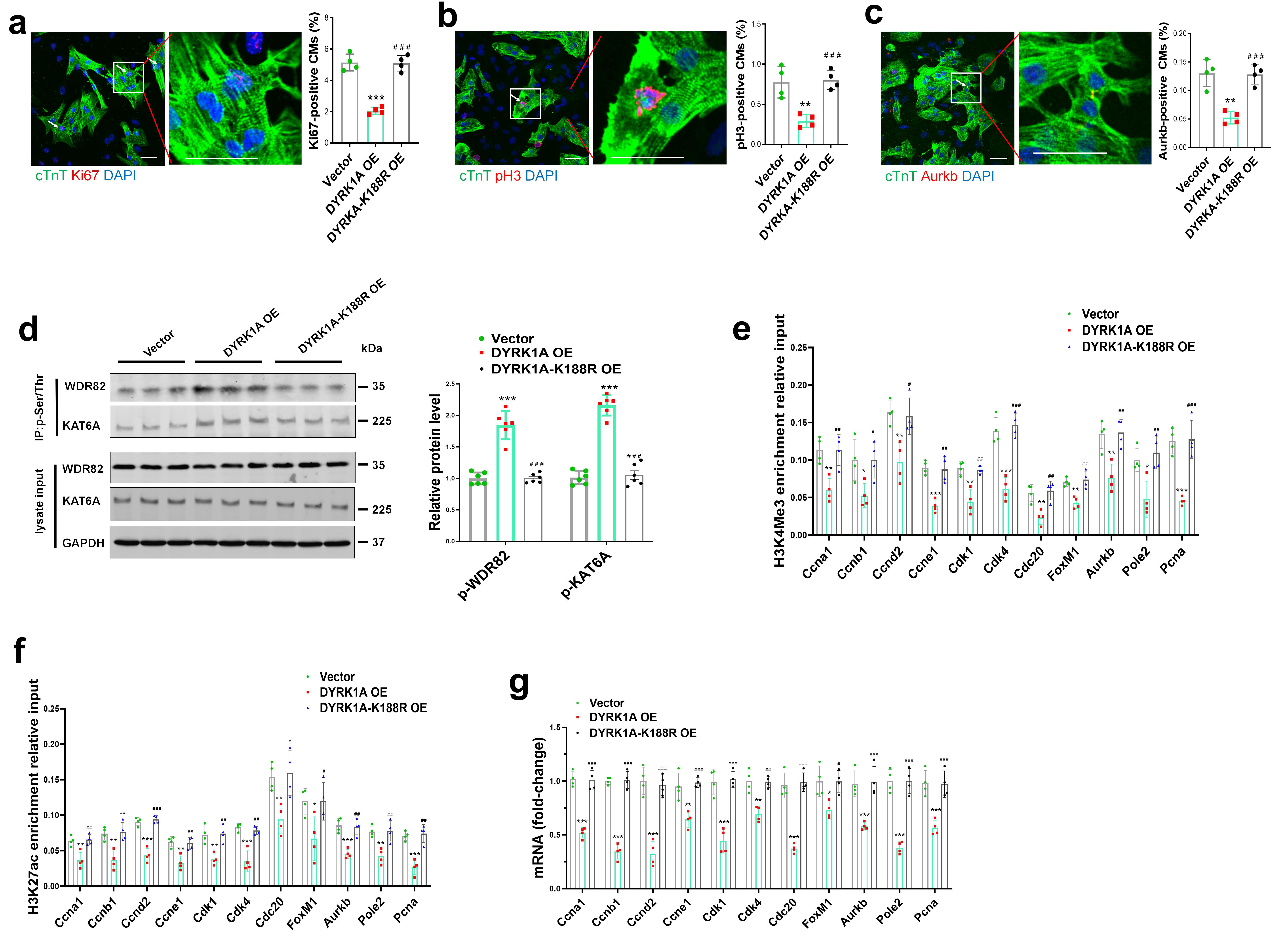


**Supplementary Figure 8. DYRK1A regulation of cardiomyocyte cell cycle activity is dependent on its kinase activity**. Neonatal cardiomyocytes in primary culture were transfected with control vector plasmid (Vector), DYRK1A overexpression (DYRK1A OE) plasmid, or a kinase-dead DYRK1A overexpression (DYRK1A-K188R OE) plasmid for 48 h. (**a-c**) Immunofluorescence staining (left) and quantification (right) of Ki67- (**a**), pH3- (**b**), and Aurkb- (**c**) positive cardiomyocytes were studied to show cardiomyocyte cell cycle activity. Ki67-, pH3-, and Aurkb-positive cardiomyocytes are indicated by arrows. Scale bar=40 μm. An enlarged view of cardiomyocytes, indicated by white squares, is also shown. CMs = cardiomyocytes (>10,000 cardiomyocytes from four independent experiments per group were analyzed). (^**^P<0.01, ^***^P<0.001) versus vector group; ^###^P<0.001 versus DYRK1A OE group. (**d**) The effect of DYRK1A overexpression on the phosphorylation of WDR82 and KAT6A was detected using Co-IP assays. Total phosphorylation (Ser and Thr) antibody was used for immunoprecipitation and WDR82 or KAT6A antibody was used for immunoblotting. (n=6 samples per group). ^***^P<0.001 versus vector group; ^###^P<0.001 versus DYRK1A OE group. (**e–f**) Effect of DYRK1A overexpression on H3K4me3 (**e**) and H3K27ac (**f**) deposition on promoters of cell cycle regulatory genes. ChIP-qPCR assays were performed (n=4 samples per group). (^*^P<0.05, ^**^P<0.01, ^***^P<0.001) versus vector group; (^#^P<0.05, ^##^P<0.01, ^###^P<0.001) versus DYRK1A OE group. (**g**) Effect of DYRK1A overexpression on the expression of cell cycle regulatory genes. qPCR assays were performed (n=4 samples per group). (^*^P<0.05, ^**^P<0.01, ^***^P<0.001) versus vector group; (^#^P<0.05, ^##^P<0.01, ^###^P<0.001) versus DYRK1A OE group. All data are expressed as mean ± SD and analyzed using one-way ANOVA followed by Tukey’s multiple-comparison test.


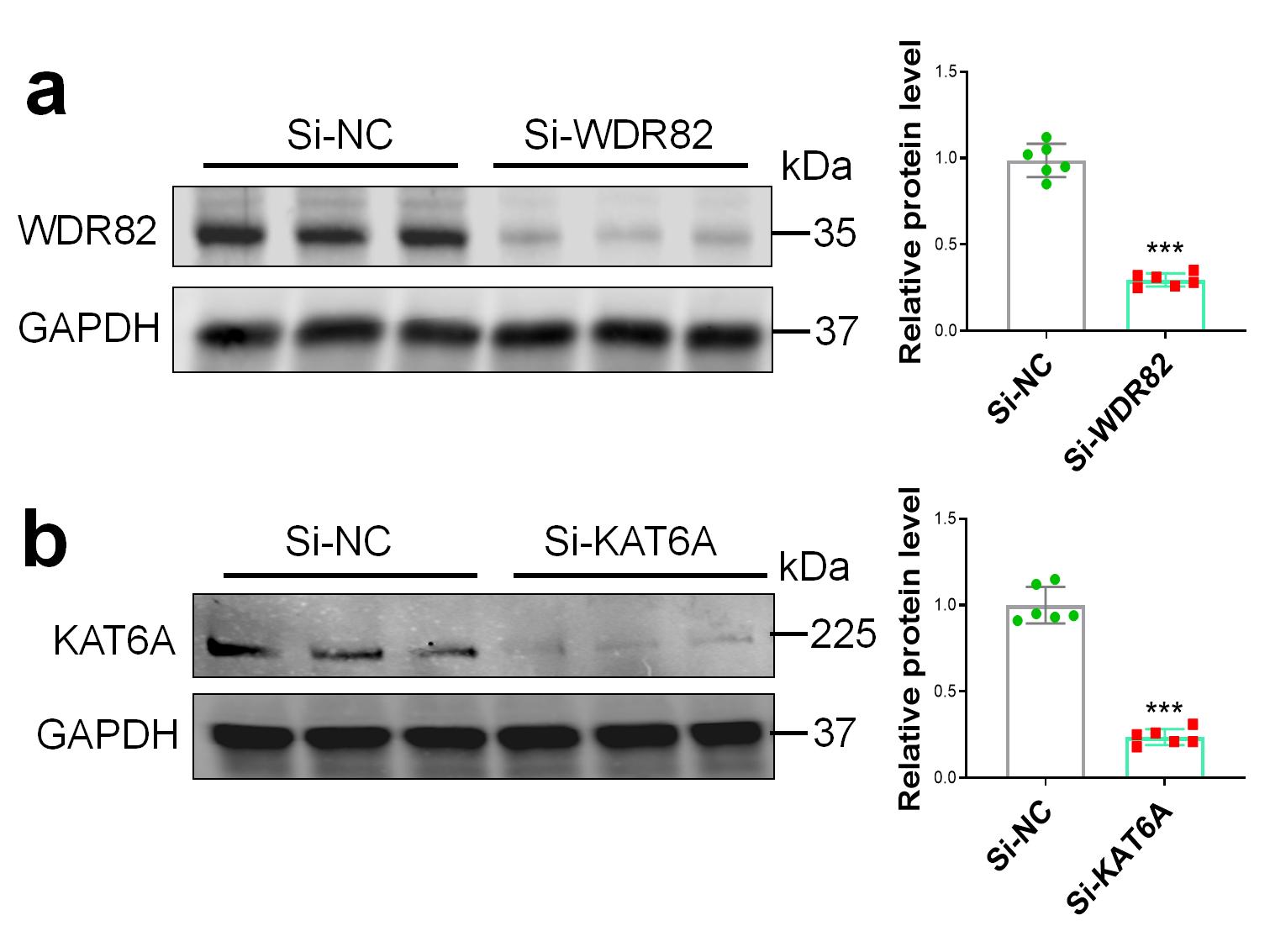


**Supplementary Figure 9. Knockdown of WDR82 and KAT6A with the corresponding siRNA in cardiomyocytes.** Neonatal cardiomyocytes in primary culture were transfected with scramble (si-NC), WDR82 siRNA (si-WDR82), or KAT6A siRNA (si-KAT6A) for 48 h. (**a**) WDR82 expression in cardiomyocytes transfected with si-NC or si-WDR82 was detected by immunoblotting (n=6 samples per group). Representative immunoblots (left) and protein level quantification (right) are shown. (**b**) KAT6A expression in cardiomyocytes transfected with si-NC or si-KAT6A was detected by immunoblotting (n=6 samples per group). Representative immunoblots (left) and protein level quantification (right) are shown. All data are expressed as mean ± SD and significant differences were analyzed using the two-tailed unpaired Student’s t test. ^***^P<0.001 versus si-NC group.


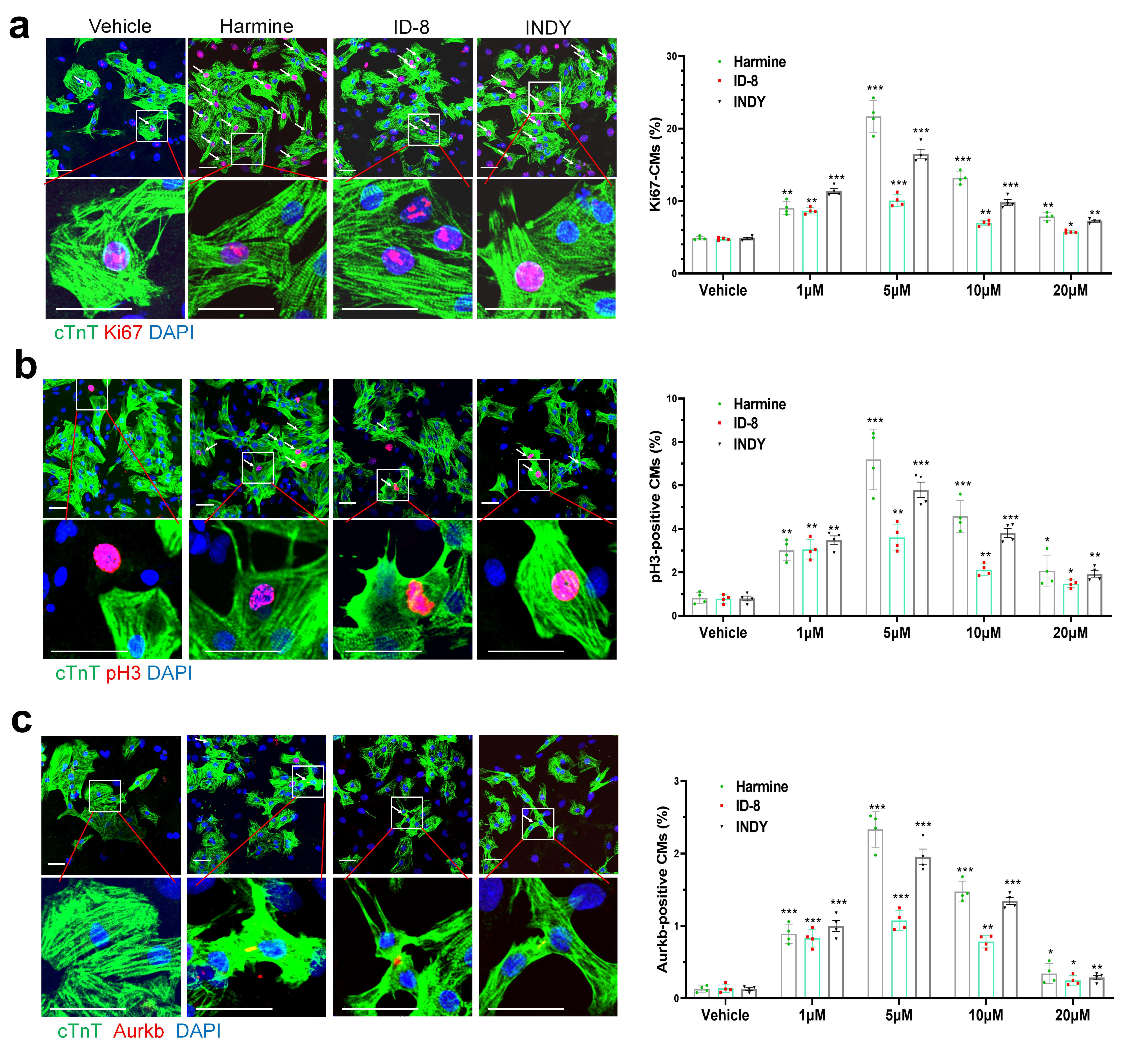


**Supplementary Figure 10. Inhibition of DYRK1A promotes cardiomyocyte cell cycle activation *in vitro*.** (**a–c**) Immunofluorescence staining (left) and quantification (right) of Ki67- (**a**), pH3- (**b**), and Aurkb- (**c**) positive cardiomyocytes were used to show cardiomyocyte cell cycle activity 24 h after treatment with control vehicle (DMSO), DYRK1A inhibitors harmine, ID-8, and INDY at the indicated concentrations. Ki67, pH3, and Aurkb-positive cardiomyocytes are indicated by arrows. Scale bar=40 μm. An enlarged view of cardiomyocytes, indicated by white squares, is also shown. CMs: cardiomyocytes (>10,000 cardiomyocytes from four independent experiments per group were analyzed). All data are expressed as mean ± SD and analyzed using one-way ANOVA followed by Tukey’s multiple-comparison test. (^*^P<0.05, ^**^P<0.01, ^***^P<0.001) versus vehicle group.


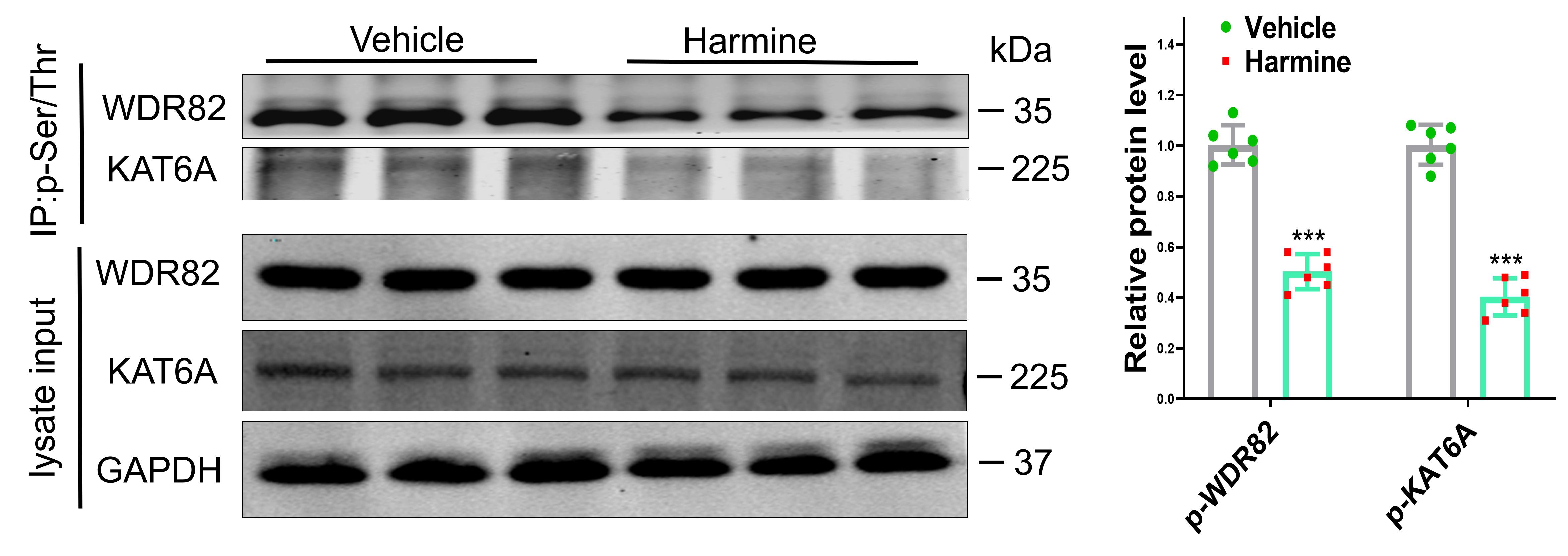


**Supplementary Figure 11. Inhibition of DYRK1A by harmine reduces WDR82 and KAT6A phosphorylation.** Co-IP assays showed the effect of harmine on the phosphorylation of WDR82 and KAT6A. Total phosphorylation (Ser and Thr) antibody was used for immunoprecipitation and WDR82 or KAT6A antibody was used for immunoblotting. Co-IP was performed 24 h after treatment with control vehicle or harmine (5 μM). (n=6 samples per group). Representative immunoblots (left) and quantification of protein levels (right) are shown. All data are expressed as mean ± SD and significant differences were analyzed using the two-tailed unpaired Student’s t test. ^***^P<0.001 versus vehicle group.


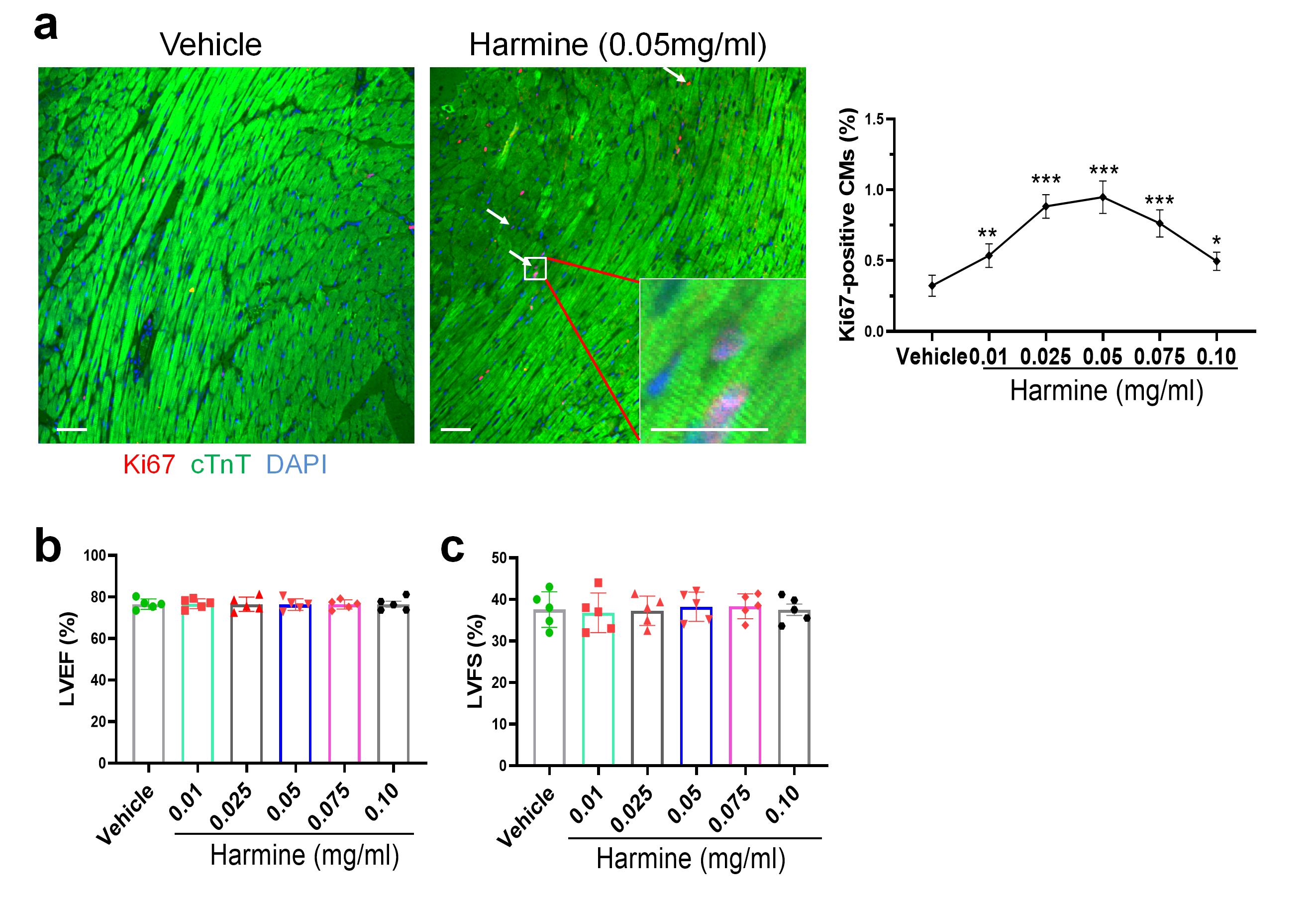


**Supplementary Figure 12. Harmine promotes cell cycle activation of cardiomyocyte in adult mice in basal state.** Harmine was added in drinking water at various concentrations (ranging from 0.01mg/ml to 0.1 mg/ml) and administered to adult mice for one week. (**a**) The effect of harmine on cardiomyocyte cell cycle activity *in vivo* was evaluated by Ki67 immunostaining. Representative images of Ki67 immunofluorescence staining (left) in hearts of control vehicle or harmine (0.05 mg/ml)-treated mice and quantifications (right) of Ki67-positive cardiomyocytes in each group are shown. Scale bar=50 μm. An enlarged view of cardiomyocytes (Scale bar=25 μm), indicated by white squares, is also shown. CMs: cardiomyocytes (>10,000 cardiomyocytes from five mice per group were analyzed). (^*^P<0.05, ^**^P<0.01, ^***^P<0.001) versus vehicle group. (**b-c**) Cardiac function were analyzed by echocardiography. Quantitative analysis of left ventricular ejection fraction (LVEF, **b**) and fraction shortening (LVFS, **c**) are shown. All data are expressed as mean ± SD and analyzed using one-way ANOVA followed by Tukey’s multiple-comparison test.


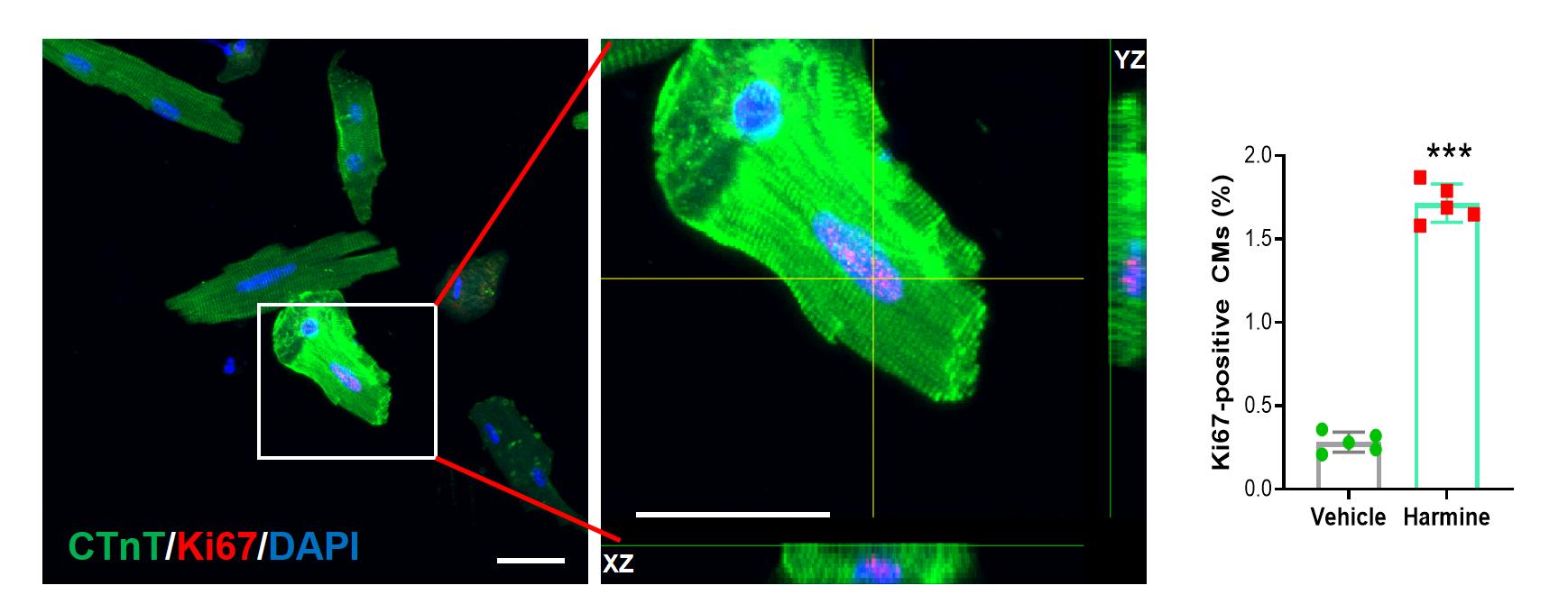


**Supplementary Figure 13. Pharmacological inhibition of DYRK1A promotes cardiomyocyte cell cycle activation following MI in adult hearts.** Mice were subjected to MI and then treated with vehicle or harmine, which was dissolved in drinking water at a concentration of 0.05 mg/ml. Hearts were harvested and dissociated at day 35 post-MI, followed by immunostaining of Ki67 to evaluate cardiomyocyte cell cycle activity. Representative images with z-stacking (left) and quantification of percentage of Ki67-positive cardiomyocytes (right) are shown. CMs: cardiomyocytes (>10,000 cardiomyocytes from five mice were analyzed in each group). Scale bar=40 µm. All data are expressed as mean ± SD and significant differences were analyzed using the two-tailed unpaired Student’s t test. ^***^P<0.001 versus vehicle group.
