# Supplementary material for: Inhibition of DYRK1A, via histone modification, promotes cardiomyocyte cell cycle activation and cardiac repair after myocardial infarction
Source: eBioMedicine. 2022 Jul 8;82:104139. doi: 10.1016/j.ebiom.2022.104139 (PMC9278077; doi:10.1016/j.ebiom.2022.104139)
Supplement: Supplementary file 4 [file mmc4.docx]

**Supplementary Table 2.** DYRK1A-interacting proteins detected by mass spectrometry analysis.

| **Protein ID** | **Protein Name** |
| --- | --- |
| F1M953 | Stress-70 protein, mitochondrial |
| P06761 | Endoplasmic reticulum chaperone BiP |
| P68035 | Actin, alpha cardiac muscle 1 |
| F1LP05 | ATP synthase subunit alpha |
| P10719 | ATP synthase subunit beta, mitochondrial |
| A0A0G2JUF6 | Isocitrate dehydrogenase [NADP] |
| P63259 | Actin, cytoplasmic 2 |
| D4A4S3 | Heat shock cognate 71 kDa protein |
| P11507 | Sarcoplasmic/endoplasmic reticulum calcium ATPase 2 |
| M0R590 | Glyceraldehyde-3-phosphate dehydrogenase |
| A0A0G2JSH5 | Serum albumin |
| F1LMU0 | Myosin-4 |
| G3V885 | Myosin-6 |
| P23928 | Alpha-crystallin B chain |
| P63039 | 60 kDa heat shock protein, mitochondrial |
| P85108 | Tubulin beta-2A chain |
| Q6AYZ1 | Tubulin alpha-1C chain |
| G3V7C6 | Tubulin beta chain |
| P20788 | Cytochrome b-c1 complex subunit Rieske, mitochondrial |
| F1M8P6 | Uncharacterized protein |
| Q6PDU6 | Beta-glo |
| P02091 | Hemoglobin subunit beta-1 |
| P19511 | ATP synthase F(0) complex subunit B1, mitochondrial |
| P31399 | ATP synthase subunit d, mitochondrial |
| A0A0G2JSH9 | Peroxiredoxin-2 |
| F1LWG8 | Sarcalumenin |
| M0R757 | Elongation factor 1-alpha |
| F1M7V4 | Protein piccolo |
| P04642 | L-lactate dehydrogenase A chain |
| P11240 | Cytochrome c oxidase subunit 5A, mitochondrial |
| P11951 | Cytochrome c oxidase subunit 6C-2 |
| P13803 | Electron transfer flavoprotein subunit alpha, mitochondrial |
| P26453 | Basigin |
| P32551 | Cytochrome b-c1 complex subunit 2, mitochondrial |
| P62260 | 14-3-3 protein epsilon |
| P63245 | Receptor of activated protein C kinase 1 |
| Q05962 | ADP/ATP translocase 1 |
| Q68FU3 | Electron transfer flavoprotein subunit beta |
| A0A096MJX4 | Fibrous sheath-interacting protein 2 |
| A0A097PE04 | Cytochrome c oxidase subunit 2 |
| A0A0G2K9Y0 | Uncharacterized protein |
| B2RYX1 | LOC685322 protein |
| Q6PCU0 | ATP synthase subunit gamma |
| D4AD48 | DCN1-like protein |
| D3ZMX6 | Syntrophin, beta 2 |
| A0A096MK47 | Muscular LMNA-interacting protein |
| A2RRU3 | U3 small nucleolar RNA-associated protein 15 homolog |
| D3ZAF6 | ATP synthase subunit f, mitochondrial |
| D4ABP9 | F-box only protein 3 |
| O35509 | Ras-related protein Rab-11B |
| O55165 | Kinesin-like protein KIF3C |
| O70535 | Leukemia inhibitory factor receptor |
| P00762 | Anionic trypsin-1 |
| P00787 | Cathepsin B |
| P01946 | Hemoglobin subunit alpha-1/2 |
| P02767 | Transthyretin |
| P02773 | Alpha-fetoprotein |
| P04764 | Alpha-enolase |
| P04916 | Retinol-binding protein 4 |
| P0C6P5 | Rho guanine nucleotide exchange factor 28 |
| P10888 | Cytochrome c oxidase subunit 4 isoform 1, mitochondrial |
| P11232 | Thioredoxin |
| P11608 | ATP synthase protein 8 |
| P16036 | Phosphate carrier protein, mitochondrial |
| P16409 | Myosin light chain 3 |
| P19234 | NADH dehydrogenase [ubiquinone] flavoprotein 2, mitochondrial |
| P20761 | Ig gamma-2B chain C region |
| P34058 | Heat shock protein HSP 90-beta |
| P35171 | Cytochrome c oxidase subunit 7A2, mitochondrial |
| P41565 | Isocitrate dehydrogenase [NAD] subunit gamma 1, mitochondrial |
| P42930 | Heat shock protein beta-1 |
| P43244 | Matrin-3 |
| P51868 | Calsequestrin-2 |
| P61016 | Cardiac phospholamban |
| P62804 | Histone H4 |
| P62997 | Transformer-2 protein homolog beta |
| P63156 | BarH-like 1 homeobox protein |
| P70585 | Probable G-protein coupled receptor 19 |
| P81556 | Metalloproteinase inhibitor 4 |
| P84079 | ADP-ribosylation factor 1 |
| P85834 | Elongation factor Tu, mitochondrial |
| Q01750 | General transcription factor IIF subunit 2 |
| Q06647 | ATP synthase subunit O, mitochondrial |
| Q3MIF2 | Lethal(3)malignant brain tumor-like protein 2 |
| Q498U4 | SAP domain-containing ribonucleoprotein |
| Q499P8 | RUS1 family protein C16orf58 homolog |
| Q5XI46 | Uncharacterized protein C10orf88 homolog |
| Q63120 | Canalicular multispecific organic anion transporter 1 |
| Q63472 | Potassium voltage-gated channel subfamily H member 1 |
| Q641Y2 | NADH dehydrogenase [ubiquinone] iron-sulfur protein 2, mitochondrial |
| Q641Y6 | Protein phosphatase 1J |
| Q64716 | Insulin receptor-related protein |
| Q66HD5 | CAP-Gly domain-containing linker protein 4 |
| Q68FY0 | Cytochrome b-c1 complex subunit 1, mitochondrial |
| Q6PDU7 | ATP synthase subunit g, mitochondrial |
| Q6U6G5 | Zinc finger CCCH domain-containing protein 15 |
| Q8R2H7 | Trafficking kinesin-binding protein 2 |
| Q8R4C1 | Calcium-transporting ATPase type 2C member 2 |
| Q9EPV5 | Apoptotic protease-activating factor 1 |
| Q9ERA7 | Mesothelin |
| Q9JIM0 | Double-strand break repair protein MRE11 |
| Q9R1Z0 | Voltage-dependent anion-selective channel protein 3 |
| A0A0A0MY12 | Histone acetyltransferase |
| A0A0G2JU82 | Microtubule-actin cross-linking factor 1 |
| A0A0G2JW80 | COP9 signalosome complex subunit 1 |
| A0A0G2JWS3 | Piezo-type mechanosensitive ion channel component |
| A0A0G2JXC2 | Formin-like 3 |
| A0A0G2K1C5 | Uncharacterized protein |
| A0A0G2K3X1 | Troponin I type 2 (Skeletal, fast), isoform CRA_a |
| A0A0G2K7C2 | Kinesin family member 20B |
| A0A0G2K8Z9 | Kinesin family member 13B |
| A0A0G2K929 | Centrosomal protein 290 |
| A0A0G2K972 | Tudor domain-containing 15 |
| A0A0G2K9R9 | Proteasome activator subunit 4 |
| A1EC78 | Transcriptional adapter 2-alpha |
| B0BMX4 | LOC683469 protein |
| B0BNE6 | NADH dehydrogenase (Ubiquinone) Fe-S protein 8 (Predicted), isoform CRA_a |
| B1H223 | Down syndrome critical region gene 3 |
| B1H235 | Banp protein |
| B1WBT1 | Cdc14a protein |
| B2GVA0 | B-box and SPRY domain containing |
| B2RYJ5 | Tmprss13 protein |
| B2RZD6 | NDUFA4, mitochondrial complex-associated |
| B5DFA8 | mRNA-capping enzyme |
| D3ZFQ8 | Cytochrome c-1 |
| D3ZHR4 | Ankyrin repeat domain 35 |
| D3ZIE9 | Delta-1-pyrroline-5-carboxylate synthase |
| D3ZJT9 | L3MBTL3, histone methyl-lysine-binding protein |
| D3ZKC5 | Sterile alpha motif domain-containing 3 |
| D3ZKD4 | Chromatin licensing and DNA replication factor 1 |
| D3ZLA6 | SLAM family member 9 |
| D3ZMB0 | A disintegrin-like and metalloprotease (Reprolysin type) with thrombospondin type 1 motif, 9 (Predicted) |
| D3ZP67 | Protein phosphatase 1, regulatory (Inhibitor) subunit 15b (Predicted) |
| D3ZQR6 | Echinoderm microtubule-associated protein-like 6 |
| D3ZSG2 | Tetratricopeptide repeat and ankyrin repeat-containing 1 |
| D3ZU52 | COP9 (Constitutive photomorphogenic) homolog, subunit 7b (Arabidopsis thaliana) (Predicted), isoform CRA_b |
| D3ZUD5 | OFD1, centriole and centriolar satellite protein |
| D3ZUH5 | Proline-rich 36 |
| D3ZV29 | Uncharacterized protein |
| D3ZWZ1 | Integrin subunit alpha X |
| D3ZY07 | Serine/threonine-protein kinase PLK |
| D3ZY10 | Kelch-like family member 34 |
| D4A0T0 | NADH:ubiquinone oxidoreductase subunit B10 |
| D4A2A5 | Beta-1,4-galactosyltransferase 2 |
| D4A3E1 | Heterogeneous nuclear ribonucleoprotein L-like |
| D4A554 | Eukaryotic translation initiation factor 4 gamma, 3 |
| D4A582 | Zinc finger, DBF-type-containing 2 |
| D4A7Z1 | RIMS-binding protein 3 |
| D4ABX7 | Similar to dystonin isoform 1 |
| D4ACL6 | Ras association (RalGDS/AF-6) domain family 3 (Predicted) |
| D4ACN3 | HECT and RLD domain-containing E3 ubiquitin protein ligase 2 |
| D4AD01 | RCG49513 |
| D4AEL1 | PNMA family member 5 |
| F1LMD9 | Cyclin-G-associated kinase |
| F1LMG7 | Leucine-rich repeat-containing protein 7 |
| F1LMV6 | Desmoplakin |
| F1LPB9 | Rabphilin-3A |
| F1LR42 | RUN and FYVE domain-containing 1 |
| F1LXF5 | Golgi to ER traffic protein 4 |
| F1LZC5 | Uncharacterized protein |
| F1M021 | Beta-1,4-N-acetylgalactosaminyltransferase |
| F1M9H4 | Human immunodeficiency virus type I enhancer-binding protein 1 |
| F7FKI5 | Pyruvate dehydrogenase E1 component subunit alpha |
| G3V7Y3 | ATP synthase subunit delta, mitochondrial |
| G3V811 | Coagulation factor XIII A chain |
| G3V8V1 | Granulin, isoform CRA_c |
| G3V925 | Pregnancy-specific beta 1-glycoprotein |
| M0R927 | Uncharacterized protein |
| M0R991 | Natural killer cell triggering receptor |
| M0RCP3 | Uncharacterized protein |
| M0RDA9 | Olfactory receptor 214 |
| P97552 | Laminin gamma1 |
| Q0D2L2 | Mitochondrial ribosomal protein S22 |
| Q2KML4 | Dynein axonemal heavy chain-like protein |
| Q3KRF2 | High density lipoprotein binding protein (Vigilin) |
| Q4QQS6 | ALG5, dolichyl-phosphate beta-glucosyltransferase |
| Q5BJV6 | Modulator of apoptosis 1 |
| Q5BJX7 | RGD1307929 protein |
| Q5BK42 | Threonine synthase-like 1 (S. cerevisiae) |
| Q5PPN0 | Hairy and enhancer of split 6 (Drosophila) |
| Q5PQZ9 | NADH dehydrogenase [ubiquinone] 1 subunit C2 |
| Q6AY07 | Fructose-bisphosphate aldolase |
| Q6IE05 | BEM46-like 3 |
| Q6TUD6 | LRRGT00108 |
| Q71DI1 | Dermcidin |
| Q7TP06 | Da1-6 |
| Q9Z212 | ULF-250 protein |
| M0RCB1 | Uncharacterized protein |
| G3V6D3 | ATP synthase subunit beta |
| P69897 | Tubulin beta-5 chain |
| Q09073 | ADP/ATP translocase 2 |
| G3V8B0 | Myosin-7 |
| Q6P9Y4 | ADP/ATP translocase 1 |
| P68370 | Tubulin alpha-1A chain |
| Q66HF1 | NADH-ubiquinone oxidoreductase 75 kDa subunit, mitochondrial |
| A0A0G2K793 | Heat shock protein HSP 90-beta |
| D3ZG43 | NADH dehydrogenase (Ubiquinone) Fe-S protein 3 (Predicted), isoform CRA_c |
| A0A0G2K9H8 | FK506 binding protein 11 |
| F1M6C2 | Uncharacterized protein |
| Q37652 | Cytochrome c oxidase subunit 2 |
| Q6IRH6 | Phosphate carrier protein, mitochondrial |
| Q6QI09 | LRRGT00199 |
| P06685 | Sodium/potassium-transporting ATPase subunit alpha-1 |
| P11980 | Pyruvate kinase PKM |
| Q29RW1 | Myosin-4 |
| Q99NA5 | Isocitrate dehydrogenase [NAD] subunit alpha, mitochondrial |
| Q9Z2L0 | Voltage-dependent anion-selective channel protein 1 |
| P08733 | Myosin regulatory light chain 2, ventricular/cardiac muscle isoform |
| P18163 | Long-chain-fatty-acid--CoA ligase 1 |
| P21571 | ATP synthase-coupling factor 6, mitochondrial |
| P28480 | T-complex protein 1 subunit alpha |
| P38983 | 40S ribosomal protein SA |
| A0A0G2JZR4 | Ras-related protein Rab-11B |
| Q0QF18 | Succinate dehydrogenase (quinone) |
| Q5BK05 | LOC367586 protein |
| Q5RK08 | Glioblastoma amplified sequence |
| Q63607 | Alpha-tropomyosin 3 |
| Q6GT74 | Basigin, isoform CRA_b |
| A0A0U1RRP2 | DCN1-like protein |
| P47709 | Rabphilin-3A |
| A1A5S1 | Pre-mRNA-processing factor 6 |
| B2GV05 | RNA-binding protein 5 |
| O55005 | Roundabout homolog 1 |
| O88807 | Protein-arginine deiminase type-4 |
| P02401 | 60S acidic ribosomal protein P2 |
| P12007 | Isovaleryl-CoA dehydrogenase, mitochondrial |
| P24898 | Transcription factor jun-B |
| P26819 | Beta-adrenergic receptor kinase 2 |
| P35286 | Ras-related protein Rab-13 |
| P41739 | Aryl hydrocarbon receptor nuclear translocator |
| P49432 | Pyruvate dehydrogenase E1 component subunit beta, mitochondrial |
| P51871 | Cytochrome P450 4F6 |
| P56819 | Beta-secretase 1 |
| P62716 | Serine/threonine-protein phosphatase 2A catalytic subunit beta isoform |
| P62836 | Ras-related protein Rap-1A |
| P67779 | Prohibitin |
| P68511 | 14-3-3 protein eta |
| P80432 | Cytochrome c oxidase subunit 7C, mitochondrial |
| P97874 | Cyclin-G-associated kinase |
| Q00715 | Histone H2B type 1 |
| Q01728 | Sodium/calcium exchanger 1 |
| Q07936 | Annexin A2 |
| Q505J3 | WSC domain-containing protein 1 |
| Q5BJU7 | Wiskott-Aldrich syndrome protein family member 1 |
| Q5U2X0 | CDKN2A-interacting protein |
| Q5U2Y9 | Lebercilin |
| Q5XIH7 | Prohibitin-2 |
| Q5XIS1 | Protein phosphatase Slingshot homolog 3 |
| Q63083 | Nucleobindin-1 |
| Q641Y0 | Dolichyl-diphosphooligosaccharide--protein glycosyltransferase 48 kDa subunit |
| Q64428 | Trifunctional enzyme subunit alpha, mitochondrial |
| Q64617 | Protein kinase C eta type |
| Q68FR2 | Bridging integrator 2 |
| Q6AYK4 | Apoptosis facilitator Bcl-2-like protein 14 |
| Q791F6 | Transmembrane protein adipocyte-associated 1 |
| Q80W89 | NADH dehydrogenase [ubiquinone] 1 alpha subcomplex subunit 11 |
| Q8R508 | Protocadherin Fat 3 |
| A0A096MJY1 | Glypican 6 |
| A0A0A0MXU2 | Coronin |
| A0A0G2JSI4 | Adhesion G protein-coupled receptor E5 |
| A0A0G2JT06 | COP9 signalosome complex subunit 1 |
| A0A0G2JT21 | Janus kinase and microtubule-interacting protein 2 |
| A0A0G2JTM5 | C-C motif chemokine |
| A0A0G2JV26 | Protein phosphatase 4, regulatory subunit 4 |
| A0A0G2JWA8 | Microtubule-actin cross-linking factor 1 |
| A0A0G2JWG6 | Golgin B1 |
| A0A0G2JXD6 | Hydin, axonemal central pair apparatus protein |
| A0A0G2JY19 | Hepatocyte growth factor receptor |
| A0A0G2JZV7 | Uncharacterized protein |
| A0A0G2K0A0 | Cytohesin 4 |
| A0A0G2K330 | Trifunctional enzyme subunit beta, mitochondrial |
| A0A0G2K4W9 | Uncharacterized protein |
| A0A0G2K9M4 | WD repeat and FYVE domain-containing 3 |
| A0A0G2KA50 | VPS39 HOPS complex subunit |
| A0A0H2UI40 | Breast cancer metastasis-suppressor 1 homolog |
| A0A140UHY0 | LCA5L, lebercilin-like |
| A0A1W2Q6F8 | NADH dehydrogenase [ubiquinone] 1 alpha subcomplex subunit 10, mitochondrial |
| B0BN16 | Insulin-like growth factor binding protein-like 1 (Predicted), isoform CRA_a |
| B2B9B0 | Eph receptor B2 |
| D3Z9Z0 | Ankyrin 1 |
| D3ZAM4 | CCM2-like scaffold protein |
| D3ZD73 | DEAD-box helicase 6 |
| D3ZFF8 | Uncharacterized protein |
| D3ZI33 | Fukutin |
| D3ZLC3 | Sperm specific antigen 2 |
| D3ZM33 | Uncharacterized protein |
| D3ZNJ5 | Indolethylamine N-methyltransferase |
| D3ZPL2 | Uncharacterized protein |
| D3ZS58 | NADH dehydrogenase [ubiquinone] 1 alpha subcomplex subunit 2 |
| D4A166 | RAS p21 protein activator 4 |
| D4A3V2 | NADH dehydrogenase [ubiquinone] 1 alpha subcomplex subunit 6 |
| D4A457 | Highly divergent homeobox |
| D4A5N7 | Uncharacterized protein |
| D4A914 | 5'-3' exoribonuclease |
| D4AA61 | Lipase |
| D4AB33 | Paternally-expressed 3 |
| D4ACF3 | Family with sequence similarity 212, member B |
| D6NSP0 | Cytochrome b |
| E9PSJ8 | Similar to RNA polymerase II transcription factor SIII subunit A2 (Elongin A2) (EloA2) (Transcription elongation factor B polypeptide 3B) |
| E9PT22 | Inverted formin, FH2 and WH2 domain containing |
| E9PU42 | DSCR3 arrestin fold containing |
| F1LN75 | Septin-9 |
| F1LPQ8 | SECIS binding protein 2, isoform CRA_a |
| F1LPU4 | Choline O-acetyltransferase |
| F1LSE6 | Liprin-alpha-3 |
| F1LSX7 | Exosome component 5 |
| F1LTJ5 | Uncharacterized protein |
| F1LU18 | SLX4 structure-specific endonuclease subunit |
| F1LZS9 | Cation channel, sperm-associated 3 |
| F1M104 | Uncharacterized protein |
| F1M779 | Clathrin heavy chain |
| F1M9Z9 | ADAM metallopeptidase domain 12 |
| G3V734 | 2,4-dienoyl CoA reductase 1, mitochondrial, isoform CRA_a |
| G3V8B1 | Glycosylphosphatidylinositol specific phospholipase D1, isoform CRA_a |
| M0R4P6 | Uncharacterized protein |
| M0R565 | WD repeat domain 82 |
| M0RDI4 | Zinc finger protein 647 |
| O88752 | Epsilon 1 globin |
| Q3ZB99 | Tight junction protein 2 |
| Q4V7C3 | Uncharacterized protein |
| Q6QI20 | LRRGT00188 |
| Q707P1 | Synaptotagmin 1 |
| Q76KC5 | Phosphodiesterase |
| Q7TP01 | Bq135360 |
| Q80XY0 | Gap junction protein |
| Q9ERE5 | PERIOD 3 |
| Q9QZF6 | Osteoactivin |
| R4GNK3 | Thioredoxin |
