# Supplementary material for: Inhibition of DYRK1A, via histone modification, promotes cardiomyocyte cell cycle activation and cardiac repair after myocardial infarction
Source: eBioMedicine. 2022 Jul 8;82:104139. doi: 10.1016/j.ebiom.2022.104139 (PMC9278077; doi:10.1016/j.ebiom.2022.104139)
Supplement: Supplementary file 5 [file mmc5.pdf]

# DYRK1A polyclonal antibody

**Catalog # :** PAB19417 **規格 :** [ 100 ug ]

List All

## Specification

| | |
|---|---|
| **Product Description:** | Rabbit polyclonal antibody raised against synthetic peptide of DYRK1A. |
| **Immunogen:** | A synthetic peptide corresponding to 17 amino acids near C-terminus of human DYRK1A. |
| **Host:** | Rabbit |
| **Reactivity:** | Human, Mouse, Rat |
| **Specificity:** | Multiple isoforms of DYRK1A are known to exist. DYRK1A antibody will not cross-react with other DYRK family members. |
| **Form:** | Liquid |
| **Purification:** | Peptide affinity purification |
| **Concentration:** | 1 mg/mL |
| **Recommend Usage:** | Western Blot (1-2 ug/mL)<br>Immunocytochemistry (10 ug/mL)<br>The optimal working dilution should be determined by the end user. |
| **Storage Buffer:** | In PBS (0.02% sodium azide) |
| **Storage Instruction:** | Store at 4°C for three months. For long term storage store at -20°C. Aliquot to avoid repeated freezing and thawing. |
| **Note:** | This product contains sodium azide: a POISONOUS AND HAZARDOUS SUBSTANCE which should be handled by trained staff only. |
| **Datasheet:** | Download |

## Publication Reference


1. Impact of Dyrk1A level on alcohol metabolism.
Renon M, Legrand B, Blanc E, Daubigney F, Bokobza C, Mortreux M, Paul J, Delabar J, Rouach H, Andreau K, Janel N.Biochimica et Biophysica Acta - Molecular Basis of Disease. 2016 May 20. [Epub ahead of print]


## Applications

**Western Blot (Cell lysate)**

## Application Image

Western Blot (Cell lysate)

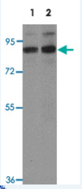


enlarge

Immunocytochemistry

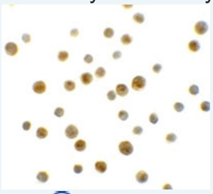

enlarge

Enzyme-linked Immunoabsorbent Assay

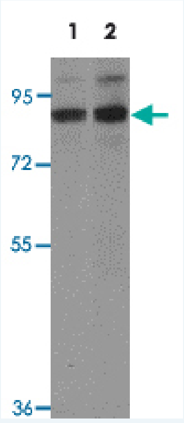


Western blot analysis of DYRK1A in HeLa cell lysate with DYRK1A polyclonal antibody (Cat # PAB19417) at 1 ug/mL (lane 1) and 2 ug/mL (lane 2).

**Immunocytochemistry**

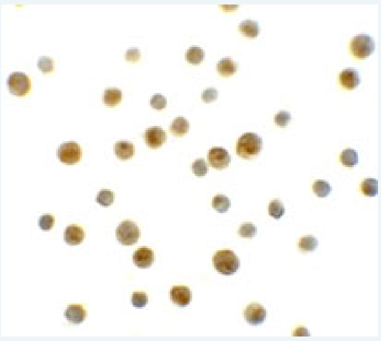

Immunocytochemical staining of HeLa cells with DYRK1A polyclonal antibody (Cat # PAB19417) at 10 ug/mL.

**Enzyme-linked Immunoabsorbent Assay**

## Gene Information

| | |
|---|---|
| **Entrez GeneID:** | 1859 |
| **Protein Accession#:** | NP_001387 |
| **Gene Name:** | DYRK1A |
| **Gene Alias:** | DYRK,DYRK1,HP86,MNB,MNBH |
| **Gene Description:** | dual-specificity tyrosine-(Y)-phosphorylation regulated kinase 1A |
| **Omim ID:** | 600855 |
| **Gene Ontology:** | Hyperlink |
| **Gene Summary:** | This gene encodes a member of the Dual-specificity tyrosine phosphorylation-regulated kinase (DYRK) family. This member contains a nuclear targeting signal sequence, a protein kinase domain, a leucine zipper motif, and a highly conservative 13-consecutive-histidine repeat. It catalyzes its autophosphorylation on serine/threonine and tyrosine residues. It may play a significant role in a signaling pathway regulating cell proliferation and may be involved in brain development. This gene is a homolog of Drosophila mnb (minibrain) gene and rat Dyrk gene. It is localized in the Down syndrome critical region of chromosome 21, and is considered to be a strong candidate gene for learning defects associated with Down syndrome. Alternative splicing of this gene generates several transcript variants differing from each other either in the 5' UTR or in the 3' coding region. These variants encode at least five different isoforms. [provided by RefSeq |

**Other Designations:** MNB/DYRK protein kinase,OTTHUMP00000109090,dual specificity YAK1-related kinase,minibrain homolog,mnb protein kinase homolog hp86,protein kinase minibrain homolog,serine/threonine kinase MNB,serine/threonine-specific protein kinase

## Related Disease

Alzheimer Disease Genetic Predisposition to Disease

服務條款 | 隱私權政策 | 著作及商標 | 網站地圖

©2022 亞諾法生技股份有限公司 Abnova Corporation. 版權所有.

# Cardiac Troponin T Monoclonal Antibody (13-11)

| Product Details | |
|---|---|
| Size | 200 µL |
| Species Reactivity | Avian, Dog, Chicken, Fish, Guinea pig, Human, Mouse, Pig, Rabbit, Rat |
| Published Species | Dog, Rat, Hamster, Zebrafish, Mouse, Human, Xenopus |
| Host/Isotype | Mouse / IgG1 |
| Class | Monoclonal |
| Type | Antibody |
| Clone | 13-11 |
| Conjugate | Unconjugated |
| Immunogen | Purified rabbit cardiac troponin T isoform (TnT4R) |
| Form | Liquid |
| Concentration | 0.5 mg/mL |
| Purification | Affinity chromatography |
| Storage buffer | PBS, pH 7.2, with 0.2% BSA |
| Contains | 0.09% sodium azide |
| Storage conditions | 4° C |
| RRID | AB_11000742 |

| Applications | Tested Dilution | Publications |
|---|---|---|
| Western Blot (WB) | 1 µg/mL | 10 Publications |
| Immunohistochemistry (IHC) | - | 54 Publications |
| Immunohistochemistry (Paraffin) (IHC (P)) | 1-2 µg/mL | 4 Publications |
| Immunohistochemistry (PFA fixed) (IHC (PFA)) | - | 3 Publications |
| Immunohistochemistry (Frozen) (IHC (F)) | 5 µg/mL | 4 Publications |
| Immunocytochemistry (ICC/IF) | 5 µg/mL | 85 Publications |
| Flow Cytometry (Flow) | - | 42 Publications |
| Miscellaneous PubMed (Misc) | - | 1 Publication |

### Product Specific Information

MA5-12960 targets Troponin T Cardiac Isoform in IF/ICC, IHC (P), and IM applications and shows reactivity with Avian, Canine, Chicken, Fish, Guinea Pig, Human, mouse, Porcine, Rabbit, and Rat samples.

The MA5-12960 immunogen is purified rabbit cardiac troponin T isoform (TnT4R).

### Product Images For Cardiac Troponin T Monoclonal Antibody (13-11)

**Cardiac Troponin T Antibody (MA5-12960) in ICC/IF**
Immunofluorescent analysis of Troponin T Cardiac Isoform (green) in cultured primary cardiomyocytes. Primary cardiomyocytes were isolated and cultured using the Primary Cardiomyocyte Isolation Kit (Product # 88281). Cells were grown in 24-well plates (seeded at 5x10^5 cells/well) or in 35mm glass bottom plates (at a density of 2.5 x 106 cells/well). At day 1 and day 7, cells were fixed with 4% paraformaldehyde, permeablilized with 0.1% Triton X-100 in HBSS for 10 minutes at room temperature, and blocked with 3% BSA in PBS (Product # 37525) for 30 minutes at room temperature. Cells were probed with a Troponin T Cardiac Isoform monoclonal antibody (Product # MA5-12960) at dilution of 1:300 for 2 hours at room temperature or overnight at 4 °C, washed with HBSS, and incubated with a DyLight 488-conjugated goat anti-mouse IgG secondary antibody (Product # 35502) at dilution of 1:500 for 1 hour at room temperature. Nuclei (blue) were visualized using Hoechst 33342 (Product # 62249) and dead cells were visualized using Propidium iodide (red). Images were taken at 20X (Panels A and B) or 60X (Panel C) magnifications on a Carl Zeiss microscope (AxioVision Rel. 4.7).

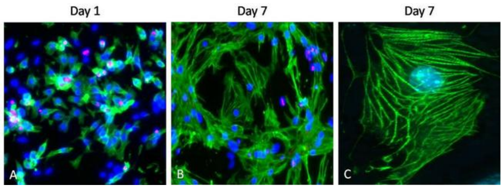


**Cardiac Troponin T Antibody (MA5-12960)**
Antibody specificity was demonstrated by detection of differential basal expression of the target across tissue models owing to their inherent genetic constitution. Expression of Cardiac Troponin T was observed specifically in heart tissue and was negative for skeletal muscle and lung tissue using Anti-Cardiac Troponin T Monoclonal Antibody (Product # MA5-12960) in western blot. {RE}

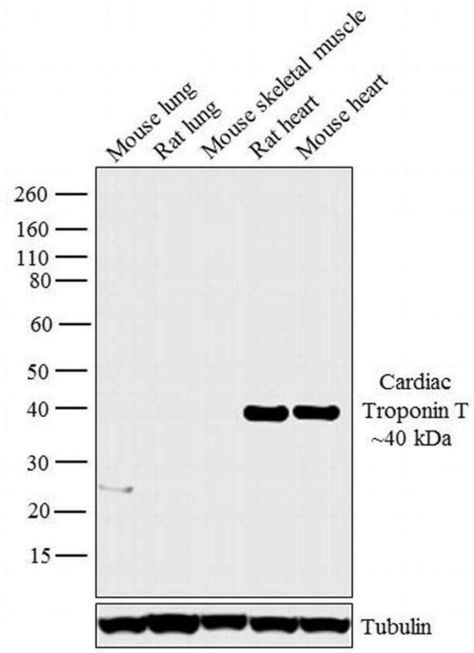


**Cardiac Troponin T Antibody (MA5-12960) in IHC (F)**
Immunofluorescence analysis of Cardiac Troponin T in mouse heart tissue: Frozen sections were fixed with 4% paraformaldehyde for 20 minutes, permeabilized with 0.1% Triton™ X-100 for 15 minutes, and blocked with 10% Normal goat serum for 1 hour at room temperature. Whole heart longitudinal sections were then incubated with Cardiac Troponin T Mouse Monoclonal Antibody (Product# MA5-12960, 5µg /mL) overnight at 4°C, followed by Goat anti-Mouse IgG (H+L) Superclonal™ Secondary Antibody, Alexa Fluor® 488 conjugate (Product # A28175, 1:2000,45 mins). Nuclei (blue) were stained using SlowFade® Gold Antifade Mountant with DAPI (Product # S36938), and cytoskeletal F-actin (red) was stained using Rhodamine Phalloidin (Product # R415, 1:300). Panel a) represents staining with the matched isotype control. Panel b) shows representative sections stained for Cardiac Troponin T (green). The images were captured at 20X magnification.

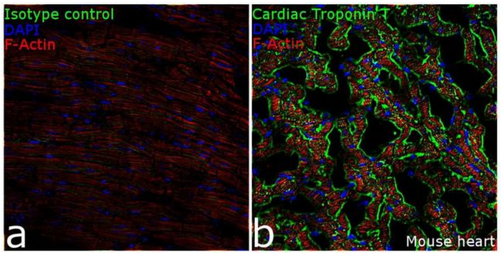

## View more figures on thermofisher.cn

## 203 References

### Western Blot (10)

**International journal of molecular sciences**

**Bioactive Lipid O-cyclic phytosphingosine-1-phosphate Promotes Differentiation of Human Embryonic Stem Cells into Cardiomyocytes via ALK3/BMPR Signaling.**

"Published figure using Cardiac Troponin T monoclonal antibody (Product # MA5-12960) in Western Blot"

Authors: Jang JH,Kim MS,Antao AM,Jo WJ,Kim HJ,Kim SJ,Choi MJ,Ramakrishna S,Kim KS

**Species**
Human
Not Applicable

**Dilution**
Not Cited
Not Cited

**Year**
2021

**Scientific reports**

**AAV-mediated YAP expression in cardiac fibroblasts promotes inflammation and increases fibrosis.**

"Published figure using Cardiac Troponin T monoclonal antibody (Product # MA5-12960) in Immunohistochemistry (Paraffin)"

Authors: Francisco J,Zhang Y,Nakada Y,Jeong JI,Huang CY,Ivessa A,Oka S,Babu GJ,Del Re DP

**Species**
Mouse
Not Applicable

**Dilution**
1:2000
Not Cited

**Year**
2021

View more WB references on thermofisher.cn

### Immunohistochemistry (54)

**Stem cells international**

**miRNAs in Extracellular Vesicles from iPS-Derived Cardiac Progenitor Cells Effectively Reduce Fibrosis and Promote Angiogenesis in Infarcted Heart.**

"MA5-12960 was used in Immunohistochemistry to report that extracellular vesicles (EV) secreted by ISX-9-induced CPCs (EV-CPCISX-9) faithfully recapitulate the beneficial effects of their parent CPCs with regard to postinfarction remodeling."

Authors: Xuan W,Wang L,Xu M,Weintraub NL,Ashraf M

**Species**
Mouse

**Dilution**
1:300

**Year**
2022

**Stem cell reports**

**Pharmacologic therapy for engraftment arrhythmia induced by transplantation of human cardiomyocytes.**

"Published figure using Cardiac Troponin T monoclonal antibody (Product # MA5-12960) in Immunohistochemistry"

Authors: Nakamura K,Neidig LE,Yang X,Weber GJ,El-Nachef D,Tsuchida H,Dupras S,Kalucki FA,Jayabalu A,Futakuchi-Tsuchida A,Nakamura DS,Marchianò S,Bertero A,Robinson MR,Cain K,Whittington D,Tian R,Reinecke H,Pabon L, Knollmann BC,Kattman S,Thies RS,MacLellan WR,Murry CE

**Species**
Not Applicable

**Dilution**
Not Cited

**Year**
2021

View more IHC references on thermofisher.cn

### More applications with references on thermofisher.cn

IHC (P) (4) | IHC (PFA) (3) | IHC (F) (4) | ICC/IF (85) | Flow (42) | Misc (1)

For Research Use Only. Not for use in diagnostic procedures. Not for resale without express authorization. Products are warranted to operate or perform substantially in conformance with published Product specifications in effect at the time of sale, as set forth in the Production documentation, specifications and/or accompanying package inserts ("Documentation"). No claim of suitability for use in applications regulated by FDA is made. The warranty provided herein is valid only when used by properly trained individuals. Unless otherwise stated in the Documentation, this warranty is limited to one year from date of shipment when the Product is subjected to normal, proper and intended usage. This warranty does not extend to anyone other than the Buyer. Any model or sample furnished to Buyer is merely illustrative of the general type and quality of goods and does not represent that any Product will conform to such model or sample. NO OTHER WARRANTIES, EXPRESS OR IMPLED, ARE GRANTED INCLUDING WITHOUT LIMITATION, IMPLIED WARRANTIES OF MERCHANTABILITY, FITNESS FOR ANY PARTICULAR PURPOSE, OR NON INFRINGEMENT. BUYER'S EXCLUSIVE REMEDY FOR NON-CONFORMING PRODUCTS DURING THE WARRANTY PERIOD IS LIMITED TO REPAIR, REPLACEMENT OF OR REFUND FOR THE NON-CONFORMING PRODUCT(S) AT SELLER'S SOLE OPTION. THERE IS NO OBLIGATION TO REPAIR, REPLACE OR REFUND FOR PRODUCTS AS THE RESULT OF (I) ACCIDENT, DISASTER OR EVENT OF FORCE MAJEURE, (II) MISUSE, FAULT OR NEGLIGENCE OF OR BY BUYER, (III) USE OF THE PRODUCTS IN A MANNER FOR WHICH THEY WERE NOT DESIGNED, OR (IV) IMPROPER STORAGE AND HANDLING OF THE PRODUCTS. Unless otherwise expressly stated on the Product or in the documentation accompanying the Product, the Product is intended for research only and is not to be used for any other purpose, including without limitation, unauthorized commercial uses, in vitro diagnostic uses, ex vivo or in vivo therapeutic uses, or any type of consumption by or application to human or animals.

# Ki-67 Polyclonal Antibody

| Product Details | |
|---|---|
| Size | 100 µg |
| Species Reactivity | Bovine, Dog, Horse, Hamster, Human, Mouse, Non-human primate, Sheep, Pig, Rabbit, Rat |
| Published Species | Rat, Sheep, Mouse, Human |
| Host/Isotype | Rabbit / IgG |
| Class | Polyclonal |
| Type | Antibody |
| Conjugate | Unconjugated |
| Immunogen | Synthetic peptide conjugated to KLH derived from within residues 1200 - 1300 of Human Ki67. |
| Form | Liquid |
| Concentration | 1 mg/mL |
| Purification | Antigen affinity chromatography |
| Storage buffer | PBS, pH 7.4 |
| Contains | 0.02% sodium azide |
| Storage conditions | Store at 4°C short term. For long term storage, store at -20°C, avoiding freeze/thaw cycles. |
| RRID | AB_10981523 |

| Applications | Tested Dilution | Publications |
|---|---|---|
| Immunohistochemistry (IHC) | - | 36 Publications |
| Immunohistochemistry (Paraffin) (IHC (P)) | 0.1 - 5 µg/mL | 4 Publications |
| Immunohistochemistry (Frozen) (IHC (F)) | - | 3 Publications |
| Immunocytochemistry (ICC/IF) | 0.5-1 µg/ml | 6 Publications |
| Miscellaneous PubMed (Misc) | - | 4 Publications |

**Product Specific Information**

Heat-mediated antigen retrieval is recommended prior to tissue staining.

Recommended positive controls:

ICC/IF - Wildtype HAP1 cells, SK-N-SH cells, HeLa cells, MEF1 cells

IHC (P) - Mouse and human spleen tissue, human skin carcinoma tissue, human colon tissue, mouse tumor tissue, human skin tissue

## Product Images For Ki-67 Polyclonal Antibody

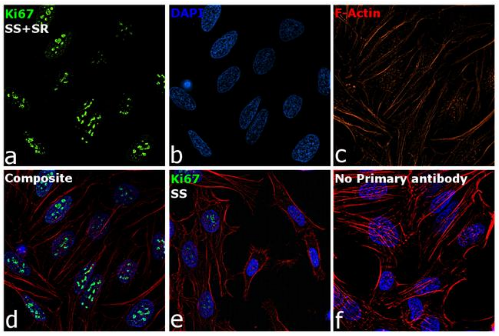


**Ki-67 Antibody (PA5-19462) in ICC/IF**

Immunofluorescence analysis of Ki67 was performed using 70% confluent log phase HeLa cells serum starved for 36 Hrs followed by serum release for 6 Hrs. The cells were fixed with 4% Paraformaldehyde for 10 minutes, permeabilized with 0.1% Triton™ X-100 for 10 minutes, and blocked with 2% BSA for 10 minutes at room temperature. The cells were labeled with Ki-67 Polyclonal Antibody (Product # PA5-19462) at 1 µg/mL in 0.1% BSA, incubated at 4 degree celsius overnight and then labeled with Goat anti-Rabbit IgG (H+L) Superclonal™ Secondary Antibody, Alexa Fluor® 488 conjugate (Product # A27034, 1:2000 dilution) for 45 minutes at room temperature (Panel a: Green). Nuclei (Panel b: Blue) were stained with SlowFade® Gold Antifade Mountant with DAPI (Product # S36938). F-actin (Panel c: Red) was stained with Rhodamine Phalloidin (Product # R415, 1:300). Panel d represents the merged image showing nuclear localization. Panel e represents serum starved cells with reduced signal. Panel f represents control cells with no primary antibody to assess background. The images were captured at 60X magnification.

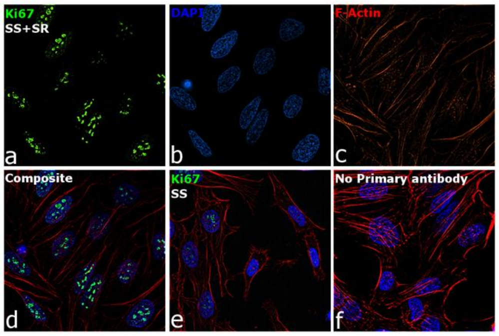


**Ki-67 Antibody (PA5-19462)**

Altered expression of target protein upon cell treatment demonstrates antibody specificity. Immunofluorescence analysis using Anti-Ki-67 Polyclonal Antibody (Product # PA5-19462) shows increased expression of proteins upon serum starvation followed by serum release in HeLa cells. {TM}

---

### View more figures on thermofisher.cn

## 53 References

### Immunohistochemistry (36)

**Frontiers in neuroscience**

**Differential Proliferation and Maturation of Subcortical Astrocytes During Postnatal Development.**

"Published figure using Ki-67 polyclonal antibody (Product # PA5-19462) in Immunocytochemistry"

Authors: Shoneye T,Orrego AT,Jarvis R,Men Y,Chiang MSR,Yang Y

**Species**
Mouse
Not Applicable

**Dilution**
1:100
Not Cited

**Year**
2022

**Cell transplantation**

**LncRNA MNX1-AS1 Contributes to Laryngeal Squamous Cell Carcinoma Growth and Migration by Regulating mir-744-5p/bcl9/-Catenin Axis.**

"Published figure using Ki-67 polyclonal antibody (Product # PA5-19462) in Immunohistochemistry"

Authors: Ma B,Ren G,Xu J,Yin C,Shi Y

**Species**
Not Applicable

**Dilution**
Not Cited

**Year**
2021

View more IHC references on thermofisher.cn

### Immunohistochemistry (Paraffin) (4)

**Scientific reports**

**Conditional ablation of TGF- signaling inhibits tumor progression and invasion in an induced mouse bladder cancer model.**

"Published figure using Ki-67 polyclonal antibody (Product # PA5-19462) in Immunohistochemistry"

Authors: Liang Y,Zhu F,Zhang H,Chen D,Zhang X,Gao Q,Li Y

**Species**
Mouse

**Dilution**
Not Cited

**Year**
2016

**Oncotarget**

**High fat diet increases melanoma cell growth in the bone marrow by inducing osteopontin and interleukin 6.**

"Published figure using Ki-67 polyclonal antibody (Product # PA5-19462) in Immunofluorescence"

Authors: Chen GL,Luo Y,Eriksson D,Meng X,Qian C,Bäuerle T,Chen XX,Schett G,Bozec A

**Species**
Mouse

**Dilution**
1:50

**Year**
2016

View more IHC (P) references on thermofisher.cn

### More applications with references on thermofisher.cn

**IHC (F) (3)** **ICC/IF (6)** **Misc (4)**

For Research Use Only. Not for use in diagnostic procedures. Not for resale without express authorization. Products are warranted to operate or perform substantially in conformance with published Product specifications in effect at the time of sale, as set forth in the Production documentation, specifications and/or accompanying package inserts ("Documentation"). No claim of suitability for use in applications regulated by FDA is made. The warranty provided herein is valid only when used by properly trained individuals. Unless otherwise stated in the Documentation, this warranty is limited to one year from date of shipment when the Product is subjected to normal, proper and intended usage. This warranty does not extend to anyone other than the Buyer. Any model or sample furnished to Buyer is merely illustrative of the general type and quality of goods and does not represent that any Product will conform to such model or sample. NO OTHER WARRANTIES, EXPRESS OR IMPLED, ARE GRANTED INCLUDING WITHOUT LIMITATION, IMPLIED WARRANTIES OF MERCHANTABILITY, FITNESS FOR ANY PARTICULAR PURPOSE, OR NON INFRINGEMENT. BUYER'S EXCLUSIVE REMEDY FOR NON-CONFORMING PRODUCTS DURING THE WARRANTY PERIOD IS LIMITED TO REPAIR, REPLACEMENT OF OR REFUND FOR THE NON-CONFORMING PRODUCT(S) AT SELLER'S SOLE OPTION. THERE IS NO OBLIGATION TO REPAIR, REPLACE OR REFUND FOR PRODUCTS AS THE RESULT OF (I) ACCIDENT, DISASTER OR EVENT OF FORCE MAJEURE, (II) MISUSE, FAULT OR NEGLIGENCE OF OR BY BUYER, (III) USE OF THE PRODUCTS IN A MANNER FOR WHICH THEY WERE NOT DESIGNED, OR (IV) IMPROPER STORAGE AND HANDLING OF THE PRODUCTS. Unless otherwise expressly stated on the Product or in the documentation accompanying the Product, the Product is intended for research only and is not to be used for any other purpose, including without limitation, unauthorized commercial uses, in vitro diagnostic uses, ex vivo or in vivo therapeutic uses, or any type of consumption by or application to human or animals.

# Phospho-Histone H3 (Ser10) Polyclonal Antibody

| **Product Details** | |
|---|---|
| Size | 100 µL |
| Species Reactivity | Fruit fly, Human, Mouse, Non-human primate, Rat, Xenopus |
| Published Species | Rat, Fruit fly, Human, Mouse |
| Host/Isotype | Rabbit / IgG |
| Class | Polyclonal |
| Type | Antibody |
| Conjugate | Unconjugated |
| Immunogen | Synthetic phosphopeptide corresponding to residues surrounding pSer10 of human histone H3 |
| Form | Liquid |
| Purification | Antigen affinity chromatography |
| Storage buffer | 0.01M HEPES, pH 7.5, with 100µg/mL BSA, 0.15M NaCl, 50% glycerol |
| Contains | no preservative |
| Storage conditions | -20°C |
| RRID | AB_10984484 |

| **Applications** | **Tested Dilution** | **Publications** |
|---|---|---|
| Western Blot (WB) | 1:1,000 | 7 Publications |
| Immunohistochemistry (IHC) | - | 6 Publications |
| Immunohistochemistry (Paraffin) (IHC (P)) | 1:200 | - |
| Immunohistochemistry (PFA fixed) (IHC (PFA)) | - | 1 Publication |
| Immunohistochemistry (Frozen) (IHC (F)) | 1:400 | - |
| Immunocytochemistry (ICC/IF) | 1:800 | 4 Publications |
| Flow Cytometry (Flow) | 1:50 | - |
| Peptide Array (Array) | 1:2,000 | - |

### Product Specific Information

Phospho-Histone H3 (Ser10) Antibody detects endogenous levels of histone H3 only when phosphorylated at Ser10; however, this antibody does not detect phosphorylated Ser10 when Lys9 is acetylated or methylated. This antibody does not cross-react with histone H3 phosphorylated at Ser28.

This antibody is not cross-reactive with other phosphorylated histones or with acetylated histones.

# Product Images For Phospho-Histone H3 (Ser10) Polyclonal Antibody

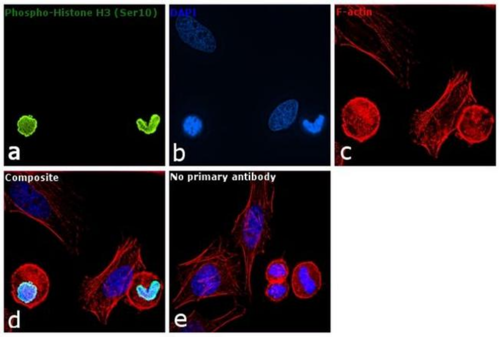


**Phospho-Histone H3 (Ser10) Antibody (PA5-17869)**
Detection of differential subcellular localization of the target protein demonstrates antibody specificity. Immunofluorescence analysis of Phospho-Histone H3(Ser10) using anti-Phospho-Histone H3(Ser10) Polyclonal antibody (Product # PA5-17869 ) shows localization of Phospho-Histone H3 (Ser 10) predominantly in nucleus of dividing HeLa cells. {RE}

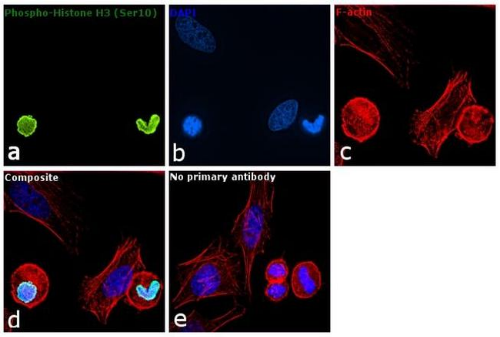


**Phospho-Histone H3 (Ser10) Antibody (PA5-17869) in ICC/IF**
Immunofluorescence analysis of Phospho-Histone H3 (ser 10) was performed using 70% confluent log phase HeLa cells. The cells were fixed with 4% paraformaldehyde for 10 minutes, permeabilized with 0.1% Triton™ X-100 for 15 minutes, and blocked with 1% BSA for 1 hour at room temperature. The cells were labeled with Phospho-Histone H3 (Ser10) Rabbit Polyclonal antibody (Product # PA5-17869) at 1:100 dilution in 0.1% BSA, incubated at 4 degree Celsius overnight and then labeled with Goat anti-Rabbit IgG (H+L) Superclonal™ Secondary Antibody, Alexa Fluor® 488 conjugate (Product # A27034) at a dilution of 1:2000 for 45 minutes at room temperature (Panel a: green). Nuclei (Panel b: blue) were stained with SlowFade® Gold Antifade Mountant with DAPI (Product # S36938). F-actin (Panel c: red) was stained with Rhodamine Phalloidin (Product # R415, 1:300). Panel d represents the merged image showing nuclear localization in dividing cells. Panel e represents control cells with no primary antibody to assess background. The images were captured at 60X magnification.

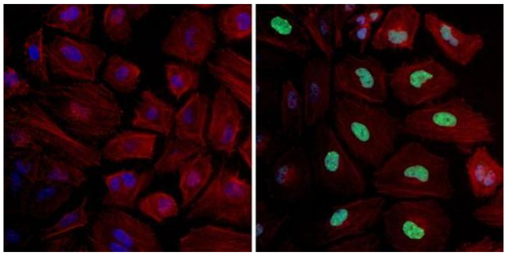

**Phospho-Histone H3 (Ser10) Antibody (PA5-17869) in ICC/IF**
Immunofluorescent analysis of Phospho-Histone H3 pSer10 (green) in HeLa cells. Formalin fixed cells were permeabilized with 0.1% Triton X-100 in TBS for 10 minutes at room temperature and blocked with 1% Blocker BSA (Product # 37525) for 15 minutes at room temperature. Cells were probed without (left panel) or with (right panel) a Phospho-Histone H3 polyclonal antibody (Product # PA5-17869) at a dilution of 1:100 for at least 1 hour at room temperature, washed with PBS, and incubated with DyLight 488 goat anti-rabbit IgG secondary antibody (Product # 35552) at a dilution of 1:400 for 30 minutes at room temperature. F-Actin (red) was stained with DyLight 554 Phalloidin (Product # 21834) and nuclei (blue) were stained with Hoechst 33342 dye (Product # 62249). Images were taken on a Thermo Scientific ArrayScan or a ToxInsight Instrument at 20X magnification.

## View more figures on thermofisher.cn

## 18 References

### Western Blot (7)

**PLoS genetics**

#### Functional assessment of the "two-hit" model for neurodevelopmental defects in Drosophila and X. laevis.

"PA5-17869 was used in Western Blot to suggest that several 16p12.1 genes sensitize the genome towards neurodevelopmental defects, and complex interactions with "second-hit" genes determine the ultimate phenotypic manifestation."

Authors: Pizzo L,Lasser M,Yusuff T,Jensen M,Ingraham P,Huber E,Singh MD,Monahan C,Iyer J,Desai I,Karthikeyan S, Gould DJ,Yennawar S,Weiner AT,Pounraja VK,Krishnan A,Rolls MM,Lowery LA,Girirajan S

**Species**
Fruit fly
Not Applicable

**Dilution**
1:500
Not Cited

**Year**
2021

**Scientific reports**

#### Loss of FOXF1 expression promotes human lung-resident mesenchymal stromal cell migration via ATX/LPA/LPA1 signaling axis.

"PA5-17869 was used in Western Blotting to identify FOXF1 as a novel transcriptional repressor of ATX and demonstrate that loss of FOXF1 promotes LR-MSC migration via the ATX/LPA/LPA1 signalling axis."

Authors: Cao P,Walker NM,Braeuer RR,Mazzoni-Putman S,Aoki Y,Misumi K,Wheeler DS,Vittal R,Lama VN

**Species**
Human
Not Applicable

**Dilution**
1:500
Not Cited

**Year**
2020

View more WB references on thermofisher.cn

### Immunohistochemistry (6)

**Frontiers in cell and developmental biology**

#### MicroRNA Signatures of the Developing Primate Fovea.

"Published figure using Phospho-Histone H3 (Ser10) polyclonal antibody (Product # PA5-17869) in Immunohistochemistry"

Authors: Fishman ES,Louie M,Miltner AM,Cheema SK,Wong J,Schlaeger NM,Moshiri A,Simó S,Tarantal AF,La Torre A

**Species**
Not Applicable

**Dilution**
Not Cited

**Year**
2021

**bioRxiv : the preprint server for biology**

#### Human Taste Cells Express ACE2: a Portal for SARS-CoV-2 Infection.

"Published figure using Phospho-Histone H3 (Ser10) polyclonal antibody (Product # PA5-17869) in Immunohistochemistry"

Authors: Doyle ME,Appleton A,Liu QR,Yao Q,Mazucanti CH,Egan JM

**Species**
Not Applicable

**Dilution**
Not Cited

**Year**
2021

View more IHC references on thermofisher.cn

### More applications with references on thermofisher.cn

IHC (PFA) (1) ICC/IF (4)

For Research Use Only. Not for use in diagnostic procedures. Not for resale without express authorization. Products are warranted to operate or perform substantially in conformance with published Product specifications in effect at the time of sale, as set forth in the Production documentation, specifications and/or accompanying package inserts ("Documentation"). No claim of suitability for use in applications regulated by FDA is made. The warranty provided herein is valid only when used by properly trained individuals. Unless otherwise stated in the Documentation, this warranty is limited to one year from date of shipment when the Product is subjected to normal, proper and intended usage. This warranty does not extend to anyone other than the Buyer. Any model or sample furnished to Buyer is merely illustrative of the general type and quality of goods and does not represent that any Product will conform to such model or sample. NO OTHER WARRANTIES, EXPRESS OR IMPLED, ARE GRANTED INCLUDING WITHOUT LIMITATION, IMPLIED WARRANTIES OF MERCHANTABILITY, FITNESS FOR ANY PARTICULAR PURPOSE, OR NON INFRINGEMENT. BUYER'S EXCLUSIVE REMEDY FOR NON-CONFORMING PRODUCTS DURING THE WARRANTY PERIOD IS LIMITED TO REPAIR, REPLACEMENT OF OR REFUND FOR THE NON-CONFORMING PRODUCT(S) AT SELLER'S SOLE OPTION. THERE IS NO OBLIGATION TO REPAIR, REPLACE OR REFUND FOR PRODUCTS AS THE RESULT OF (I) ACCIDENT, DISASTER OR EVENT OF FORCE MAJEURE, (II) MISUSE, FAULT OR NEGLIGENCE OF OR BY BUYER, (III) USE OF THE PRODUCTS IN A MANNER FOR WHICH THEY WERE NOT DESIGNED, OR (IV) IMPROPER STORAGE AND HANDLING OF THE PRODUCTS. Unless otherwise expressly stated on the Product or in the documentation accompanying the Product, the Product is intended for research only and is not to be used for any other purpose, including without limitation, unauthorized commercial uses, in vitro diagnostic uses, ex vivo or in vivo therapeutic uses, or any type of consumption by or application to human or animals.

# WDR82 Polyclonal Antibody

| **Product Details** | |
|---|---|
| Size | 100 µL |
| Species Reactivity | Human, Mouse, Rat |
| Host/Isotype | Rabbit / IgG |
| Class | Polyclonal |
| Type | Antibody |
| Conjugate | Unconjugated |
| Immunogen | Recombinant fusion protein containing a sequence corresponding to amino acids 174-313 of human WDR82 (NP_079498.2). |
| Form | Liquid |
| Concentration | 3.51 mg/mL |
| Purification | Antigen affinity chromatography |
| Storage buffer | PBS, pH 7.3, with 50% glycerol |
| Contains | 0.02% sodium azide |
| Storage conditions | -20° C, Avoid Freeze/Thaw Cycles |
| RRID | AB_2855991 |

| **Applications** | **Tested Dilution** | **Publications** |
|---|---|---|
| Western Blot (WB) | 1:200-1:2,000 | - |

### Product Specific Information

Sequence of this protein is as follows: YDLRSFDKGP FATFKMQYDR TCEWTGLKFS NDGKLILIST NGSFIRLIDA FKGVVMHTFG GYANSKAVTL EASFTPDSQF IMIGSEDGKI HVWNGESGIK VAVLDGKHTG PITCLQFNPK FMTFASACSN MAFWLPTIDD

## Product Images For WDR82 Polyclonal Antibody

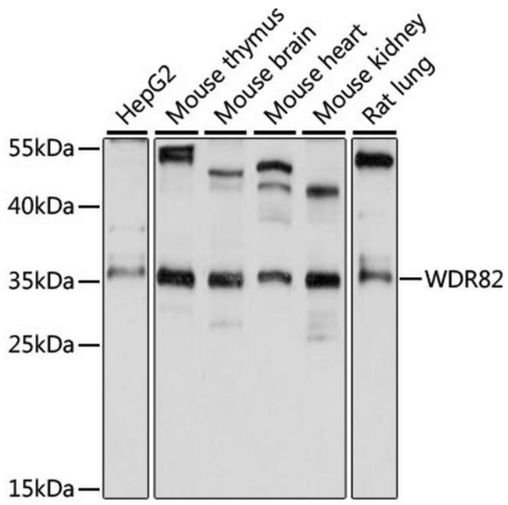


**WDR82 Antibody (PA5-110580) in WB**
Western blot analysis of WDR82 in HepG2, mouse thymus, mouse brain, mouse heart, mouse kidney, rat lung. Samples were incubated in polyclonal WDR82 antibody (Product # PA5-110580) using a dilution of 1:1000, followed by HRP Goat Anti-Rabbit IgG (H+L) at a dilution of 1:10000.

For Research Use Only. Not for use in diagnostic procedures. Not for resale without express authorization. Products are warranted to operate or perform substantially in conformance with published Product specifications in effect at the time of sale, as set forth in the Production documentation, specifications and/or accompanying package inserts ("Documentation"). No claim of suitability for use in applications regulated by FDA is made. The warranty provided herein is valid only when used by properly trained individuals. Unless otherwise stated in the Documentation, this warranty is limited to one year from date of shipment when the Product is subjected to normal, proper and intended usage. This warranty does not extend to anyone other than the Buyer. Any model or sample furnished to Buyer is merely illustrative of the general type and quality of goods and does not represent that any Product will conform to such model or sample. NO OTHER WARRANTIES, EXPRESS OR IMPLED, ARE GRANTED INCLUDING WITHOUT LIMITATION, IMPLIED WARRANTIES OF MERCHANTABILITY, FITNESS FOR ANY PARTICULAR PURPOSE, OR NON INFRINGEMENT. BUYER'S EXCLUSIVE REMEDY FOR NON-CONFORMING PRODUCTS DURING THE WARRANTY PERIOD IS LIMITED TO REPAIR, REPLACEMENT OF OR REFUND FOR THE NON-CONFORMING PRODUCT(S) AT SELLER'S SOLE OPTION. THERE IS NO OBLIGATION TO REPAIR, REPLACE OR REFUND FOR PRODUCTS AS THE RESULT OF (I) ACCIDENT, DISASTER OR EVENT OF FORCE MAJEURE, (II) MISUSE, FAULT OR NEGLIGENCE OF OR BY BUYER, (III) USE OF THE PRODUCTS IN A MANNER FOR WHICH THEY WERE NOT DESIGNED, OR (IV) IMPROPER STORAGE AND HANDLING OF THE PRODUCTS. Unless otherwise expressly stated on the Product or in the documentation accompanying the Product, the Product is intended for research only and is not to be used for any other purpose, including without limitation, unauthorized commercial uses, in vitro diagnostic uses, ex vivo or in vivo therapeutic uses, or any type of consumption by or application to human or animals.

# bs-0573R

**[Primary Antibody]**

www.bioss.com.cn
sales@bioss.com.cn
techsupport@bioss.com.cn
400-901-9800

## Rabbit Anti-Cyclin E1 Polyclonal Antibody

### DATASHEET

**Host:** Rabbit

**Target Protein:** Cyclin E1

**IR:** Immunogen Range:375-411/411

**Clonality:** Polyclonal

**Isotype:** IgG

**Entrez Gene:** 25729

**Swiss Prot:** P39949

**Source:** KLH conjugated synthetic peptide derived from rat Cyclin E:375-411/411

**Purification:** affinity purified by Protein A

**Storage:** 0.01M TBS(pH7.4) with 1% BSA, 0.03% Proclin300 and 50% Glycerol. Shipped at 4℃. Store at -20 °C for one year. Avoid repeated freeze/thaw cycles.

**Background:** The protein encoded by this gene belongs to the highly conserved cyclin family, whose members are characterized by a dramatic periodicity in protein abundance through the cell cycle. Cyclins function as regulators of CDK kinases. Different cyclins exhibit distinct expression and degradation patterns which contribute to the temporal coordination of each mitotic event. This cyclin forms a complex with and functions as a regulatory subunit of CDK2, whose activity is required for cell cycle G1/S transition. This protein accumulates at the G1-S phase boundary and is degraded as cells progress through S phase. Overexpression of this gene has been observed in many tumors, which results in chromosome instability, and thus may contribute to tumorigenesis. This protein was found to associate with, and be involved in, the phosphorylation of NPAT protein (nuclear protein mapped to the ATM locus), which participates in cell-cycle regulated histone gene expression and plays a critical role in promoting cell-cycle progression in the absence of pRB. Two alternatively spliced transcript variants of this gene, which encode distinct isoforms, have been described. Two additional splice variants were reported but detailed nucleotide sequence information is not yet available. Transcript Variant: This variant (1) contains a different 5' end region, which includes an upstream in-frame translation start codon, when compared to variant 2. The encoded protein has a 15 aa longer N-terminus, as compared to isoform 2.

**Size:** 50ul

**Concentration:** 1mg/ml

**Applications:** WB(1:500-2000)
ELISA(1:5000-10000)
IHC-P(1:100-500)
IHC-F(1:100-500)
Flow-Cyt(1μg/Test)
IF(1:100-500)

**Cross Reactive Species:** Human
Mouse
Rat
.

For research use only. Not intended for diagnostic or therapeutic use.

### VALIDATION IMAGES

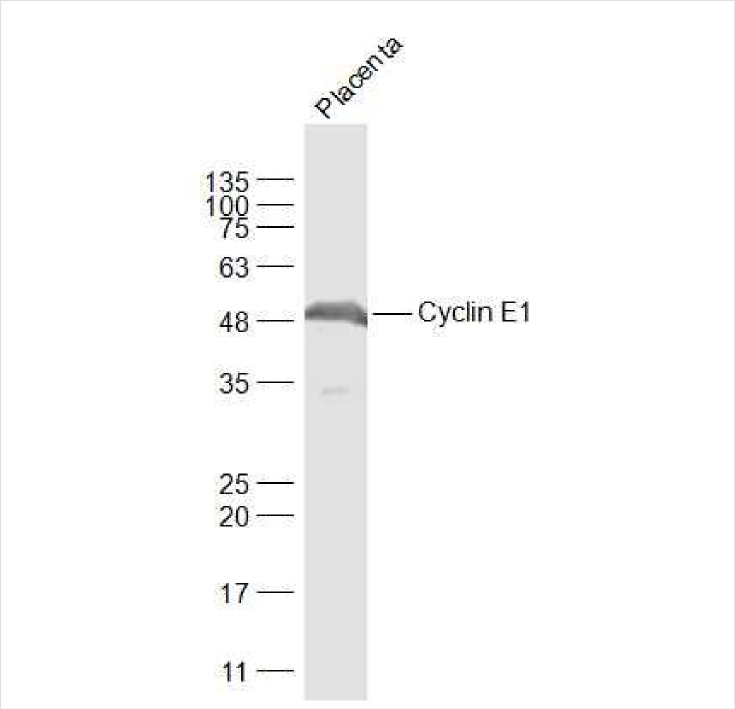


Sample:

Placenta (Mouse) Lysate at 40 ug

Primary: Anti-Cyclin E1 (bs-0573R) at 1/1000 dilution

Secondary: IRDye800CW Goat Anti-Rabbit IgG at 1/20000 dilution

Predicted band size: 45 kD

Observed band size: 48 kD

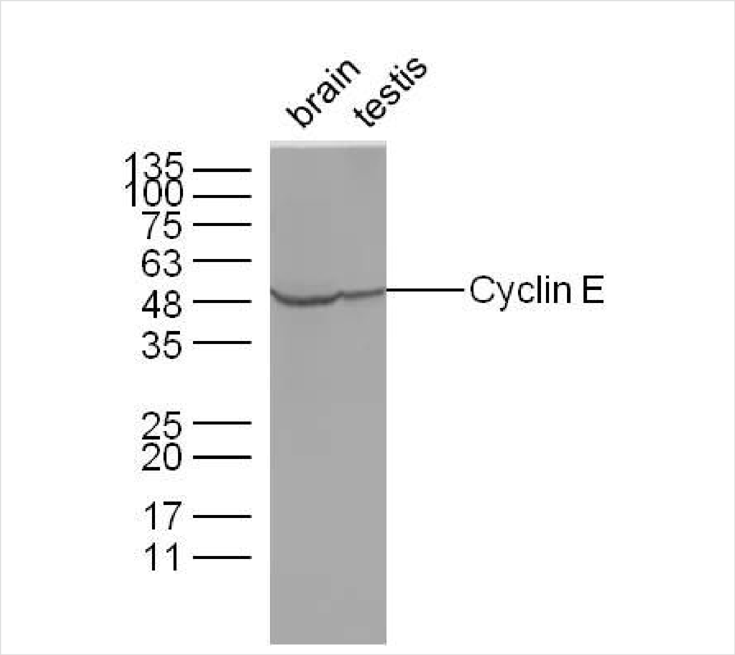


Sample:

Brain(Mouse) Lysate at 40 ug

Testis(Mouse) Lysate at 40 ug

Primary: Anti-Cyclin E (bs-0573R) at 1/300 dilution

Secondary: IRDye800CW Goat Anti-Rabbit IgG at 1/10000 dilution

Predicted band size: 45 kD

Observed band size: 48 kD

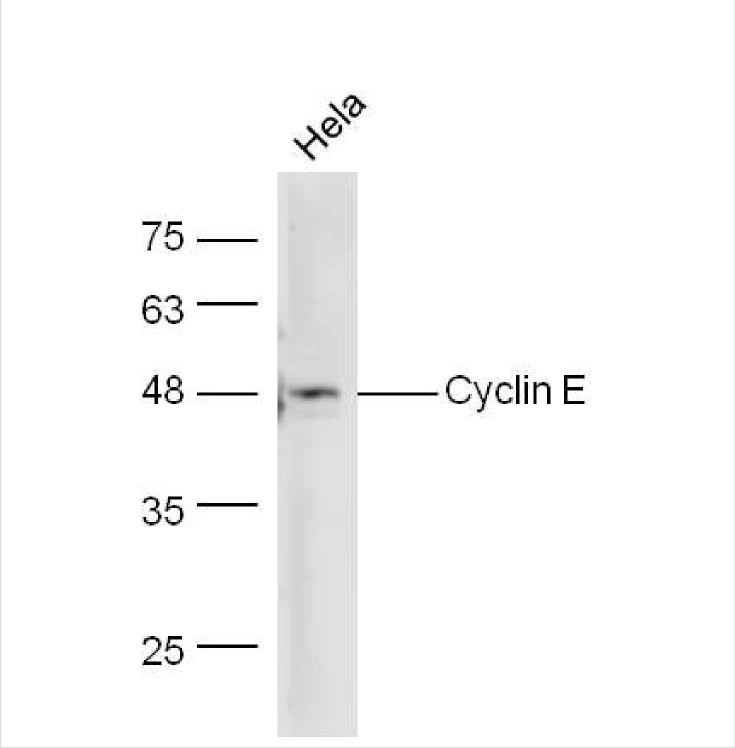


Sample: Hela Lysate at 40 ug

Primary: Anti-Cyclin E (bs-0573R) at 1/300 dilution

Secondary: IRDye800CW Goat Anti-Rabbit IgG at 1/10000 dilution

Predicted band size: 45 kD

Observed band size: 48 kD

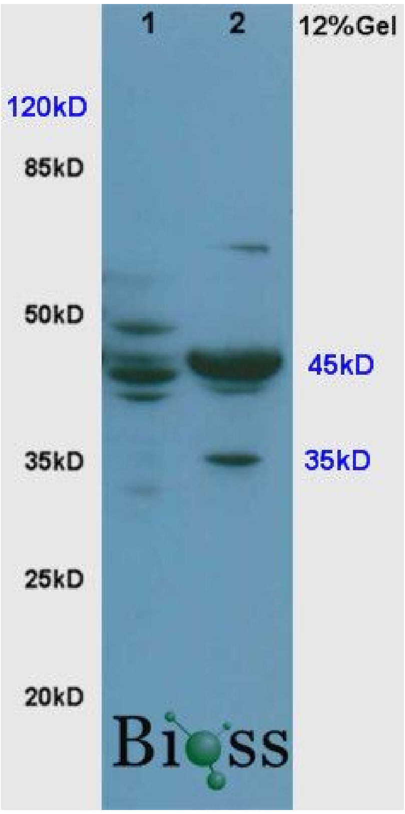


Sample:

Brain(Rat) lysate at30ug;

Lung(Rat) lysate at 30ug;

Primary: Anti-Cyclin E (bs-0573R) at 1:200;

Secondary: HRP conjugated Goat-Anti-Rabbit IgG(bse-0295G) at 1: 3000;

Predicted band size : 45kD

Observed band size : 45kD

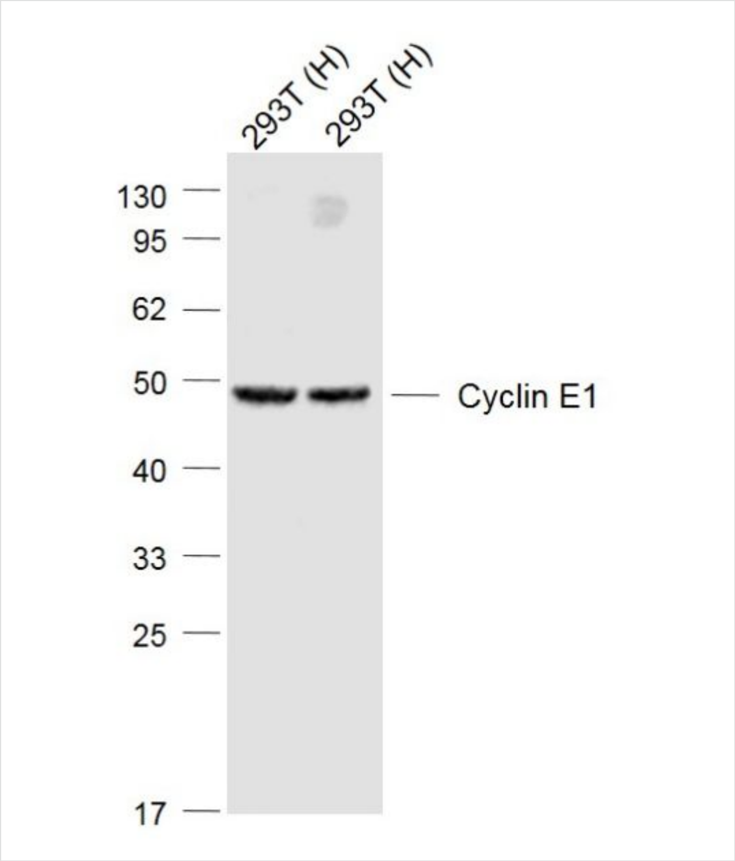


Sample:

Lane 1: 293T (Human) Cell Lysate at 30 ug

Lane 2: 293T (Human) Cell Lysate at 30 ug

Primary: Anti-Cyclin E1 (bs-0573R) at 1/1000 dilution

Secondary: IRDye800CW Goat Anti-Rabbit IgG at 1/20000 dilution

Predicted band size: 50 kD

Observed band size: 49 kD

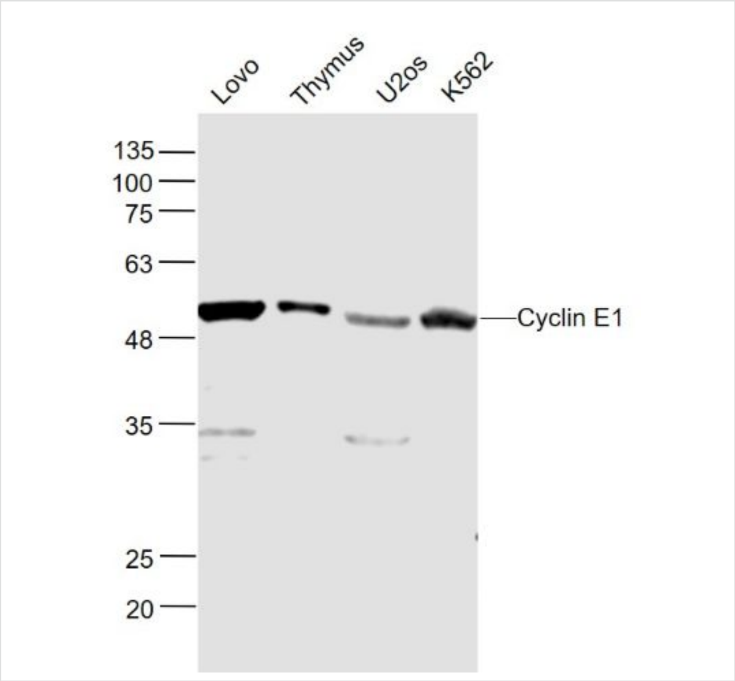


Sample:

Lovo (Human) Cell Lysate at 30 ug

Thymus (Mouse) Lysate at 40 ug

U2os (Human) Cell Lysate at 30 ug

K562 (Human) Cell Lysate at 30 ug

Primary: Anti- Cyclin E1 (bs-0573R) at 1/1000 dilution

Secondary: IRDye800CW Goat Anti-Rabbit IgG at 1/20000 dilution

Predicted band size: 45 kD

Observed band size: 50 kD

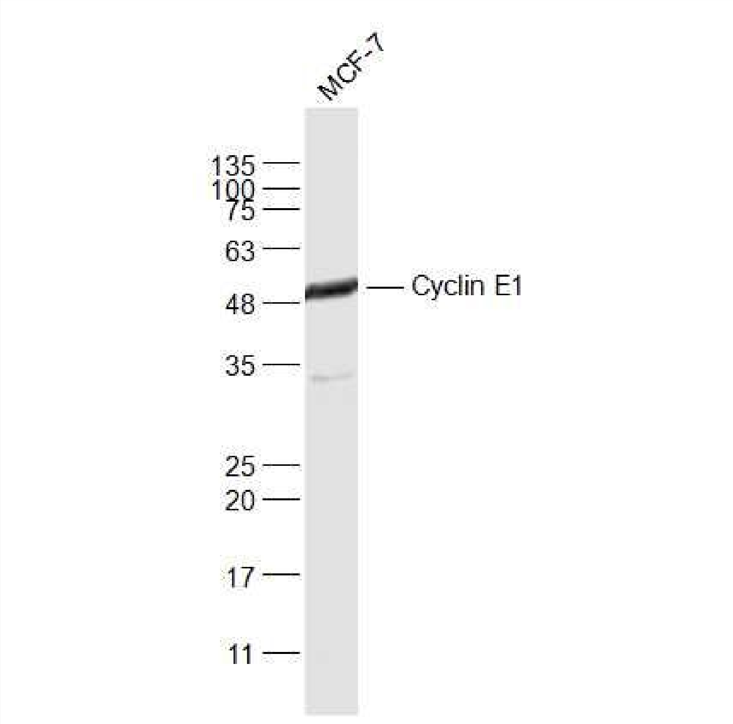


Sample:

MCF-7(Human) Cell Lysate at 30 ug

Primary: Anti-Cyclin E1 (bs-0573R) at 1/1000 dilution

Secondary: IRDye800CW Goat Anti-Rabbit IgG at 1/20000 dilution

Predicted band size: 45 kD

Observed band size: 48 kD

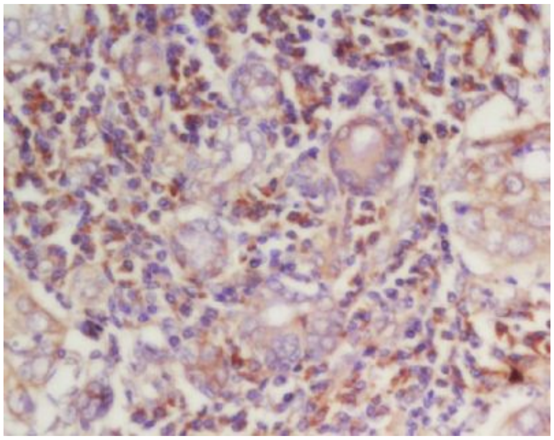

Tissue/cell: human laryngocarcinoma; 4% Paraformaldehyde-fixed and paraffin-embedded;

Antigen retrieval: citrate buffer ( 0.01M, pH 6.0 ), Boiling bathing for 15min; Block endogenous peroxidase by 3% Hydrogen peroxide for 30min; Blocking buffer (normal goat serum,C-0005) at 37℃ for 20 min;

Incubation: Anti-Cyclin-E Polyclonal Antibody, Unconjugated(bs-0573R) 1:200, overnight at 4°C, followed by conjugation to the secondary antibody(SP-0023) and DAB(C-0010) staining

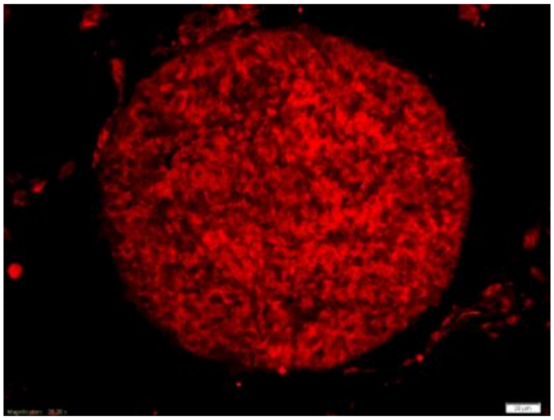

Tissue/cell: rat testis tissue;4% Paraformaldehyde-fixed and paraffin-embedded;

Antigen retrieval: citrate buffer ( 0.01M, pH 6.0 ), Boiling bathing for 15min; Blocking buffer (normal goat serum,C-0005) at 37℃ for 20 min;

Incubation: Anti-Cyclin E Polyclonal Antibody, Unconjugated(bs-0573R) 1:200, overnight at 4°C; The secondary antibody was Goat Anti-Rabbit IgG, Cy3 conjugated(bs-0295G-Cy3)used at 1:200 dilution for 40 minutes at 37°C.

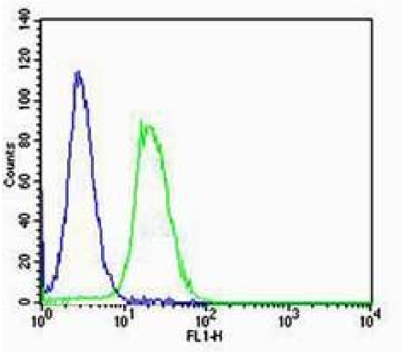


Cell: NIH/3T3

Concentration:1:100

Host/Isotype:Rabbit/IgG

Flow cytometric analysis of primary antibody (Cat#: bs-0573R) on NIH/3T3(green) compared with Rabbit IgG isotype control in the absence of primary antibody (blue) followed by Alexa Fluor 488-conjugated goat anti-rabbit IgG(H+L) secondary antibody .

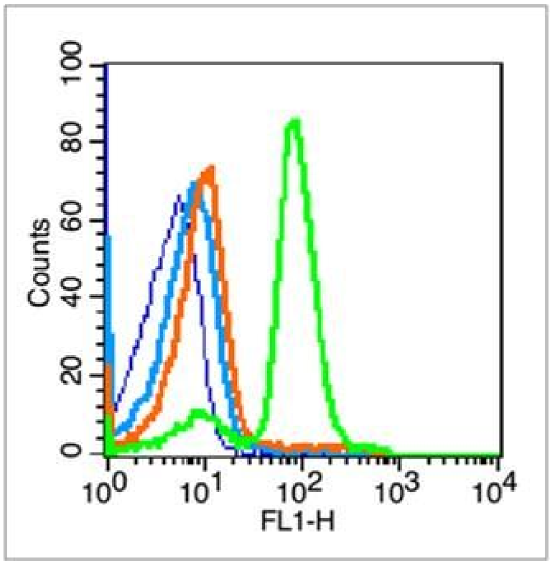


Blank control (blue line): Mouse spleen cells (blue).

Primary Antibody (green line): Rabbit Anti-Cyclin E1 antibody (bs-0573R)

Dilution: 1μg /10^6 cells;

Isotype Control Antibody (orange line): Rabbit IgG .

Secondary Antibody (white blue line): Goat anti-rabbit IgG-FITC

Dilution: 1μg /test.

Protocol

The cells were fixed with 70% ethanol (overninght at 4℃) and then permeabilized with 0.1% PBS-Tween for 20 min at room temperature. Cells stained with Primary Antibody for 30 min at room temperature. The cells were then incubated in 1 X PBS/2%BSA/10% goat serum to block non-specific protein-protein interactions followed by the antibody for 15 min at room temperature. The secondary antibody used for 40 min at room temperature. Acquisition of 20,000 events was performed.

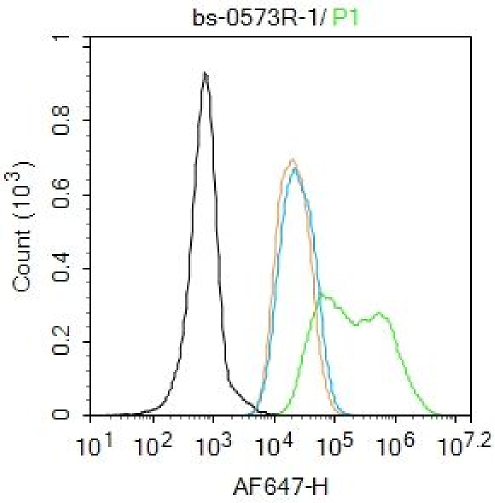


Blank control: MCF7.

Primary Antibody (green line): Rabbit Anti-Cyclin E1 antibody (bs-0573R)

Dilution: 2μg /10^6 cells;

Isotype Control Antibody (orange line): Rabbit IgG .

Secondary Antibody : Goat anti-rabbit IgG-AF647

Dilution: 1μg /test.

Protocol

The cells were fixed with 4% PFA (10min at room temperature)and then permeabilized with 90% ice-cold methanol for 20 min at-20℃.The cells were then incubated in 5%BSA to block non-specific protein-protein interactions for 30 min at room temperature .Cells stained with Primary Antibody for 30 min at room temperature. The secondary antibody used for 40 min at room temperature. Acquisition of 20,000 events was performed.

## PRODUCT SPECIFIC PUBLICATIONS


- Luya Pu. et al. Icariin arrests cell cycle progression and induces cell apoptosis through the mitochondrial pathway in human fibroblast-like synoviocytes. Eur J Pharmacol. 2021 Dec;912:174585 Read more>>
- Ji K et al. Differential Expression of lncRNAs and predicted target genes in normal mouse melanocytes and B16 cells. Experimental Dermatology. 2018. Read more>>
- Zhang J et al. Silica nanoparticles induce abnormal mitosis and apoptosis via PKC-δ mediated negative signaling pathway in GC-2 cells of mice. Chemosphere,2018 208, 942–950. Read more>>
- Li et al. Up-Regulation of Long Noncoding RNA SRA Promotes Cell Growth, Inhibits Cell Apoptosis, and Induces Secretion of Estradiol and Progesterone in Ovarian Granular Cells of Mice. (2018) Med.Sci.Monit. 24:2384-2390 Read more>>
- Lv et al. Synthesis, biological evaluation and mechanism studies of deoxytylophorinine and its derivatives as potential anticancer agents. (2012) PLoS.On. 7:e30342 Read more>>
- Muhammad T et al. Aloperine in combination with therapeutic adenoviral vector synergistically suppressed the growth of non-small cell lung cancer. J Cancer Res Clin Oncol. 2020 Feb 22. Read more>>
- Chang et al. Analysis of the ways and methods of signaling pathways in regulating cell cycle of NIH3T3 at transcriptional level. (2015) BMC.Cell.Biol. 16:25 Read more>>
- Chen et al. STAT1 inhibits human hepatocellular carcinoma cell growth through induction of p53 and Fbxw7. (2015) Cancer.Cell.Int. 15:111 Read more>>
- Zhang, Wen-feng, et al. "Angelica polysaccharides inhibit the growth and promote the apoptosis of U251 glioma cells in vitro and in vivo." Phytomedicine (2017). Read more>>
- Xu, X., et al. "Concentration-Dependent Diversifcation Effects of Free Cholesterol Loading on Macrophage Viability and Polarization." Cellular Physiology and Biochemistry 37.2 (2015): 419-431. Read more>>

**bs-1148R**

**[Primary Antibody]**

www.bioss.com.cn
sales@bioss.com.cn
techsupport@bioss.com.cn
400-901-9800

# Rabbit Anti-Cyclin D2 Polyclonal Antibody

## DATASHEET

**Host:** Rabbit

**Target Protein:** Cyclin D2

**IR:** Immunogen Range:161-288/288

**Clonality:** Polyclonal

**Isotype:** IgG

**Entrez Gene:** 894

**Swiss Prot:** P30279

**Source:** KLH conjugated synthetic peptide derived from the middle of human Cyclin D2:161-288/288

**Purification:** affinity purified by Protein A

**Storage:** 0.01M TBS(pH7.4) with 1% BSA, 0.03% Proclin300 and 50% Glycerol. Shipped at 4℃. Store at -20 °C for one year. Avoid repeated freeze/thaw cycles.

**Background:** The protein encoded by this gene belongs to the highly conserved cyclin family, whose members are characterized by a dramatic periodicity in protein abundance throughout the cell cycle. Cyclins function as regulators of CDK kinases. Different cyclins exhibit distinct expression and degradation patterns which contribute to the temporal coordination of each mitotic event. This cyclin forms a complex with and functions as a regulatory subunit of CDK4 or CDK6, whose activity is required for cell cycle G1/S transition. This protein has been shown to interact with tumor suppressor protein Rb and the expression of this gene is regulated positively by Rb. Mutations, amplification and overexpression of this gene, which alters cell cycle progression, are observed frequently in a variety of tumors and may contribute to tumorigenesis.

**Size:** 50ul

**Concentration:** 1mg/ml

**Applications:** ELISA(1:5000-10000)
IHC-P(1:100-500)
IHC-F(1:100-500)
Flow-Cyt(5μg/Test)
IF(1:100-500)

**Cross Reactive Species:** Human
Mouse
Rat
.

For research use only. Not intended for diagnostic or therapeutic use.

## VALIDATION IMAGES

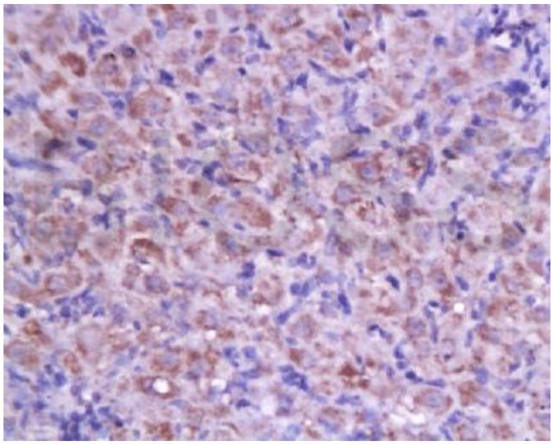

Tissue/cell: rat ovary tissue; 4% Paraformaldehyde-fixed and paraffin-embedded;
Antigen retrieval: citrate buffer ( 0.01M, pH 6.0 ), Boiling bathing for 15min; Block endogenous peroxidase by 3% Hydrogen peroxide for 30min; Blocking buffer (normal goat serum,C-0005) at 37℃ for 20 min;
Incubation: Anti-Cyclin D2 Polyclonal Antibody, Unconjugated(bs-1148R) 1:200, overnight at 4°C, followed by conjugation to the secondary antibody(SP-0023) and DAB(C-0010) staining

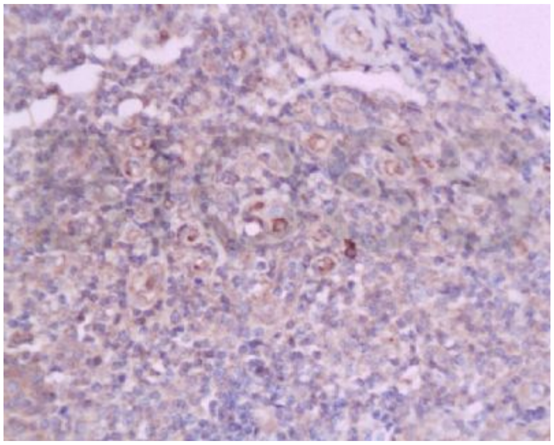

Tissue/cell: human flat moss tinea tissue; 4% Paraformaldehyde-fixed and paraffin-embedded;
Antigen retrieval: citrate buffer ( 0.01M, pH 6.0 ), Boiling bathing for 15min; Block endogenous peroxidase by 3% Hydrogen peroxide for 30min; Blocking buffer (normal goat serum,C-0005) at 37℃ for 20 min;
Incubation: Anti-Cyclin D2 Polyclonal Antibody, Unconjugated(bs-1148R) 1:200, overnight at 4°C, followed by conjugation to the secondary antibody(SP-0023) and DAB(C-0010) staining

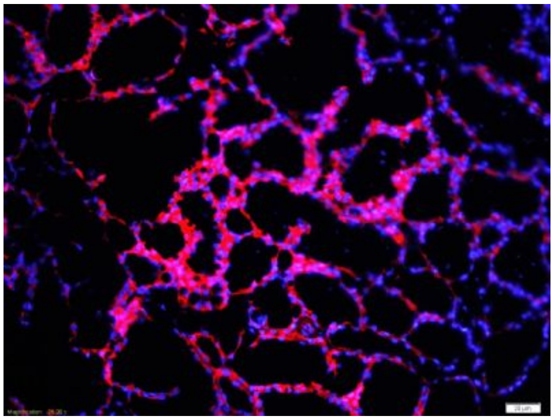

Tissue/cell: rat mammary tissue;4% Paraformaldehyde-fixed and paraffin-embedded;
Antigen retrieval: citrate buffer ( 0.01M, pH 6.0 ), Boiling bathing for 15min; Blocking buffer (normal goat serum,C-0005) at 37℃ for 20 min;
Incubation: Anti-Cyclin D2 Polyclonal Antibody, Unconjugated(bs-1148R) 1:200, overnight at 4°C; The secondary antibody was Goat Anti-Rabbit IgG, PE conjugated (bs-0295G-PE)used at 1:200 dilution for 40 minutes at 37°C. DAPI(5ug/ml,blue,C-0033) was used to stain the cell nuclei

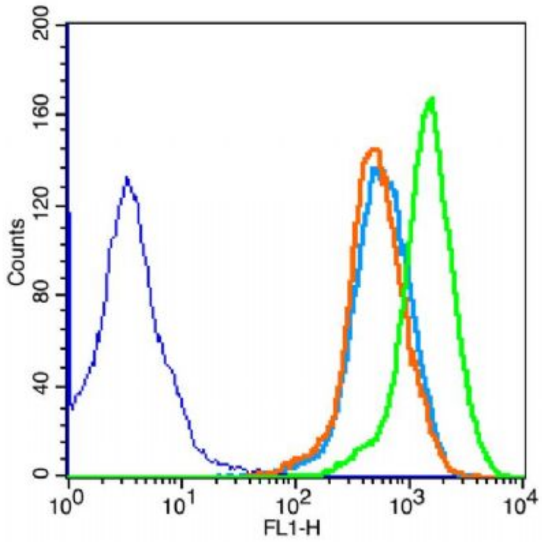


The figure annotation: The blue histogram is unstained cells. The Wathet Blue histogram is cells stained with secondary antibody(bs-0295G-FITC) alone. The Orange histogram is cells stained with rabbit IgG isotype control(bs-0295P) antibody plus secondary antibody. The green histogram is cells stained with Rabbit Anti-Cyclin D2 antibody (bs-1148R)plus secondary antibody.

.

Concebtration: 5μg/10^6 cells.

Positive control: MCF-7 cells.

## PRODUCT SPECIFIC PUBLICATIONS


- Wang, Y., et al. "Elevated toll-like receptor 3 inhibits pancreatic b-cell proliferation through G1 phase cell cycle arrest." Molecular and Cellular Endocrinology (2013) Read more>>

# bs-2006R

**[Primary Antibody]**

www.bioss.com.cn
sales@bioss.com.cn
techsupport@bioss.com.cn
400-901-9800

## Rabbit Anti-PCNA Polyclonal Antibody

## DATASHEET

**Host:** Rabbit

**Target Protein:** PCNA

**IR:** Immunogen Range:151-261/261

**Clonality:** Polyclonal

**Isotype:** IgG

**Entrez Gene:** 5111

**Swiss Prot:** P12004

**Source:** KLH conjugated synthetic peptide derived from human PCNA:151-261/261

**Purification:** affinity purified by Protein A

**Storage:** 0.01M TBS(pH7.4) with 1% BSA, 0.03% Proclin300 and 50% Glycerol. Shipped at 4℃. Store at -20 °C for one year. Avoid repeated freeze/thaw cycles.

**Background:** Proliferating cell nuclear antigen (PCNA) is a 28kDa nuclear protein associated with the cell cycle, a nuclear protein vital for cellular DNA synthesis. Proliferating cell nuclear antigen was originally identified by immunofluorescence as a nuclear protein whose appearance correlated with the proliferate state of the cell. PCNA is required for replication of DNA in vitro and has been identified as the auxiliary protein (cofactor) for DNA polymerase delta. The anti-PCNA antibodies react with the nuclei of proliferating cells. PCNA is essential for cellular DNA synthesis and is also required for the in vitro replication of simian virus 40 (SV40) DNA where it acts to coordinate leading and lagging strand synthesis at the replication fork. The PCNA protein may fulfil several separate roles in the cell nucleus associated with changes in its antigenic structure.

**Size:** 50ul

**Concentration:** 1mg/ml

**Applications:** WB(1:500-2000)
ELISA(1:5000-10000)
IHC-P(1:100-500)
IHC-F(1:100-500)
Flow-Cyt(1ug/Test)
IF(1:100-500)

**Cross Reactive Species:** Human
Mouse
Rat
Chicken
Pig
Cow
Rabbit
Sheep
Guinea Pig
.

For research use only. Not intended for diagnostic or therapeutic use.

## VALIDATION IMAGES

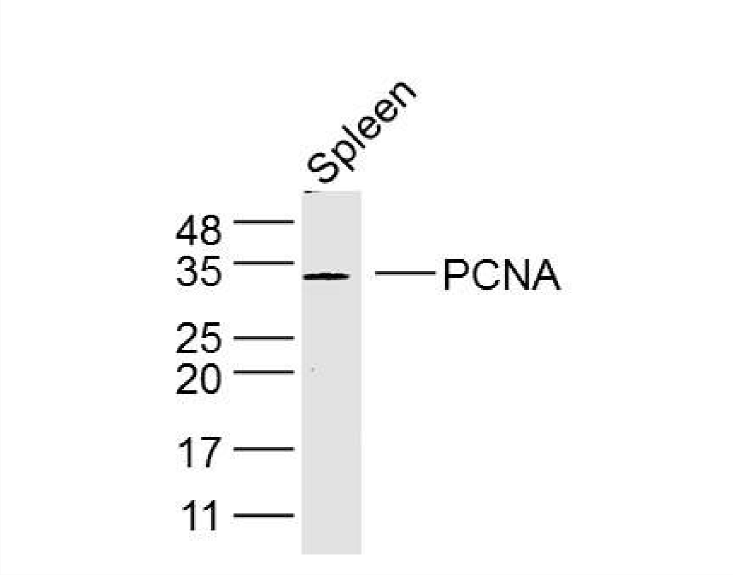


Sample:Spleen (Mouse) Lysate at 40 ug

Primary: Anti-PCNA (bs-2006R) at 1/300 dilution

Secondary: IRDye800CW Goat Anti-Rabbit IgG at 1/20000 dilution

Predicted band size: 29 kD

Observed band size: 32 kD

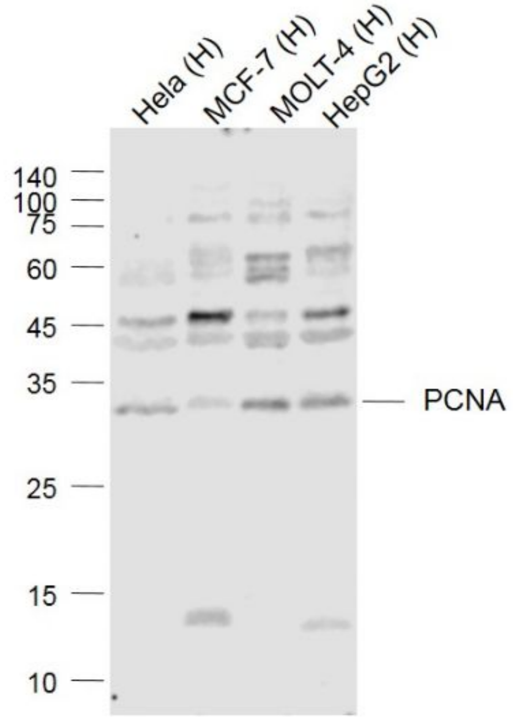


Sample:

Lane 1: Hela (Human) Cell Lysate at 30 ug

Lane 2: MCF-7 (Human) Cell Lysate at 30 ug

Lane 3: MOLT-4 (Human) Cell Lysate at 30 ug

Lane 4: HepG2 (Human) Cell Lysate at 30 ug

Primary: Anti-PCNA (bs-2006R) at 1/1000 dilution

Secondary: IRDye800CW Goat Anti-Rabbit IgG at 1/20000 dilution

Predicted band size: 32 kD

Observed band size: 32 kD

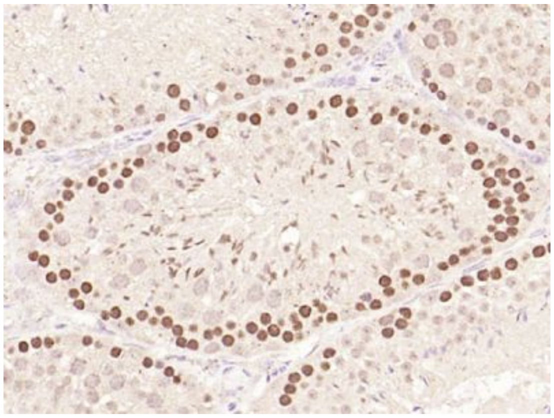

Paraformaldehyde-fixed, paraffin embedded (mouse testis); Antigen retrieval by boiling in sodium citrate buffer (pH6.0) for 15min; Block endogenous peroxidase by 3% hydrogen peroxide for 20 minutes; Blocking buffer (normal goat serum) at 37°C for 30min; Antibody incubation with (PCNA) Polyclonal Antibody, Unconjugated (bs-2006R) at 1:200 overnight at 4°C, followed by operating according to SP Kit(Rabbit) (sp-0023) instructionsand DAB staining.

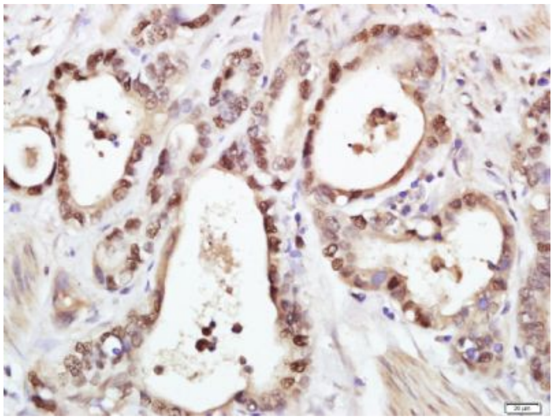

Paraformaldehyde-fixed, paraffin embedded (human gastric carcinoma); Antigen retrieval by boiling in sodium citrate buffer (pH6.0) for 15min; Block endogenous peroxidase by 3% hydrogen peroxide for 20 minutes; Blocking buffer (normal goat serum) at 37°C for 30min; Antibody incubation with (PCNA) Polyclonal Antibody, Unconjugated (bs-2006R) at 1:200 overnight at 4°C, followed by a conjugated secondary (sp-0023) for 20 minutes and DAB staining.

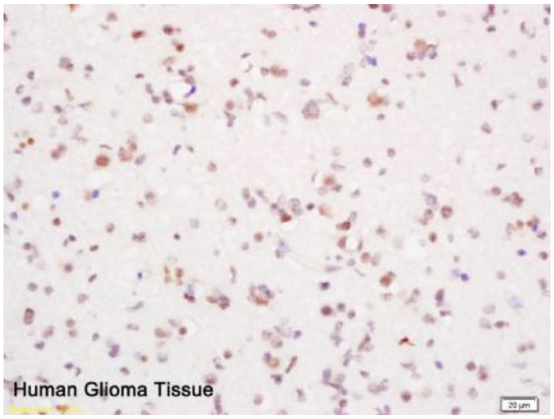


Tissue/cell: human glioma tissue; 4% Paraformaldehyde-fixed and paraffin-embedded;
Antigen retrieval: citrate buffer ( 0.01M, pH 6.0 ), Boiling bathing for 15min; Block endogenous peroxidase by 3% Hydrogen peroxide for 30min; Blocking buffer (normal goat serum,C-0005) at 37℃ for 20 min;
Incubation: Anti-PCNA Polyclonal Antibody, Unconjugated(bs-2006R) 1:200, overnight at 4°C, followed by conjugation to the secondary antibody(SP-0023) and DAB(C-0010) staining

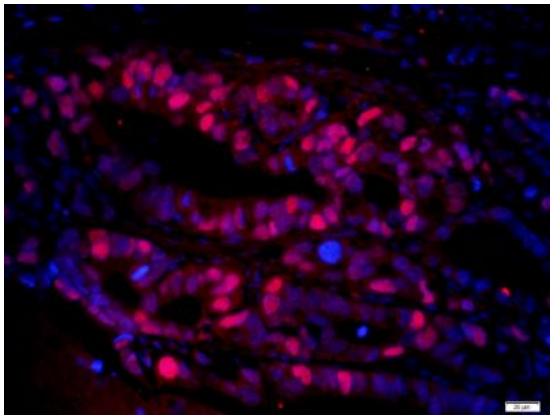

Paraformaldehyde-fixed, paraffin embedded (human gastric carcinoma); Antigen retrieval by boiling in sodium citrate buffer (pH6) for 15min; Block endogenous peroxidase by 3% hydrogen peroxide for 20 minutes; Blocking buffer (normal goat serum) at 37°C for 30min; Antibody incubation with (PCNA) Polyclonal Antibody, Unconjugated (bs-0754R) at 1:200 overnight at 4°C, followed by a conjugated secondary (bs-0295G-Cy3) at [1:500] for 90 minutes and DAPI staining of the nuclei.

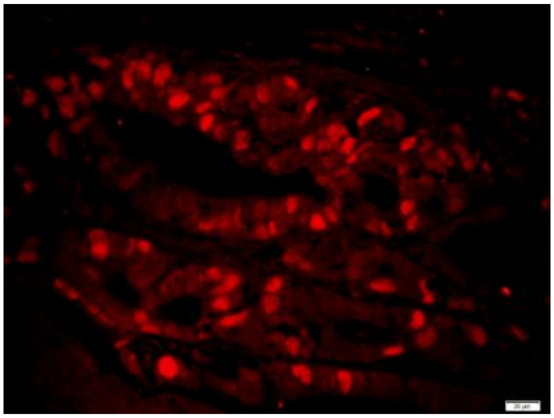

Paraformaldehyde-fixed, paraffin embedded (human gastric carcinoma); Antigen retrieval by boiling in sodium citrate buffer (pH6) for 15min; Block endogenous peroxidase by 3% hydrogen peroxide for 20 minutes; Blocking buffer (normal goat serum) at 37°C for 30min; Antibody incubation with (PCNA) Polyclonal Antibody, Unconjugated (bs-2006R) at 1:200 overnight at 4°C, followed by a conjugated secondary (bs-0295G-Cy3) at [1:500] for 90 minutes and DAPI staining of the nuclei.

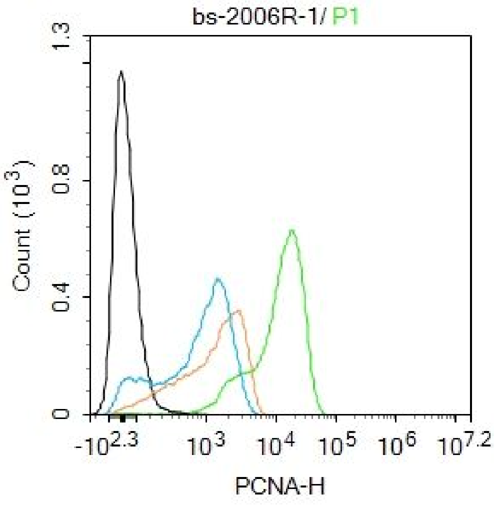


Blank control:Jurkat.
Primary Antibody (green line): Rabbit Anti-PCNA antibody (bs-2006R)
Dilution: 1ug/Test;
Secondary Antibody : Goat anti-rabbit IgG-FITC
Dilution: 0.5ug/Test.
Protocol
The cells were fixed with 4% PFA (10min at room temperature)and then permeabilized with 90% ice-cold methanol for 20 min at -20℃.The cells were then incubated in 5%BSA to block non-specific protein-protein interactions for 30 min at room temperature

.Cells stained with Primary Antibody for 30 min at room temperature. The secondary antibody used for 40 min at room temperature. Acquisition of 20,000 events was performed.

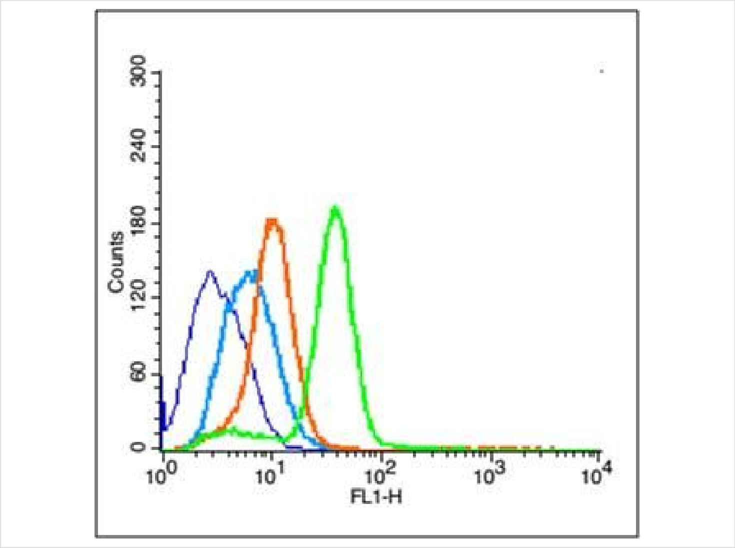


Blank control (blue line): U251 (blue).

Primary Antibody (green line): Rabbit Anti-PCNA antibody (bs-2006R)

Dilution: 3μg /10^6 cells;

Isotype Control Antibody (orange line): Rabbit IgG .

Secondary Antibody (white blue line): Goat anti-rabbit IgG-PE

Dilution: 1μg /test.

Protocol

The cells were fixed with 2% paraformaldehyde (10 min)and then permeabilized with 0.1% PBS-Tween for 20 min at room temperature. Cells stained with Primary Antibody for 30 min at room temperature. The cells were then incubated in 1 X PBS/2%BSA/10% goat serum to block non-specific protein-protein interactions followed by the antibody for 15 min at room temperature. The secondary antibody used for 40 min at room temperature. Acquisition of 20,000 events was performed.

## PRODUCT SPECIFIC PUBLICATIONS


- Wan Boyang. et al. Zearalenone promotes follicle development through activating SIRT1/PGC-1α signaling pathway in the ovaries of weaned gilts. J Anim Sci. 2022 Feb;: Read more>>
- Suihui Li. et al. Hsa_circ_0048674 facilitates hepatocellular carcinoma progression and natural killer cell exhaustion depending on the regulation of miR-223-3p/PDL1. Histol Histopathol. 2022 Feb 21;18440 Read more>>
- Yu TT. et al. Chlorin e6-Induced Photodynamic Effect Polarizes the Macrophage Into an M1 Phenotype Through Oxidative DNA Damage and Activation of STING.. Front Pharmacol. 2022 Mar;13:837784-837784 Read more>>
- Ning Han. et al. Ferroptosis triggered by dihydroartemisinin facilitates chlorin e6 induced photodynamic therapy by inhibiting GPX4 and enhancing ROS. Eur J Pharmacol. 2022 Feb;:174797 Read more>>
- Yu, Ting-Ting. et al. Harnessing chlorin e6 loaded by functionalized iron oxide nanoparticles linked with glucose for target photodynamic therapy and improving of the immunogenicity of lung cancer. J Cancer Res Clin. 2022 Jan;:1-13 Read more>>
- Qing Li. et al. Fumonisin B1 Inhibits Cell Proliferation and Decreases Barrier Function of Swine Umbilical Vein Endothelial Cells. Toxins. 2021 Dec;13(12):863 Read more>>
- Wang C et al. 4-Amino-2-trifluoromethyl-phenyl retinate induced differentiation of human myelodysplastic syndromes SKM-1 cell lines by up-regulating DDX23. Biomed Pharmacother. 2019 Dec 16;123:109736. Read more>>
- Zhang,et al.Lysosomal deposition of copper oxide nanoparticles triggers HUVEC cells death.(2018) Biomaterials. 161:228-239. Read more>>
- Jolly,et al.Targeted endothelial gene deletion of Triggering Receptor Expressed on Myeloid cells-1 protects mice during septic shock.(2018) Cardiovascular Research. 114:907-918. Read more>>
- Zhou et al. Induced pluripotent stem cell-conditioned medium suppresses pulmonary fibroblast-to-myofibroblast differentiation via the inhibition of TGF-β1/Smad pathway. (2018) Int.J.Mol.Med. 41:473-484 Read more>>
- Zhao, Yong, et al. "Inhibition of peripubertal sheep mammary gland development by cysteamine through reducing progesterone and growth factor production." Theriogenology (2016). Read more>>
- Yang Zhang. et al. Plumbagin Inhibits Proliferation, Migration, and Invasion of Retinal Pigment Epithelial Cells Induced by FGF-2. Tissue Cell. 2021 Oct;72:101547 Read more>>
- Wu Z et al. Compound xiebai capsule alleviates pulmonary vascular remodeling in monocrotaline-induced pulmonary arterial hypertension in rats. Tropical Journal of Pharmaceutical Research January 2020; 19 (1): 107-114. Read more>>

- Jin J et al. A novel S1P1 modulator IMMH002 ameliorates psoriasis in multiple animal models. Acta Pharmaceutica Sinica B. 2019. Read more>>
- Gao Y et al. Ginsenoside Re inhibits vascular neointimal hyperplasia in balloon-injured carotid arteries through activating the eNOS/NO/cGMP pathway in rats Y Gao, CY Gao, P Zhu, SF Xu, YM Luo, J Deng… - Biomedicine & …, 2018Biomed Pharmacother. 2018 Oct;106:1091-1097. Read more>>
- Zhou,et al.CXCR4 antagonist AMD3100 enhances the response of MDA-MB-231 triple-negative breast cancer cells to ionizing radiation. (2018) Cancer Letters. 418:196-203. Read more>>
- Chai et al. Hypoxia induces pulmonary arterial fibroblast proliferation, migration, differentiation and vascular remodeling via the PI3K/Akt/p70S6K signaling pathway. (2018) Int.J.Mol.Med. 41:2461-2472 Read more>>
- Ji, Wei, et al. "Triptolide inhibits proliferation, differentiation and induces apoptosis of osteoblastic MC3T3-E1 cells." Molecular Medicine Reports. Read more>>
- Liang, Sixian, et al. "Silencing of CXCR4 sensitizes triple-negative breast cancer cells to cisplatin." Oncotarget 6.2 (2015): 1020-1030. Read more>>

**bs-2758R**

**[Primary Antibody]**

www.bioss.com.cn
sales@bioss.com.cn
techsupport@bioss.com.cn
400-901-9800

# Rabbit Anti-Cdc25A Polyclonal Antibody

## DATASHEET

**Host:** Rabbit

**Target Protein:** Cdc25A

**IR:** Immunogen Range:451-524/524

**Clonality:** Polyclonal

**Isotype:** IgG

**Entrez Gene:** 993

**Swiss Prot:** P30304

**Source:** KLH conjugated synthetic peptide derived from human cdc25A:451-524/524

**Purification:** affinity purified by Protein A

**Storage:** 0.01M TBS(pH7.4) with 1% BSA, 0.03% Proclin300 and 50% Glycerol. Shipped at 4℃. Store at -20 °C for one year. Avoid repeated freeze/thaw cycles.

**Background:** CDC25A is a member of the CDC25 family of phosphatases. CDC25A is required for progression from G1 to the S phase of the cell cycle. It activates the cyclin-dependent kinase CDC2 by removing two phosphate groups. CDC25A is specifically degraded in response to DNA damage, which prevents cells with chromosomal abnormalities from progressing through cell division. CDC25A is an oncogene, although its exact role in oncogenesis has not been demonstrated. Two transcript variants encoding different isoforms have been found for this gene. [provided by RefSeq, Jul 2008]

**Size:** 50ul

**Concentration:** 1mg/ml

**Applications:** WB(1:500-2000)
ELISA(1:5000-10000)
IHC-P(1:100-500)
IHC-F(1:100-500)
IF(1:100-500)

**Cross Reactive Species:** Human
Mouse
Rat
Chicken
Dog
Cow
Rabbit
.

For research use only. Not intended for diagnostic or therapeutic use.

## VALIDATION IMAGES

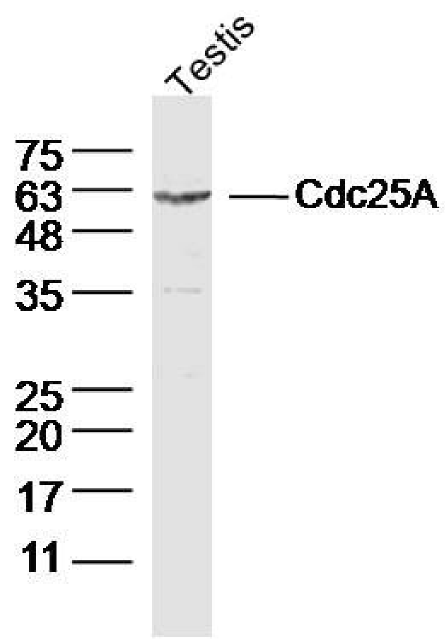


Sample: Testis (Mouse) Lysate at 40 ug

Primary: Anti- Cdc25A (bs-2758R) at 1/300 dilution

Secondary: IRDye800CW Goat Anti-Rabbit IgG at 1/20000 dilution

Predicted band size: 59 kD

Observed band size: 61 kD

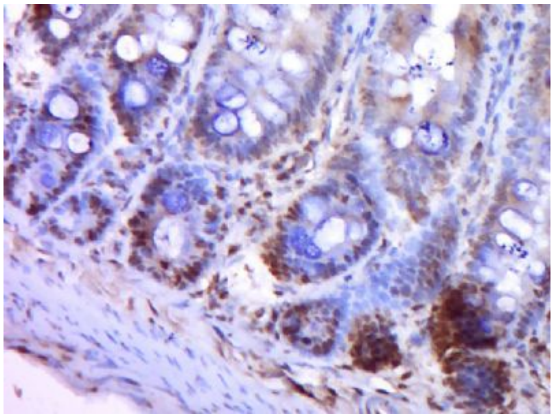

Paraformaldehyde-fixed, paraffin embedded (Rat small intestine); Antigen retrieval by boiling in sodium citrate buffer (pH6.0) for 15min; Block endogenous peroxidase by 3% hydrogen peroxide for 20 minutes; Blocking buffer (normal goat serum) at 37°C for 30min; Antibody incubation with (Cdc25A) Polyclonal Antibody, Unconjugated (bs-2758R) at 1:400 overnight at 4°C, followed by operating according to SP Kit(Rabbit) (sp-0023) instructionsand DAB staining.

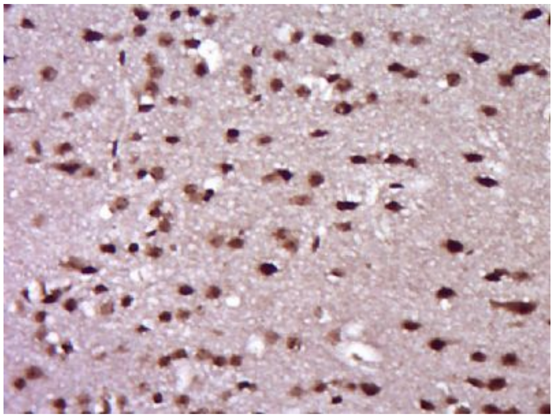

Paraformaldehyde-fixed, paraffin embedded (Mouse brain); Antigen retrieval by boiling in sodium citrate buffer (pH6.0) for 15min; Block endogenous peroxidase by 3% hydrogen peroxide for 20 minutes; Blocking buffer (normal goat serum) at 37°C for 30min; Antibody incubation with (Cdc25A) Polyclonal Antibody, Unconjugated (bs-2758R) at 1:400 overnight at 4°C, followed by operating according to SP Kit(Rabbit) (sp-0023) instructionsand DAB staining.

## PRODUCT SPECIFIC PUBLICATIONS


- Wei, Jialiu, et al. "Endosulfan induces cell dysfunction through cycle arrest resulting from DNA damage and DNA damage response signaling pathways." Science of The Total Environment 589 (2017): 97-106. Read more>>

# bs-3535R

**[Primary Antibody]**

www.bioss.com.cn
sales@bioss.com.cn
techsupport@bioss.com.cn
400-901-9800

## Rabbit Anti-PLK1 Polyclonal Antibody

## DATASHEET

**Host:** Rabbit

**Target Protein:** PLK1

**IR:** Immunogen Range:201-300/603

**Clonality:** Polyclonal

**Isotype:** IgG

**Entrez Gene:** 5347

**Swiss Prot:** P53350

**Source:** KLH conjugated synthetic peptide derived from human PLK1:201-300/603

**Purification:** affinity purified by Protein A

**Storage:** 0.01M TBS(pH7.4) with 1% BSA, 0.03% Proclin300 and 50% Glycerol. Shipped at 4℃. Store at -20 °C for one year. Avoid repeated freeze/thaw cycles.

**Background:** The Ser/Thr protein kinase encoded by this gene belongs to the CDC5/Polo subfamily. It is highly expressed during mitosis and elevated levels are found in many different types of cancer. Depletion of this protein in cancer cells dramatically inhibited cell proliferation and induced apoptosis; hence, it is a target for cancer therapy. [provided by RefSeq, Sep 2015]

**Size:** 50ul

**Concentration:** 1mg/ml

**Applications:** WB(1:500-2000)
ELISA(1:5000-10000)
IHC-P(1:100-500)
IHC-F(1:100-500)
ICC(1:100-500)
IF(1:100-500)

**Cross Reactive Species:** Human
Mouse
Rat
Dog
Pig
Rabbit
.

For research use only. Not intended for diagnostic or therapeutic use.

## VALIDATION IMAGES

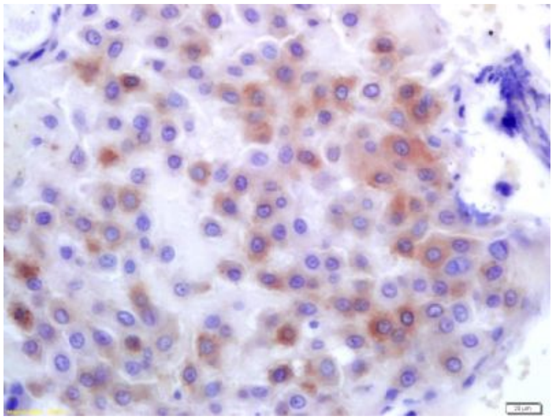

Tissue/cell: human placenta tissue; 4% Paraformaldehyde-fixed and paraffin-embedded; Antigen retrieval: citrate buffer ( 0.01M, pH 6.0 ), Boiling bathing for 15min; Block endogenous peroxidase by 3% Hydrogen peroxide for 30min; Blocking buffer (normal goat serum,C-0005) at 37℃ for 20 min; Incubation: Anti-PLK1 Polyclonal Antibody, Unconjugated(bs-3535R) 1:200, overnight at 4°C, followed by conjugation to the secondary antibody(SP-0023) and DAB(C-0010) staining

## PRODUCT SPECIFIC PUBLICATIONS


- Gaizhen Kuang. et al. Near-Infrared Light-Triggered Polyprodrug/siRNA Loaded Upconversion Nanoparticles for Multi-Modality Imaging and Synergistic Cancer Therapy. 2021 Jul 03 Read more>>
- Chen Y et al. PLK1 regulates hepatic stellate cell activation and liver fibrosis through Wnt/β-catenin signalling pathway. J Cell Mol Med. 2020 Jul;24(13):7405-7416. Read more>>
- Gibori et al. Amphiphilic nanocarrier-induced modulation of PLK1 and miR-34a leads to improved therapeutic response in pancreatic cancer. (2018) Nat.Commun. 9:16 Read more>>

# bs-5739R

[Primary Antibody]

www.bioss.com.cn
sales@bioss.com.cn
techsupport@bioss.com.cn
400-901-9800

## Rabbit Anti-Cyclin A1 Polyclonal Antibody

DATASHEET

**Host:** Rabbit

**Target Protein:** Cyclin A1

**IR:** Immunogen Range:211-310/465

**Clonality:** Polyclonal

**Isotype:** IgG

**Entrez Gene:** 8900

**Swiss Prot:** P78396

**Source:** KLH conjugated synthetic peptide derived from human Cyclin A1:211-310/465

**Purification:** affinity purified by Protein A

**Storage:** 0.01M TBS(pH7.4) with 1% BSA, 0.03% Proclin300 and 50% Glycerol. Shipped at 4℃. Store at -20 °C for one year. Avoid repeated freeze/thaw cycles.

**Background:** Cyclin A1 belongs to the highly conserved cyclin family, whose members are characterized by a dramatic periodicity in protein abundance through the cell cycle. Cyclins function as regulators of CDK kinases. Different cyclins exhibit distinct expression and degradation patterns which contribute to the temporal coordination of each mitotic event. Cyclin A1 is expressed in testis and brain, as well as in several leukemic cell lines, and is thought to primarily function in the control of the germline meiotic cell cycle. It binds both CDK2 and CDC2 kinases, which give two distinct kinase activities, one appearing in S phase, the other in G2, and thus regulate separate functions in cell cycle. Cyclin A1 was found to bind to important cell cycle regulators, such as Rb family proteins, transcription factor E2F-1, and the p21 family proteins.

**Size:** 50ul

**Concentration:** 1mg/ml

**Applications:** ELISA(1:5000-10000)
IHC-P(1:100-500)
IHC-F(1:100-500)
IF(1:100-500)

**Cross Reactive Species:** Human
Mouse
Rat
Dog
Pig
Cow
Horse
Rabbit
.

For research use only. Not intended for diagnostic or therapeutic use.

## VALIDATION IMAGES

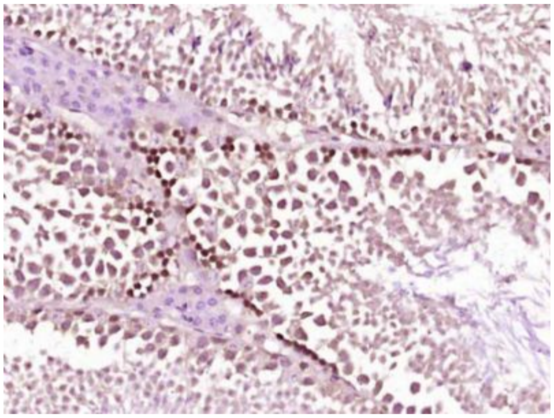

Paraformaldehyde-fixed, paraffin embedded (Mouse testis); Antigen retrieval by boiling in sodium citrate buffer (pH6.0) for 15min; Block endogenous peroxidase by 3% hydrogen peroxide for 20 minutes; Blocking buffer (normal goat serum) at 37°C for 30min; Antibody incubation with (Cyclin A1) Polyclonal Antibody, Unconjugated (bs-5739R) at 1:400 overnight at 4°C, followed by operating according to SP Kit(Rabbit) (sp-0023) instructionsand DAB staining.

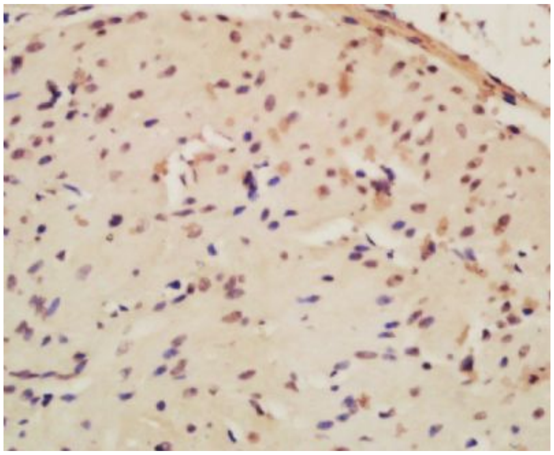

Tissue/cell: rat ovary tissue; 4% Paraformaldehyde-fixed and paraffin-embedded;
Antigen retrieval: citrate buffer ( 0.01M, pH 6.0 ), Boiling bathing for 15min; Block endogenous peroxidase by 3% Hydrogen peroxide for 30min; Blocking buffer (normal goat serum,C-0005) at 37℃ for 20 min;
Incubation: Anti-Cyclin A1 Polyclonal Antibody, Unconjugated(bs-5739R) 1:200, overnight at 4°C, followed by conjugation to the secondary antibody(SP-0023) and DAB(C-0010) staining

## PRODUCT SPECIFIC PUBLICATIONS


- A K Shendgeet al. A natural flavonoid, apigenin isolated from Clerodendrum viscosum leaves, induces G2/M phase cell cycle arrest and apoptosis in MCF-7 cells through the regulation of p53 and caspase-cascade pathway. Clin Transl Oncol . 2020 Jul 27. Read more>>
- Zhang, Jin, et al. "Silica nanoparticles induce start inhibition of meiosis and cell cycle arrest via down-regulating meiotic relevant factors." Toxicology Research (2016). Read more>>
- Wang, Chengke, and Zhenxin Wang. "Studying the relationship between cell cycle and Alzheimer’s disease by gold nanoparticle probes." Analytical Biochemistry (2015). Read more>>
- Zhao, Yong, et al. "Inhibition of peripubertal sheep mammary gland development by cysteamine through reducing progesterone and growth factor production." Theriogenology (2016). Read more>>
- Anil Khushalrao Shendge. et al. The natural flavones, acacetin and apigenin, induce Cdk-Cyclin mediated G2/M phase arrest and trigger ROS-mediated apoptosis in glioblastoma cells. Mol Biol Rep. 2021 Jan;48(1):539-549 Read more>>
- Zhang L et al. Silica Nanoparticles exacerbates reproductive toxicity development in high-fat diet-treated Wistar rats. J Hazard Mater. 2019 Oct 1:121361. Read more>>
- Hao X et al. Reverse the down regulation of miR-92b-3p by hypoxia can suppress the proliferation of pulmonary artery smooth muscle cells by targeting USP28. Biochem Biophys Res Commun. 2018 Sep 18;503(4):3064-3077. Read more>>
- Ghate, N. B., et al. "Sundew plant, a potential source of anti-inflammatory agents, selectively induces G2/M arrest and apoptosis in MCF-7 cells through upregulation of p53 and Bax/Bcl-2 ratio." Cell Death Discovery 2 (2016). Read more>>
- Ghate, Nikhil Baban, et al. "An Antioxidant Extract of Tropical Lichen, Parmotrema reticulatum, Induces Cell Cycle Arrest and Apoptosis in Breast Carcinoma Cell Line MCF-7." PLOS ONE 8.12 (2013): e82293. Read more>>

**bs-14356R**

**[Primary Antibody]**

www.bioss.com.cn
sales@bioss.com.cn
techsupport@bioss.com.cn
400-901-9800

# Rabbit Anti-DNA Polymerase epsilon p59 Polyclonal Antibody

## DATASHEET

**Host:** Rabbit

**Target Protein:** DNA Polymerase epsilon p59

**IR:** Immunogen Range:101-200/527

**Clonality:** Polyclonal

**Isotype:** IgG

**Entrez Gene:** 5427

**Swiss Prot:** P56282

**Source:** KLH conjugated synthetic peptide derived from human DNA Polymerase epsilon p59:101-200/527

**Purification:** affinity purified by Protein A

**Storage:** 0.01M TBS(pH7.4) with 1% BSA, 0.03% Proclin300 and 50% Glycerol. Shipped at 4℃. Store at -20 °C for one year. Avoid repeated freeze/thaw cycles.

**Background:** DNA replication, recombination and repair, all of which are necessary for genome stability, require the presence of exonucleases

**Size:** 50ul

**Concentration:** 1mg/ml

**Applications:** ELISA(1:5000-10000)
IHC-P(1:100-500)
IHC-F(1:100-500)
ICC(1:100-500)
IF(1:100-500)

**Cross Reactive Species:** Human
Mouse
Rat
Cow
Horse
Sheep
.

For research use only. Not intended for diagnostic or therapeutic use.

## VALIDATION IMAGES

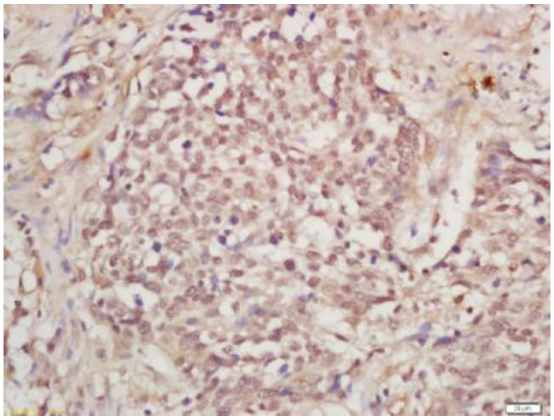

Tissue/cell: human laryngo carcinoma; 4% Paraformaldehyde-fixed and paraffin-embedded; Antigen retrieval: citrate buffer ( 0.01M, pH 6.0 ), Boiling bathing for 15min; Block endogenous peroxidase by 3% Hydrogen peroxide for 30min; Blocking buffer (normal goat serum,C-0005) at 37℃ for 20 min; Incubation: Anti-POLE2 Polyclonal Antibody, Unconjugated(bs-14356R) 1:200, overnight at 4°C, followed by conjugation to the secondary antibody(SP-0023) and DAB(C-0010) staining

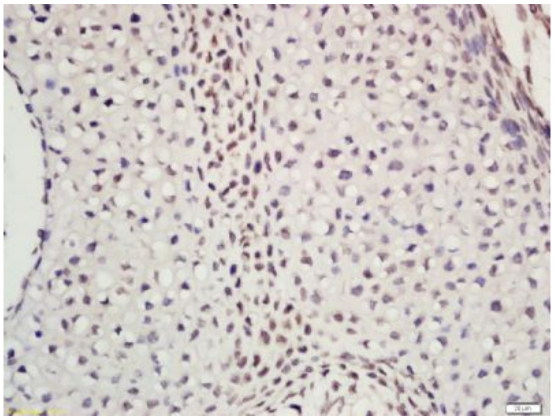

Tissue/cell: Mouse embryos tissue; 4% Paraformaldehyde-fixed and paraffin-embedded; Antigen retrieval: citrate buffer ( 0.01M, pH 6.0 ), Boiling bathing for 15min; Block endogenous peroxidase by 3% Hydrogen peroxide for 30min; Blocking buffer (normal goat serum,C-0005) at 37℃ for 20 min; Incubation: Anti-DNA Polymerase epsilon p59 Polyclonal Antibody, Unconjugated(bs-14356R) 1:200, overnight at 4°C, followed by conjugation to the secondary antibody(SP-0023) and DAB(C-0010) staining

## PRODUCT SPECIFIC PUBLICATIONS


- Zhang Chuanjie. et al. Targeting POLE2 Creates a Novel Vulnerability in Renal Cell Carcinoma via Modulating Stanniocalcin 1. Front Cell Dev Biol. 2021 Feb;9:228 Read more>>
- Yongjun Zhu. et al. POLE2 knockdown reduce tumorigenesis in esophageal squamous cells. Cancer Cell Int. 2020 Dec;20(1):1-12 Read more>>

# bs-20596R

[Primary Antibody]

www.bioss.com.cn
sales@bioss.com.cn
techsupport@bioss.com.cn
400-901-9800

## Rabbit Anti-Cyclin D1 Polyclonal Antibody

## DATASHEET

**Host:** Rabbit

**Target Protein:** Cyclin D1

**IR:** Immunogen Range:101-200/295

**Clonality:** Polyclonal

**Isotype:** IgG

**Entrez Gene:** 595

**Swiss Prot:** P24385

**Source:** KLH conjugated synthetic peptide derived from human Cyclin D1:101-200/295

**Purification:** affinity purified by Protein A

**Storage:** 0.01M TBS(pH7.4) with 1% BSA, 0.03% Proclin300 and 50% Glycerol. Shipped at 4℃. Store at -20 °C for one year. Avoid repeated freeze/thaw cycles.

**Background:** The protein encoded by this gene belongs to the highly conserved cyclin family, whose members are characterized by a dramatic periodicity in protein abundance throughout the cell cycle. Cyclins function as regulators of CDK kinases. Different cyclins exhibit distinct expression and degradation patterns which contribute to the temporal coordination of each mitotic event. This cyclin forms a complex with and functions as a regulatory subunit of CDK4 or CDK6, whose activity is required for cell cycle G1/S transition. This protein has been shown to interact with tumor suppressor protein Rb and the expression of this gene is regulated positively by Rb. Mutations, amplification and overexpression of this gene, which alters cell cycle progression, are observed frequently in a variety of tumors and may contribute to tumorigenesis. [provided by RefSeq, Jul 2008].

**Size:** 50ul

**Concentration:** 1mg/ml

**Applications:** WB(1:500-2000)
IHC-P(1:100-500)
IHC-F(1:100-500)
Flow-Cyt(3μg/Test)
ICC(1:100)
IF(1:100-500)

**Cross Reactive Species:** Human
Mouse
Rat
.

For research use only. Not intended for diagnostic or therapeutic use.

## VALIDATION IMAGES

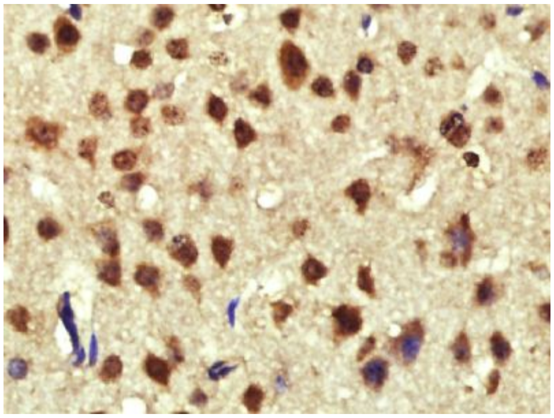

Paraformaldehyde-fixed, paraffin embedded (Mouse brain); Antigen retrieval by boiling in sodium citrate buffer (pH6.0) for 15min; Block endogenous peroxidase by 3% hydrogen peroxide for 20 minutes; Blocking buffer (normal goat serum) at 37°C for 30min; Antibody incubation with (Cyclin D1) Polyclonal Antibody, Unconjugated (bs-20596R) at 1:400 overnight at 4°C, followed by operating according to SP Kit(Rabbit) (sp-0023) instructionsand DAB staining.

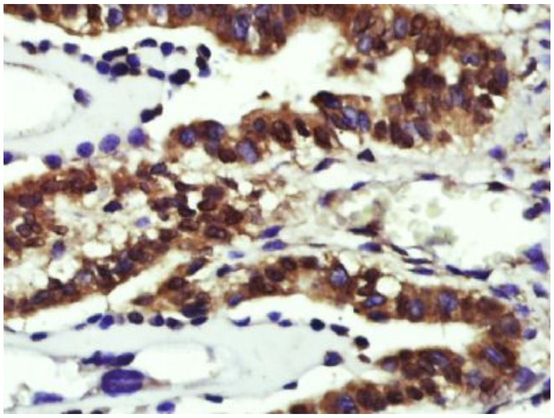

Paraformaldehyde-fixed, paraffin embedded (Mouse placenta); Antigen retrieval by boiling in sodium citrate buffer (pH6.0) for 15min; Block endogenous peroxidase by 3% hydrogen peroxide for 20 minutes; Blocking buffer (normal goat serum) at 37°C for 30min; Antibody incubation with (Cyclin D1) Polyclonal Antibody, Unconjugated (bs-20596R) at 1:400 overnight at 4°C, followed by operating according to SP Kit(Rabbit) (sp-0023) instructionsand DAB staining.

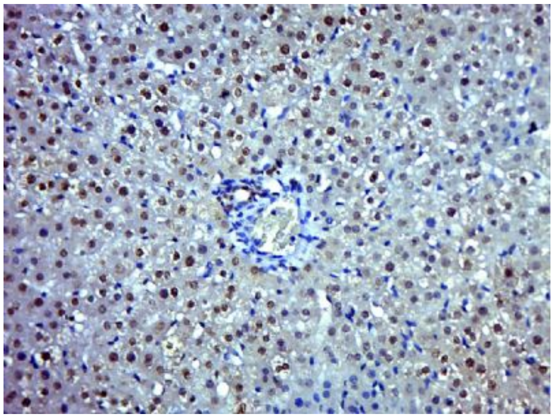

Paraformaldehyde-fixed, paraffin embedded (Rat liver); Antigen retrieval by boiling in sodium citrate buffer (pH6.0) for 15min; Block endogenous peroxidase by 3% hydrogen peroxide for 20 minutes; Blocking buffer (normal goat serum) at 37°C for 30min; Antibody incubation with (Cyclin D1) Polyclonal Antibody, Unconjugated (bs-20596R) at 1:500 overnight at 4°C, followed by a conjugated secondary (sp-0023) for 20 minutes and DAB staining.

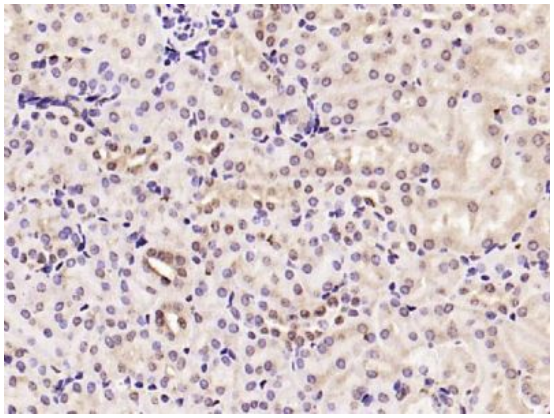

Paraformaldehyde-fixed, paraffin embedded (mouse kidney); Antigen retrieval by boiling in sodium citrate buffer (pH6.0) for 15min; Block endogenous peroxidase by 3% hydrogen peroxide for 20 minutes; Blocking buffer (normal goat serum) at 37°C for 30min; Antibody incubation with (Cyclin D1) Polyclonal Antibody, Unconjugated (bs-20596R) at 1:200 overnight at 4°C, followed by operating according to SP Kit(Rabbit) (sp-0023) instructionsand DAB staining.

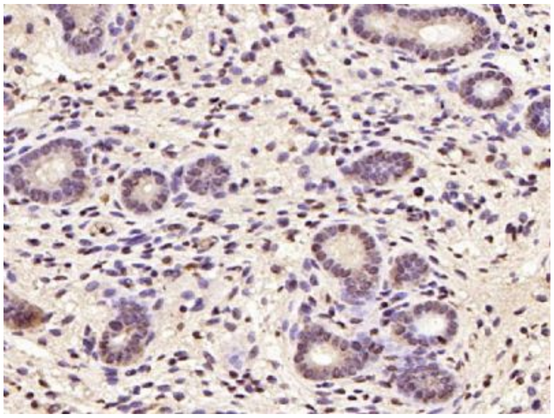

Paraformaldehyde-fixed, paraffin embedded (rat uterus); Antigen retrieval by boiling in sodium citrate buffer (pH6.0) for 15min; Block endogenous peroxidase by 3% hydrogen peroxide for 20 minutes; Blocking buffer (normal goat serum) at 37°C for 30min; Antibody incubation with (Cyclin D1) Polyclonal Antibody, Unconjugated (bs-20596R) at 1:200 overnight at 4°C, followed by operating according to SP Kit(Rabbit) (sp-0023) instructionsand DAB staining.

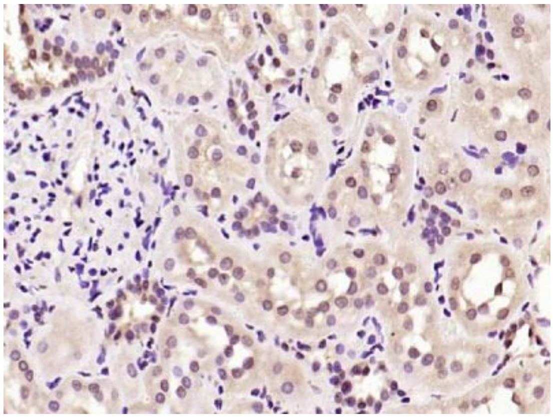

Paraformaldehyde-fixed, paraffin embedded (rat kidney); Antigen retrieval by boiling in sodium citrate buffer (pH6.0) for 15min; Block endogenous peroxidase by 3% hydrogen peroxide for 20 minutes; Blocking buffer (normal goat serum) at 37°C for 30min; Antibody incubation with (Cyclin D1) Polyclonal Antibody, Unconjugated (bs-20596R) at 1:200 overnight at 4°C, followed by operating according to SP Kit(Rabbit) (sp-0023) instructionsand DAB staining.

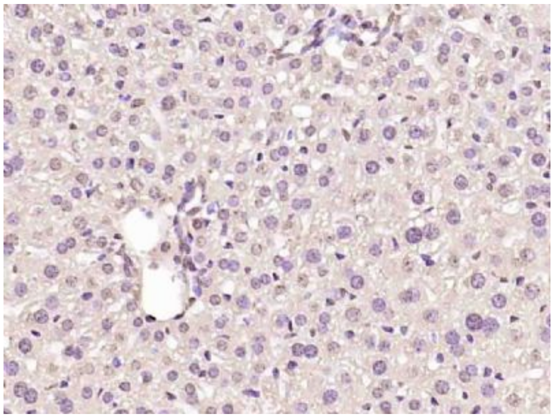

Paraformaldehyde-fixed, paraffin embedded (mouse liver); Antigen retrieval by boiling in sodium citrate buffer (pH6.0) for 15min; Block endogenous peroxidase by 3% hydrogen peroxide for 20 minutes; Blocking buffer (normal goat serum) at 37°C for 30min; Antibody incubation with (Cyclin D1) Polyclonal Antibody, Unconjugated (bs-20596R) at 1:200 overnight at 4°C, followed by operating according to SP Kit(Rabbit) (sp-0023) instructionsand DAB staining.

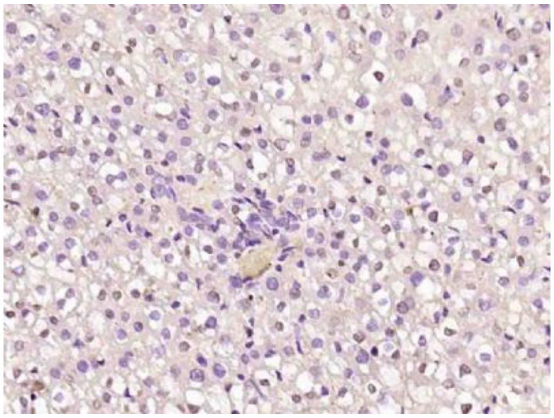

Paraformaldehyde-fixed, paraffin embedded (rat liver); Antigen retrieval by boiling in sodium citrate buffer (pH6.0) for 15min; Block endogenous peroxidase by 3% hydrogen peroxide for 20 minutes; Blocking buffer (normal goat serum) at 37°C for 30min; Antibody incubation with (Cyclin D1) Polyclonal Antibody, Unconjugated (bs-20596R) at 1:200 overnight at 4°C, followed by operating according to SP Kit(Rabbit) (sp-0023) instructionsand DAB staining.

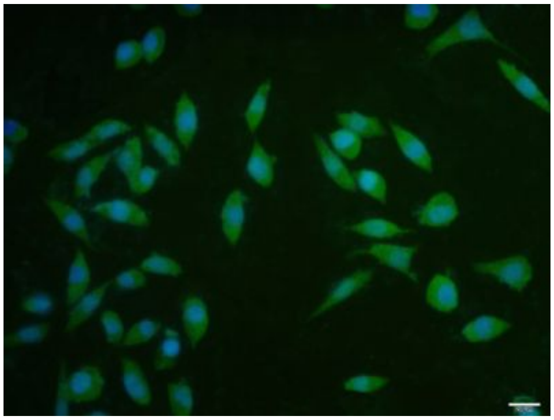

A431 cell; 4% Paraformaldehyde-fixed; Triton X-100 at room temperature for 20 min; Blocking buffer (normal goat serum, C-0005) at 37°C for 20 min; Antibody incubation with (Cyclin D1) polyclonal Antibody, Unconjugated (bs-20596R) 1:100, 90 minutes at 37°C; followed by a conjugated Goat Anti-Rabbit IgG antibody at 37°C for 90 minutes, DAPI (blue, C02-04002) was used to stain the cell nuclei.

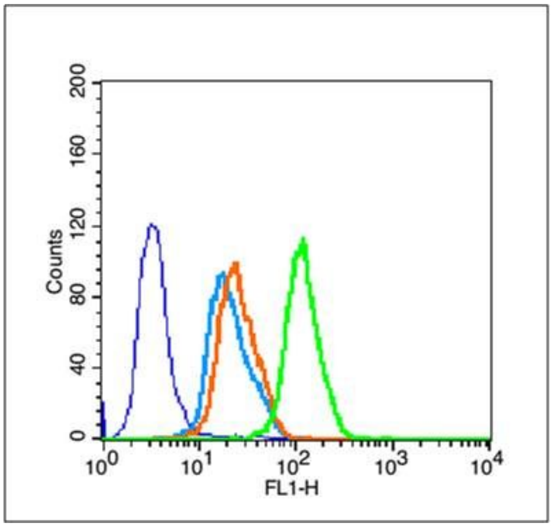


Blank control (blue line): MCF 7 (fixed with 70% methanol (Overnight at 4℃) and then permeabilized with 90% ice-cold methanol for 30 min on ice).
Primary Antibody (green line): Rabbit Anti-Cyclin D1 antibody (bs-20596R),Dilution: 3μg /10^5 cells;
Isotype Control Antibody (orange line): Rabbit IgG .
Secondary Antibody (white blue line): Goat anti-rabbit IgG-FITC,Dilution: 1μg /test.

## PRODUCT SPECIFIC PUBLICATIONS


- Juan Tan. et al. Circ_0124644 Serves as a ceRNA for miR-590-3p to Promote Hypoxia-Induced Cardiomyocytes Injury via Regulating SOX4. Front Genet. 2021; 12: 667724 Read more>>
- Jiancong Lu. et al. Circ_0020123 Increases ZFX Expression to Facilitate Non-Small Cell Lung Cancer Progression by Sponging miR-142-3p. Cancer Manag Res. 2021; 13: 1687–1698 Read more>>
- Yunqi Hua. et al. Circular RNA Circ_0006282 Promotes Cell Proliferation and Metastasis in Gastric Cancer by Regulating MicroRNA-144-5p/Tyrosine 3-Monooxygenase/Tryptophan 5-Monooxygenase Activation Protein β Axis. Cancer Manag Res. 2021; 13: 815–827 Read more>>
- Zhang Wei. et al. C1QTNF6 regulates cell proliferation and apoptosis of NSCLC in vitro and in vivo. Bioscience Rep. 2021 Jan;41(1):BSR20201541 Read more>>
- Zou P et al. Mechanisms of Stress-Induced Spermatogenesis Impairment in Male Rats Following Unpredictable Chronic Mild Stress (uCMS). Int. J. Mol. Sci. 2019, 20, 4470. Read more>>
- Jiang L et al. Astrocytes induce proliferation of oligodendrocyte progenitor cells via connexin 47-mediated activation of Chi3l1 expression. Eur Rev Med Pharmacol Sci. 2019 Apr;23(7):3012-3020. Read more>>

**bsm-33346M**

**[Primary Antibody]**

www.bioss.com.cn
sales@bioss.com.cn
techsupport@bioss.com.cn
400-901-9800

# Mouse Anti-Flag Tag Monoclonal Antibody

## DATASHEET

**Host:** Mouse

**Target Protein:** Flag Tag

**IR:** Immunogen Range:

**Clonality:** Monoclonal

**Isotype:** IgG

**Entrez Gene:** N/A

**Swiss Prot:** N/A

**Source:** KLH conjugated Flag Tag:

**Purification:** affinity purified by Protein G

**Storage:** 0.01M TBS(pH7.4) with 1% BSA, 0.03% Proclin300 and 50% Glycerol. Shipped at 4℃. Store at -20 °C for one year. Avoid repeated freeze/thaw cycles.

**Background:** This is a useful tool for the localisation and characterisation of DDDDK tagged proteins.

**Size:** 50ul

**Concentration:** 1mg/ml

**Applications:** WB(1:5000-20000)
ELISA(1:5000-10000)
IP(1:20-100)
IF(1:100-500)

**Cross Reactive Species:** Species independent.

For research use only. Not intended for diagnostic or therapeutic use.

## VALIDATION IMAGES

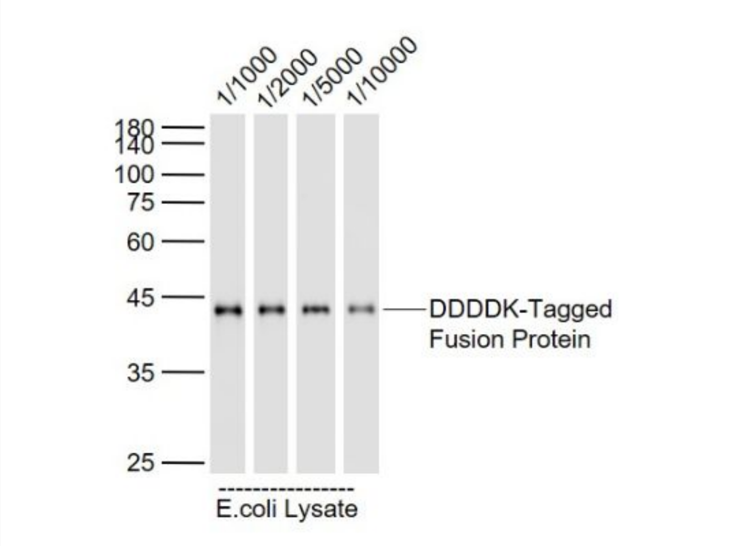


Sample:
DDDDK-Tagged Fusion Protein Overexpression E.coli Lysate (Cat#: bs-41230P) at 4 ug
Primary: Anti-Flag Tag (bsm-33346M) at 1/1000 ~ 1/10000 dilution
Secondary: IRDye800CW Goat Anti-Mouse IgG at 1/20000 dilution
Predicted band size: 41 kD
Observed band size: 41 kD

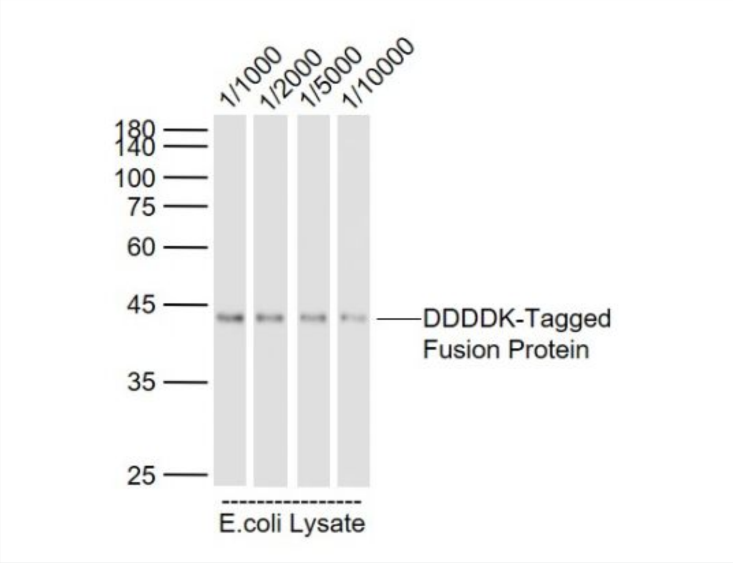


Sample:

DDDDK-Tagged Fusion Protein Overexpression E.coli Lysate (Cat#: bs-41230P) at 2 ug

Primary: Anti-Flag Tag (bsm-33346M) at 1/1000 ~ 1/10000 dilution

Secondary: IRDye800CW Goat Anti-Mouse IgG at 1/20000 dilution

Predicted band size: 41 kD

Observed band size: 41 kD

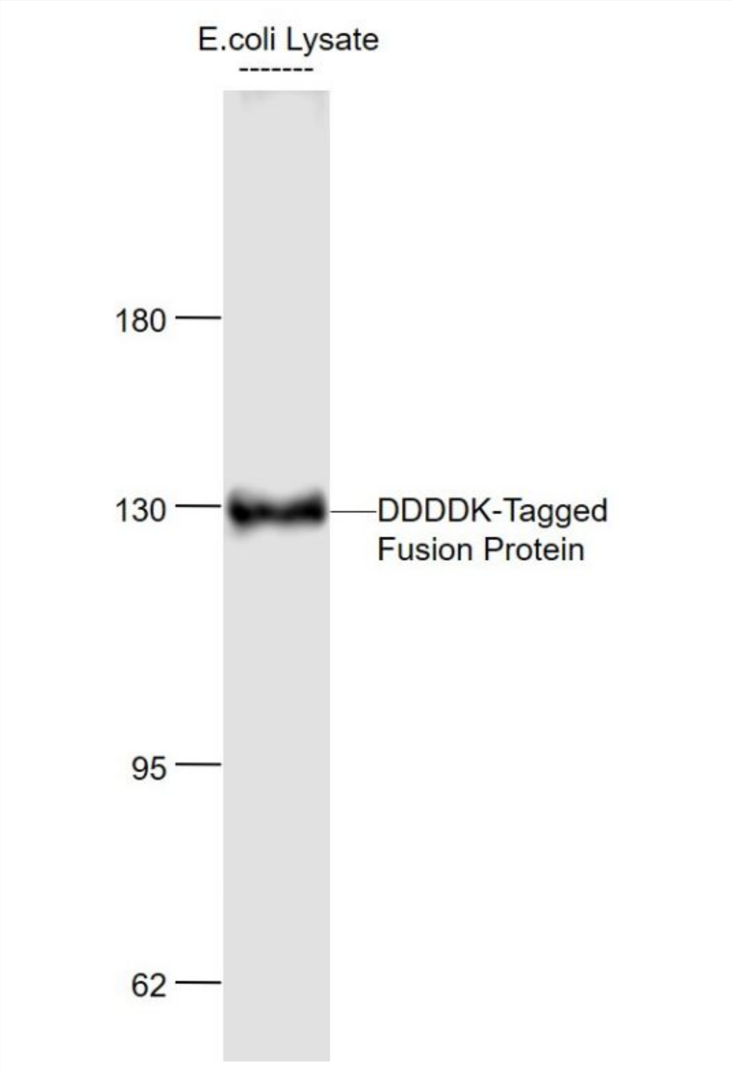


Sample:

Lane1: DDDDK-Tagged Fusion Protein Overexpression E.coli Lysate at 2ug

Primary: Anti-FLAG Tag (bsm-33346M) at 1/1000 dilution

Secondary: IRDye800CW Goat Anti-Mouse IgG at 1/20000 dilution

Predicted band size: 130 kD

Observed band size: 130 kD

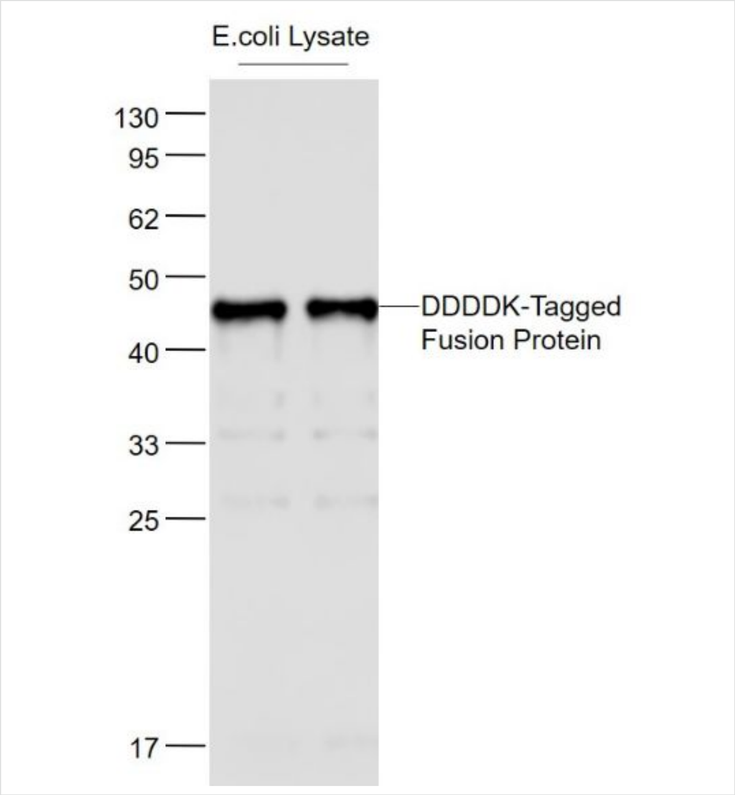


Sample:

Lane1: DDDDK-Tagged Fusion Protein Overexpression E.coli Lysate at 2ug

Lane2: DDDDK-Tagged Fusion Protein Overexpression E.coli Lysate at 2ug

Primary: Anti-Flag Tag (bsm-33346M) at 1/1000 dilution

Secondary: IRDye800CW Goat Anti-Mouse IgG at 1/20000 dilution

Predicted band size: 41 kD

Observed band size: 45 kD

## PRODUCT SPECIFIC PUBLICATIONS

**bsm-52026R**

**[Primary Antibody]**

www.bioss.com.cn
sales@bioss.com.cn
techsupport@bioss.com.cn
400-901-9800

# Rabbit Anti-CDK1 Monoclonal Antibody

## DATASHEET

**Host:** Rabbit

**Target Protein:** CDK1

**IR:** Immunogen Range:

**Clonality:** Monoclonal

**Isotype:** IgG

**Entrez Gene:** 983

**Swiss Prot:** P06493

**Source:** Recombinant human CDK1 protein, full length:

**Purification:** affinity purified by Protein A

**Storage:** 0.01M TBS(pH7.4) with 1% BSA, 0.03% Proclin300 and 50% Glycerol. Shipped at 4℃. Store at -20 °C for one year. Avoid repeated freeze/thaw cycles.

**Background:** The cell division control protein cdc2, also known as cyclin dependent kinase 1 (Cdk1) or p34/cdk1, plays a key role in the control of the eukaryotic cell cycle, where it is required for entry into S phase and mitosis. Cdc2 exists as a complex with both cyclin A and cyclin B. The best characterized of these associations is the Cdc2 p34 cyclin B complex, which is required for the G2 to M phase transition. Activation of Cdc2 is controlled at several steps including cyclin binding and phosphorylation of threonine 161. However, the critical regulatory step in activating cdc2 during progression into mitosis appears to be dephosphorylation of Tyr15 and Tyr14. Phosphorylation at Tyr15 and inhibition of Cdc2 is carried out by WEE1 and MIK protein kinases while Tyr15 dephosphorylation and activation of Cdc2 is carried out by the cdc25 phosphatase. The isoform CDC2deltaT is found in breast cancer tissues. Furthermore, cdc2/Cdk1 is a key mediator of neuronal cell death in brain development and degeneration.

**Size:** 50ul

**Concentration:** 1mg/ml

**Applications:** WB(1:500-2000)
IHC-P(1:50-200)
IHC-F(1:50-200)
Flow-Cyt(1:50)
ICC(1:50)
IF(1:50-200)

**Cross Reactive Species:** Human
Mouse
Rat
.

For research use only. Not intended for diagnostic or therapeutic use.

## VALIDATION IMAGES

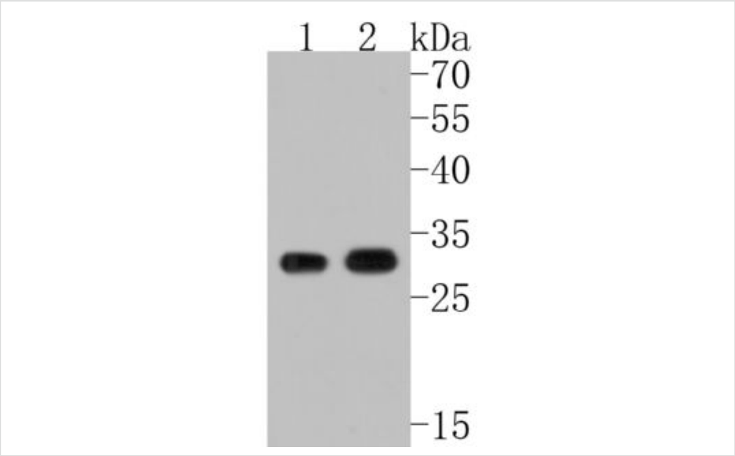


Sample:

Lane 1: HepG2 (Human) Cell Lysate at 30 ug

Lane 2: Jurkat (Human) Cell Lysate at 30 ug

Primary: Anti-CDK1 (bsm-52026R) at 1/500 dilution

Secondary: Goat Anti-Rabbit IgG - HRP at 1/5000 dilution

Predicted band size: 34 kD

Observed band size: 34 kD

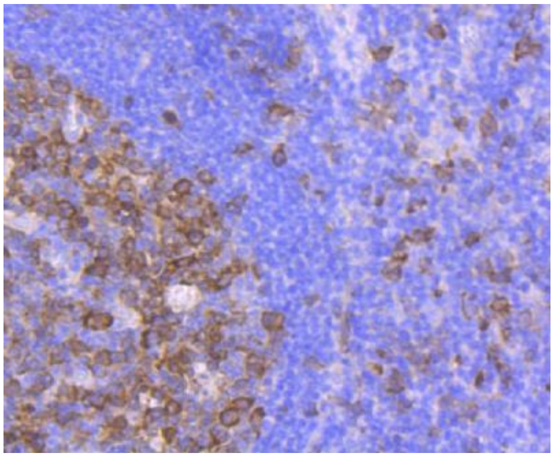

Paraformaldehyde-fixed, paraffin embedded (human tonsil); Antigen retrieval by boiling in sodium citrate buffer (pH6.0) for 15min; Block endogenous peroxidase by 3% hydrogen peroxide for 20 minutes; Blocking buffer (normal goat serum) at 37°C for 30min; Antibody incubation with (CDK1) Monoclonal Antibody, Unconjugated (bsm-52026R) at 1:50 overnight at 4°C, followed by operating according to SP Kit(Rabbit) (sp-0023) instructionsand DAB staining.

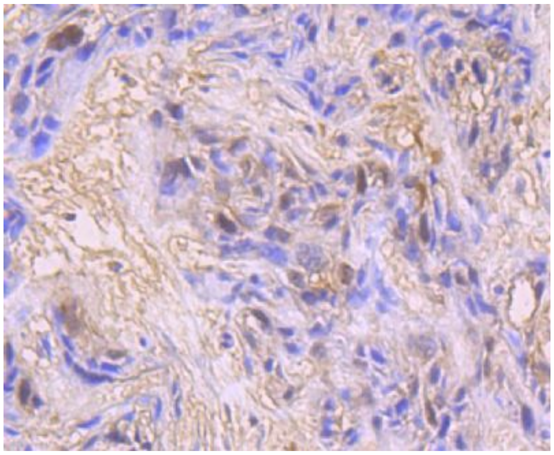

Paraformaldehyde-fixed, paraffin embedded (human breast carcinoma); Antigen retrieval by boiling in sodium citrate buffer (pH6.0) for 15min; Block endogenous peroxidase by 3% hydrogen peroxide for 20 minutes; Blocking buffer (normal goat serum) at 37°C for 30min; Antibody incubation with (CDK1) Monoclonal Antibody, Unconjugated (bsm-52026R) at 1:50 overnight at 4°C, followed by operating according to SP Kit(Rabbit) (sp-0023) instructionsand DAB staining.

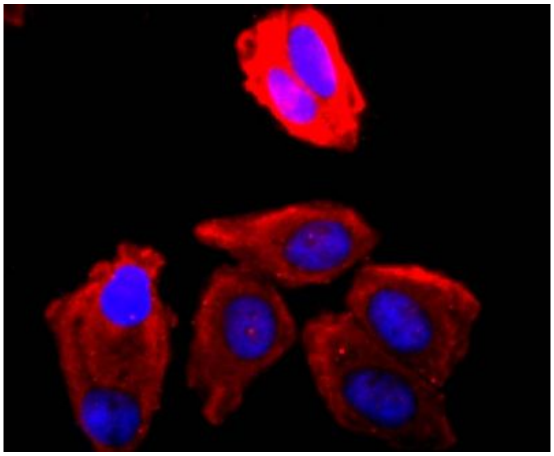

MCF-7 cell; 4% Paraformaldehyde-fixed; Triton X-100 at room temperature for 20 min; Blocking buffer (normal goat serum, C-0005) at 37°C for 20 min; Antibody incubation with (CDK1) monoclonal Antibody, Unconjugated (bsm-52026R) 1:50, 90 minutes at 37°C; followed by a conjugated Goat Anti-Rabbit IgG antibody at 37°C for 90 minutes, DAPI (blue, C02-04002) was used to stain the cell nuclei.

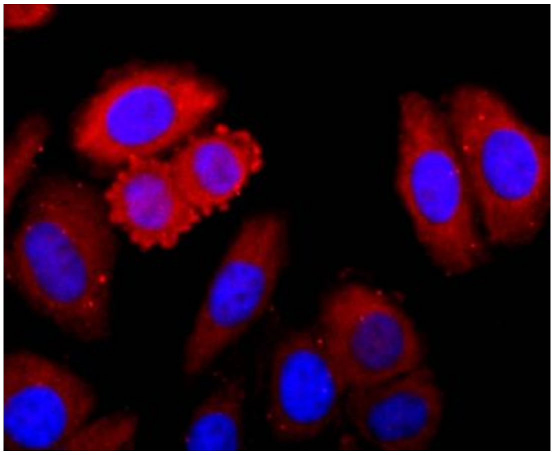

HepG2 cell; 4% Paraformaldehyde-fixed; Triton X-100 at room temperature for 20 min; Blocking buffer (normal goat serum, C-0005) at 37°C for 20 min; Antibody incubation with (CDK1) monoclonal Antibody, Unconjugated (bsm-52026R) 1:50, 90 minutes at 37°C; followed by a conjugated Goat Anti-Rabbit IgG antibody at 37°C for 90 minutes, DAPI (blue, C02-04002) was used to stain the cell nuclei.

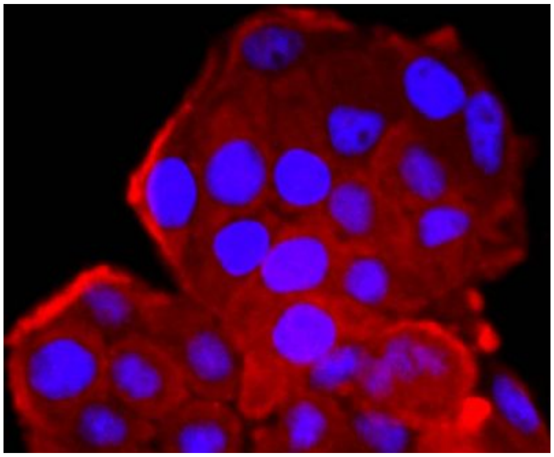

Hela cell; 4% Paraformaldehyde-fixed; Triton X-100 at room temperature for 20 min; Blocking buffer (normal goat serum, C-0005) at 37°C for 20 min; Antibody incubation with (CDK1) monoclonal Antibody, Unconjugated (bsm-52026R) 1:50, 90 minutes at 37°C; followed by a conjugated Goat Anti-Rabbit IgG antibody at 37°C for 90 minutes, DAPI (blue, C02-04002) was used to stain the cell nuclei.

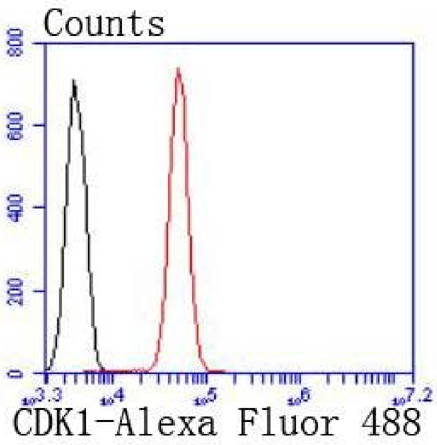


Blank control: Jurkat.

Primary Antibody (green line): Rabbit Anti-CDK1 antibody (bsm-52026R)

Dilution: 1:50;

Isotype Control Antibody (orange line): Rabbit IgG .

Secondary Antibody : Goat anti-rabbit IgG-AF488

Dilution: 1:1000.

Protocol

The cells were fixed with 4% PFA (10min at room temperature)and then permeabilized with 90% ice-cold methanol for 20 min at-20℃. The cells were then incubated in 5%BSA to block non-specific protein-protein interactions for 30 min at room temperature .Cells stained with Primary Antibody for 30 min at room temperature. The secondary antibody used for 40 min at room temperature. Acquisition of 20,000 events was performed.

## PRODUCT SPECIFIC PUBLICATIONS

**bsm-52028M** **[Primary Antibody]**

www.bioss.com.cn
sales@bioss.com.cn
techsupport@bioss.com.cn
400-901-9800

# Mouse Anti-Cdk4 Monoclonal Antibody

## DATASHEET

**Host:** Mouse

**Target Protein:** Cdk4

**IR:** Immunogen Range:

**Clonality:** Monoclonal

**Isotype:** IgG

**Entrez Gene:** 1019

**Swiss Prot:** P11802

**Source:** KLH conjugated synthetic peptide derived from human Cdk4:

**Purification:** affinity purified by Protein A

**Storage:** 0.01M TBS(pH7.4) with 1% BSA, 0.03% Proclin300 and 50% Glycerol. Shipped at 4℃. Store at -20 °C for one year. Avoid repeated freeze/thaw cycles.

**Background:** The protein encoded by this gene is a member of the Ser/Thr protein kinase family. This protein is highly similar to the gene products of S. cerevisiae cdc28 and S. pombe cdc2. It is a catalytic subunit of the protein kinase complex that is important for cell cycle G1 phase progression. The activity of this kinase is restricted to the G1-S phase, which is controlled by the regulatory subunits D-type cyclins and CDK inhibitor p16(INK4a). This kinase was shown to be responsible for the phosphorylation of retinoblastoma gene product (Rb). Mutations in this gene as well as in its related proteins including D-type cyclins, p16(INK4a) and Rb were all found to be associated with tumorigenesis of a variety of cancers. Multiple polyadenylation sites of this gene have been reported. [provided by RefSeq, Jul 2008]

**Size:** 50ul

**Concentration:** 1mg/ml

**Applications:** WB(1:500-1000)
IHC-P(1:50-200)
IHC-F(1:50-200)
ICC(1:50-200)
IF(1:50-200)

**Cross Reactive Species:** Human
Mouse
Rat
.

For research use only. Not intended for diagnostic or therapeutic use.

## VALIDATION IMAGES

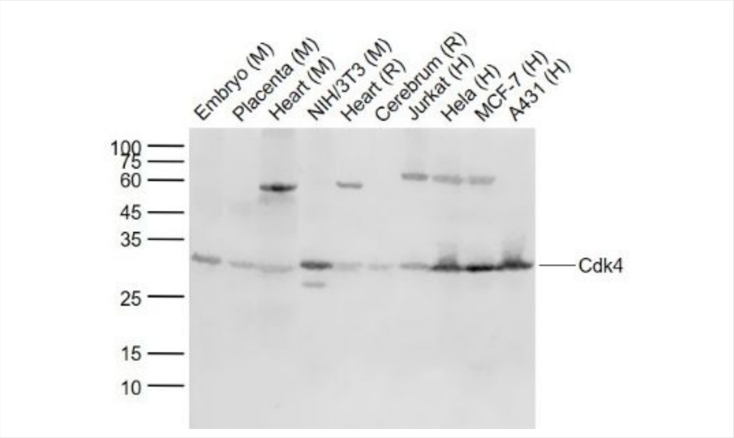


Sample:

Lane 1: Embryo (Mouse) Lysate at 40 ug

Lane 2: Placenta (Mouse) Lysate at 40 ug

Lane 3: Heart (Mouse) Lysate at 40 ug

Lane 4: NIH/3T3 (Mouse) Cell Lysate at 30 ug

Lane 5: Heart (Rat) Lysate at 40 ug

Lane 6: Cerebrum (Rat) Lysate at 40 ug

Lane 7: Jurkat (Human) Cell Lysate at 30 ug

Lane 8: Hela (Human) Cell Lysate at 30 ug

Lane 9: MCF-7 (Human) Cell Lysate at 30 ug

Lane 10: A431 (Human) Cell Lysate at 30 ug

Primary:

Anti-Cdk4 (bsm-52028M) at 1/1000 dilution

Secondary: IRDye800CW Goat Anti-Mouse IgG at 1/20000 dilution

Predicted band size: 34 kD

Observed band size: 32 kD

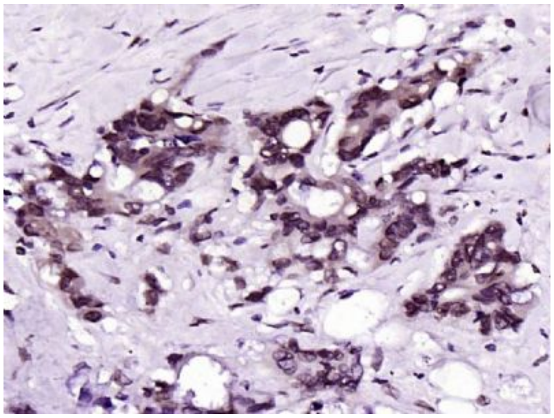

Paraformaldehyde-fixed, paraffin embedded (human rectal carcinoma); Antigen retrieval by boiling in sodium citrate buffer (pH6.0) for 15min; Block endogenous peroxidase by 3% hydrogen peroxide for 20 minutes; Blocking buffer (normal goat serum) at 37°C for 30min; Antibody incubation with (Cdk4) Monoclonal Antibody, Unconjugated (bsm-52028M) at 1:200 overnight at 4°C, followed by operating according to SP Kit(Mouse)(sp-0024) instructionsand DAB staining.

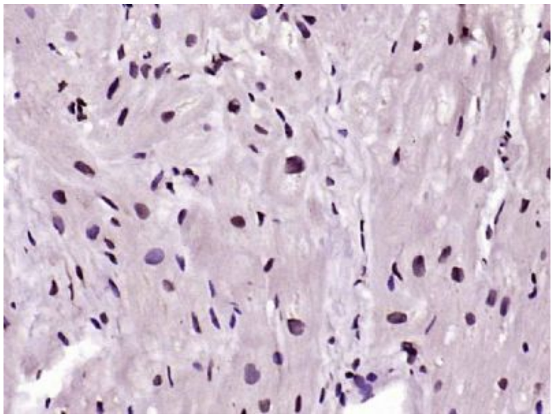

Paraformaldehyde-fixed, paraffin embedded (human myocardium); Antigen retrieval by boiling in sodium citrate buffer (pH6.0) for 15min; Block endogenous peroxidase by 3% hydrogen peroxide for 20 minutes; Blocking buffer (normal goat serum) at 37°C for 30min; Antibody incubation with (Cdk4) Monoclonal Antibody, Unconjugated (bsm-52028M) at 1:200 overnight at 4°C, followed by operating according to SP Kit(Mouse)(sp-0024) instructionsand DAB staining.

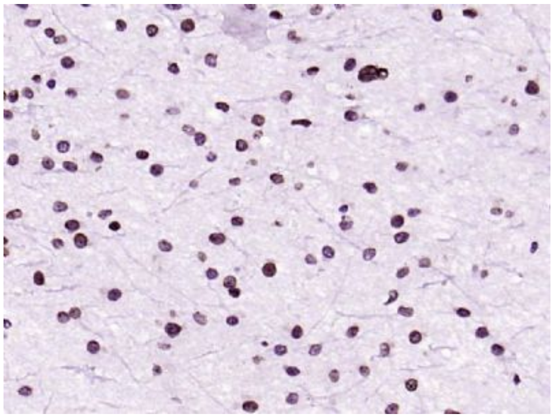

Paraformaldehyde-fixed, paraffin embedded (human brain); Antigen retrieval by boiling in sodium citrate buffer (pH6.0) for 15min; Block endogenous peroxidase by 3% hydrogen peroxide for 20 minutes; Blocking buffer (normal goat serum) at 37°C for 30min; Antibody incubation with (Cdk4) Monoclonal Antibody, Unconjugated (bsm-52028M) at 1:200 overnight at 4°C, followed by operating according to SP Kit(Mouse)(sp-0024) instructionsand DAB staining.

## PRODUCT SPECIFIC PUBLICATIONS


- Que, Tianshi. et al. HMGA1 stimulates MYH9-dependent ubiquitination of GSK-3β via PI3K/Akt/c-Jun signaling to promote malignant progression and chemoresistance in gliomas. Cell Death Dis. 2021 Dec;12(12):1-12 Read more>>

# bsm-52044R

**[Primary Antibody]**

Bioss ANTIBODIES
www.bioss.com.cn
sales@bioss.com.cn
techsupport@bioss.com.cn
400-901-9800

## Rabbit Anti-Cyclin B1 Monoclonal Antibody

DATASHEET

**Host:** Rabbit

**Target Protein:** Cyclin B1

**IR:** Immunogen Range:

**Clonality:** Monoclonal

**Isotype:** IgG

**Entrez Gene:** 891

**Swiss Prot:** P24860

**Source:** Recombinant human Cyclin B1 protein（1-150aa）：

**Purification:** affinity purified by Protein A

**Storage:** 0.01M TBS(pH7.4) with 1% BSA, 0.03% Proclin300 and 50% Glycerol. Shipped at 4℃. Store at -20 °C for one year. Avoid repeated freeze/thaw cycles.

**Background:** The protein encoded by this gene is a regulatory protein involved in mitosis. The gene product complexes with p34(cdc2) to form the maturation-promoting factor (MPF). Two alternative transcripts have been found, a constitutively expressed transcript and a cell cycle-regulated transcript, that is expressed predominantly during G2/M phase. The different transcripts result from the use of alternate transcription initiation sites. [provided by RefSeq, Jul 2008].

**Size:** 50ul

**Concentration:** 1mg/ml

**Applications:** WB(1:500-2000)
ELISA(1:5000-10000)
IHC-P(1:50-200)
IHC-F(1:50-200)
ICC(1:50-200)
IF(1:50-200)

**Cross Reactive Species:** Human
Mouse
.

For research use only. Not intended for diagnostic or therapeutic use.

## VALIDATION IMAGES

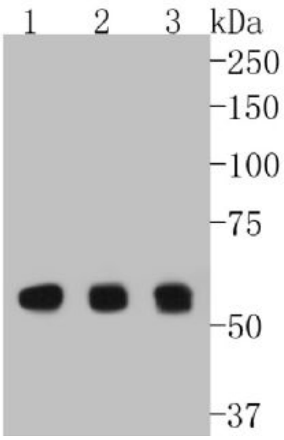


Sample:

Lane 1: Hela cell lysate

Lane 2: Daudi cell lysate

Lane 3: K562 cell lysate

Primary: Anti-Cyclin B1 (bsm-52044R) at 1/500 dilution

Secondary: Goat Anti-Rabbit IgG - HRP at 1/5000 dilution

Predicted band size: 48 kD

Observed band size: 55 kD

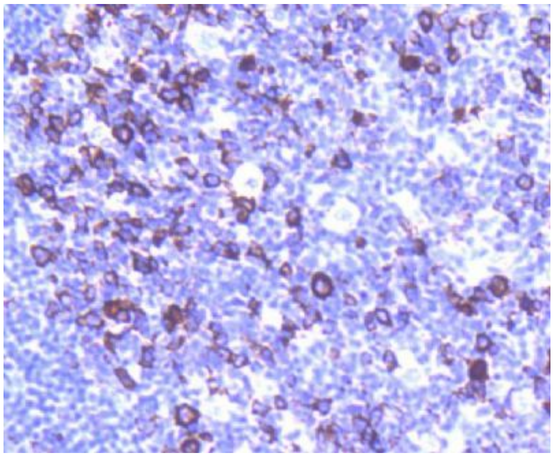

Paraformaldehyde-fixed, paraffin embedded (human tonsil); Antigen retrieval by boiling in sodium citrate buffer (pH6.0) for 15min; Block endogenous peroxidase by 3% hydrogen peroxide for 20 minutes; Blocking buffer (normal goat serum) at 37°C for 30min; Antibody incubation with (Cyclin B1) Monoclonal Antibody, Unconjugated (bsm-52044R) at 1:50 overnight at 4°C, followed by operating according to SP Kit(Rabbit) (sp-0023) instructionsand DAB staining.

## PRODUCT SPECIFIC PUBLICATIONS

abcam

# Product datasheet

# Anti-Histone H3 antibody - Nuclear Marker and ChIP Grade ab1791

★★★★★ 232 Abreviews 3819 References 14 图像

## 概述

| | |
|---|---|
| 产品名称 | Anti-Histone H3抗体-核Marker and ChIP Grade |
| 描述 | 兔多克隆抗体to Histone H3 -核Marker and ChIP Grade |
| 宿主 | Rabbit |
| 特异性 | Based only on sequence homology, we expect the antibody to react with multiple variants of H3 such as H3.1, H3.2 and H3.3. |
| 经测试应用 | **适用于:** ICC, IHC-P, ChIP, IP, WB |
| 种属反应性 | **与反应:** Mouse, Rat, Human, Saccharomyces cerevisiae, Xenopus laevis, Arabidopsis thaliana, Drosophila melanogaster, Indian muntjac, Schizosaccharomyces pombe<br>**预测可用于:** Chicken, Dog, Caenorhabditis elegans, Ferret, Zebrafish, a wide range of other species, Mammals, Silk worm, Dictyostelium discoideum, Rainbow trout, Neurospora crassa, Toxoplasma gondii, Rice, Schistosoma mansoni, Candida albicans, Cyanidioschyzon merolae |
| 免疫原 | Synthetic peptide. This information is proprietary to Abcam and/or its suppliers.<br>(Peptide available as ab12149) |
| 常规说明 | A recombinant rabbit monoclonal alternative is available to this target – ab176842<br>Rabbit polyclonal IgG (ab171870) is suitable for use as an isotype control with this antibody.<br>The Life Science industry has been in the grips of a reproducibility crisis for a number of years. Abcam is leading the way in addressing this with our range of recombinant monoclonal antibodies and knockout edited cell lines for gold-standard validation. Please check that this product meets your needs before purchasing.<br>If you have any questions, special requirements or concerns, please send us an inquiry and/or contact our Support team ahead of purchase. Recommended alternatives for this product can be found below, along with publications, customer reviews and Q&As |

## 性能

| | |
|---|---|
| 形式 | Liquid |

| | |
|---|---|
| **存放说明** | Shipped at 4°C. Store at +4°C short term (1-2 weeks). Upon delivery aliquot. Store at -20°C or -80°C. Avoid freeze / thaw cycle. |
| **存储溶液** | Preservative: 0.02% Sodium azide<br>Constituents: PBS, 40% Glycerol (glycerin, glycerine), 0.05% BSA<br><br>Batches of this product that have a concentration < 1mg/ml may have BSA added as a stabilising agent. If you would like information about the formulation of a specific lot, please contact our scientific support team who will be happy to help. |
| **纯度** | Immunogen affinity purified |
| **克隆** | 多克隆 |
| **同种型** | IgG |

## 应用

**The Abpromise guarantee** Abpromise™承诺保证使用ab1791于以下的经测试应用

"应用说明"部分 下显示的仅为推荐的起始稀释度；实际最佳的稀释度/浓度应由使用者检定。

| 应用 | Ab评论 | 说明 |
|---|---|---|
| ICC | ★★★★★ (4) | Use a concentration of 1 µg/ml. |
| IHC-P | ★★★★★ (20) | 1/100 - 1/400. Perform heat mediated antigen retrieval before commencing with IHC staining protocol. |
| ChIP | ★★★★★ (46) | Use 2µg for $10^6$ cells. |
| IP | ★★★★★ (4) | Use a concentration of 5 µg/ml. |
| WB | ★★★★★ (121) | 1/1000 - 1/5000. Detects a band of approximately 17 kDa (predicted molecular weight: 15 kDa).Can be blocked with Human Histone H3 peptide (ab12149).<br>We recommend Goat Anti-Rabbit IgG H&L (HRP) (ab6721) secondary antibody. |

## 靶标

| | |
|---|---|
| **功能** | Core component of nucleosome. Nucleosomes wrap and compact DNA into chromatin, limiting DNA accessibility to the cellular machineries which require DNA as a template. Histones thereby play a central role in transcription regulation, DNA repair, DNA replication and chromosomal stability. DNA accessibility is regulated via a complex set of post-translational modifications of histones, also called histone code, and nucleosome remodeling. |
| **序列相似性** | Belongs to the histone H3 family. |
| **发展阶段** | Expressed during S phase, then expression strongly decreases as cell division slows down during the process of differentiation. |
| **翻译后修饰** | Acetylation is generally linked to gene activation. Acetylation on Lys-10 (H3K9ac) impairs methylation at Arg-9 (H3R8me2s). Acetylation on Lys-19 (H3K18ac) and Lys-24 (H3K24ac) favors methylation at Arg-18 (H3R17me).<br>Citrullination at Arg-9 (H3R8ci) and/or Arg-18 (H3R17ci) by PADI4 impairs methylation and |

repesses transcription.
Asymmetric dimethylation at Arg-18 (H3R17me2a) by CARM1 is linked to gene activation.
Symmetric dimethylation at Arg-9 (H3R8me2s) by PRMT5 is linked to gene repression.
Asymmetric dimethylation at Arg-3 (H3R2me2a) by PRMT6 is linked to gene repression and is mutually exclusive with H3 Lys-5 methylation (H3K4me2 and H3K4me3). H3R2me2a is present at the 3' of genes regardless of their transcription state and is enriched on inactive promoters, while it is absent on active promoters.
Methylation at Lys-5 (H3K4me), Lys-37 (H3K36me) and Lys-80 (H3K79me) are linked to gene activation. Methylation at Lys-5 (H3K4me) facilitates subsequent acetylation of H3 and H4. Methylation at Lys-80 (H3K79me) is associated with DNA double-strand break (DSB) responses and is a specific target for TP53BP1. Methylation at Lys-10 (H3K9me) and Lys-28 (H3K27me) are linked to gene repression. Methylation at Lys-10 (H3K9me) is a specific target for HP1 proteins (CBX1, CBX3 and CBX5) and prevents subsequent phosphorylation at Ser-11 (H3S10ph) and acetylation of H3 and H4. Methylation at Lys-5 (H3K4me) and Lys-80 (H3K79me) require preliminary monoubiquitination of H2B at 'Lys-120'. Methylation at Lys-10 (H3K9me) and Lys-28 (H3K27me) are enriched in inactive X chromosome chromatin.
Phosphorylated at Thr-4 (H3T3ph) by GSG2/haspin during prophase and dephosphorylated during anaphase. Phosphorylation at Ser-11 (H3S10ph) by AURKB is crucial for chromosome condensation and cell-cycle progression during mitosis and meiosis. In addition phosphorylation at Ser-11 (H3S10ph) by RPS6KA4 and RPS6KA5 is important during interphase because it enables the transcription of genes following external stimulation, like mitogens, stress, growth factors or UV irradiation and result in the activation of genes, such as c-fos and c-jun. Phosphorylation at Ser-11 (H3S10ph), which is linked to gene activation, prevents methylation at Lys-10 (H3K9me) but facilitates acetylation of H3 and H4. Phosphorylation at Ser-11 (H3S10ph) by AURKB mediates the dissociation of HP1 proteins (CBX1, CBX3 and CBX5) from heterochromatin. Phosphorylation at Ser-11 (H3S10ph) is also an essential regulatory mechanism for neoplastic cell transformation. Phosphorylated at Ser-29 (H3S28ph) by MLTK isoform 1, RPS6KA5 or AURKB during mitosis or upon ultraviolet B irradiation. Phosphorylation at Thr-7 (H3T6ph) by PRKCBB is a specific tag for epigenetic transcriptional activation that prevents demethylation of Lys-5 (H3K4me) by LSD1/KDM1A. At centromeres, specifically phosphorylated at Thr-12 (H3T11ph) from prophase to early anaphase, by DAPK3 and PKN1. Phosphorylation at Thr-12 (H3T11ph) by PKN1 is a specific tag for epigenetic transcriptional activation that promotes demethylation of Lys-10 (H3K9me) by KDM4C/JMJD2C.
Phosphorylation at Tyr-42 (H3Y41ph) by JAK2 promotes exclusion of CBX5 (HP1 alpha) from chromatin.
Monoubiquitinated by RAG1 in lymphoid cells, monoubiquitination is required for V(D)J recombination (By similarity). Ubiquitinated by the CUL4-DDB-RBX1 complex in response to ultraviolet irradiation. This may weaken the interaction between histones and DNA and facilitate DNA accessibility to repair proteins.

**细胞定位** Nucleus. Chromosome.

## 图片

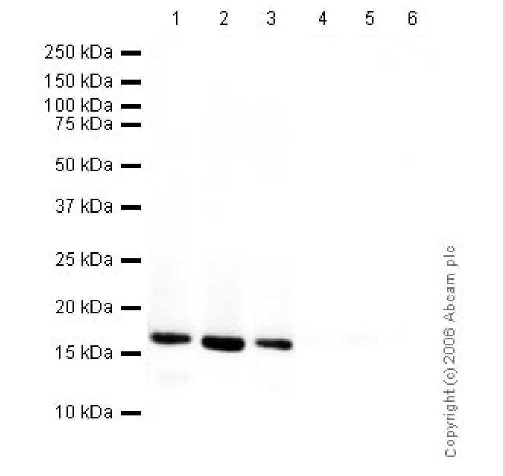


Western blot - Anti-Histone H3 antibody - Nuclear Marker and ChIP Grade (ab1791)

**All lanes :** Anti-Histone H3 antibody - Nuclear Marker and ChIP Grade (ab1791) at 1/1000 dilution

**Lane 1 :** A431 (Human epithelial carcinoma cell line) Whole Cell Lysate
**Lane 2 :** Jurkat (Human T cell lymphoblast-like cell line) Whole Cell Lysate
**Lane 3 :** HEK293 (Human embryonic kidney cell line) Whole Cell Lysate
**Lane 4 :** A431 (Human epithelial carcinoma cell line) Whole Cell Lysate with Human Histone H3 peptide (ab12149) at 1 µg/ml
**Lane 5 :** Jurkat (Human T cell lymphoblast-like cell line) Whole Cell Lysate with Human Histone H3 peptide (ab12149) at 1 µg/ml
**Lane 6 :** HEK293 (Human embryonic kidney cell line) Whole Cell Lysate with Human Histone H3 peptide (ab12149) at 1 µg/ml

Lysates/proteins at 20 µg per lane.

**Secondary**
**All lanes :** Goat Anti-Rabbit IgG H&L (HRP) (ab6721) at 1/5000 dilution

Developed using the ECL technique.

Performed under reducing conditions.

**Predicted band size:** 15 kDa
**Observed band size:** 17 kDa

**Exposure time:** 10 seconds

This blot was produced using a 4-12% Bis-tris gel under the MES buffer system. The gel was run at 200V for 35 minutes before being transferred onto a Nitrocellulose membrane at 30V for 70 minutes. The membrane was then blocked for an hour using 2% Bovine Serum Albumin before being incubated with ab1791 overnight at 4°C.

Goat Anti-Rabbit IgG H&L (HRP) (ab6721) secondary antibody was used for detection.

Antibody binding was visualised using ECL development solution ab133406.

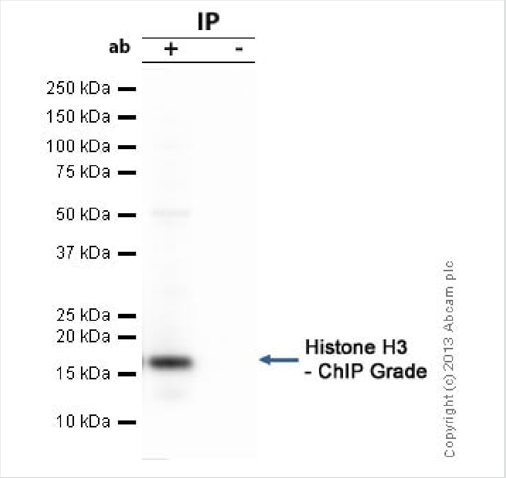


Immunoprecipitation - Anti-Histone H3 antibody - Nuclear Loading Control and ChIP Grade (ab1791)

Histone H3 - ChIP Grade was immunoprecipitated using 0.5mg HeLa (Human epithelial cell line from cervix adenocarcinoma) whole cell extract, 5 µg of Rabbit polyclonal to and 50 µl of protein G magnetic beads (+). No antibody was added to the control (-).

The antibody was incubated under agitation with Protein G beads for 10 minutes, HeLa whole cell extract lysate diluted in RIPA buffer was added to each sample and incubated for a further 10 minutes under agitation.

Proteins were eluted by addition of 40 µl SDS loading buffer and incubated for 10 minutes at 70°C; 10 µl of each sample was separated on a SDS PAGE gel, transferred to a nitrocellulose membrane, blocked with 5% BSA and probed with ab1791.

Secondary Antibody: Mouse anti-rabbit HRP light chain (HRP) (ab99697).

Band: 15kDa; Histone H3 - ChIP Grade

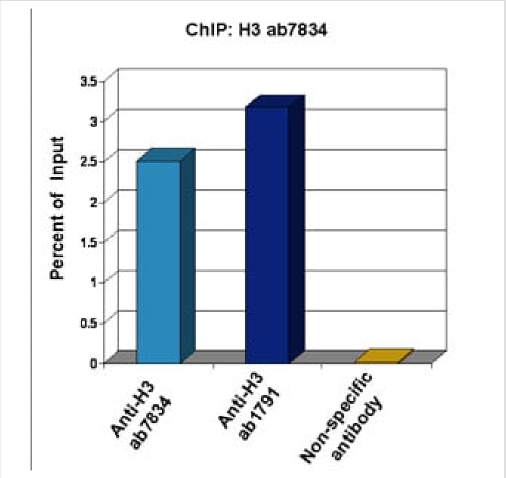


ChIP - Anti-Histone H3 antibody - Nuclear Loading Control and ChIP Grade (ab1791)

Chromatin from *Xenopus laevis* oocytes was prepared according to the Abcam X-ChIP protocol.

Oocytes were fixed with formaldehyde for 10 minutes. The ChIP was performed with 25 mg of chromatin, 3 mg of ab7834 (anti-H3, light blue) and 3 µg of ab1791 (anti-H3, dark blue), and 20 ml of Protein A/G sepharose beads. A non-specific antibody was used as a control (yellow).

The immunoprecipitated DNA was quantified by real time PCR (Taqman approach).

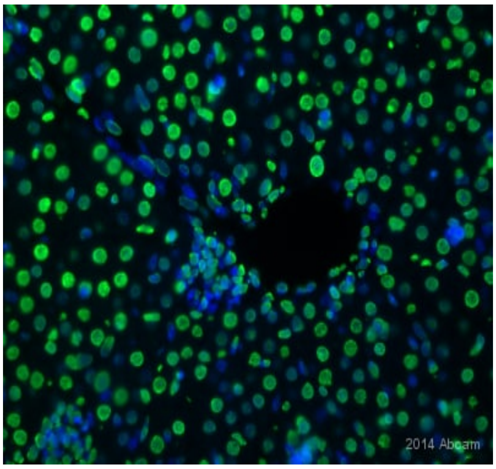


Immunohistochemistry (Formalin/PFA-fixed paraffin-embedded sections) - Anti-Histone H3 antibody - Nuclear Loading Control and ChIP Grade (ab1791)

This image is courtesy of an anonymous Abreview

ab1796 staining Histone H3 in mouse liver tissue sections by Immunohistochemistry (IHC-P - paraformaldehyde-fixed, paraffin-embedded sections).

Tissue was fixed with paraformaldehyde, permeabilized with 0.05% Triton X-100 in PBS for 30 minutes and blocked with 5% BSA for 1 hour; antigen retrieval was by heat mediation in sodium citrate pH 6. Samples were incubated with the primary antibody (1/500 in blocking buffer) for 16 hours at 4°C. An Alexa Fluor® 488-conjugated goat anti-rabbit IgG polyclonal (1/400) was used as the secondary antibody.

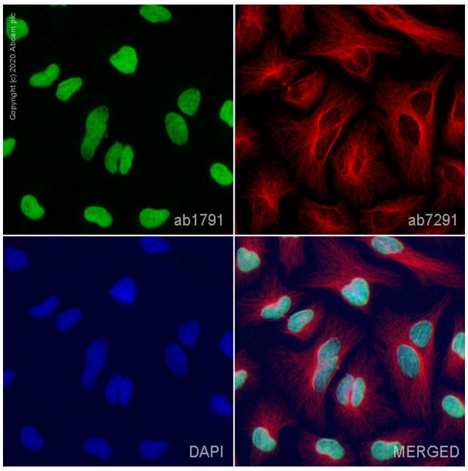


Immunocytochemistry - Anti-Histone H3 antibody - Nuclear Marker and ChIP Grade (ab1791)

ab1791 staining Histone H3 in HeLa cells. The cells were fixed with 100% methanol (5 min), permeabilized with 0.1% PBS-Triton X-100 for 5 minutes and then blocked with 1% BSA/10% normal goat serum/0.3M glycine in 0.1% PBS-Tween for 1h. The cells were then incubated overnight at 4°C with ab1791 at 0.1 µg/mL and ab7291, Mouse monoclonal [DM1A] to alpha Tubulin - Loading Control. Cells were then incubated with ab150081, Goat polyclonal Secondary Antibody to Rabbit IgG - H&L (Alexa Fluor® 488), pre-adsorbed at 1/1000 dilution (shown in green) and ab150120, Goat polyclonal Secondary Antibody to Mouse IgG - H&L (Alexa Fluor® 594), pre-adsorbed at 1/1000 dilution (shown in pseudocolour red). Nuclear DNA was labelled with DAPI (shown in blue).

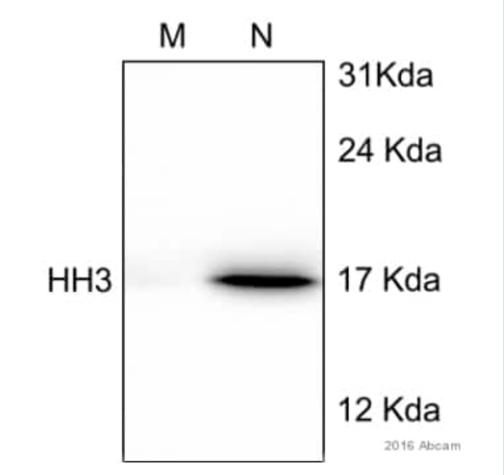


Western blot - Anti-Histone H3 antibody - Nuclear Loading Control and ChIP Grade (ab1791)

This image is courtesy of an anonymous Abreview

**All lanes :** Anti-Histone H3 antibody - Nuclear Marker and ChIP Grade (ab1791) at 1/1000 dilution

**Lane 1 :** Mouse skeletal muscle mitochondrial fraction
**Lane 2 :** Mouse skeletal muscle nuclear fraction

Lysates/proteins at 20 µg per lane.

**Secondary**
**All lanes :** HRP-conjugated goat anti-rabbit IgG at 1/4000 dilution

Developed using the ECL technique.

Performed under reducing conditions.

**Predicted band size:** 15 kDa
**Observed band size:** 17 kDa

**Exposure time:** 7 minutes

Blocked with 3% milk for 1 hour at 25°C.

Incubated with the primary antibody for 16 hours at 4°C in 3% milk in TBS-tween.

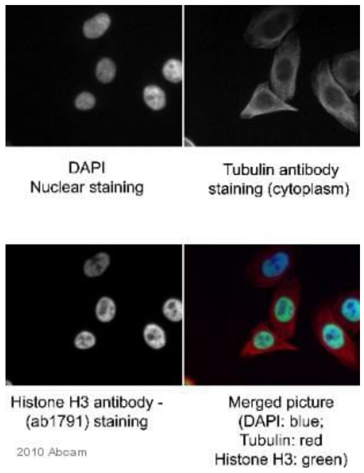


Immunocytochemistry - Anti-Histone H3 antibody - Nuclear Marker and ChIP Grade (ab1791)

This image is courtesy of an anonymous Abreview

ab1791 staining Histone H3 in HeLa (Human epithelial cell line from cervix adenocarcinoma) by ICC/IF (Immunocytochemistry/immunofluorescence).

Cells were fixed with methanol and blocked with 0.2% fish scale gelatin for 1 hour at 25°C. Samples were incubated with the primary antibody (1/300 in PBS + 0.2% gelatin) for 20 minutes at 25°C. An Alexa Fluor® 488-conjugated donkey anti-rabbit IgG polyclonal (1/500) was used as the secondary antibody.

Green - Histone H3.

Blue - DAPI.

Red - Tubulin.

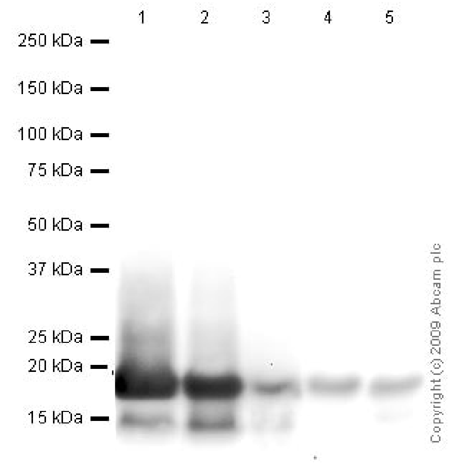


Western blot - Anti-Histone H3 antibody - Nuclear Loading Control and ChIP Grade (ab1791)

**All lanes :** Anti-Histone H3 antibody - Nuclear Marker and ChIP Grade (ab1791) at 1 µg/ml

**Lane 1 :** HeLa (Human epithelial carcinoma cell line) Whole Cell Lysate

**Lane 2 :** NIH/3T3 whole cell lysate (ab7179)

**Lane 3 :** Drosophila embryo nuclear extract (from melanogaster embryos 0-12Hr)

**Lane 4 :** S.cerevisiae (Y190) Whole Cell Lysate

**Lane 5 :** S.pombe Whole Cell Lysate

Lysates/proteins at 10 µg per lane.

**Secondary**

**All lanes :** Goat polyclonal to Rabbit IgG - H&L - Pre-Adsorbed (HRP) at 1/3000 dilution

Performed under reducing conditions.

**Predicted band size:** 15 kDa

**Observed band size:** 17 kDa

ab1791 is tested in western blot on a range of species.  We recommend loading higher amounts of protein (20-30ug) to increase the signal in yeast lysates

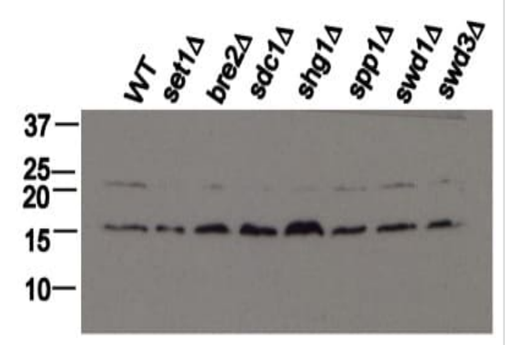


Western blot - Anti-Histone H3 antibody - Nuclear Loading Control and ChIP Grade (ab1791)

This image is courtesy of John E. Mueller and J. Ruth German (Mary Bryk lab)

Rabbit polyclonal to Histone H3 (ab1791) at 1/5000 on S. cerevisiae whole cell lysate (40 ug per lane).

Protein resolved on 15% SDS-PAGE gel. After transfer to PVDF membrane, blots were blocked in 1X PBS, 0.1% Tween-20, and 5% milk. ab1791 was diluted in 5 ml blocking buffer at 1/5000. Blots plus primary antibodies were either incubated overnight at 4°C or at RT for 2 hours. Blots were washed 6X for 10 minutes each in PBS with 0.1% Tween-20 before addition of secondary antibodies. Secondary antibodies were diluted 1/2,000 in blocking buffer and incubated with blots for 2 hours at RT. Secondary blots were washed 4X for 10 minutes each in PBS with 0.1% Tween-20 and 2X for 10 minutes each in PBS.

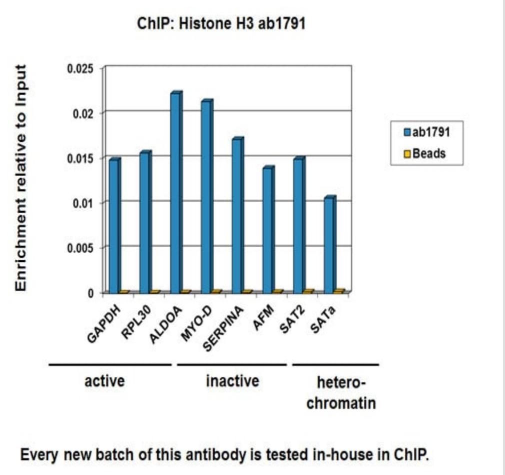


ChIP - Anti-Histone H3 antibody - Nuclear Loading Control and ChIP Grade (ab1791)

Chromatin was prepared from HeLa (Human epithelial cell line from cervix adenocarcinoma) cells according to the Abcam X-ChIP protocol.

Cells were fixed with formaldehyde for 10 minutes. The ChIP was performed with 25 µg of chromatin, 2 µg of ab1791 (blue), and 20 µl of Protein A/G sepharose beads. No antibody was added to the beads control (yellow).

The immunoprecipitated DNA was quantified by real time PCR (Taqman approach for active and inactive loci, Sybr green approach for heterochromatic loci). Primers and probes are located in the first kb of the transcribed region.

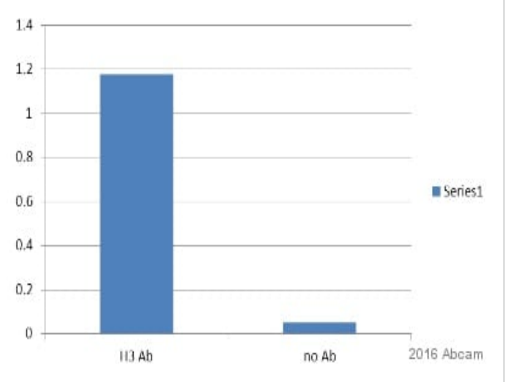


ChIP - Anti-Histone H3 antibody - Nuclear Loading Control and ChIP Grade (ab1791)

The ChIP was performed with chromatin from mouse gut cell lysate and ab1791 at 1/250 dilution.

**Negative control:** No antibody was used (right bar).

The immunoprecipitated DNA was quantified by real time PCR.

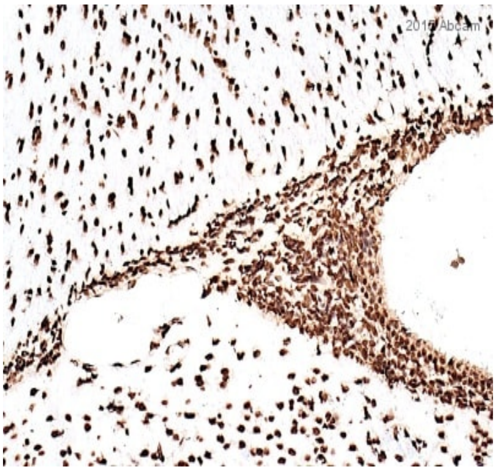

Immunohistochemistry (Formalin/PFA-fixed paraffin-embedded sections) - Anti-Histone H3 antibody - Nuclear Loading Control and ChIP Grade (ab1791)

Paraffin-embedded rat brain tissue stained for Histone H3 using ab1791 at 1/8000 dilution in immunohistochemical analysis.

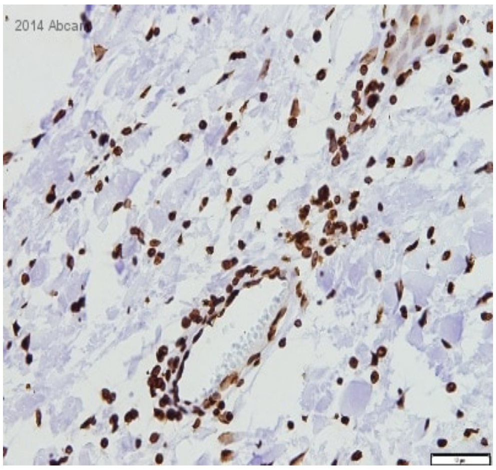


Immunohistochemistry (Formalin/PFA-fixed paraffin-embedded sections) - Anti-Histone H3 antibody - Nuclear Loading Control and ChIP Grade (ab1791)

This image is courtesy of an anonymous Abreview

ab1791 staining Histone H3 in human infantile fibromatosis tissue sections by Immunohistochemistry (IHC-P - paraformaldehyde-fixed, paraffin-embedded sections).

Tissue was fixed with formaldehyde and blocked with 1% FBS/BSA for 3 hours at room temperature; antigen retrieval was by heat mediation in Tris pH 9. Samples were incubated with primary antibody (1/100 in TBS + 1% BSA + 1% FBS) for 16 hours. An undiluted HRP-conjugated goat anti-rabbit IgG polyclonal was used as the secondary antibody.

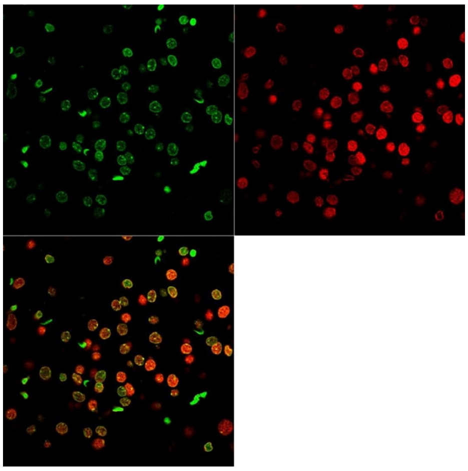

Immunohistochemistry (Formalin/PFA-fixed paraffin-embedded sections) - Anti-Histone H3 antibody - Nuclear Loading Control and ChIP Grade (ab1791)

ab1791 staining Histone H3 (red) in rat brain tissue sections by Immunohistochemistry (IHC-P - paraformaldehyde-fixed, paraffin-embedded sections).

Tissue was fixed with formaldehyde, permeabilized with 0.1% TBS-TritonX and blocked with 10% serum for 1 hour at 25°C; antigen retrieval was by heat mediation in a citrate buffer. Samples were incubated with the primary antibody (1/500 in 10% normal goat serum) for 24 hours at 24°C. An Alexa Fluor® 594-conjugated goat anti-rabbit IgG polyclonal (1/500) was used as the secondary antibody.

Green - Nucleus staining.
Red - Histone H3 staining.

**Please note:** All products are "FOR RESEARCH USE ONLY. NOT FOR USE IN DIAGNOSTIC PROCEDURES"

## Our Abpromise to you: Quality guaranteed and expert technical support

- Replacement or refund for products not performing as stated on the datasheet
- Valid for 12 months from date of delivery
- Response to your inquiry within 24 hours

- We provide support in Chinese, English, French, German, Japanese and Spanish
- Extensive multi-media technical resources to help you
- We investigate all quality concerns to ensure our products perform to the highest standards

If the product does not perform as described on this datasheet, we will offer a refund or replacement. For full details of the Abpromise, please visit https://www.abcam.cn/abpromise or contact our technical team.

## Terms and conditions

- Guarantee only valid for products bought direct from Abcam or one of our authorized distributors

abcam

## Product datasheet

# Anti-Aurora B antibody ab2254

★★★★☆ 36 Abreviews 196 References 11 图像

### 概述

| | |
|---|---|
| **产品名称** | Anti-Aurora B抗体 |
| **描述** | 兔多克隆抗体to Aurora B |
| **宿主** | Rabbit |
| **经测试应用** | **适用于:** ICC/IF, IHC-P, WB |
| **种属反应性** | **与反应:** Mouse, Rat, Human<br>**预测可用于:** Hamster, Pig |
| **免疫原** | Synthetic peptide. This information is proprietary to Abcam and/or its suppliers. |
| **常规说明** | The Life Science industry has been in the grips of a reproducibility crisis for a number of years. Abcam is leading the way in addressing this with our range of recombinant monoclonal antibodies and knockout edited cell lines for gold-standard validation. Please check that this product meets your needs before purchasing.<br>If you have any questions, special requirements or concerns, please send us an inquiry and/or contact our Support team ahead of purchase. Recommended alternatives for this product can be found below, along with publications, customer reviews and Q&As |

### 性能

| | |
|---|---|
| **形式** | Liquid |
| **存放说明** | Shipped at 4°C. Store at +4°C short term (1-2 weeks). Upon delivery aliquot. Store at -20°C or -80°C. Avoid freeze / thaw cycle. |
| **存储溶液** | pH: 7.40<br>Preservative: 0.02% Sodium azide<br>Constituent: PBS<br><br>Batches of this product that have a concentration < 1mg/ml may have BSA added as a stabilising agent. If you would like information about the formulation of a specific lot, please contact our scientific support team who will be happy to help. |
| **纯度** | Immunogen affinity purified |
| **克隆** | 多克隆 |
| **同种型** | IgG |

## 应用

**The Abpromise guarantee** Abpromise™承诺保证使用ab2254于以下的经测试应用

“应用说明”部分 下显示的仅为推荐的起始稀释度；实际最佳的稀释度/浓度应由使用者检定。

| 应用 | Ab评论 | 说明 |
|---|---|---|
| ICC/IF | ★★★★★ (13) | Use a concentration of 0.5 - 1 µg/ml.<br>Methanol fixation recommended. |
| IHC-P | ★★★★☆ (4) | 1/200. |
| WB | ★★★★☆ (14) | 1/1000 - 1/2000. Detects a band of approximately 39 kDa (predicted molecular weight: 39 kDa). |

## 靶标

**功能** May be directly involved in regulating the cleavage of polar spindle microtubules and is a key regulator for the onset of cytokinesis during mitosis. Component of the chromosomal passenger complex (CPC), a complex that acts as a key regulator of mitosis. The CPC complex has essential functions at the centromere in ensuring correct chromosome alignment and segregation and is required for chromatin-induced microtubule stabilization and spindle assembly. Phosphorylates 'Ser-10' and 'Ser-28' of histone H3 during mitosis. Required for kinetochore localization of BUB1 and SGOL1. Interacts with INCENP.

**组织特异性** High level expression seen in the thymus. It is also expressed in the spleen, lung, testis, colon, placenta and fetal liver. Expressed during S and G2/M phase and expression is up-regulated in cancer cells during M phase.

**疾病相关** Note=Disruptive regulation of expression is a possibile mechanism of the perturbation of chromosomal integrity in cancer cells through its dominant-negative effect on cytokinesis.

**序列相似性** Belongs to the protein kinase superfamily. Ser/Thr protein kinase family. Aurora subfamily. Contains 1 protein kinase domain.

**翻译后修饰** Ubiquitinated by different BCR (BTB-CUL3-RBX1) E3 ubiquitin ligase complexes. Ubiquitinated by the BCR(KLHL9-KLHL13) E3 ubiquitin ligase complex, ubiquitination leads to removal from mitotic chromosomes and is required for cytokinesis. During anaphase, the BCR(KLHL21) E3 ubiquitin ligase complex recruits the CPC complex from chromosomes to the spindle midzone and mediates the ubiquitination of AURKB. Ubiquitination of AURKB by BCR(KLHL21) E3 ubiquitin ligase complex may not lead to its degradation by the proteasome.

**细胞定位** Nucleus. Chromosome. Chromosome > centromere. Cytoplasm > cytoskeleton > spindle. Localizes on chromosome arms and inner centromeres from prophase through metaphase and then transferring to the spindle midzone and midbody from anaphase through cytokinesis. Colocalized with gamma tubulin in the mid-body.

## 图片

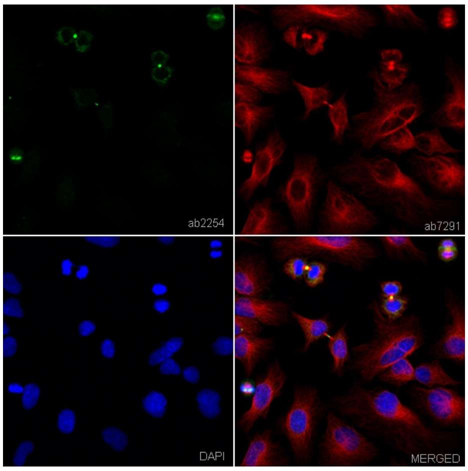


Immunocytochemistry/ Immunofluorescence - Anti-Aurora B antibody (ab2254)

ab2254 stained in Hela cells. Cells were fixed with 4% paraformaldehyde (10min) at room temperature and incubated with PBS containing 10% goat serum, 0.3 M glycine, 1% BSA and 0.1% triton for 1h at room temperature to permeabilise the cells and block non-specific protein-protein interactions. The cells were then incubated with the antibody ab2254 at 1µg/ml and ab7291 (Mouse monoclonal [DM1A] to alpha Tubulin - Loading Control) at 1/1000 dilution overnight at +4°C. The secondary antibodies were ab150120 (pseudo-colored red) and ab150081 (colored green) used at 1 ug/ml for 1hour at room temperature. DAPI was used to stain the cell nuclei (colored blue) at a concentration of 1.43µM for 1hour at room temperature.

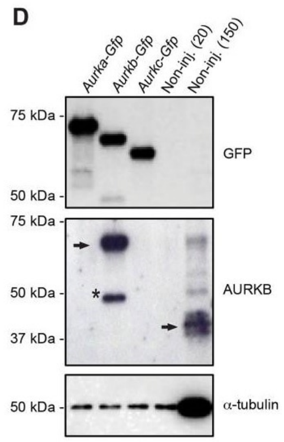


Western blot - Anti-Aurora B antibody (ab2254)

Balboula and Schindler PLoS Genet. 2014 Feb 27;10(2):e1004194. doi: 10.1371/journal.pgen.1004194. eCollection 2014 Feb. Fig 1. Reproduced under the Creative Commons license http://creativecommons.org/licenses/by/4.0/

**AURKB is expressed in mouse oocytes.**

(Panel D) 20 GV-intact oocytes were collected from CF1 mice and micro-injected with the indicated cRNA. Two hours after injection, the oocytes were matured to Met II *in vitro* (16 h). The total numbers of non-injected control oocytes (Non-inj.) are indicated in parenthesis. Total cellular lysates were probed with the indicated antibody. The panels are images of the same membrane that was stripped and re-probed. The arrows indicate the specific AURKB protein band, and the asterisk indicates a presumed degradation product of AURKB-GFP.

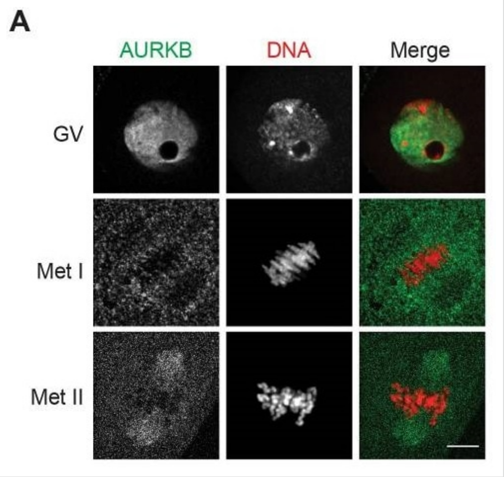


Immunocytochemistry/ Immunofluorescence - Anti-Aurora B antibody (ab2254)

Balboula and Schindler PLoS Genet. 2014 Feb 27;10(2):e1004194. doi: 10.1371/journal.pgen.1004194. eCollection 2014 Feb. Fig 1.

**AURKB is expressed in mouse oocytes.**

(Panel A) GV-intact oocytes were collected from CF1 mice and matured *in vitro* for 8 h (Met I), or 16 h (Met II), prior to fixation and staining with an anti-AURKB antibody (ab2254).

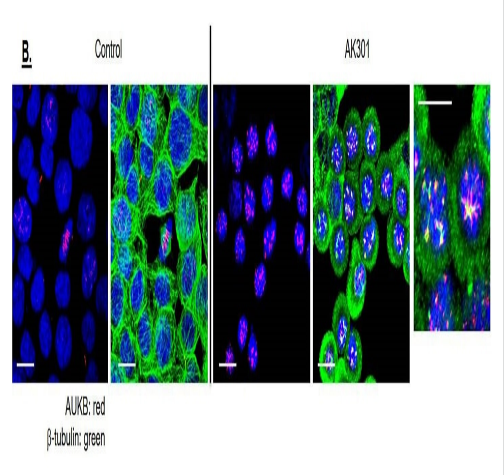


Immunocytochemistry/ Immunofluorescence - Anti-Aurora B antibody (ab2254)

Chopra et al PLoS One. 2016 Apr 20;11(4):e0153818. doi: 10.1371/journal.pone.0153818. eCollection 2016. Fig 6. Reproduced under the Creative Commons license http://creativecommons.org/licenses/by/4.0/

**HCT 116 (Human colorectal carcinoma cell line) cells were examined by immunofluorescence confocal microscopy.**

Cells were treated with 500 nM AK301 for 16 hours, and then processed for Aurora B (ab2254) and β-tubulin staining (**Panel B**). The color key and 20 µm bars are shown. Images of representative field is shown with a 20 µm bar. End-labeled DNA is shown in red and DAPI-stained DNA is blue.

Cells cultured on coverslips were fixed with 4% paraformaldehyde at room temperature or 100% ice cold methanol at 4°C and then permeabilized with 0.5% Triton X-100 in PBS. Cells were blocked in 5% serum (in PBS) and then incubated with primary antibody (in 5% serum) on shaker for 1 h at room temperature.

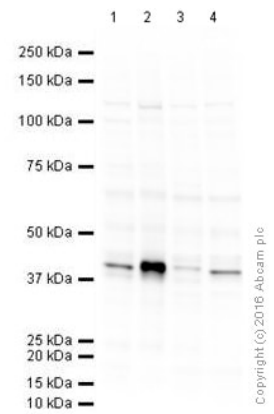


Western blot - Anti-Aurora B antibody (ab2254)

**All lanes :** Anti-Aurora B antibody (ab2254) at 1 µg/ml

**Lane 1 :** HeLa cell lysate

**Lane 2 :** HeLa nocodozole treated cell lysate

**Lane 3 :** NIH3T3 cell lysate

**Lane 4 :** PC12 cell lysate

Lysates/proteins at 10 µg per lane.

**Secondary**

**All lanes :** Goat Anti-Rabbit IgG H&L (HRP) (ab97051) at 1/50000 dilution

Performed under reducing conditions.

**Predicted band size:** 39 kDa

Blocked with 2% BSA.

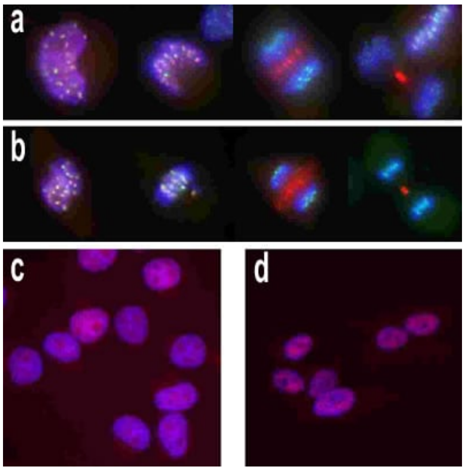


Immunocytochemistry/ Immunofluorescence - Anti-Aurora B antibody (ab2254)

Immunofluorescence in human cells using Rabbit polyclonal to Aurora B (red), DAPI (blue) and CREST serum (binds to centromeres)(green).

(a) HeLa cells - transition from interphase (left) through mitosis

(b) RPE-1 cells - as in (a)

(c) HeLa cells - interphase

(d) RPE-1 cells - interphase

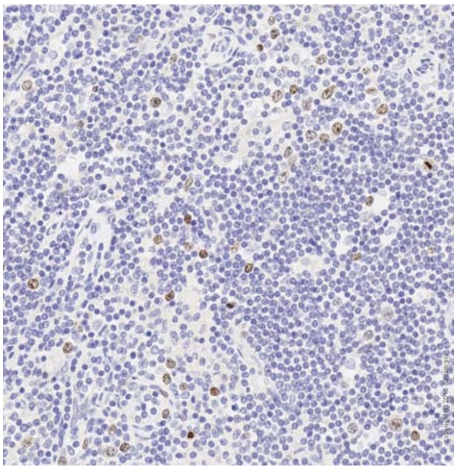

Immunohistochemistry (Formalin/PFA-fixed paraffin-embedded sections) - Anti-Aurora B antibody (ab2254)

IHC image of Aurora B staining in Human Lymph node Hodgkins disease formalin fixed paraffin embedded tissue section*, performed on a Leica Bond™ system using the standard protocol F. The section was pre-treated using heat mediated antigen retrieval with sodium citrate buffer (pH6, epitope retrieval solution 1) for 20 mins. The section was then incubated with ab2254, 5µg/ml, for 15 mins at room temperature and detected using an HRP conjugated compact polymer system. DAB was used as the chromogen. The section was then counterstained with haematoxylin and mounted with DPX.

For other IHC staining systems (automated and non-automated) customers should optimize variable parameters such as antigen retrieval conditions, primary antibody concentration and antibody incubation times.

*Tissue obtained from the Human Research Tissue Bank, supported by the NIHR Cambridge Biomedical Research Centre

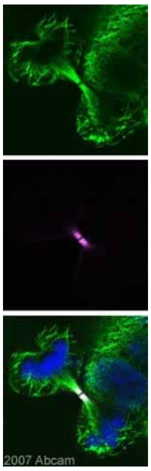


Immunocytochemistry/ Immunofluorescence - Anti-Aurora B antibody (ab2254)

This image is courtesy of an Abreview from Lux Fatimathas.

ab2254 staining human A431 (epithelial) cells by ICC/IF. The sample was fixed in paraformaldehyde and permeabilized by incubation with 0.1% Triton X100. 1% BSA was used as the blocking agent prior to a 1 hour incubation with the primary antibody, diluted 1/1000 with 1% BSA made up in PBS. An Alexa Fluor® 647 conjugated Donkey anti-Rabbit IgG (H+L) antibody was used as the secondary. Blocking and antibody incubation steps were carried out at room temperature.

In this set of images, the tubulin is stained green, Aurora B in pink and DNA in blue.

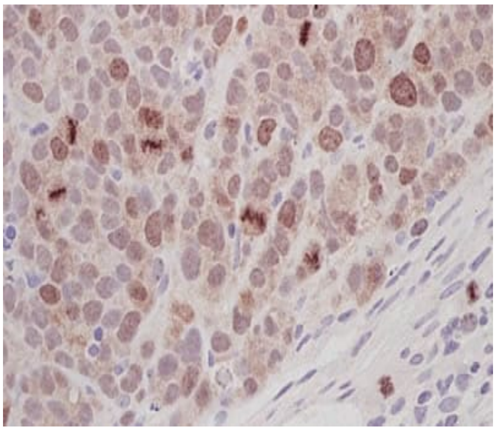

Immunohistochemistry (Formalin/PFA-fixed paraffin-embedded sections) - Anti-Aurora B antibody (ab2254)

Rabbit polyclonal to Aurora B (ab2254) used to stain SW620 human tumour xenografts (in mouse).

The sections were microwave pretreated in citrate buffer (pH 6.0) for 5 mins high then 5 mins simmer (800W conventional microwave). Slides were then incubated for 1 hour with the Aurora B primary antibody diluted 1/200 in TBS, then visualised using DAB, after application of an appropriate secondary.

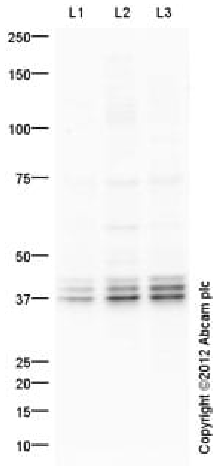


Western blot - Anti-Aurora B antibody (ab2254)

**All lanes :** Anti-Aurora B antibody (ab2254) at 1 µg/ml

**Lane 1 :** HeLa Whole Cell Lysate
**Lane 2 :** HeLa Nuclear Lysate
**Lane 3 :** Jurkat Whole Cell Lysate

Lysates/proteins at 20 µg per lane.

**Secondary**
**All lanes :** Goat Anti-Rabbit IgG H&L (HRP) (ab97051) at 1/10000 dilution

Developed using the ECL technique.

Performed under reducing conditions.

**Predicted band size:** 39 kDa
**Observed band size:** 39 kDa
**Additional bands at:** 37 kDa (possible isoform)

**Exposure time:** 150 seconds

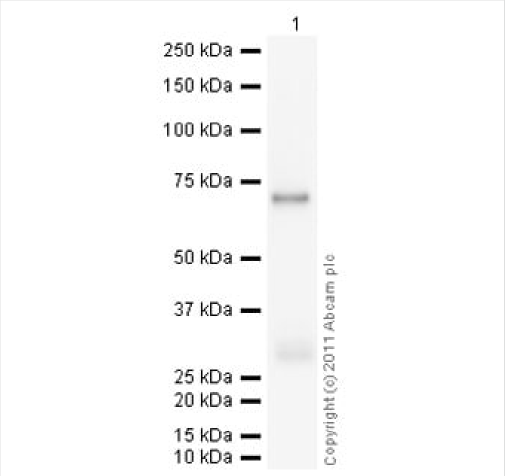


Western blot - Anti-Aurora B antibody (ab2254)

Anti-Aurora B antibody (ab2254) at 1/2000 dilution + Recombinant human Aurora B protein (ab51435) at 0.1 µg

**Secondary**

Goat Anti-Rabbit IgG H&L (HRP) preadsorbed (ab97080) at 1/5000 dilution

Developed using the ECL technique.

Performed under reducing conditions.

**Predicted band size:** 39 kDa

**Exposure time:** 30 seconds

**Please note:** All products are "FOR RESEARCH USE ONLY. NOT FOR USE IN DIAGNOSTIC PROCEDURES"

## Our Abpromise to you: Quality guaranteed and expert technical support

- Replacement or refund for products not performing as stated on the datasheet
- Valid for 12 months from date of delivery
- Response to your inquiry within 24 hours

- We provide support in Chinese, English, French, German, Japanese and Spanish
- Extensive multi-media technical resources to help you
- We investigate all quality concerns to ensure our products perform to the highest standards

If the product does not perform as described on this datasheet, we will offer a refund or replacement. For full details of the Abpromise, please visit https://www.abcam.cn/abpromise or contact our technical team.

## Terms and conditions

- Guarantee only valid for products bought direct from Abcam or one of our authorized distributors

abcam

# Product datasheet

# Anti-Histone H3 (acetyl K27) antibody - ChIP Grade ab4729

★★★★★ 81 Abreviews 1539 References 10 图像

## 概述

| | |
|---|---|
| **产品名称** | Anti-Histone H3 (acetyl K27)抗体- ChIP Grade |
| **描述** | 兔多克隆抗体to Histone H3 (acetyl K27) - ChIP Grade |
| **宿主** | Rabbit |
| **经测试应用** | **适用于:** ICC/IF, WB, IHC-P, ChIP, PepArr |
| **种属反应性** | **与反应:** Mouse, Rat, Cow, Human, Recombinant fragment<br>**预测可用于:** Chicken, Xenopus laevis, Arabidopsis thaliana, Drosophila melanogaster, Monkey, Zebrafish, Plasmodium falciparum, Rice, Cyanidioschyzon merolae |
| **免疫原** | Synthetic peptide corresponding to Human Histone H3 aa 1-100 (acetyl K27) conjugated to keyhole limpet haemocyanin.<br>(Peptide available as ab24404) |
| **阳性对照** | WB : HeLa (human cervix adenocarcinoma epithelial cell) cell lysate - Sodium butyrate-treated, HeLa (human cervix adenocarcinoma epithelial cell) nuclear lysate (triton enriched), NIH/3T3 (mouse embryonic fibroblast cell line) nuclear lysate (triton enriched) and PC-12 (rat adrenal gland pheochromocytoma cell) nuclear lysate (triton enriched). |
| **常规说明** | Learn about ChIP assay kits, other ChIP antibodies, protocols and more in the ChIP assay guide.<br><br>The Life Science industry has been in the grips of a reproducibility crisis for a number of years. Abcam is leading the way in addressing this with our range of recombinant monoclonal antibodies and knockout edited cell lines for gold-standard validation. Please check that this product meets your needs before purchasing.<br><br>If you have any questions, special requirements or concerns, please send us an inquiry and/or contact our Support team ahead of purchase. Recommended alternatives for this product can be found below, along with publications, customer reviews and Q&As |

## 性能

| | |
|---|---|
| **形式** | Liquid |
| **存放说明** | Shipped at 4°C. Store at +4°C short term (1-2 weeks). Upon delivery aliquot. Store at -20°C or -80°C. Avoid freeze / thaw cycle. |
| **存储溶液** | pH: 7.40<br>Preservative: 0.02% Sodium azide<br>Constituent: PBS |

| | |
|---|---|
| | Batches of this product that have a concentration < 1mg/ml may have BSA added as a stabilising agent. If you would like information about the formulation of a specific lot, please contact our scientific support team who will be happy to help. |
| **纯度** | Immunogen affinity purified |
| **克隆** | 多克隆 |
| **同种型** | IgG |

## 应用

**The Abpromise guarantee** Abpromise™承诺保证使用ab4729于以下的经测试应用

“应用说明”部分 下显示的仅为推荐的起始稀释度；实际最佳的稀释度/浓度应由使用者检定。

| 应用 | Ab评论 | 说明 |
|---|---|---|
| **ICC/IF** | ★★★★★ (21) | Use a concentration of 0.5 µg/ml.<br>Can be used with paraformaldehyde- or methanol- fixed cells. |
| **WB** | ★★★★★ (20) | Use a concentration of 1 µg/ml. Detects a band of approximately 17 kDa (predicted molecular weight: 15 kDa).<br>We recommend Goat Anti-Rabbit IgG H&L (HRP) (ab97051) secondary antibody. |
| **IHC-P** | ★★★★★ (4) | Use a concentration of 1 µg/ml. Perform heat mediated antigen retrieval before commencing with IHC staining protocol. |
| **ChIP** | ★★★★★ (27) | Use 2 µg for 25 µg of chromatin.<br>We recommend GAPDH positive control ChIP primer pair ab267832 as a positive control. |
| **PepArr** | | Use a concentration of 0.2 - 0.02 µg/ml. |

## 靶标

| | |
|---|---|
| **功能** | Core component of nucleosome. Nucleosomes wrap and compact DNA into chromatin, limiting DNA accessibility to the cellular machineries which require DNA as a template. Histones thereby play a central role in transcription regulation, DNA repair, DNA replication and chromosomal stability. DNA accessibility is regulated via a complex set of post-translational modifications of histones, also called histone code, and nucleosome remodeling. |
| **序列相似性** | Belongs to the histone H3 family. |
| **发展阶段** | Expressed during S phase, then expression strongly decreases as cell division slows down during the process of differentiation. |
| **翻译后修饰** | Acetylation is generally linked to gene activation. Acetylation on Lys-10 (H3K9ac) impairs methylation at Arg-9 (H3R8me2s). Acetylation on Lys-19 (H3K18ac) and Lys-24 (H3K24ac) favors methylation at Arg-18 (H3R17me).<br>Citrullination at Arg-9 (H3R8ci) and/or Arg-18 (H3R17ci) by PADI4 impairs methylation and represses transcription.<br>Asymmetric dimethylation at Arg-18 (H3R17me2a) by CARM1 is linked to gene activation. |

Symmetric dimethylation at Arg-9 (H3R8me2s) by PRMT5 is linked to gene repression. Asymmetric dimethylation at Arg-3 (H3R2me2a) by PRMT6 is linked to gene repression and is mutually exclusive with H3 Lys-5 methylation (H3K4me2 and H3K4me3). H3R2me2a is present at the 3' of genes regardless of their transcription state and is enriched on inactive promoters, while it is absent on active promoters.
Methylation at Lys-5 (H3K4me), Lys-37 (H3K36me) and Lys-80 (H3K79me) are linked to gene activation. Methylation at Lys-5 (H3K4me) facilitates subsequent acetylation of H3 and H4. Methylation at Lys-80 (H3K79me) is associated with DNA double-strand break (DSB) responses and is a specific target for TP53BP1. Methylation at Lys-10 (H3K9me) and Lys-28 (H3K27me) are linked to gene repression. Methylation at Lys-10 (H3K9me) is a specific target for HP1 proteins (CBX1, CBX3 and CBX5) and prevents subsequent phosphorylation at Ser-11 (H3S10ph) and acetylation of H3 and H4. Methylation at Lys-5 (H3K4me) and Lys-80 (H3K79me) require preliminary monoubiquitination of H2B at 'Lys-120'. Methylation at Lys-10 (H3K9me) and Lys-28 (H3K27me) are enriched in inactive X chromosome chromatin.
Phosphorylated at Thr-4 (H3T3ph) by GSG2/haspin during prophase and dephosphorylated during anaphase. Phosphorylation at Ser-11 (H3S10ph) by AURKB is crucial for chromosome condensation and cell-cycle progression during mitosis and meiosis. In addition phosphorylation at Ser-11 (H3S10ph) by RPS6KA4 and RPS6KA5 is important during interphase because it enables the transcription of genes following external stimulation, like mitogens, stress, growth factors or UV irradiation and result in the activation of genes, such as c-fos and c-jun. Phosphorylation at Ser-11 (H3S10ph), which is linked to gene activation, prevents methylation at Lys-10 (H3K9me) but facilitates acetylation of H3 and H4. Phosphorylation at Ser-11 (H3S10ph) by AURKB mediates the dissociation of HP1 proteins (CBX1, CBX3 and CBX5) from heterochromatin. Phosphorylation at Ser-11 (H3S10ph) is also an essential regulatory mechanism for neoplastic cell transformation. Phosphorylated at Ser-29 (H3S28ph) by MLTK isoform 1, RPS6KA5 or AURKB during mitosis or upon ultraviolet B irradiation. Phosphorylation at Thr-7 (H3T6ph) by PRKCBB is a specific tag for epigenetic transcriptional activation that prevents demethylation of Lys-5 (H3K4me) by LSD1/KDM1A. At centromeres, specifically phosphorylated at Thr-12 (H3T11ph) from prophase to early anaphase, by DAPK3 and PKN1. Phosphorylation at Thr-12 (H3T11ph) by PKN1 is a specific tag for epigenetic transcriptional activation that promotes demethylation of Lys-10 (H3K9me) by KDM4C/JMJD2C. Phosphorylation at Tyr-42 (H3Y41ph) by JAK2 promotes exclusion of CBX5 (HP1 alpha) from chromatin.
Monoubiquitinated by RAG1 in lymphoid cells, monoubiquitination is required for V(D)J recombination (By similarity). Ubiquitinated by the CUL4-DDB-RBX1 complex in response to ultraviolet irradiation. This may weaken the interaction between histones and DNA and facilitate DNA accessibility to repair proteins.

| | |
|---|---|
| **细胞定位** | Nucleus. Chromosome. |

## 图片

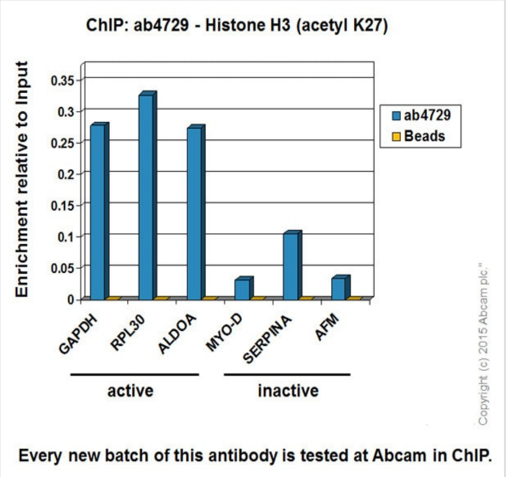


ChIP - Anti-Histone H3 (acetyl K27) antibody - ChIP Grade (ab4729)

Chromatin was prepared from HeLa (Human epithelial cell line from cervix adenocarcinoma) cells according to the Abcam X-ChIP protocol. Cells were fixed with formaldehyde for 10 minutes. The ChIP was performed with 25 µg of chromatin, 2 µg of ab4729 (blue), and 20 µl of Protein A/G sepharose beads.

No antibody was added to the beads control (yellow).

The immunoprecipitated DNA was quantified by real time PCR (Taqman approach). Primers and probes are located in the first kb of the transcribed region.

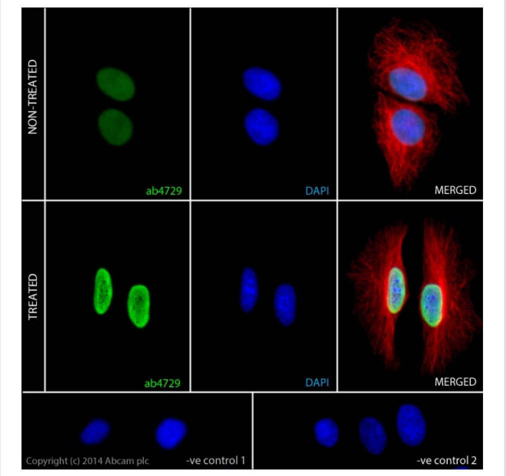


Immunocytochemistry/ Immunofluorescence - Anti-Histone H3 (acetyl K27) antibody - ChIP Grade (ab4729)

ab4729 staining Histone H3 (acetyl K27) in HeLa (Human epithelial cell line from cervix adenocarcinoma) cells.

The cells were incubated with 10 mM sodium butyrate (ab120948) for 6 hours (Treated) or solvent-only for control purposes (Non-treated). Cells were fixed with 100% methanol (5 minutes) and then blocked in 1% BSA/10% normal goat serum/0.3M glycine in 0.1%PBS-Tween for 1 hour. The cells were then incubated with ab4729 at 0.5 µg/ml and ab7291 at 1 µg/ml overnight at +4°C, followed by a further incubation at room temperature for 1 hour with a anti-rabbit AlexaFluor®488 secondary antibody (ab150077) at 2 µg/ml (shown in green) and a goat anti-mouse AlexaFluor®594 (ab150120) at 2 µg/ml (shown in pseudo colour red). Nuclear DNA was labeled in blue with DAPI.

**Negative controls:** 1– Rabbit primary and anti-mouse secondary antibody; 2 – Mouse primary antibody and anti-rabbit secondary antibody. Controls 1 and 2 indicate that there is no unspecific reaction between primary and secondary antibodies used.

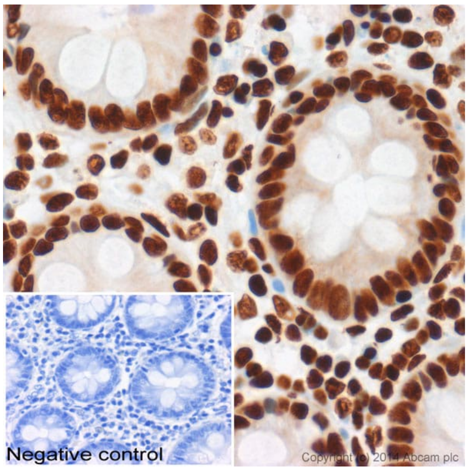


Immunohistochemistry (Formalin/PFA-fixed paraffin-embedded sections) - Anti-Histone H3 (acetyl K27) antibody - ChIP Grade (ab4729)

IHC image of ab4729 staining Histone H3 (acetyl K27) in human colon formalin-fixed paraffin-embedded tissue sections*, performed on a Leica Bond.

The section was pre-treated using heat mediated antigen retrieval with sodium citrate buffer pH 6 for 20 minutes. The section was then incubated with ab4729, 5 µg/ml, for 15 minutes at room temperature and detected using an HRP conjugated compact polymer system. DAB was used as the chromogen. The section was then counterstained with haematoxylin and mounted with DPX.

No primary antibody was used in the negative control (shown on the inset).

For other IHC staining systems (automated and non-automated) customers should optimize variable parameters such as antigen retrieval conditions, primary antibody concentration and antibody incubation times.

*Tissue obtained from the Human Research Tissue Bank, supported by the NIHR Cambridge Biomedical Research Centre

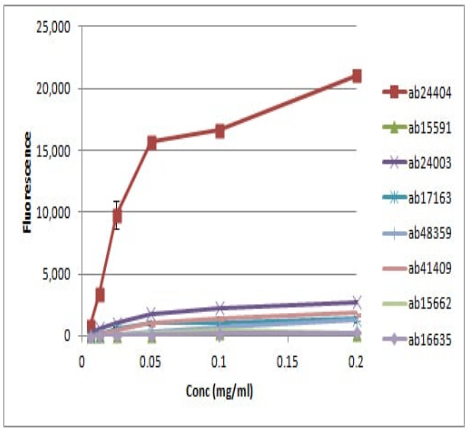


Peptide Array - Anti-Histone H3 (acetyl K27) antibody - ChIP Grade (ab4729)

All batches of ab4729 are tested in Peptide Array against peptides to different Histone H3 modifications. Six dilutions of each peptide are printed on to the Peptide Array in triplicate and results are averaged before being plotted on to a graph. Results show strong binding to Histone H3 - acetyl K27 peptide (ab24404), indicating that this antibody specifically recognises the Histone H3 - acetyl K27 modification.

ab24404 - Histone H3 - acetyl K27

ab15591 - Histone H3 - acetyl K14

ab24003 - Histone H3 - acetyl K18

ab17163 - Histone H3 unmodified

ab48359 - Histone H3 - acetyl K23

ab41409 - Histone H3 - acetyl K36

ab15662 - Histone H4 - acetyl K12

ab16635 - Histone H3 acetyl K9

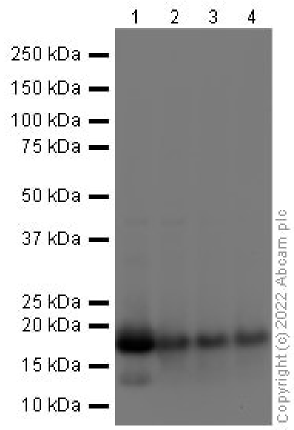


Western blot - Anti-Histone H3 (acetyl K27) antibody - ChIP Grade (ab4729)

**All lanes :** Anti-Histone H3 (acetyl K27) antibody - ChIP Grade (ab4729) at 1 µg/ml

**Lane 1 :** HeLa (human cervix adenocarcinoma epithelial cell) cell lysate - Sodium butyrate-treated
**Lane 2 :** HeLa (human cervix adenocarcinoma epithelial cell) nuclear lysate (triton enriched)
**Lane 3 :** NIH/3T3 (mouse embryonic fibroblast cell line) nuclear lysate (triton enriched)
**Lane 4 :** PC-12 (rat adrenal gland pheochromocytoma cell) nuclear lysate (triton enriched)

Lysates/proteins at 10 µg per lane.

**Secondary**
**All lanes :** Goat polyclonal to Rabbit IgG - H&L - Pre-Adsorbed (HRP) at 1/50000 dilution

**Predicted band size:** 15 kDa
**Observed band size:** 17 kDa

**Exposure time:** 30 seconds

**Blocking buffer :** 2% BSA block

**Gel type :** MES

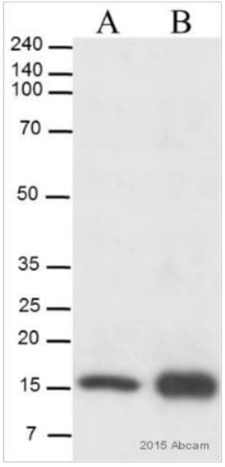


Western blot - Anti-Histone H3 (acetyl K27) antibody - ChIP Grade (ab4729)

**All lanes :** Anti-Histone H3 (acetyl K27) antibody - ChIP Grade (ab4729) at 1/2500 dilution

**Lane 1 :** Untreated Mouse MEF cell lysate

**Lane 2 :** 0.4 µM Trichostatin A treatment for 18 hr Mouse MEF cell lysate

Lysates/proteins at 9 µg per lane.

**Secondary**

**All lanes :** Donkey Anti-Rabbit IgG H&L (HRP) (ab6802) at 1/20000 dilution

**Predicted band size:** 15 kDa

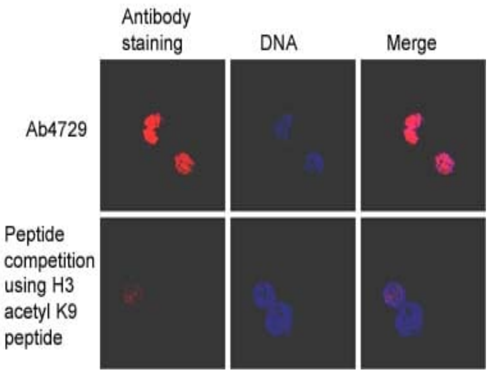


Immunocytochemistry/ Immunofluorescence - Anti-Histone H3 (acetyl K27) antibody - ChIP Grade (ab4729)

This image is courtesy of Petra Hajkova - Gurdon Institute, Cambridge University

Primary antibody: ab4729 (H3 acetyl K27)

Dilution: 1/100

ab4729 strongly stained histones of mouse ES cells. However, fluroescence was greatly diminished following pre-blocking using a H3 acetyl K9 peptide. This suggests the antibody cross-reacts with the K9 and K27 residues.

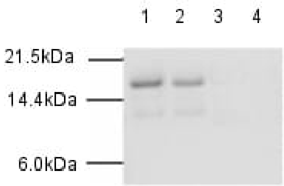


Western blot - Anti-Histone H3 (acetyl K27) antibody - ChIP Grade (ab4729)

**Lanes 1 & 3 :** Anti-Histone H3 (acetyl K27) antibody - ChIP Grade (ab4729) at 0.2 µg/ml

**Lanes 2 & 4 :** Anti-Histone H3 (acetyl K27) antibody - ChIP Grade (ab4729) at 0.1 µg/ml

**Lanes 1-2 :** Calf thymus histone lysate

**Lanes 3-4 :** Calf thymus histone lysate with Human Histone H3 (acetyl K27) peptide (ab24404) at 2 µg

Lysates/proteins at 1 µg per lane.

**Secondary**

**All lanes :** Goat anti-rabbit (HRP) at 1/2000 dilution

**Predicted band size:** 15 kDa
**Observed band size:** 17 kDa

ab4729 specifically recognises acetyl K27 histone H3 in catlf thymus histone lysate, which is specifically blocked using the immunizing peptide ab24404.

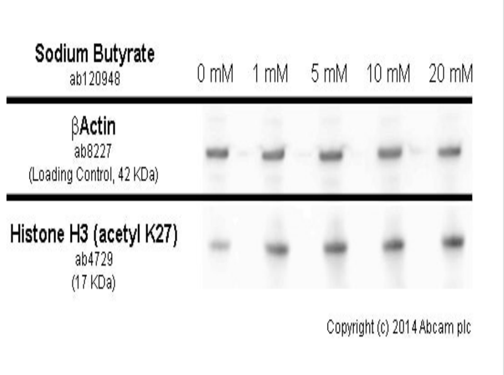


Western blot - Anti-Histone H3 (acetyl K27) antibody - ChIP Grade (ab4729)

HeLa (Human epithelial cell line from cervix adenocarcinoma) cells were incubated at 37°C for 6 hours with vehicle control (0 µM) and different concentrations of sodium butyrate (ab120948). Increased expression of histone H3 (acetyl K27)(ab4729) in HeLa cells correlates with an increase in sodium butyrate concentration, as described in literature.

Whole cell lysates were prepared with RIPA buffer (containing protease inhibitors and sodium orthovanadate), 2.5 µg of each were loaded on the gel and the WB was run under reducing conditions. After transfer the membrane was blocked for an hour using 5% BSA before being incubated with ab4927 at 1 µg/ml and ab8227 at 1 µg/ml overnight at 4°C. Antibody binding was detected using an anti-rabbit antibody conjugated to HRP (ab97051) at 1/10,000 dilution and visualised using ECL development solution.

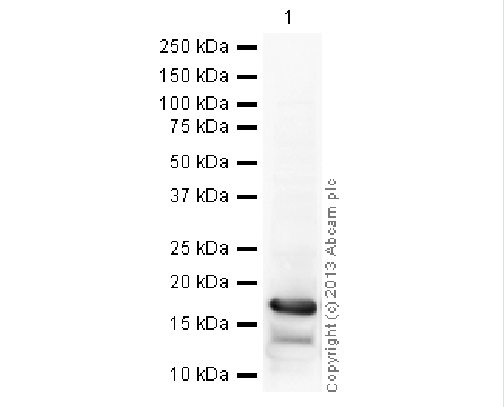


Western blot - Anti-Histone H3 (acetyl K27) antibody - ChIP Grade (ab4729)

Anti-Histone H3 (acetyl K27) antibody - ChIP Grade (ab4729) at 1 µg/ml + HeLa (Human epithelial cell line from cervix adenocarcinoma) histone preparation, nuclear Lysate - Butyrate treated at 2.5 µg

**Secondary**
Goat Anti-Rabbit IgG H&L (HRP) (ab97051) at 1/10000 dilution

Developed using the ECL technique.

Performed under reducing conditions.

**Predicted band size:** 15 kDa
**Additional bands at:** 17 kDa. We are unsure as to the identity of these extra bands.

**Exposure time:** 10 seconds

**Please note:** All products are "FOR RESEARCH USE ONLY. NOT FOR USE IN DIAGNOSTIC PROCEDURES"

## Our Abpromise to you: Quality guaranteed and expert technical support

- Replacement or refund for products not performing as stated on the datasheet
- Valid for 12 months from date of delivery
- Response to your inquiry within 24 hours

- We provide support in Chinese, English, French, German, Japanese and Spanish
- Extensive multi-media technical resources to help you
- We investigate all quality concerns to ensure our products perform to the highest standards

If the product does not perform as described on this datasheet, we will offer a refund or replacement. For full details of the Abpromise, please visit https://www.abcam.cn/abpromise or contact our technical team.

## Terms and conditions

- Guarantee only valid for products bought direct from Abcam or one of our authorized distributors

abcam

# Product datasheet

# Anti-Histone H3 (tri methyl K27) antibody [mAbcam 6002] - ChIP Grade ab6002

★★★★☆ 71 Abreviews 794 References 8 图像

## 概述

| | |
|---|---|
| 产品名称 | Anti-Histone H3 (tri methyl K27)抗体[mAbcam 6002] - ChIP Grade |
| 描述 | 小鼠单克隆抗体[mAbcam 6002] to Histone H3 (tri methyl K27) - ChIP Grade |
| 宿主 | Mouse |
| 特异性 | This antibody is specific for histone H3 tri-methylated at K27. The antibody is blocked in Western blot by tri methyl K27 peptide and slightly by di methyl K27 peptide (there is <12% cross reactivity with di methyl K27 as determined by ELISA). It is not blocked by mono methyl K4, di methyl K4, tri methyl K4, mono methyl K9, di methyl K9, tri methyl K9, mono methyl K27 or unmodified K27 peptides (see the peptide blocking assay blot below). |
| 经测试应用 | **适用于:** ChIP, ELISA, WB, IHC - Wholemount, ICC/IF |
| 种属反应性 | **与反应:** Mouse, Cow, Human, Recombinant fragment<br>**预测可用于:** Rat, Rabbit, Chicken, Xenopus laevis, Arabidopsis thaliana, Drosophila melanogaster, Plants, Zebrafish, Rhesus monkey, Chinese hamster, Rice |
| 免疫原 | Synthetic peptide. This information is proprietary to Abcam and/or its suppliers.<br>(Peptide available as ab1782) |
| 阳性对照 | WB: Calf Thymus Histone Preparation Nuclear Lysate, HeLa cell lysates, mouse ES whole cell lysate ChIP: HeLa cells and K562 cells. ICC/IF: HeLa cells. IHC-Wholemount: blastocysts and germ cells and in culture in pluripotent stem cells. ELISA: Histone H3 - tri methyl K27 peptide. |
| 常规说明 | This antibody clone is manufactured by Abcam. If you require a custom buffer formulation or conjugation for your experiments, please contact orders@abcam.com.<br><br>The Life Science industry has been in the grips of a reproducibility crisis for a number of years. Abcam is leading the way in addressing this with our range of recombinant monoclonal antibodies and knockout edited cell lines for gold-standard validation. Please check that this product meets your needs before purchasing.<br><br>If you have any questions, special requirements or concerns, please send us an inquiry and/or contact our Support team ahead of purchase. Recommended alternatives for this product can be found below, along with publications, customer reviews and Q&As |

## 性能

| | |
|---|---|
| 形式 | Liquid |

| | |
|---|---|
| **存放说明** | Shipped at 4°C. Store at +4°C short term (1-2 weeks). Upon delivery aliquot. Store at -20°C or -80°C. Avoid freeze / thaw cycle. |
| **存储溶液** | pH: 7.40<br>Preservative: 0.02% Sodium azide<br>Constituents: PBS, 6.97% L-Arginine |
| **纯度** | Protein A purified |
| **克隆** | 单克隆 |
| **克隆编号** | mAbcam 6002 |
| **同种型** | IgG3 |
| **轻链类型** | kappa |

## 应用

**The Abpromise guarantee** Abpromise™承诺保证使用ab6002于以下的经测试应用

"应用说明"部分 下显示的仅为推荐的起始稀释度；实际最佳的稀释度/浓度应由使用者检定。

| 应用 | Ab评论 | 说明 |
|---|---|---|
| **ChIP** | ★★★★★ (19) | Use 5-10 µg for 25 µg of chromatin.<br>Use Myo-D ChIP primer pair ab269261 as positive control. |
| **ELISA** | | Use a concentration of 0.025 - 1 µg/ml. |
| **WB** | ★★★★☆ (20) | Use a concentration of 1 - 5 µg/ml. Detects a band of approximately 17 kDa (predicted molecular weight: 15 kDa).Can be blocked with Human Histone H3 (tri methyl K27) peptide (ab1782).<br>Blocking: we recommend using 3% milk block for 1 hour at room temperature. This step may need to be optimized for your experiments. |
| **IHC - Wholemount** | | Use at an assay dependent concentration. |
| **ICC/IF** | ★★★★☆ (17) | Use a concentration of 5 µg/ml. |

## 靶标

| | |
|---|---|
| **功能** | Core component of nucleosome. Nucleosomes wrap and compact DNA into chromatin, limiting DNA accessibility to the cellular machineries which require DNA as a template. Histones thereby play a central role in transcription regulation, DNA repair, DNA replication and chromosomal stability. DNA accessibility is regulated via a complex set of post-translational modifications of histones, also called histone code, and nucleosome remodeling. |
| **序列相似性** | Belongs to the histone H3 family. |
| **发展阶段** | Expressed during S phase, then expression strongly decreases as cell division slows down during the process of differentiation. |
| **翻译后修饰** | Acetylation is generally linked to gene activation. Acetylation on Lys-10 (H3K9ac) impairs methylation at Arg-9 (H3R8me2s). Acetylation on Lys-19 (H3K18ac) and Lys-24 (H3K24ac) |

favors methylation at Arg-18 (H3R17me).
Citrullination at Arg-9 (H3R8ci) and/or Arg-18 (H3R17ci) by PADI4 impairs methylation and represses transcription.
Asymmetric dimethylation at Arg-18 (H3R17me2a) by CARM1 is linked to gene activation.
Symmetric dimethylation at Arg-9 (H3R8me2s) by PRMT5 is linked to gene repression.
Asymmetric dimethylation at Arg-3 (H3R2me2a) by PRMT6 is linked to gene repression and is mutually exclusive with H3 Lys-5 methylation (H3K4me2 and H3K4me3). H3R2me2a is present at the 3' of genes regardless of their transcription state and is enriched on inactive promoters, while it is absent on active promoters.
Methylation at Lys-5 (H3K4me), Lys-37 (H3K36me) and Lys-80 (H3K79me) are linked to gene activation. Methylation at Lys-5 (H3K4me) facilitates subsequent acetylation of H3 and H4. Methylation at Lys-80 (H3K79me) is associated with DNA double-strand break (DSB) responses and is a specific target for TP53BP1. Methylation at Lys-10 (H3K9me) and Lys-28 (H3K27me) are linked to gene repression. Methylation at Lys-10 (H3K9me) is a specific target for HP1 proteins (CBX1, CBX3 and CBX5) and prevents subsequent phosphorylation at Ser-11 (H3S10ph) and acetylation of H3 and H4. Methylation at Lys-5 (H3K4me) and Lys-80 (H3K79me) require preliminary monoubiquitination of H2B at 'Lys-120'. Methylation at Lys-10 (H3K9me) and Lys-28 (H3K27me) are enriched in inactive X chromosome chromatin.
Phosphorylated at Thr-4 (H3T3ph) by GSG2/haspin during prophase and dephosphorylated during anaphase. Phosphorylation at Ser-11 (H3S10ph) by AURKB is crucial for chromosome condensation and cell-cycle progression during mitosis and meiosis. In addition phosphorylation at Ser-11 (H3S10ph) by RPS6KA4 and RPS6KA5 is important during interphase because it enables the transcription of genes following external stimulation, like mitogens, stress, growth factors or UV irradiation and result in the activation of genes, such as c-fos and c-jun. Phosphorylation at Ser-11 (H3S10ph), which is linked to gene activation, prevents methylation at Lys-10 (H3K9me) but facilitates acetylation of H3 and H4. Phosphorylation at Ser-11 (H3S10ph) by AURKB mediates the dissociation of HP1 proteins (CBX1, CBX3 and CBX5) from heterochromatin. Phosphorylation at Ser-11 (H3S10ph) is also an essential regulatory mechanism for neoplastic cell transformation. Phosphorylated at Ser-29 (H3S28ph) by MLTK isoform 1, RPS6KA5 or AURKB during mitosis or upon ultraviolet B irradiation. Phosphorylation at Thr-7 (H3T6ph) by PRKCBB is a specific tag for epigenetic transcriptional activation that prevents demethylation of Lys-5 (H3K4me) by LSD1/KDM1A. At centromeres, specifically phosphorylated at Thr-12 (H3T11ph) from prophase to early anaphase, by DAPK3 and PKN1. Phosphorylation at Thr-12 (H3T11ph) by PKN1 is a specific tag for epigenetic transcriptional activation that promotes demethylation of Lys-10 (H3K9me) by KDM4C/JMJD2C. Phosphorylation at Tyr-42 (H3Y41ph) by JAK2 promotes exclusion of CBX5 (HP1 alpha) from chromatin.
Monoubiquitinated by RAG1 in lymphoid cells, monoubiquitination is required for V(D)J recombination (By similarity). Ubiquitinated by the CUL4-DDB-RBX1 complex in response to ultraviolet irradiation. This may weaken the interaction between histones and DNA and facilitate DNA accessibility to repair proteins.

| | |
|---|---|
| **细胞定位** | Nucleus. Chromosome. |

**图片**

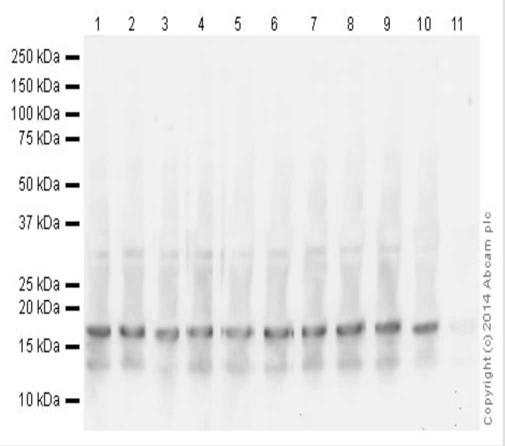


Western blot - Anti-Histone H3 (tri methyl K27) antibody [mAbcam 6002] - ChIP Grade (ab6002)

**All lanes :** Anti-Histone H3 (tri methyl K27) antibody [mAbcam 6002] - ChIP Grade (ab6002) at 1 µg/ml

**Lane 1 :** Calf Thymus Histone Preparation Nuclear Lysate
**Lane 2 :** Calf Thymus Histone Preparation Nuclear Lysate with Human Histone H3 peptide (ab17163) at 0.5 µg/ml
**Lane 3 :** Calf Thymus Histone Preparation Nuclear Lysate with Human Histone H3 (mono methyl K4) peptide (ab1340) at 0.5 µg/ml
**Lane 4 :** Calf Thymus Histone Preparation Nuclear Lysate with Human Histone H3 (di methyl K4) peptide (ab7768) at 0.5 µg/ml
**Lane 5 :** Calf Thymus Histone Preparation Nuclear Lysate with Human Histone H3 (tri methyl K4) peptide (ab1342) at 0.5 µg/ml
**Lane 6 :** Calf Thymus Histone Preparation Nuclear Lysate with Human Histone H3 (mono methyl K9) peptide (ab1771) at 0.5 µg/ml
**Lane 7 :** Calf Thymus Histone Preparation Nuclear Lysate with Human Histone H3 (di methyl K9) peptide (ab1772) at 0.5 µg/ml
**Lane 8 :** Calf Thymus Histone Preparation Nuclear Lysate with Human Histone H3 (tri methyl K9) peptide (ab1773) at 0.5 µg/ml
**Lane 9 :** Calf Thymus Histone Preparation Nuclear Lysate with Human Histone H3 (mono methyl K27) peptide (ab1780) at 0.5 µg/ml
**Lane 10 :** Calf Thymus Histone Preparation Nuclear Lysate with Human Histone H3 (di methyl K27) peptide (ab1781) at 0.5 µg/ml
**Lane 11 :** Calf Thymus Histone Preparation Nuclear Lysate with Human Histone H3 (tri methyl K27) peptide (ab1782) at 0.5 µg/ml

Lysates/proteins at 0.25 µg per lane.

**Secondary**
**All lanes :** Goat Anti-Mouse IgG H&L (HRP) preadsorbed (ab97040) at 1/50000 dilution

Developed using the ECL technique.

Performed under reducing conditions.

**Predicted band size:** 15 kDa
**Observed band size:** 17 kDa

**Exposure time:** 3 minutes

This blot was produced using a 4-12% Bis-tris gel under the MES buffer system. The gel was run at 200V for 35 minutes before being transferred onto a Nitrocellulose membrane at 30V for 70 minutes. The membrane was then blocked for an hour using 3% milk before being incubated with ab6002 overnight at 4°C. Antibody binding was detected using an anti-mouse antibody conjugated to HRP, and visualised using ECL development solution ab133406.

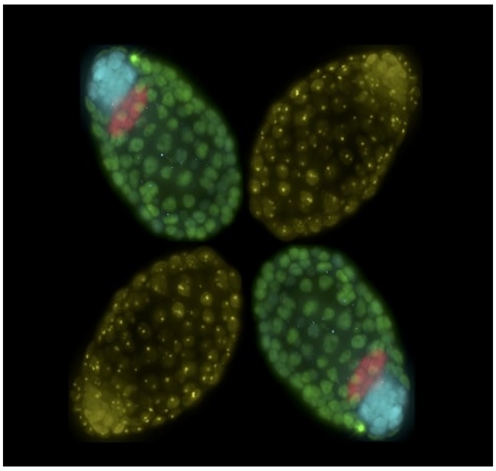

IHC - Wholemount - Anti-Histone H3 (tri methyl K27) antibody [mAbcam 6002] - ChIP Grade (ab6002)

In mice, the X-inactivation process is reversed naturally by X-reactivation in blastocysts and germ cells and in culture in pluripotent stem cells. The image shows late blastocyst-stage mouse embryos consisting of three cell types: epiblast (NANOG-positive, cyan), primitive endoderm (GATA4-positive, red) and trophectoderm (CDX2-positive, green). The inactive X-chromosome (H3K27me3-positive, yellow dots) is reactivated only in the epiblast (cells without yellow spots), which will form the embryo. The germ cell factor PRDM14 and the long noncoding RNA Tsix collaborate during the X-reactivation process in blastocysts and pluripotent stem cells and thereby link epigenetic with cellular reprogramming events.

Image is courtesy of Bernhard Payer, runner-up of the immunofluorescence imaging competition 2017.

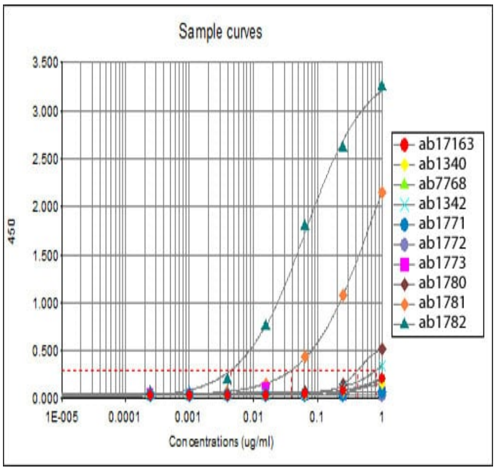


ELISA - Anti-Histone H3 (tri methyl K27) antibody [mAbcam 6002] - ChIP Grade (ab6002)

All batches of ab6002 are tested in ELISA against peptides to different Histone H3 modifications. Results show strong binding to Histone H3 - tri methyl K27 peptide (ab1782), indicating that this antibody specifically recognizes the Histone H3 tri methyl K27 modification. Weak binding is also detected against the Histone H3 di methyl K27 modification (<12%) (ab1781).

ab17163 - Histone H3 - unmodified

ab1340 - Histone H3 - mono methyl K4

ab7768 - Histone H3 - di methyl K4

ab1342 - Histone H3 - tri methyl K4

ab1771 - Histone H3 - mono methyl K9

ab1772 - Histone H3 - di methyl K9

ab1773 - Histone H3 - tri methyl K9

ab1780 - Histone H3 - mono methyl K27

ab1781 - Histone H3 - di methyl K27

ab1782 - Histone H3 - tri methyl K27

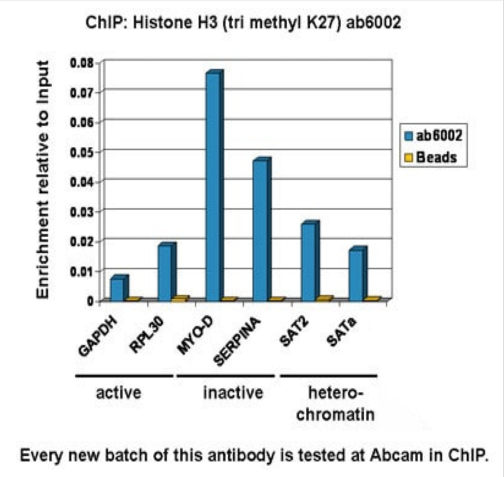


ChIP - Anti-Histone H3 (tri methyl K27) antibody [mAbcam 6002] - ChIP Grade (ab6002)

Chromatin was prepared from Hela cells according to the Abcam X-ChIP protocol. Cells were fixed with formaldehyde for 10 min. The ChIP was performed with 25 µg of chromatin, 5 µg of ab6002 (blue), and 20 µl of Protein A/G sepharose beads. No antibody was added to the beads control (yellow). The immunoprecipitated DNA was quantified by real time PCR (Taqman approach for active and inactive loci, Sybr green approach for heterochromatic loci). Primers and probes are located in the first kb of the transcribed region.

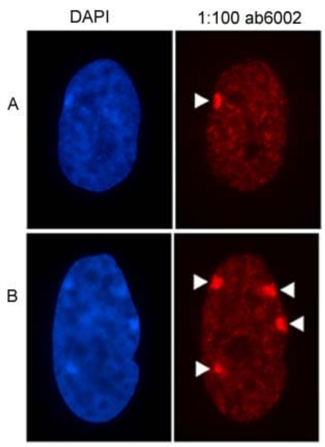


Immunocytochemistry/ Immunofluorescence - Anti-Histone H3 (tri methyl K27) antibody [mAbcam 6002] - ChIP Grade (ab6002)

Figure showing the nuclear distribution of H3 (tri-methyl K27) antibody, ab6002 in a) a 46 chromosome, XX cell line, and b) a 49 chromosome, XXXXX cell line.

The location of facultative heterochromatin at the inactive X chromosome is indicated by white arrow heads.

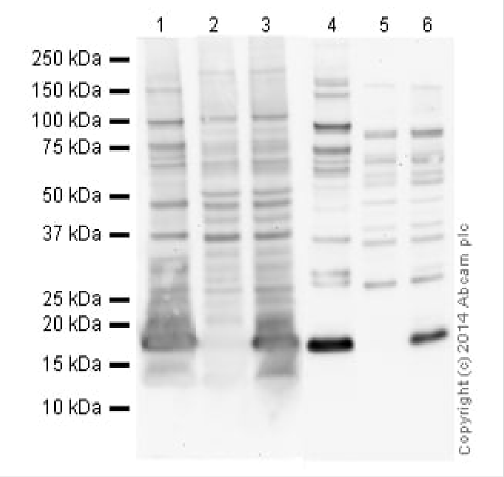


Western blot - Anti-Histone H3 (tri methyl K27) antibody [mAbcam 6002] - ChIP Grade (ab6002)

**Lanes 1-3 :** Anti-Histone H3 (tri methyl K27) antibody [mAbcam 6002] - ChIP Grade (ab6002) at 1 µg/ml (2% BSA)
**Lanes 4-6 :** Anti-Histone H3 (tri methyl K27) antibody [mAbcam 6002] - ChIP Grade (ab6002) at 1 µg/ml (3% MILK)

**Lanes 1 & 4 :** HeLa (Human epithelial carcinoma cell line) Nuclear Lysate
**Lanes 2 & 5 :** EED-/- mouse ES Whole Cell Lysate
**Lanes 3 & 6 :** WT mouse ES Whole Cell Lysate

Lysates/proteins at 10 µg per lane.

**Secondary**
**All lanes :** Goat Anti-Mouse IgG H&L (HRP) preadsorbed (ab97040) at 1/50000 dilution

Developed using the ECL technique.

Performed under reducing conditions.

**Predicted band size:** 15 kDa
**Observed band size:** 17 kDa

**Exposure time:** 12 minutes

This blot was produced using a 4-12% Bis-tris gel under the MES buffer system. The gel was run at 200V for 35 minutes before being transferred onto a Nitrocellulose membrane at 30V for 70 minutes. The membrane was then blocked for an hour using 2% Bovine Serum Albumin (lanes 1-3) and 3% milk (lanes 4-6) before being incubated with ab6002 overnight at 4°C. Antibody binding was detected using an anti-mouse antibody conjugated to HRP, and visualised using ECL development solution ab133406.

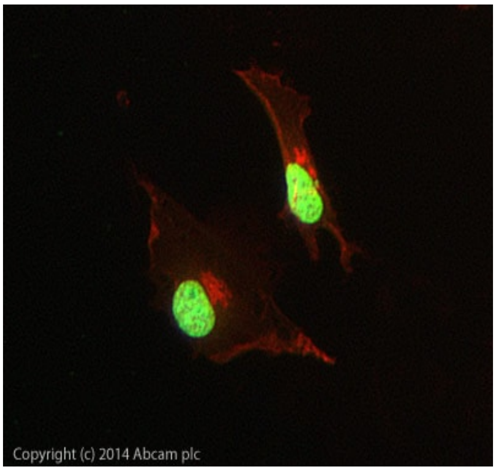


Immunocytochemistry/ Immunofluorescence - Anti-Histone H3 (tri methyl K27) antibody [mAbcam 6002] - ChIP Grade (ab6002)

ICC/IF image of ab6002 stained HeLa cells. The cells were 4% PFA fixed (10 min) and then incubated in 1%BSA / 10% normal goat serum / 0.3M glycine in 0.1% PBS-Tween for 1h to permeabilise the cells and block non-specific protein-protein interactions. The cells were then incubated with the antibody (ab6002, 5µg/ml) overnight at +4°C. The secondary antibody (green) was Alexa Fluor® 488 goat anti-mouse IgG (H+L) used at a 1/1000 dilution for 1h. Alexa Fluor® 594 WGA was used to label plasma membranes (red) at a 1/200 dilution for 1h. DAPI was used to stain the cell nuclei (blue) at a concentration of 1.43µM. This antibody also gave a positive result in 4% PFA fixed (10 min) Hek293, HepG2 and MCF7 cells at 5µg/ml, and in 100% methanol fixed (5 min) HeLa, Hek293, HepG2 and MCF7 cells at 5µg/ml.

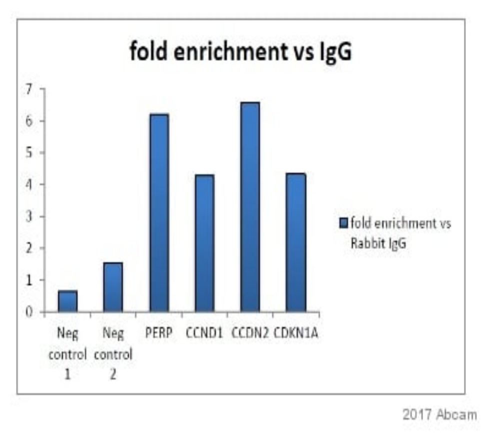


ChIP - Anti-Histone H3 (tri methyl K27) antibody [mAbcam 6002] - ChIP Grade (ab6002)

This image is courtesy of an anonymous abreview.

Chromatin was prepared from K562 cells. Cells were fixed with formaldehyde for 10 min. A GATA1 antibody was used as the positive control and a Rabbit IgG was used as the negative control. Incubation with primary antibody was in Immuno Precipitation Dilution buffer for 16 hours at 4°C. The immunoprecipitated DNA was quantified by real time PCR.

**Please note:** All products are "FOR RESEARCH USE ONLY. NOT FOR USE IN DIAGNOSTIC PROCEDURES"

## Our Abpromise to you: Quality guaranteed and expert technical support

- Replacement or refund for products not performing as stated on the datasheet

- Valid for 12 months from date of delivery
- Response to your inquiry within 24 hours

- We provide support in Chinese, English, French, German, Japanese and Spanish
- Extensive multi-media technical resources to help you
- We investigate all quality concerns to ensure our products perform to the highest standards

If the product does not perform as described on this datasheet, we will offer a refund or replacement. For full details of the Abpromise, please visit https://www.abcam.cn/abpromise or contact our technical team.

## Terms and conditions

- Guarantee only valid for products bought direct from Abcam or one of our authorized distributors

abcam

# Product datasheet

# Anti-GAPDH antibody [6C5] - Loading Control ab8245

★★★★★ 100 Abreviews   3486 References   6 图像

## 概述

| | |
|---|---|
| **产品名称** | Anti-GAPDH抗体[6C5] - Loading Control |
| **描述** | 小鼠单克隆抗体[6C5] to GAPDH - Loading Control |
| **宿主** | Mouse |
| **特异性** | This GAPDH antibody can be used as a loading control antibody. GAPDH is a 146 kDa tetramer composed of four 30-40 kDa subunits. There is no cross-reaction with GAPDH from yeast. Preliminary data indicates that the GAPDH antibody- loading control ab8245 recognizes the monomer (36 kDa) and also the dimer forms of GAPDH, but not the tetrameric form of the protein. |
| **经测试应用** | **适用于:** WB, ICC/IF |
| **种属反应性** | **与反应:** Mouse, Rat, Human<br>**预测可用于:** Horse, Chicken, Guinea pig, Hamster, Cat, Dog, Pig, Xenopus laevis, Fish, Monkey, Zebrafish, Baboon, Xenopus tropicalis **不与反应:** Goat, Cow, Saccharomyces cerevisiae |
| **免疫原** | Full length native protein (purified) corresponding to GAPDH.<br>Database link: P46406 |
| **阳性对照** | ICC/IF: HeLa cells, NIH3T3 cells, SV40LT-SMC cells. WB: HeLa, A431, Jurkat, HEK-293, Raji whole cell lysate. |
| **常规说明** | This product switched from ascites to tissue culture supernatant on 31 July 2017. Lot numbers higher than [GR291713] will be from tissue culture supernatant.<br><br>The Life Science industry has been in the grips of a reproducibility crisis for a number of years. Abcam is leading the way in addressing this with our range of recombinant monoclonal antibodies and knockout edited cell lines for gold-standard validation. Please check that this product meets your needs before purchasing.<br><br>If you have any questions, special requirements or concerns, please send us an inquiry and/or contact our Support team ahead of purchase. Recommended alternatives for this product can be found below, along with publications, customer reviews and Q&As |

## 性能

| | |
|---|---|
| **形式** | Liquid |
| **存放说明** | Shipped at 4°C. Store at +4°C short term (1-2 weeks). Upon delivery aliquot. Store at -20°C or -80°C. Avoid freeze / thaw cycle. |

| | |
|---|---|
| **存储溶液** | pH: 7.40<br>Preservative: 0.09% Sodium azide<br>Constituent: PBS |
| **纯度** | Protein A purified |
| **纯化说明** | Chromatography on protein A Sepharose |
| **克隆** | 单克隆 |
| **克隆编号** | 6C5 |
| **骨髓瘤** | Sp2/0 |
| **同种型** | IgG1 |

## 应用

**The Abpromise guarantee** Abpromise™承诺保证使用ab8245于以下的经测试应用

"应用说明"部分 下显示的仅为推荐的起始稀释度；实际最佳的稀释度/浓度应由使用者检定。

| 应用 | Ab评论 | 说明 |
|---|---|---|
| **WB** | ★★★★★ (96) | 1/500 - 1/10000. Detects a band of approximately 36 kDa (predicted molecular weight: 40.2 kDa). |
| **ICC/IF** | ★★★★★ (1) | Use a concentration of 1 - 5 µg/ml. |

## 靶标

| | |
|---|---|
| **功能** | Has both glyceraldehyde-3-phosphate dehydrogenase and nitrosylase activities, thereby playing a role in glycolysis and nuclear functions, respectively. Participates in nuclear events including transcription, RNA transport, DNA replication and apoptosis. Nuclear functions are probably due to the nitrosylase activity that mediates cysteine S-nitrosylation of nuclear target proteins such as SIRT1, HDAC2 and PRKDC (By similarity). Glyceraldehyde-3-phosphate dehydrogenase is a key enzyme in glycolysis that catalyzes the first step of the pathway by converting D-glyceraldehyde 3-phosphate (G3P) into 3-phospho-D-glyceroyl phosphate. |
| **通路** | Carbohydrate degradation; glycolysis; pyruvate from D-glyceraldehyde 3-phosphate: step 1/5. |
| **序列相似性** | Belongs to the glyceraldehyde-3-phosphate dehydrogenase family. |
| **翻译后修饰** | S-nitrosylation of Cys-152 leads to interaction with SIAH1, followed by translocation to the nucleus.<br>ISGylated. |
| **细胞定位** | Cytoplasm > cytosol. Nucleus. Cytoplasm > perinuclear region. Membrane. Translocates to the nucleus following S-nitrosylation and interaction with SIAH1, which contains a nuclear localization signal (By similarity). Postnuclear and Perinuclear regions. |

## 图片

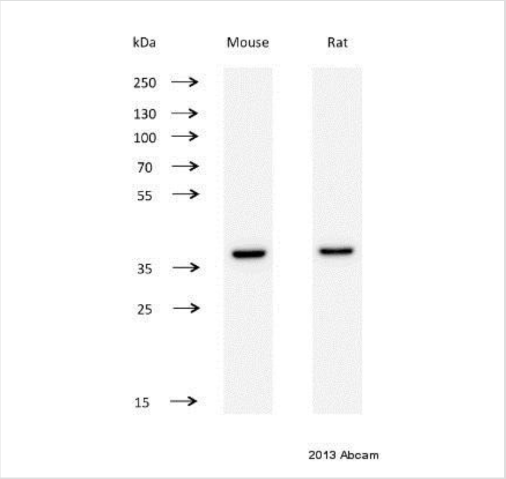


Western blot - Anti-GAPDH antibody [6C5] - Loading Control (ab8245)

This image is courtesy of an anonymous Abreview

**All lanes :** Anti-GAPDH antibody [6C5] - Loading Control (ab8245)

**Lane 1 :** Mouse hippocampus whole cell lysate

**Lane 2 :** Rat hippocampus whole cell lysate

Lysates/proteins at 20 µg per lane.

**Secondary**

**All lanes :** HRP-conjugated Rabbit anti-mouse at 1/5000 dilution

Developed using the ECL technique.

Performed under reducing conditions.

**Predicted band size:** 40.2 kDa

**Observed band size:** 36 kDa

**Exposure time:** 10 seconds

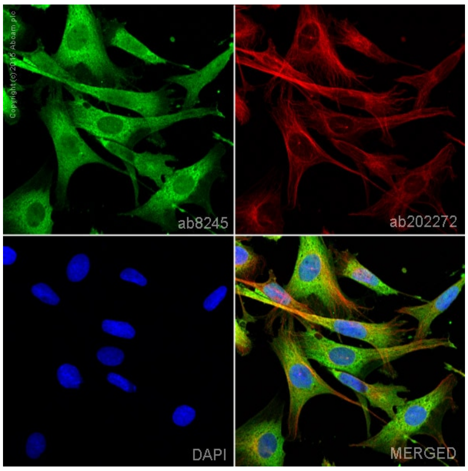


Immunocytochemistry/ Immunofluorescence - Anti-GAPDH antibody [6C5] - Loading Control (ab8245)

ab8245 staining GAPDH in SV40LT-SMC (Rat SV40-transfected aorta smooth cell line) cells.

The cells were fixed with 4% formaldehyde (10 minutes), permeabilized with 0.1% Triton X-100 for 5 minutes and then blocked in 1% BSA/10% normal goat serum/0.3M glycine in 0.1%PBS-Tween for 1 hour. The cells were then incubated with ab8245 at 5µg/ml and ab202272 at 1/250 overnight at +4°C, followed by a further incubation at room temperature for 1h with Goat Anti-Mouse IgG H&L (Alexa Fluor® 488) preadsorbed (ab150117) (shown in green). Nuclear DNA was labeled in blue with DAPI.

Image was taken with a confocal microscope (Leica-Microsystems, TCS SP8).

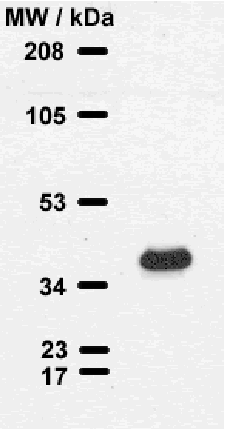


Western blot - Anti-GAPDH antibody [6C5] - Loading Control (ab8245)

Anti-GAPDH antibody [6C5] - Loading Control (ab8245) at 10 µg/ml + Raji (Human Burkitt's lymphoma cell line) whole cell lysate at 20 µg

**Predicted band size:** 40.2 kDa

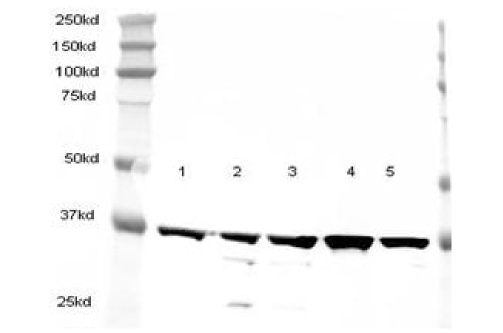


Western blot - Anti-GAPDH antibody [6C5] - Loading Control (ab8245)

**All lanes :** Anti-GAPDH antibody [6C5] - Loading Control (ab8245) at 2.5 µg/ml

**Lane 1 :** HeLa (Human epithelial cell line from cervix adenocarcinoma) Nuclear
**Lane 2 :** HeLa (Human epithelial cell line from cervix adenocarcinoma) whole cell lysate
**Lane 3 :** A431 (Human epidermoid carcinoma cell line) cell lysate
**Lane 4 :** Jurkat (Human T cell leukemia cell line from peripheral blood) cell lysate
**Lane 5 :** HEK-293 (Human epithelial cell line from embryonic kidney) cell lysate

Lysates/proteins at 20 µg per lane.

**Secondary**
**All lanes :** Alexa Fluor anti-mouse at 1/5000 dilution

Performed under reducing conditions.

**Predicted band size:** 40.2 kDa
**Observed band size:** 37 kDa

Fluorescence detection of secondary antibody.

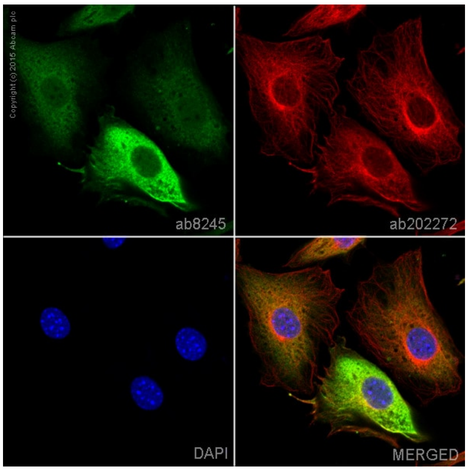


Immunocytochemistry/ Immunofluorescence - Anti-GAPDH antibody [6C5] - Loading Control (ab8245)

ab8245 staining GAPDH in NIH/3T3 (Mouse embryo fibroblast cell line) cells.

The cells were fixed with 4% formaldehyde (10 minutes) and then blocked in 1% BSA/10% normal goat serum/0.3M glycine in 0.1%PBS-Tween for 1 hour. The cells were then incubated with ab8245 at 1 µg/ml and ab202272 at 1/250 overnight at +4°C, followed by a further incubation at room temperature for 1 hour with Goat Anti-Mouse IgG H&L (Alexa Fluor® 488) preadsorbed (ab150117) (shown in green). Nuclear DNA was labeled in blue with DAPI.

Image was taken with a confocal microscope (Leica-Microsystems, TCS SP8).

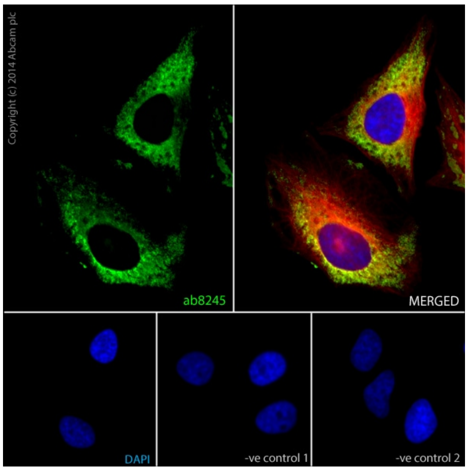


Immunocytochemistry/ Immunofluorescence - Anti-GAPDH antibody [6C5] - Loading Control (ab8245)

ab8245 staining GAPDH in HeLa (Human epithelial cell line from cervix adenocarcinoma) cells.

The cells were fixed with 100% methanol (5 minutes) and then blocked in 1% BSA/10% normal goat serum/0.3M glycine in 0.1%PBS-Tween for 1 hour. The cells were then incubated with ab8245 at 5 µg/ml and ab6046 at 1 µg/ml overnight at +4°C, followed by a further incubation at room temperature for 1 hour with Goat Anti-Mouse IgG H&L (Alexa Fluor® 488) preadsorbed (ab150117) at 2 µg/ml (shown in green) and Goat Anti-Rabbit IgG H&L (Alexa Fluor® 594) preadsorbed (ab150088) at 2 µg/ml (shown in pseudo color red). Nuclear DNA was labeled in blue with DAPI.

**Negative controls:** 1– Rabbit primary antibody and anti-mouse secondary antibody; 2 – Mouse primary antibody and anti-rabbit secondary antibody. Controls 1 and 2 indicate that there is no unspecific reaction between primary and secondary antibodies used.

**Please note:** All products are "FOR RESEARCH USE ONLY. NOT FOR USE IN DIAGNOSTIC PROCEDURES"

## Our Abpromise to you: Quality guaranteed and expert technical support

- Replacement or refund for products not performing as stated on the datasheet
- Valid for 12 months from date of delivery

- Response to your inquiry within 24 hours
- We provide support in Chinese, English, French, German, Japanese and Spanish
- Extensive multi-media technical resources to help you
- We investigate all quality concerns to ensure our products perform to the highest standards

If the product does not perform as described on this datasheet, we will offer a refund or replacement. For full details of the Abpromise, please visit https://www.abcam.cn/abpromise or contact our technical team.

## Terms and conditions

- Guarantee only valid for products bought direct from Abcam or one of our authorized distributors

abcam

# Product datasheet

# Anti-Histone H3 (tri methyl K4) antibody - ChIP Grade ab8580

★★★★★ 88 Abreviews 1741 References 9 图像

## 概述

| | |
|---|---|
| **产品名称** | Anti-Histone H3 (tri methyl K4)**抗体**- ChIP Grade |
| **描述** | **兔多克隆抗体**to Histone H3 (tri methyl K4) - ChIP Grade |
| **宿主** | Rabbit |
| **经测试应用** | **适用于:** PepArr, ChIP, WB, IHC-P, ICC/IF |
| **种属反应性** | **与反应:** Cow, Human<br>**预测可用于:** Mouse, Rat, Rabbit, Pig, Saccharomyces cerevisiae, Tetrahymena, Xenopus laevis, Arabidopsis thaliana, Caenorhabditis elegans, Drosophila melanogaster, Indian muntjac, Oikopleura, Plants, Zebrafish, Mammals, Trypanosoma cruzi, Common marmoset, Rice, Xenopus tropicalis |
| **免疫原** | Synthetic peptide within Human Histone H3 aa 1-100 (tri methyl K4) conjugated to keyhole limpet haemocyanin. The exact sequence is proprietary.<br>(Peptide available as ab92374) |
| **常规说明** | In immunofluorescence, a distinct property of tri methyl lysine 4 is its apparent 'ringing' of regions that appear as nucleoplasmic 'holes'. These represent the positions of splicing factor compartments, which often are easy to identify using only DNA stains in Indian muntjac fibroblasts. These splicing factor compartments are known to be preferentially associated with active genes and highly acetylated histone H3. This antibody, as expected, fails to stain heterochromatin (work by Kirk McManus, lab of Michael Hendzel).<br>The immunofluorescence results suggest this antibody is **an exceptional euchromatin probe**.<br><br>Learn about ChIP assay kits, other ChIP antibodies, protocols and more in the ChIP assay guide.<br>Abcam recommended secondaries - Goat Anti-Rabbit HRP (ab205718) and Goat Anti-Rabbit Alexa Fluor® 488 (ab150077). See other anti-rabbit secondary antibodies that can be used with this antibody.<br>The Life Science industry has been in the grips of a reproducibility crisis for a number of years. Abcam is leading the way in addressing this with our range of recombinant monoclonal antibodies and knockout edited cell lines for gold-standard validation. Please check that this product meets your needs before purchasing.<br>If you have any questions, special requirements or concerns, please send us an inquiry and/or contact our Support team ahead of purchase. Recommended alternatives for this product can be found below, along with publications, customer reviews and Q&As |

## 性能

| | |
|---|---|
| **形式** | Liquid |
| **存放说明** | Shipped at 4°C. Store at +4°C short term (1-2 weeks). Upon delivery aliquot. Store at -20°C or -80°C. Avoid freeze / thaw cycle. |
| **存储溶液** | pH: 7.40<br>Preservative: 0.02% Sodium azide<br>Constituent: PBS<br><br>Batches of this product that have a concentration < 1mg/ml may have BSA added as a stabilising agent. If you would like information about the formulation of a specific lot, please contact our scientific support team who will be happy to help. |
| **纯度** | Immunogen affinity purified |
| **克隆** | 多克隆 |
| **同种型** | IgG |

## 应用

**The Abpromise guarantee** Abpromise™承诺保证使用ab8580于以下的经测试应用

“应用说明”部分 下显示的仅为推荐的起始稀释度；实际最佳的稀释度/浓度应由使用者检定。

| 应用 | Ab评论 | 说明 |
|---|---|---|
| **PepArr** | | Use a concentration of 0.2 - 0.02 µg/ml.<br>Slight cross reactivity is observed with the Histone H3 - di methyl K4 modification. Optimisation is recommended to avoid array signal saturation. |
| **ChIP** | ★★★★★ (27) | Use 2 µg for 25 µg of chromatin.<br>We recommend GAPDH positive control ChIP primer pair ab267832 as positive control. |
| **WB** | ★★★★★ (20) | Use a concentration of 1 µg/ml. Detects a band of approximately 17 kDa (predicted molecular weight: 15 kDa). |
| **IHC-P** | ★★★★★ (8) | Use at an assay dependent concentration. |
| **ICC/IF** | ★★★★★ (23) | Use a concentration of 1 µg/ml. 1/100 - 1/5000 |

## 靶标

| | |
|---|---|
| **功能** | Core component of nucleosome. Nucleosomes wrap and compact DNA into chromatin, limiting DNA accessibility to the cellular machineries which require DNA as a template. Histones thereby play a central role in transcription regulation, DNA repair, DNA replication and chromosomal stability. DNA accessibility is regulated via a complex set of post-translational modifications of histones, also called histone code, and nucleosome remodeling. |
| **序列相似性** | Belongs to the histone H3 family. |
| **发展阶段** | Expressed during S phase, then expression strongly decreases as cell division slows down |

| | |
|---|---|
| | during the process of differentiation. |
| **翻译后修饰** | Acetylation is generally linked to gene activation. Acetylation on Lys-10 (H3K9ac) impairs methylation at Arg-9 (H3R8me2s). Acetylation on Lys-19 (H3K18ac) and Lys-24 (H3K24ac) favors methylation at Arg-18 (H3R17me).<br>Citrullination at Arg-9 (H3R8ci) and/or Arg-18 (H3R17ci) by PADI4 impairs methylation and represses transcription.<br>Asymmetric dimethylation at Arg-18 (H3R17me2a) by CARM1 is linked to gene activation. Symmetric dimethylation at Arg-9 (H3R8me2s) by PRMT5 is linked to gene repression. Asymmetric dimethylation at Arg-3 (H3R2me2a) by PRMT6 is linked to gene repression and is mutually exclusive with H3 Lys-5 methylation (H3K4me2 and H3K4me3). H3R2me2a is present at the 3' of genes regardless of their transcription state and is enriched on inactive promoters, while it is absent on active promoters.<br>Methylation at Lys-5 (H3K4me), Lys-37 (H3K36me) and Lys-80 (H3K79me) are linked to gene activation. Methylation at Lys-5 (H3K4me) facilitates subsequent acetylation of H3 and H4. Methylation at Lys-80 (H3K79me) is associated with DNA double-strand break (DSB) responses and is a specific target for TP53BP1. Methylation at Lys-10 (H3K9me) and Lys-28 (H3K27me) are linked to gene repression. Methylation at Lys-10 (H3K9me) is a specific target for HP1 proteins (CBX1, CBX3 and CBX5) and prevents subsequent phosphorylation at Ser-11 (H3S10ph) and acetylation of H3 and H4. Methylation at Lys-5 (H3K4me) and Lys-80 (H3K79me) require preliminary monoubiquitination of H2B at 'Lys-120'. Methylation at Lys-10 (H3K9me) and Lys-28 (H3K27me) are enriched in inactive X chromosome chromatin.<br>Phosphorylated at Thr-4 (H3T3ph) by GSG2/haspin during prophase and dephosphorylated during anaphase. Phosphorylation at Ser-11 (H3S10ph) by AURKB is crucial for chromosome condensation and cell-cycle progression during mitosis and meiosis. In addition phosphorylation at Ser-11 (H3S10ph) by RPS6KA4 and RPS6KA5 is important during interphase because it enables the transcription of genes following external stimulation, like mitogens, stress, growth factors or UV irradiation and result in the activation of genes, such as c-fos and c-jun. Phosphorylation at Ser-11 (H3S10ph), which is linked to gene activation, prevents methylation at Lys-10 (H3K9me) but facilitates acetylation of H3 and H4. Phosphorylation at Ser-11 (H3S10ph) by AURKB mediates the dissociation of HP1 proteins (CBX1, CBX3 and CBX5) from heterochromatin. Phosphorylation at Ser-11 (H3S10ph) is also an essential regulatory mechanism for neoplastic cell transformation. Phosphorylated at Ser-29 (H3S28ph) by MLTK isoform 1, RPS6KA5 or AURKB during mitosis or upon ultraviolet B irradiation. Phosphorylation at Thr-7 (H3T6ph) by PRKCBB is a specific tag for epigenetic transcriptional activation that prevents demethylation of Lys-5 (H3K4me) by LSD1/KDM1A. At centromeres, specifically phosphorylated at Thr-12 (H3T11ph) from prophase to early anaphase, by DAPK3 and PKN1. Phosphorylation at Thr-12 (H3T11ph) by PKN1 is a specific tag for epigenetic transcriptional activation that promotes demethylation of Lys-10 (H3K9me) by KDM4C/JMJD2C. Phosphorylation at Tyr-42 (H3Y41ph) by JAK2 promotes exclusion of CBX5 (HP1 alpha) from chromatin.<br>Monoubiquitinated by RAG1 in lymphoid cells, monoubiquitination is required for V(D)J recombination (By similarity). Ubiquitinated by the CUL4-DDB-RBX1 complex in response to ultraviolet irradiation. This may weaken the interaction between histones and DNA and facilitate DNA accessibility to repair proteins. |
| **细胞定位** | Nucleus. Chromosome. |

## 图片

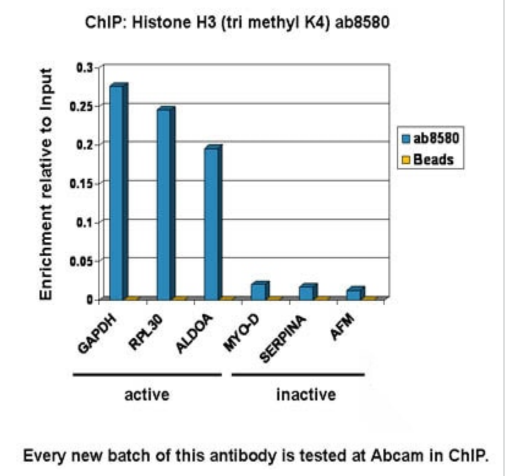


ChIP - Anti-Histone H3 (tri methyl K4) antibody - ChIP Grade (ab8580)

Chromatin was prepared from U-2 OS (Human bone osteosarcoma epithelial cell line) cells according to the Abcam X-ChIP protocol.

Cells were fixed with formaldehyde for 10 minutes. The ChIP was performed with 25 µg of chromatin, 2 µg of ab8580 (blue), and 20 µl of Protein A/G sepharose beads. No antibody was added to the beads control (yellow). The immunoprecipitated DNA was quantified by real time PCR (Taqman approach). Primers and probes are located in the first kb of the transcribed region.

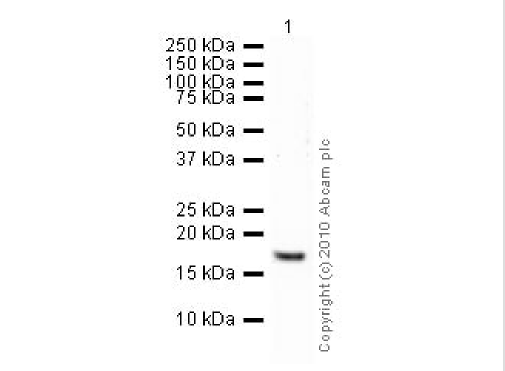


Western blot - Anti-Histone H3 (tri methyl K4) antibody - ChIP Grade (ab8580)

Anti-Histone H3 (tri methyl K4) antibody - ChIP Grade (ab8580) at 1 µg/ml + Calf thymus histone preparation (nuclear lysate) at 0.5 µg

**Secondary**

Goat Anti-Rabbit IgG (H+L) HRP- conjugated antibody at 1/50000 dilution

Performed under reducing conditions.

**Predicted band size:** 15 kDa

**Observed band size:** 17 kDa

**Exposure time:** 8 minutes

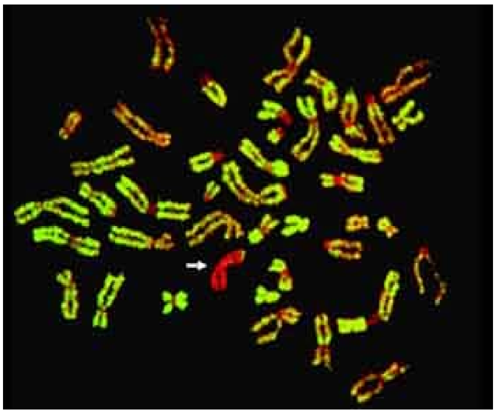

Immunocytochemistry/ Immunofluorescence - Anti-Histone H3 (tri methyl K4) antibody - ChIP Grade (ab8580)

This image is courtesy of Ahmad Khalil and Daniel Driscoll, University of Florida College of Medicine.

Human female lymphoblast immunostained with ab8580 (1:100) (yellowish green) specific for histone H3 lysine 4 (H3-K4) trimethylation; the DNA is stained red with propidium iodide (PI).

Note the inactive X chromosome (arrow) and pericentromeric heterochromatin are largely devoid of this modification.

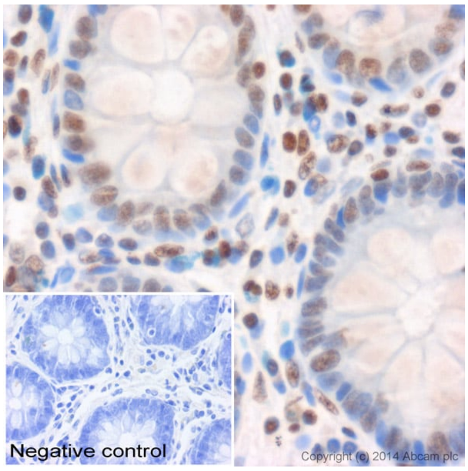


Immunohistochemistry (Formalin/PFA-fixed paraffin-embedded sections) - Anti-Histone H3 (tri methyl K4) antibody - ChIP Grade (ab8580)

IHC image of ab8580 staining Histone H3 (tri methyl K4) in human colon formalin-fixed paraffin-embedded tissue sections*, performed on a Leica Bond.

The section was pre-treated using heat mediated antigen retrieval with sodium citrate buffer (pH 6, epitope retrieval solution 1) for 20 minutes. The section was then incubated with ab8580, 1/500 dilution, for 15 minutes at room temperature and detected using an HRP conjugated compact polymer system. DAB was used as the chromogen. The section was then counterstained with haematoxylin and mounted with DPX.

No primary antibody was used in the negative control (inset).

For other IHC staining systems (automated and non-automated) customers should optimize variable parameters such as antigen retrieval conditions, primary antibody concentration and antibody incubation times.

*Tissue obtained from the Human Research Tissue Bank, supported by the NIHR Cambridge Biomedical Research Centre

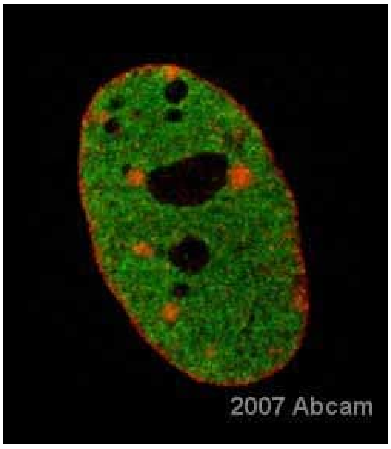


Immunocytochemistry/ Immunofluorescence - Anti-Histone H3 (tri methyl K4) antibody - ChIP Grade (ab8580)

This image is courtesy of an Abreview submitted by Dr Eva Bartova

ab8580 staining cultured human primary fibroblasts by ICC.

Cells were fixed in PFA and permeabilized in Triton X-100 and saponin prior to blocking with 1% BSA for 1 hour at RT. The primary antibody was diluted 1/100 and incubated with the sample for 16 hours at 4°C. An FITC-conjugated rabbit anti-rabbit IgG antibody was used as the secondary.

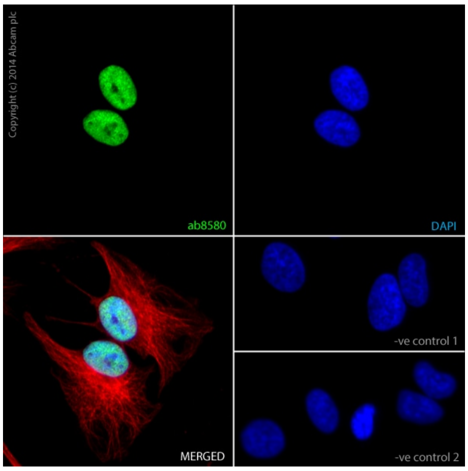


Immunocytochemistry/ Immunofluorescence - Anti-Histone H3 (tri methyl K4) antibody - ChIP Grade (ab8580)

ab8580 staining Histone H3 (tri methyl K4) in HeLa (Human epithelial cell line from cervix adenocarcinoma) cells.

All cells were fixed with 100% methanol (5 minutes) and then blocked in 1% BSA/10% normal goat serum/0.3M glycine in 0.1%PBS-Tween for 1 hour. The cells were then incubated with ab4729 at 1/1000 and ab7291 at 1µg/ml overnight at +4°C, followed by a further incubation at room temperature for 1 hour with goat anti-rabbit Alexa-Fluor®488 secondary (ab150077) at 2 µg/ml (shown in green) and goat anti-mouse Alexa-Fluor®594 secondary (ab150120) at 2 µg/ml (shown in pseudo color red). Nuclear DNA was labeled in blue with DAPI.

**Negative controls:** 1– Rabbit primary and anti-mouse secondary antibody; 2 – Mouse primary antibody and anti-rabbit secondary antibody. Controls 1 and 2 indicate that there is no non-specific reaction between primary and secondary antibodies used.

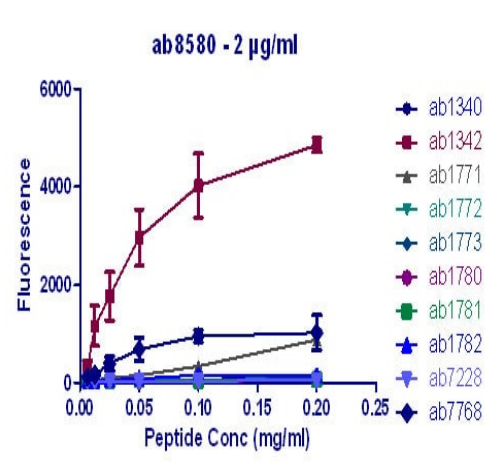


Peptide Array - Anti-Histone H3 (tri methyl K4) antibody - ChIP Grade (ab8580)

All batches of ab8580 are tested in Peptide Array against peptides to different Histone H3 modifications. Six dilutions of each peptide are printed on to the Peptide Array in triplicate and results are averaged before being plotted on to a graph. Results show strong binding to Histone H3 - tri methyl K4 peptide (ab1342), indicating that this antibody specifically recognises the Histone H3 - tri methyl K4 modification. Slight cross reactivity is observed with the Histone H3 - di methyl K4 modification. Optimization is recommended to avoid array signal saturation.

ab1340 - Histone H3 - mono methyl K4

ab1342 - Histone H3 - tri methyl K4

ab1771 - Histone H3 - mono methyl K9

ab1772 - Histone H3 - di methyl K9

ab1773 - Histone H3 - tri methyl K9

ab1780 - Histone H3 - mono methyl K27

ab1781 - Histone H3 - di methyl K27

ab1782 - Histone H3 - tri methyl K27

ab7228 - Histone H3 - unmodified

ab7768 - Histone H3 - di methyl K4

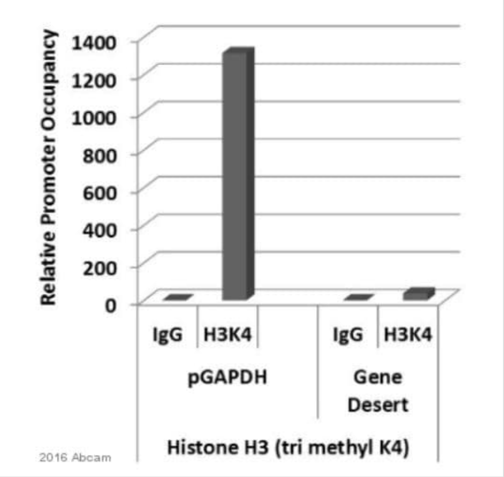


ChIP - Anti-Histone H3 (tri methyl K4) antibody - ChIP Grade (ab8580)

This image is courtesy of an anonymous Abreview

Chromatin was prepared from human cell lysate - nuclear B cells according to the Abcam X-ChIP protocol.

Cells were fixed with formaldehyde for 10 minutes. The ChIP was performed with 0.5 µg of ab8580 per µg chromatin in ChIP Buffer fot 16 hours at 4°C. The immunoprecipitated DNA was quantified by real time PCR (Taqman approach). Primers and probes are located in the first kb of the transcribed region. Negative control: IgG and Gene Desert. Positive control: GAPDH Promoter.

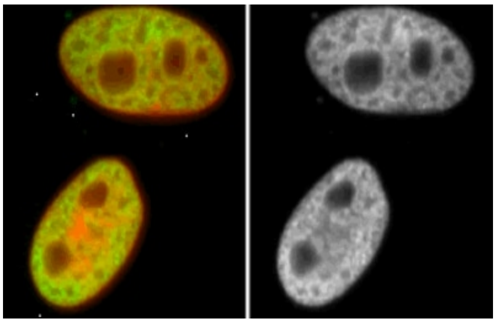

Immunocytochemistry/ Immunofluorescence - Anti-Histone H3 (tri methyl K4) antibody - ChIP Grade (ab8580)

This image is courtesy of Kirk McManus in the lab of Michael Hendzel, University of Alberta

Staining (green) with the anti-trimethyl Lysine K4 of Histone H3 antibody (ab8580) shows ringing of regions that appear as nucleoplasmic holes. These represent the positions of splicing factor compartments, which are preferentially associated with active genes and highly acetylated histone H3.

The antibody, as expected, fails to stain heterochromatin (red).

**Please note:** All products are "FOR RESEARCH USE ONLY. NOT FOR USE IN DIAGNOSTIC PROCEDURES"

## Our Abpromise to you: Quality guaranteed and expert technical support

- Replacement or refund for products not performing as stated on the datasheet
- Valid for 12 months from date of delivery
- Response to your inquiry within 24 hours

- We provide support in Chinese, English, French, German, Japanese and Spanish
- Extensive multi-media technical resources to help you
- We investigate all quality concerns to ensure our products perform to the highest standards

If the product does not perform as described on this datasheet, we will offer a refund or replacement. For full details of the Abpromise, please visit https://www.abcam.cn/abpromise or contact our technical team.

## Terms and conditions

- Guarantee only valid for products bought direct from Abcam or one of our authorized distributors

abcam

## Product datasheet

# Anti-Histone H3 (tri methyl K9) antibody - ChIP Grade ab8898

★★★★★ 89 Abreviews 1406 References 5 图像

### 概述

| | |
|---|---|
| 产品名称 | Anti-Histone H3 (tri methyl K9)抗体- ChIP Grade |
| 描述 | 兔多克隆抗体to Histone H3 (tri methyl K9) - ChIP Grade |
| 宿主 | Rabbit |
| 特异性 | Histone H3 (tri methyl K9) antibody (ab8898) is specific for Histone H3 tri methyl Lysine 9. Shows slight cross-reactivity with tri methyl K27, which shares a similar epitope (please see Western blot image). Does not react with mono or di methylated K9. |
| 经测试应用 | **适用于:** WB, IHC-P, ICC, ChIP |
| 种属反应性 | **与反应:** Mouse, Cow, Human<br>**预测可用于:** Rat, Chicken, Saccharomyces cerevisiae, Xenopus laevis, Drosophila melanogaster, Indian muntjac, Mammals, Xenopus tropicalis, Cyanidioschyzon merolae |
| 免疫原 | Synthetic peptide. This information is proprietary to Abcam and/or its suppliers.<br>(Peptide available as ab1773) |
| 阳性对照 | ChIP: U2OS cells, mouse ES cells. WB: Calf Thymus Histone Preparation Nuclear Lysate. IHC-P: Normal human colon. ICC: Mouse 3T3MEF, Indian muntjac fibroblast cells, HeLa cells, Mouse Embryonic Stem cells. |
| 常规说明 | Every new batch of ab8898 is tested in house in ChIP. Learn about ChIP assay kits, other ChIP antibodies, protocols and more in the ChIP assay guide.<br><br>The Life Science industry has been in the grips of a reproducibility crisis for a number of years. Abcam is leading the way in addressing this with our range of recombinant monoclonal antibodies and knockout edited cell lines for gold-standard validation. Please check that this product meets your needs before purchasing.<br><br>If you have any questions, special requirements or concerns, please send us an inquiry and/or contact our Support team ahead of purchase. Recommended alternatives for this product can be found below, along with publications, customer reviews and Q&As |

### 性能

| | |
|---|---|
| 形式 | Liquid |
| 存放说明 | Shipped at 4°C. Store at +4°C short term (1-2 weeks). Upon delivery aliquot. Store at -20°C or -80°C. Avoid freeze / thaw cycle. |
| 存储溶液 | pH: 7.40 |

Preservative: 0.02% Sodium azide
Constituent: PBS

Batches of this product that have a concentration < 1mg/ml may have BSA added as a stabilising agent. If you would like information about the formulation of a specific lot, please contact our scientific support team who will be happy to help.

| | |
|---|---|
| **纯度** | Immunogen affinity purified |
| **克隆** | 多克隆 |
| **同种型** | IgG |

## 应用

**The Abpromise guarantee** Abpromise™承诺保证使用ab8898于以下的经测试应用

"应用说明"部分 下显示的仅为推荐的起始稀释度；实际最佳的稀释度/浓度应由使用者检定。

| 应用 | Ab评论 | 说明 |
|---|---|---|
| **WB** | ★★★★★ (29) | Use at an assay dependent concentration. Predicted molecular weight: 15 kDa.Can be blocked with Human Histone H3 (tri methyl K9) peptide (ab1773). |
| **IHC-P** | ★★★★★ (9) | 1/400. Perform heat mediated antigen retrieval before commencing with IHC staining protocol. |
| **ICC** | | Use a concentration of 0.5 µg/ml. |
| **ChIP** | ★★★★★ (25) | Use 2-4 µg for 25 µg of chromatin.<br>We recommend SAT-alpha ChIP primer pair ab269263 as a positive control. |

## 靶标

**功能**

Core component of nucleosome. Nucleosomes wrap and compact DNA into chromatin, limiting DNA accessibility to the cellular machineries which require DNA as a template. Histones thereby play a central role in transcription regulation, DNA repair, DNA replication and chromosomal stability. DNA accessibility is regulated via a complex set of post-translational modifications of histones, also called histone code, and nucleosome remodeling.

**序列相似性**

Belongs to the histone H3 family.

**发展阶段**

Expressed during S phase, then expression strongly decreases as cell division slows down during the process of differentiation.

**翻译后修饰**

Acetylation is generally linked to gene activation. Acetylation on Lys-10 (H3K9ac) impairs methylation at Arg-9 (H3R8me2s). Acetylation on Lys-19 (H3K18ac) and Lys-24 (H3K24ac) favors methylation at Arg-18 (H3R17me).
Citrullination at Arg-9 (H3R8ci) and/or Arg-18 (H3R17ci) by PADI4 impairs methylation and represses transcription.
Asymmetric dimethylation at Arg-18 (H3R17me2a) by CARM1 is linked to gene activation.
Symmetric dimethylation at Arg-9 (H3R8me2s) by PRMT5 is linked to gene repression.
Asymmetric dimethylation at Arg-3 (H3R2me2a) by PRMT6 is linked to gene repression and is

mutually exclusive with H3 Lys-5 methylation (H3K4me2 and H3K4me3). H3R2me2a is present at the 3' of genes regardless of their transcription state and is enriched on inactive promoters, while it is absent on active promoters.
Methylation at Lys-5 (H3K4me), Lys-37 (H3K36me) and Lys-80 (H3K79me) are linked to gene activation. Methylation at Lys-5 (H3K4me) facilitates subsequent acetylation of H3 and H4. Methylation at Lys-80 (H3K79me) is associated with DNA double-strand break (DSB) responses and is a specific target for TP53BP1. Methylation at Lys-10 (H3K9me) and Lys-28 (H3K27me) are linked to gene repression. Methylation at Lys-10 (H3K9me) is a specific target for HP1 proteins (CBX1, CBX3 and CBX5) and prevents subsequent phosphorylation at Ser-11 (H3S10ph) and acetylation of H3 and H4. Methylation at Lys-5 (H3K4me) and Lys-80 (H3K79me) require preliminary monoubiquitination of H2B at 'Lys-120'. Methylation at Lys-10 (H3K9me) and Lys-28 (H3K27me) are enriched in inactive X chromosome chromatin.
Phosphorylated at Thr-4 (H3T3ph) by GSG2/haspin during prophase and dephosphorylated during anaphase. Phosphorylation at Ser-11 (H3S10ph) by AURKB is crucial for chromosome condensation and cell-cycle progression during mitosis and meiosis. In addition phosphorylation at Ser-11 (H3S10ph) by RPS6KA4 and RPS6KA5 is important during interphase because it enables the transcription of genes following external stimulation, like mitogens, stress, growth factors or UV irradiation and result in the activation of genes, such as c-fos and c-jun. Phosphorylation at Ser-11 (H3S10ph), which is linked to gene activation, prevents methylation at Lys-10 (H3K9me) but facilitates acetylation of H3 and H4. Phosphorylation at Ser-11 (H3S10ph) by AURKB mediates the dissociation of HP1 proteins (CBX1, CBX3 and CBX5) from heterochromatin. Phosphorylation at Ser-11 (H3S10ph) is also an essential regulatory mechanism for neoplastic cell transformation. Phosphorylated at Ser-29 (H3S28ph) by MLTK isoform 1, RPS6KA5 or AURKB during mitosis or upon ultraviolet B irradiation. Phosphorylation at Thr-7 (H3T6ph) by PRKCBB is a specific tag for epigenetic transcriptional activation that prevents demethylation of Lys-5 (H3K4me) by LSD1/KDM1A. At centromeres, specifically phosphorylated at Thr-12 (H3T11ph) from prophase to early anaphase, by DAPK3 and PKN1. Phosphorylation at Thr-12 (H3T11ph) by PKN1 is a specific tag for epigenetic transcriptional activation that promotes demethylation of Lys-10 (H3K9me) by KDM4C/JMJD2C. Phosphorylation at Tyr-42 (H3Y41ph) by JAK2 promotes exclusion of CBX5 (HP1 alpha) from chromatin.
Monoubiquitinated by RAG1 in lymphoid cells, monoubiquitination is required for V(D)J recombination (By similarity). Ubiquitinated by the CUL4-DDB-RBX1 complex in response to ultraviolet irradiation. This may weaken the interaction between histones and DNA and facilitate DNA accessibility to repair proteins.

**细胞定位** Nucleus. Chromosome.

## 图片

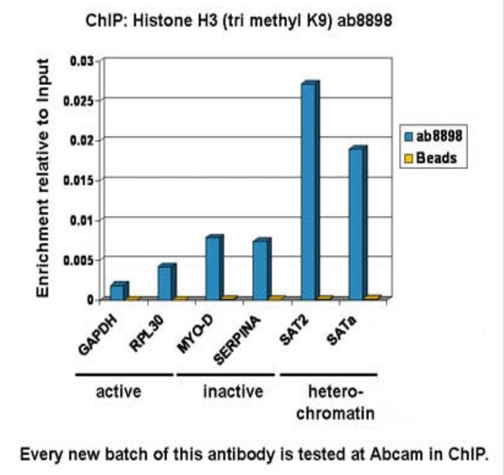


ChIP - Anti-Histone H3 (tri methyl K9) antibody - ChIP Grade (ab8898)

Chromatin was prepared from U2OS cells according to the Abcam X-ChIP protocol. Cells were fixed with formaldehyde for 10 min. The ChIP was performed with 25 µg of chromatin, 2 µg of ab8898 (blue), and 20 µl of protein A/G sepharose beads. No antibody was added to the beads control (yellow). The immunoprecipitated DNA was quantified by real time PCR (Taqman approach for active and inactive loci, Sybr green approach for heterochromatic loci). Primers and probes are located in the first kb of the transcribed region.

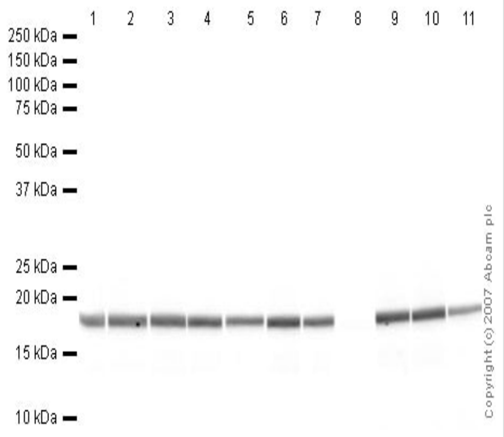


Western blot - Anti-Histone H3 (tri methyl K9) antibody - ChIP Grade (ab8898)

**All lanes :** Anti-Histone H3 (tri methyl K9) antibody - ChIP Grade (ab8898) at 1 µg/ml

**Lane 1 :** Calf Thymus Histone Preparation Nuclear Lysate
**Lane 2 :** Calf Thymus Histone Preparation Nuclear Lysate with Human Histone H3 (unmodified) peptide (ab7228) at 0.5 µg/ml
**Lane 3 :** Calf Thymus Histone Preparation Nuclear Lysate with Human Histone H3 (mono methyl K4) peptide (ab1340) at 0.5 µg/ml
**Lane 4 :** Calf Thymus Histone Preparation Nuclear Lysate with Human Histone H3 (di methyl K4) peptide (ab7768) at 0.5 µg/ml
**Lane 5 :** Calf Thymus Histone Preparation Nuclear Lysate with Human Histone H3 (tri methyl K4) peptide (ab1342) at 0.5 µg/ml
**Lane 6 :** Calf Thymus Histone Preparation Nuclear Lysate with Human Histone H3 (mono methyl K9) peptide (ab1771) at 0.5 µg/ml
**Lane 7 :** Calf Thymus Histone Preparation Nuclear Lysate with Human Histone H3 (di methyl K9) peptide (ab1772) at 0.5 µg/ml
**Lane 8 :** Calf Thymus Histone Preparation Nuclear Lysate with Human Histone H3 (tri methyl K9) peptide (ab1773) at 0.5 µg/ml
**Lane 9 :** Calf Thymus Histone Preparation Nuclear Lysate with Human Histone H3 (mono methyl K27) peptide (ab1780) at 0.5

µg/ml
**Lane 10 :** Calf Thymus Histone Preparation Nuclear Lysate with Human Histone H3 (di methyl K27) peptide (ab1781) at 0.5 µg/ml
**Lane 11 :** Calf Thymus Histone Preparation Nuclear Lysate with Human Histone H3 (tri methyl K27) peptide (ab1782) at 0.5 µg/ml

Lysates/proteins at 0.5 µg per lane.

**Secondary**
**All lanes :** IRDye 680 Conjugated Goat Anti-Rabbit IgG (H+L) at 1/10000 dilution

Performed under reducing conditions.

**Predicted band size:** 15 kDa
**Observed band size:** 17 kDa

Lane 8 shows that Rabbit polyclonal to Histone H3 (tri methyl K9) is blocked by the addition of the immunizing peptide (ab1773). Cross-reactivity with Histone H3 peptide - tri methyl K27 (ab1782) is also shown in Lane 11.

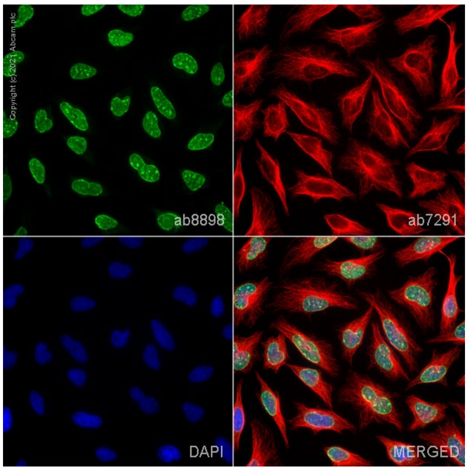


Immunocytochemistry - Anti-Histone H3 (tri methyl K9) antibody - ChIP Grade (ab8898)

ab8898 staining Histone H3 (tri methyl K9) in HeLa cells. The cells were fixed with 100% methanol (5 min), permeabilized with 0.1% PBS-Triton X-100 for 5 minutes and then blocked with 1% BSA/10% normal goat serum/0.3M glycine in 0.1% PBS-Tween for 1h. The cells were then incubated overnight at 4°C with ab8898 at 0.5 µg/ml and ab7291, Mouse monoclonal [DM1A] to alpha Tubulin - Loading Control. Cells were then incubated with ab150081, Goat polyclonal Secondary Antibody to Rabbit IgG - H&L (Alexa Fluor® 488), pre-adsorbed at 1/1000 dilution (shown in green) and ab150120, Goat polyclonal Secondary Antibody to Mouse IgG - H&L (Alexa Fluor® 594), pre-adsorbed at 1/1000 dilution (shown in pseudocolour red). Nuclear DNA was labelled with DAPI (shown in blue).

Also suitable in cells fixed with 4% paraformaldehyde (10 min).

Image was acquired with a high-content analyser (Operetta CLS, Perkin Elmer) and a maximum intensity projection of confocal sections is shown.

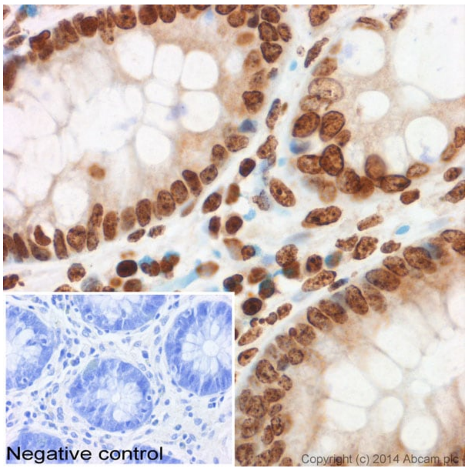


Immunohistochemistry (Formalin/PFA-fixed paraffin-embedded sections) - Anti-Histone H3 (tri methyl K9) antibody - ChIP Grade (ab8898)

IHC image of ab8898 staining Histone H3 (tri methyl K9) in normal human colon formalin-fixed paraffin-embedded tissue sections*, performed on a Leica Bond. The section was pre-treated using heat mediated antigen retrieval with sodium citrate buffer (pH6, epitope retrieval solution 1) for 20 mins. The section was then incubated with ab8898, 1/400 dilution, for 15 mins at room temperature and detected using an HRP conjugated compact polymer system. DAB was used as the chromogen. The section was then counterstained with haematoxylin and mounted with DPX. No primary antibody was used in the negative control (shown on the inset).

For other IHC staining systems (automated and non-automated) customers should optimize variable parameters such as antigen retrieval conditions, primary antibody concentration and antibody incubation times.

*Tissue obtained from the Human Research Tissue Bank, supported by the NIHR Cambridge Biomedical Research Centre

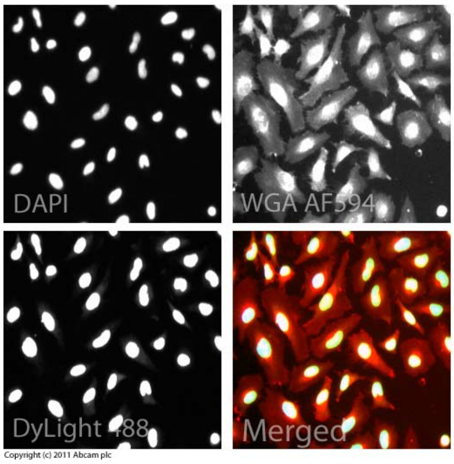


Immunocytochemistry - Anti-Histone H3 (tri methyl K9) antibody - ChIP Grade (ab8898)

ICC/IF image of ab8898 stained HeLa cells. The cells were 100% methanol fixed (5 min) and then incubated in 1%BSA / 10% normal goat serum / 0.3M glycine in 0.1% PBS-Tween for 1h to permeabilise the cells and block non-specific protein-protein interactions. The cells were then incubated with the antibody (ab8898, 0.1µg/ml) overnight at +4°C. The secondary antibody (green) was ab96899, a goat anti-rabbit DyLight® 488 (IgG; H+L) used at a 1/250 dilution for 1h. Alexa Fluor® 594 WGA was used to label plasma membranes (red) at a 1/200 dilution for 1h. DAPI was used to stain the cell nuclei (blue) at a concentration of 1.43µM.

**Please note:** All products are "FOR RESEARCH USE ONLY. NOT FOR USE IN DIAGNOSTIC PROCEDURES"

## Our Abpromise to you: Quality guaranteed and expert technical support

- Replacement or refund for products not performing as stated on the datasheet
- Valid for 12 months from date of delivery
- Response to your inquiry within 24 hours

- We provide support in Chinese, English, French, German, Japanese and Spanish
- Extensive multi-media technical resources to help you

- We investigate all quality concerns to ensure our products perform to the highest standards

If the product does not perform as described on this datasheet, we will offer a refund or replacement. For full details of the Abpromise, please visit https://www.abcam.cn/abpromise or contact our technical team.

## Terms and conditions

- Guarantee only valid for products bought direct from Abcam or one of our authorized distributors

abcam

# Product datasheet

# Anti-HA tag antibody - ChIP Grade ab9110

★★★★★ 58 Abreviews 906 References 8 图像

## 概述

| | |
|---|---|
| 产品名称 | Anti-HA tag抗体- ChIP Grade |
| 描述 | 兔多克隆抗体to HA tag - ChIP Grade |
| 宿主 | Rabbit |
| 特异性 | ELISA: The anti HA diluted 1:70.000 gave an O.D.=1.0 in a 15 minute reaction against peptide conjugated with a different carrier than used for anti peptide purification. HRP conjugated Goat anti rabbit IgG was used and TMB was the substrate. |
| 经测试应用 | **适用于:** ChIP/Chip, IP, ELISA, WB, ICC/IF, Flow Cyt, ChIP |
| 种属反应性 | **与反应:** Species independent |
| 免疫原 | Synthetic peptide corresponding to Influenza A HA tag conjugated to keyhole limpet haemocyanin.<br>Influenza hemagglutinin-HA-epitope<br>Run BLAST with ExPASy Run BLAST with NCBI |
| 阳性对照 | WB: 293FT cells transfected with 15kDa HA tagged Vpr (an HIV1 accessory protein). IP: Nuclear lysate of HEK-293T cells transiently expressing HA-tagged protein. ICC/IF: U-2 cells. Mouse olineu cells. ChIP: Xenopus laevis oocytes were injected with mRNA for HA-tagged human BORIS. |
| 常规说明 | The Life Science industry has been in the grips of a reproducibility crisis for a number of years. Abcam is leading the way in addressing this with our range of recombinant monoclonal antibodies and knockout edited cell lines for gold-standard validation. Please check that this product meets your needs before purchasing.<br>If you have any questions, special requirements or concerns, please send us an inquiry and/or contact our Support team ahead of purchase. Recommended alternatives for this product can be found below, along with publications, customer reviews and Q&As |

## 性能

| | |
|---|---|
| 形式 | Liquid |
| 存放说明 | Shipped at 4°C. Store at +4°C short term (1-2 weeks). Upon delivery aliquot. Store at -20°C or -80°C. Avoid freeze / thaw cycle. |
| 存储溶液 | Preservative: 0.1% Sodium azide<br>Constituent: PBS |
| 纯度 | Immunogen affinity purified |

| | |
|---|---|
| **纯化说明** | Antibodies were immunoaffinity purified using the peptide conjugated to a solid-phase support. |
| **克隆** | 多克隆 |
| **同种型** | IgG |

## 应用

**The Abpromise guarantee** Abpromise™承诺保证使用ab9110于以下的经测试应用

"应用说明"部分 下显示的仅为推荐的起始稀释度;实际最佳的稀释度/浓度应由使用者检定。

| 应用 | Ab评论 | 说明 |
|---|---|---|
| ChIP/Chip | ★★★★★ (1) | Use at an assay dependent concentration. |
| IP | ★★★★★ (12) | Use at an assay dependent concentration. |
| ELISA | | 1/200 - 1/500. |
| WB | ★★★★★ (30) | 1/4000 - 1/10000. |
| ICC/IF | ★★★★★ (8) | Use a concentration of 1 - 4 µg/ml. |
| Flow Cyt | ★★★★☆ (1) | Use at an assay dependent concentration.<br>ab171870 - Rabbit polyclonal IgG, is suitable for use as an isotype control with this antibody. |
| ChIP | ★★★★☆ (2) | Use 3 µg for 25 µg of chromatin. |

## 靶标

**相关性** Human influenza hemagglutinin (HA) is a surface glycoprotein required for the infectivity of the human virus. The HA tag is derived from the HA molecule corresponding to amino acids 98-106 has been extensively used as a general epitope tag in expression vectors. Many recombinant proteins have been engineered to express the HA tag, which does not appear to interfere with the bioactivity or the biodistribution of the recombinant protein. This tag facilitates the detection, isolation, and purification of the proteins.

## 图片

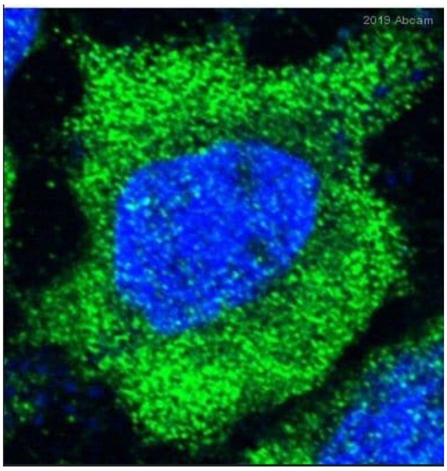


Immunocytochemistry/ Immunofluorescence - Anti-HA tag antibody - ChIP Grade (ab9110)

This image is courtesy of an Abreview

ab9110 staining HA-tagged proteins in HeLa cells by ICC/IF (Immunocytochemistry/immunoflurescence). Cells were fixed with paraformaldehyde, permeabilized with 0.1% saponin and blocked with 3% serum for 30 minutes at 37°C. Samples were incubated with primary antibody (2 µg/ml) in 1x PBS for 1 hour at 37°C. An Alexa Fluor® 488-conjugated Goat polyclonal to rabbit was used as secondary antibody.

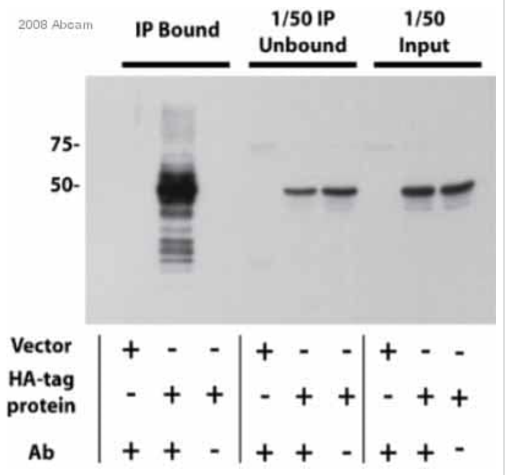


Immunoprecipitation - Anti-HA tag antibody - ChIP Grade (ab9110)

This image is courtesy of an anonymous Abreview

ab9110 was diluted to 4 µg/mg lysate and incubated with a nuclear lysate of HEK293T cells transiently expressing HA-tagged protein and a Protein A matrix for 2 hours a 23°C to achieve immunoprecipitation. 1000 µg of lysate was present in the input.

A HRP-conjugated anti-rabbit HA monoclonal antibody diluted 1/1000 was used for the Western Blot step.

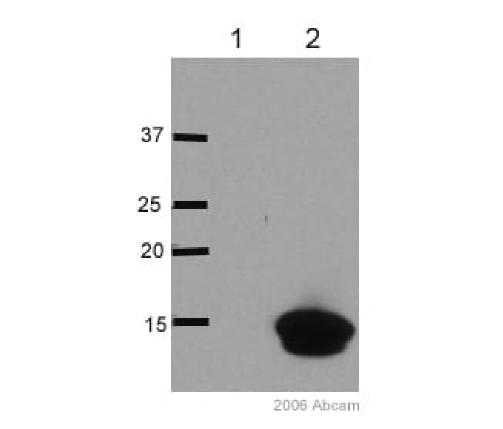


Western blot - Anti-HA tag antibody - ChIP Grade (ab9110)

**All lanes :** Anti-HA tag antibody - ChIP Grade (ab9110) at 1/4000 dilution

**Lane 1 :** 15ug untransfected wcl lysate

**Lane 2 :** 293FT cells transfected with 15kDa HA tagged Vpr (an HIV1 accessory protein)

**Secondary**

**All lanes :** HRP conjugated Goat anti-Rabbit

Developed using the ECL technique.

Performed under reducing conditions.

**Exposure time:** 5 seconds

This image is courtesy of an Abreview

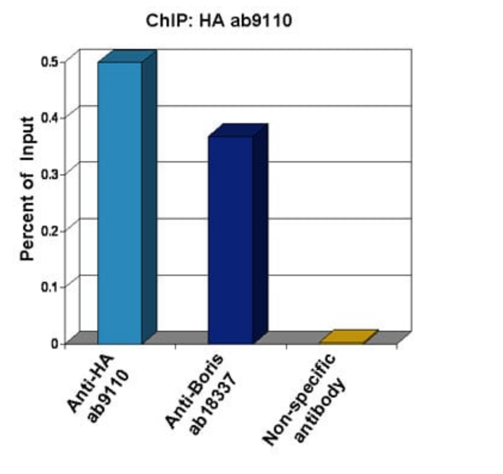


ChIP - Anti-HA tag antibody - ChIP Grade (ab9110)

Xenopus laevis oocytes were injected with mRNA for HA-tagged human BORIS. Chromatin was prepared according to the Abcam X-ChIP protocol. Oocytes were fixed with formaldehyde for 10min. The ChIP was performed with 25µg of chromatin, 20µl of Protein A/G sepharose beads, and 3µg of ab9110 (anti-HA, light blue) or, 3µg of ab18337 (anti-Boris, dark blue). A non-specific antibody was used as a control (yellow). The immunoprecipitated DNA was quantified by real time PCR (Taqman approach).

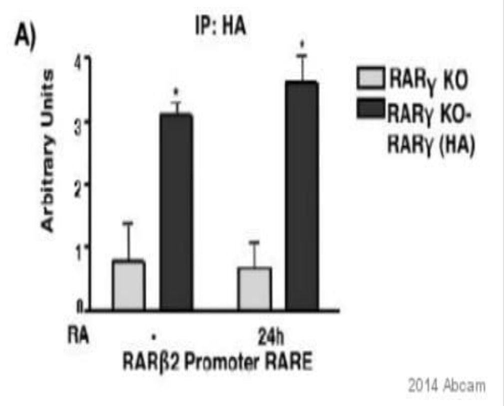


ChIP - Anti-HA tag antibody - ChIP Grade (ab9110)

This image is courtesy of an Abreview submitted by Mr. Dan Stummer

Chromatin was prepared according to the X-ChIP protocol. Mouse embryonic stem whole cell lysate treated with disuccinimidyl glutarate (cross-linking agent). ChIP was performed using ab9110 at 1/200 dilution for 16 hours at 4°C in RIPA diluent. The bound DNA was quantitated by real-time PCR. Negative control: The parent cell line. Positive control: A cell line, which stably express an HA-tagged RARgamma protein.

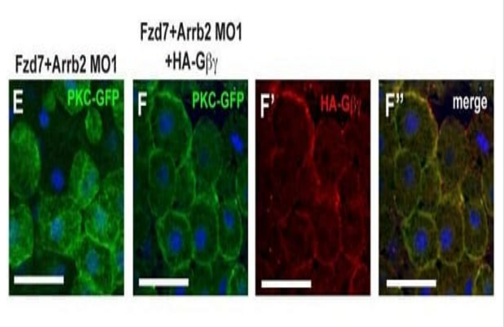


Immunocytochemistry/ Immunofluorescence - Anti-HA tag antibody - ChIP Grade (ab9110)

Seitz, K. et al Send to PLoS One. 2014 Jan 29;9(1):e87132. doi: 10.1371/journal.pone.0087132. eCollection 2014 Reproduced under the Creative Commons license http://creativecommons.org/licenses/by/4.0/

**Arrb2 depends on Gβγ to induce membrane translocation of PKCα**

Xenopus embryos were injected with 500 pg pkcα-gfp RNA and co-injected as indicated above the images. Animal Caps were prepared at stage 10 and immunostained as indicated. Nuclei were stained with Hoechst 33258 (blue). Images show representative results from at least two independent experiments with a minimum of six Animal Caps per experiment. Scale bars: 50 µm.

The inhibitory effect of Arrb2 MO1 (E) on PKCα-GFP membrane translocation was rescued by (F) co-injection of HA-Gβ and HA-Gγ mRNA (anti-HA (red): F′, merge: F").

HA was detected with ab9110.

(After Figure 2 of Seitz et al)

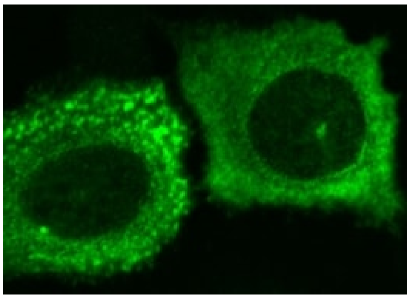

Immunocytochemistry/ Immunofluorescence - Anti-HA tag antibody - ChIP Grade (ab9110)

This image was kindly supplied as part of the review submitted by Kasper Fugger. Immunofluorescence staining of U-2 cells expressing HA-tagged protein with ab9110.

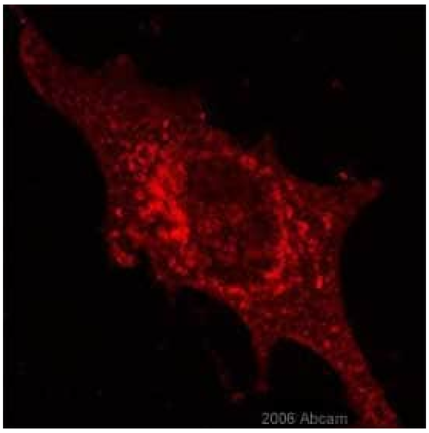


Immunocytochemistry/ Immunofluorescence - Anti-HA tag antibody - ChIP Grade (ab9110)

ab 9110 at a 1/200 dilution staining the mouse olineu cell line (oligodendrocyte precursor cell) by immunocytochemistry. The antibody was incubated with the cells for 30 minutes and then detected using a Cy5 conjugated goat anti-rabbit antibody.

This image is courtesy of an Abreview submitted by **Katarina Trajkovic** on **15 March 2006**

**Please note:** All products are "FOR RESEARCH USE ONLY. NOT FOR USE IN DIAGNOSTIC PROCEDURES"

## Our Abpromise to you: Quality guaranteed and expert technical support

- Replacement or refund for products not performing as stated on the datasheet
- Valid for 12 months from date of delivery
- Response to your inquiry within 24 hours

- We provide support in Chinese, English, French, German, Japanese and Spanish
- Extensive multi-media technical resources to help you
- We investigate all quality concerns to ensure our products perform to the highest standards

If the product does not perform as described on this datasheet, we will offer a refund or replacement. For full details of the Abpromise, please visit https://www.abcam.cn/abpromise or contact our technical team.

## Terms and conditions

- Guarantee only valid for products bought direct from Abcam or one of our authorized distributors

abcam

# Product datasheet

# Anti-Histone H4 (acetyl K8) antibody [EP1002Y] - ChIP Grade ab45166

重组 RabMAb

★★★★★ 1 Abreviews 13 References 13 图像

## 概述

| | |
|---|---|
| 产品名称 | Anti-Histone H4 (acetyl K8)抗体[EP1002Y] - ChIP Grade |
| 描述 | 兔单克隆抗体[EP1002Y] to Histone H4 (acetyl K8) - ChIP Grade |
| 宿主 | Rabbit |
| 经测试应用 | **适用于:** Flow Cyt (Intra), ChIP, ChIP-sequencing, WB, IHC-P, ICC/IF, IP |
| 种属反应性 | **与反应:** Mouse, Rat, Human |
| 免疫原 | Synthetic peptide within Human Histone H4 aa 1-100 (N terminal) (acetyl K8). The exact sequence is proprietary.<br>Database link: P62805 |
| 阳性对照 | WB: HeLa whole cell lysate +TSA, C6 cell lysate, C6 cell + TSA lysate, NIH/3T3 +TSA whole cell lysate. IHC-P: Human normal colon FFPE tissue sections, mouse kidney paraffin-embedded tissue sections, rat kidney paraffin-embedded tissue sections. ICC/IF: C6 + TSA lysates. ChIP: Chromatin prepared from HeLa cells ChiP-Seq: HeLa Cells |
| 常规说明 | This product is a recombinant monoclonal antibody, which offers several advantages including:<br>- High batch-to-batch consistency and reproducibility<br>- Improved sensitivity and specificity<br>- Long-term security of supply<br>- Animal-free production<br>For more information see here.<br>Our RabMAb® technology is a patented hybridoma-based technology for making rabbit monoclonal antibodies. For details on our patents, please refer to RabMAb® patents.<br>**We are constantly working hard to ensure we provide our customers with best in class antibodies. As a result of this work we are pleased to now offer this antibody in purified format. We are in the process of updating our datasheets. The purified format is designated 'PUR' on our product labels. If you have any questions regarding this update, please contact our Scientific Support team.** |

## 性能

| | |
|---|---|
| 形式 | Liquid |

| | |
|---|---|
| **存放说明** | Shipped at 4°C. Store at +4°C short term (1-2 weeks). Upon delivery aliquot. Store at -20°C. Avoid freeze / thaw cycle. |
| **存储溶液** | pH: 7.20<br>Preservative: 0.01% Sodium azide<br>Constituents: 59% PBS, 40% Glycerol (glycerin, glycerine), 0.17% BSA |
| **纯度** | Protein A purified |
| **克隆** | 单克隆 |
| **克隆编号** | EP1002Y |
| **同种型** | IgG |

## 应用

**The Abpromise guarantee** Abpromise™承诺保证使用ab45166于以下的经测试应用

“应用说明”部分 下显示的仅为推荐的起始稀释度；实际最佳的稀释度/浓度应由使用者检定。

| 应用 | Ab评论 | 说明 |
|---|---|---|
| **Flow Cyt (Intra)** | | Use at an assay dependent concentration. |
| **ChIP** | ★★★★★ (1) | Use 2 µg for 25 µg of chromatin. |
| **ChIP-sequencing** | | Use 4µg for $10^7$ cells. |
| **WB** | | 1/5000 - 1/10000. Predicted molecular weight: 11 kDa. |
| **IHC-P** | | 1/250 - 1/2500. Perform heat mediated antigen retrieval with Tris/EDTA buffer pH 9.0 before commencing with IHC staining protocol. See IHC antigen retrieval protocols. |
| **ICC/IF** | | 1/150 - 1/500. |
| **IP** | | 1/20 - 1/50. |

## 靶标

| | |
|---|---|
| **功能** | Core component of nucleosome. Nucleosomes wrap and compact DNA into chromatin, limiting DNA accessibility to the cellular machineries which require DNA as a template. Histones thereby play a central role in transcription regulation, DNA repair, DNA replication and chromosomal stability. DNA accessibility is regulated via a complex set of post-translational modifications of histones, also called histone code, and nucleosome remodeling. |
| **序列相似性** | Belongs to the histone H4 family. |
| **翻译后修饰** | Acetylation at Lys-6 (H4K5ac), Lys-9 (H4K8ac), Lys-13 (H4K12ac) and Lys-17 (H4K16ac) occurs in coding regions of the genome but not in heterochromatin.<br>Citrullination at Arg-4 (H4R3ci) by PADI4 impairs methylation.<br>Monomethylation and asymmetric dimethylation at Arg-4 (H4R3me1 and H4R3me2a, respectively) by PRMT1 favors acetylation at Lys-9 (H4K8ac) and Lys-13 (H4K12ac). Demethylation is performed by JMJD6. Symmetric dimethylation on Arg-4 (H4R3me2s) by the |

PRDM1/PRMT5 complex may play a crucial role in the germ-cell lineage.
Monomethylated, dimethylated or trimethylated at Lys-21 (H4K20me1, H4K20me2, H4K20me3). Monomethylation is performed by SET8. Trimethylation is performed by SUV420H1 and SUV420H2 and induces gene silencing.
Ubiquitinated by the CUL4-DDB-RBX1 complex in response to ultraviolet irradiation. This may weaken the interaction between histones and DNA and facilitate DNA accessibility to repair proteins. Monoubiquitinated at Lys-92 of histone H4 (H4K91ub1) in response to DNA damage. The exact role of H4K91ub1 in DNA damage response is still unclear but it may function as a licensing signal for additional histone H4 post-translational modifications such as H4 Lys-21 methylation (H4K20me).
Sumoylated, which is associated with transcriptional repression.

**细胞定位** Nucleus. Chromosome.

## 图片

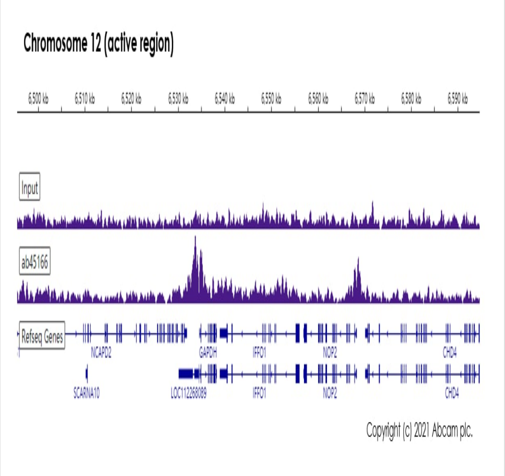


ChIP-sequencing - Anti-Histone H4 (acetyl K8) antibody [EP1002Y] - ChIP Grade (ab45166)

Chromatin was prepared from HeLa cells. Cells were fixed with 1% formaldehyde for 10 minutes. ChIP was performed with 10^7 HeLa cells and 4 µg of ab45166 [EP1002Y]. ChIP DNA was sequenced on the Illumina NovaSeq 6000 to a depth of 30 million reads.

Additional screenshots of mapped reads can be downloaded here.

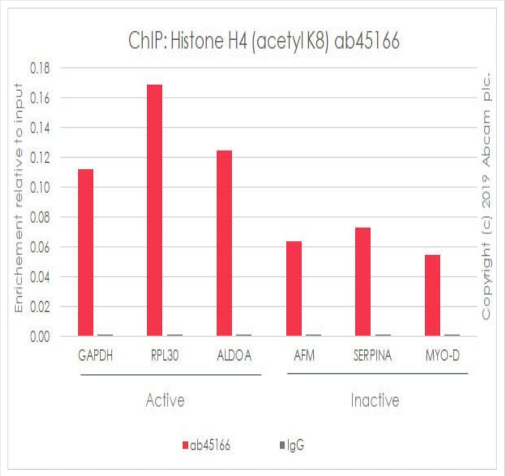


ChIP - Anti-Histone H4 (acetyl K8) antibody [EP1002Y] - ChIP Grade (ab45166)

Chromatin was prepared from HeLa cells according to the Abcam X-ChIP protocol. Cells were fixed with formaldehyde for 10 minutes. The ChIP was performed with 25µg of chromatin, 2µg of ab45166 (red), and 20µl of Protein A/G sepharose beads. No antibody was added to the beads control (grey). The immunoprecipitated DNA was quantified by real time PCR (Taqman approach). Primers and probes are located in the first kb of the transcribed region.

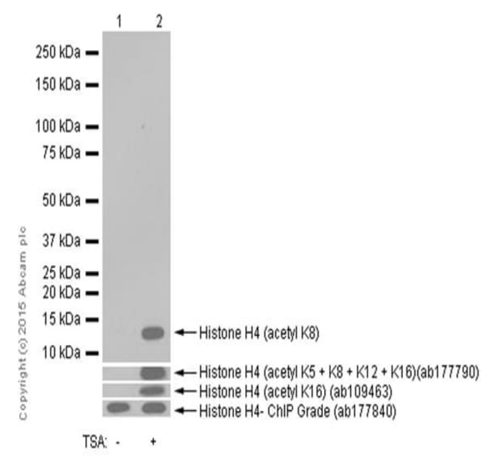


Western blot - Anti-Histone H4 (acetyl K8) antibody [EP1002Y] - ChIP Grade (ab45166)

**All lanes :** Anti-Histone H4 (acetyl K8) antibody [EP1002Y] - ChIP Grade (ab45166) at 1/5000 dilution

**Lane 1 :** Untreated HeLa (human cervix adenocarcinoma) whole cell lysate
**Lane 2 :** HeLa (human cervix adenocarcinoma) treated with Trichostatin A whole cell lysate

Lysates/proteins at 10 µg per lane.

**Secondary**
**All lanes :** Goat Anti-Rabbit IgG H&L (HRP) (ab97051) at 1/20000 dilution

**Predicted band size:** 11 kDa
**Observed band size:** 11 kDa

Blocking and diluting buffer 5% NFDM/TBST

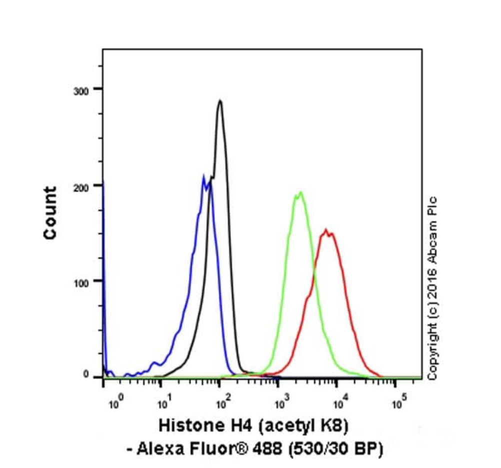


Flow Cytometry (Intracellular) - Anti-Histone H4 (acetyl K8) antibody [EP1002Y] - ChIP Grade (ab45166)

Intracellular Flow Cytometry analysis of HeLa (human cervix adenocarcinoma) treated (Red)/untreated (Green) with 500ng/ml Trichostatin A for 4 hours with purified ab45166 at 1/20 dilution. The secondary antibody was Goat anti rabbit IgG (Alexa Fluorr® 488) at 1/2000 dilution. A Rabbit monoclonal IgG (Black) was used as the isotype control and cells without incubation with primary antibody and secondary antibody (Blue) were used as unlabeled control.

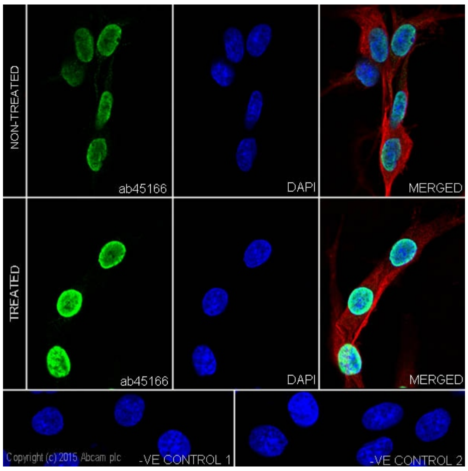


Immunocytochemistry/ Immunofluorescence - Anti-Histone H4 (acetyl K8) antibody [EP1002Y] - ChIP Grade (ab45166)

Immunocytochemistry/immunofluorescence staining of 4% paraformaldehyde fixed; 0.1% triton X 100 permeabilized C6 (rat glioma) cells (non-treated-top panels) and (C6 + TSA(500ng/ml, 4hr)-middle panels) with purified ab45166 at dilution of 1/150. The secondary antibody used was Alexa Fluor® 488; goat anti-rabbit IgG (ab150077) at a dilution of 1/1000. Nucleus was counter-stained with DAPI (blue). ab7291, a mouse anti-tubulin antibody (1/1000) was used to stain tubulin along with ab150120 (AlexaFluor®594 goat anti-mouse secondary, 1/1000) shown in the top right and middle right hand panels. The negative controls are shown in the bottom two panels- for negative control 1 rabbit primary antibody and anti-mouse secondary antibody (ab150120) was used. For negative control 2 mouse primary antibody (ab7291) and anti-rabbit secondary antibody (ab150077) was used.

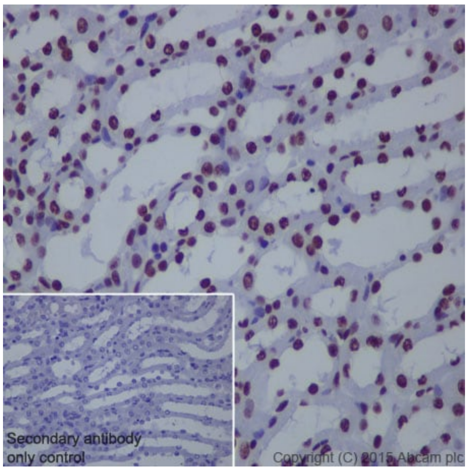


Immunohistochemistry (Formalin/PFA-fixed paraffin-embedded sections) - Anti-Histone H4 (acetyl K8) antibody [EP1002Y] - ChIP Grade (ab45166)

Immunohistochemical staining of paraffin-embedded mouse kidney sections labelling Histone H4 (acetyl K8) with purified ab45166 at dilution of 1:2500. The secondary antibody used was ab97051; a goat anti-rabbit IgG H&L (HRP) at dilution of 1/500. The sample was counter-stained with hematoxylin. Antigen retrieval was performed using EDTA Buffer; pH 9.0. PBS was used instead of the primary antibody as the negative control and is shown in the inset.

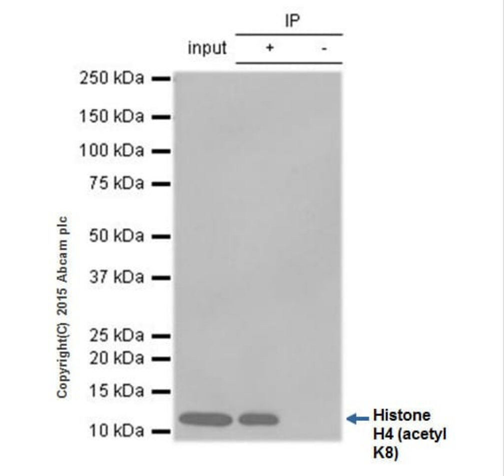


Immunoprecipitation - Anti-Histone H4 (acetyl K8) antibody [EP1002Y] - ChIP Grade (ab45166)

ab45166 (purified) at 1/20 immunoprecipitating Histone H4 (acetyl K8) in HeLa treated with Trichostatin A whole cell lysate.

Lane 1 (input): HeLa treated with Trichostatin A whole cell lysate (10µg)

Lane 2 (+): ab45166 + HeLa treated with Trichostatin A whole cell lysate.

Lane 3 (-): Rabbit monoclonal IgG (ab172730) instead of ab45166 in HeLa treated with Trichostatin A whole cell lysate.

For western blotting, ab131366 VeriBlot for IP Detection Reagent (HRP) was used for detection (1/10000).

Blocking buffer and concentration: 5% NFDM/TBST.

Diluting buffer and concentration: 5% NFDM /TBST.

**Lanes 1-2 :** Anti-Histone H4 (acetyl K8) antibody [EP1002Y] - ChIP Grade (ab45166) at 1/20 dilution
**Lane 3 :** Rabbit IgG, monoclonal [EPR25A] - Isotype Control (ab172730) at 1/20 dilution

**Lane 1 :** HeLa (human cervix adenocarcinoma) treated with Trichostatin A whole cell lysate at 10 µg
**Lanes 2-3 :** HeLa (human cervix adenocarcinoma) treated with Trichostatin A whole cell lysate

**Secondary**
**All lanes :** VeriBlot for IP Detection Reagent (HRP) (ab131366) at 1/10000 dilution

**Observed band size:** 11 kDa

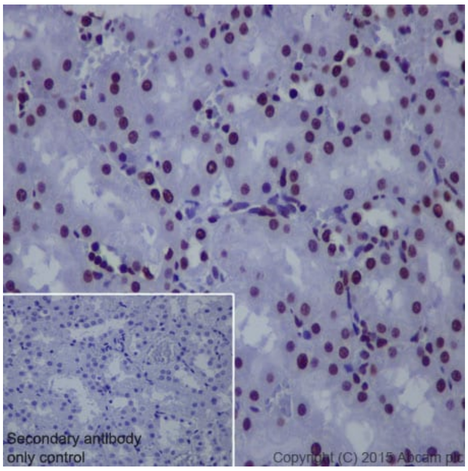


Immunohistochemistry (Formalin/PFA-fixed paraffin-embedded sections) - Anti-Histone H4 (acetyl K8) antibody [EP1002Y] - ChIP Grade (ab45166)

Immunohistochemical staining of paraffin-embedded rat kidney sections labelling Histone H4 (acetyl K8) with purified ab45166 at dilution of 1:2500. The secondary antibody used was ab97051; a goat anti-rabbit IgG H&L (HRP) at dilution of 1/500. The sample was counter-stained with hematoxylin. Antigen retrieval was performed using EDTA Buffer; pH 9.0. PBS was used instead of the primary antibody as the negative control and is shown in the inset.

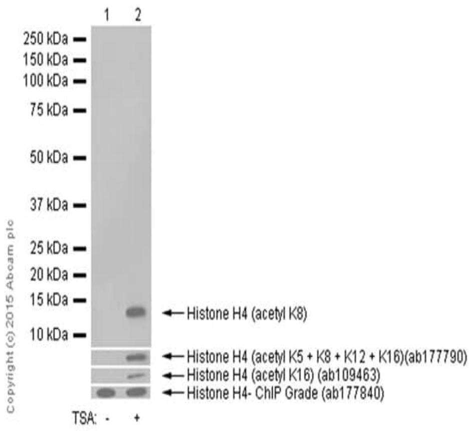


Western blot - Anti-Histone H4 (acetyl K8) antibody [EP1002Y] - ChIP Grade (ab45166)

**All lanes :** Anti-Histone H4 (acetyl K8) antibody [EP1002Y] - ChIP Grade (ab45166) at 1/5000 dilution

**Lane 1 :** Untreated C6 (rat glioma) whole cell lysate
**Lane 2 :** C6 (rat glioma) treated with Trichostatin A whole cell lysate

Lysates/proteins at 10 µg per lane.

**Secondary**
**All lanes :** Goat Anti-Rabbit IgG H&L (HRP) (ab97051) at 1/20000 dilution

**Predicted band size:** 11 kDa
**Observed band size:** 11 kDa

Blocking and diluting buffer 5% NFDM/TBST

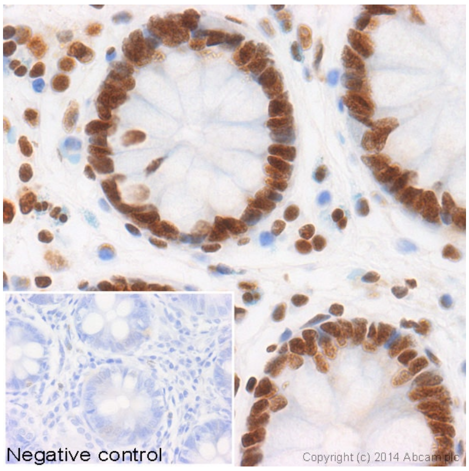


Immunohistochemistry (Formalin/PFA-fixed paraffin-embedded sections) - Anti-Histone H4 (acetyl K8) antibody [EP1002Y] - ChIP Grade (ab45166)

Immunohistochemical analysis of formalin fixed paraffin embedded human colon tissue sections labelling Histone H4 (acetyl K8) with unpurified ab45166 at dilution of 1/200.

For other IHC staining systems (automated and non-automated) customers should optimize variable parameters such as antigen retrieval conditions, primary antibody concentration and antibody incubation times.

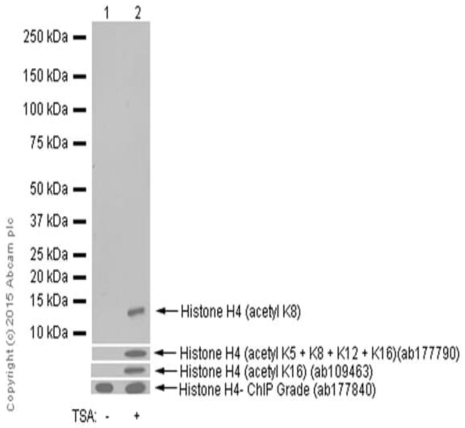


Western blot - Anti-Histone H4 (acetyl K8) antibody [EP1002Y] - ChIP Grade (ab45166)

**All lanes :** Anti-Histone H4 (acetyl K8) antibody [EP1002Y] - ChIP Grade (ab45166) at 1/5000 dilution

**Lane 1 :** Untreated NIH/3T3 (mouse embryo) whole cell lysate
**Lane 2 :** NIH/3T3 (mouse embryo) treated with Trichostatin A whole cell lysate

Lysates/proteins at 10 µg per lane.

**Secondary**
**All lanes :** Goat Anti-Rabbit IgG H&L (HRP) (ab97051) at 1/20000 dilution

**Predicted band size:** 11 kDa
**Observed band size:** 11 kDa

Blocking and diluting buffer 5% NFDM/TBST

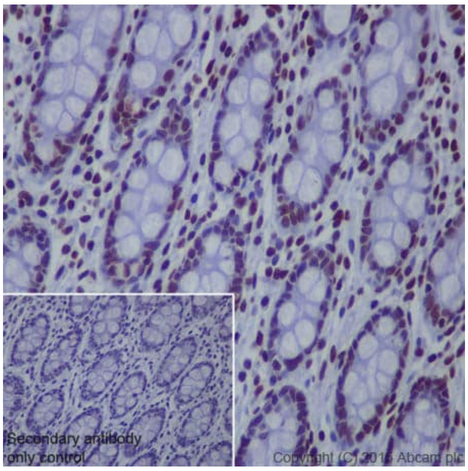


Immunohistochemistry (Formalin/PFA-fixed paraffin-embedded sections) - Anti-Histone H4 (acetyl K8) antibody [EP1002Y] - ChIP Grade (ab45166)

Immunohistochemical staining of paraffin-embedded human colon sections labelling Histone H4 (acetyl K8) with purified ab45166 at dilution of 1:2500. The secondary antibody used was ab97051; a goat anti-rabbit IgG H&L (HRP) at dilution of 1/500. The sample was counter-stained with hematoxylin. Antigen retrieval was performed using EDTA Buffer; pH 9.0. PBS was used instead of the primary antibody as the negative control and is shown in the inset.

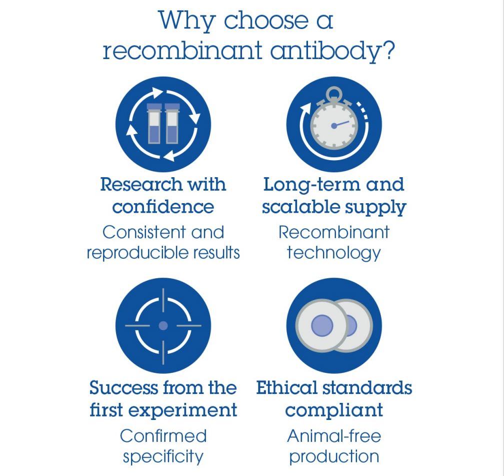


Anti-Histone H4 (acetyl K8) antibody [EP1002Y] - ChIP Grade (ab45166)

**Please note:** All products are "FOR RESEARCH USE ONLY. NOT FOR USE IN DIAGNOSTIC PROCEDURES"

## Our Abpromise to you: Quality guaranteed and expert technical support

- Replacement or refund for products not performing as stated on the datasheet
- Valid for 12 months from date of delivery
- Response to your inquiry within 24 hours

- We provide support in Chinese, English, French, German, Japanese and Spanish
- Extensive multi-media technical resources to help you
- We investigate all quality concerns to ensure our products perform to the highest standards

If the product does not perform as described on this datasheet, we will offer a refund or replacement. For full details of the Abpromise, please visit https://www.abcam.cn/abpromise or contact our technical team.

## Terms and conditions

- Guarantee only valid for products bought direct from Abcam or one of our authorized distributors

abcam

## Product datasheet

# Anti-Phospho - (Ser/Thr) antibody ab117253

★★★☆☆ 1 Abreviews 9 References

### 概述

| | |
|---|---|
| **产品名称** | Anti-Phospho - (Ser/Thr)抗体 |
| **描述** | 兔多克隆抗体to Phospho - (Ser/Thr) |
| **宿主** | Rabbit |
| **经测试应用** | **适用于:** ELISA, Dot blot |
| **种属反应性** | **与反应:** Species independent |
| **免疫原** | Phosphoserine/threonine conjugated with R-PE. |
| **常规说明** | Buffers and proteins which contain phosphate should be avoided with this antibody. Certain proteins known to contain phosphorylated serine and threonine may not be detected by this antibody due to steric hindrance.<br>The immunogen used is Phosphoserine and phosphothreonine conjugated with R-PE.<br><br>The Life Science industry has been in the grips of a reproducibility crisis for a number of years. Abcam is leading the way in addressing this with our range of recombinant monoclonal antibodies and knockout edited cell lines for gold-standard validation. Please check that this product meets your needs before purchasing.<br>If you have any questions, special requirements or concerns, please send us an inquiry and/or contact our Support team ahead of purchase. Recommended alternatives for this product can be found below, along with publications, customer reviews and Q&As |

### 性能

| | |
|---|---|
| **形式** | Liquid |
| **存放说明** | Shipped at 4°C. Store at +4°C short term (1-2 weeks). Store at -20°C or -80°C. Avoid freeze / thaw cycle. |
| **存储溶液** | pH: 7.40<br>Preservative: 0.05% Sodium azide<br>Constituents: 0.16% Tris HCl, 0.88% Sodium chloride |
| **纯度** | Protein A purified |
| **克隆** | 多克隆 |

**同种型** IgG

## 应用

**The Abpromise guarantee** Abpromise™承诺保证使用ab117253于以下的经测试应用

“应用说明”部分 下显示的仅为推荐的起始稀释度；实际最佳的稀释度/浓度应由使用者检定。

| 应用 | Ab评论 | 说明 |
|---|---|---|
| **ELISA** | | 1/10000 - 1/50000. |
| **Dot blot** | | Use at an assay dependent dilution. |

## 靶标

**相关性** A hallmark of signal transduction pathways is the reversible phosphorylation of serine and threonine residues within specific sequences, or motifs, in target proteins. Specific signaling motifs include not only sequences that are recognized by protein kinases, but also those that are recognized by phosphorylation-dependent binding proteins like 14-3-3. These modular phosphoprotein interacting domains are critical elements in modulating, directing and amplifying intracellular communications. Many critical protein kinases can be regulated by phosphorylation at a specific serine or threonine surrounded by phenylalanine or tyrosine. For example, Akt, an important kinase that regulates cell survival, is activated by phosphorylation at Ser473, a site surrounded by phenylalanine and tyrosine. RSK1, p70 S6 K, and certain PKC isoforms also contain a similar consensus phosphorylation site. Phosphorylation of these sites is required for kinase activity.

**Please note:** All products are "FOR RESEARCH USE ONLY. NOT FOR USE IN DIAGNOSTIC PROCEDURES"

## Our Abpromise to you: Quality guaranteed and expert technical support

- Replacement or refund for products not performing as stated on the datasheet
- Valid for 12 months from date of delivery
- Response to your inquiry within 24 hours

- We provide support in Chinese, English, French, German, Japanese and Spanish
- Extensive multi-media technical resources to help you
- We investigate all quality concerns to ensure our products perform to the highest standards

If the product does not perform as described on this datasheet, we will offer a refund or replacement. For full details of the Abpromise, please visit https://www.abcam.cn/abpromise or contact our technical team.

## Terms and conditions

- Guarantee only valid for products bought direct from Abcam or one of our authorized distributors

abcam

# Product datasheet

# Anti-Histone H4 (acetyl K5) antibody ab124636

2 References    3 图像

## 概述

| | |
|---|---|
| **产品名称** | Anti-Histone H4 (acetyl K5)抗体 |
| **描述** | 兔多克隆抗体to Histone H4 (acetyl K5) |
| **宿主** | Rabbit |
| **特异性** | ab124636 detects endogenous levels of total Histone H4 protein only when acetylated at Lysine 5. |
| **经测试应用** | **适用于:** WB, IHC-P, ICC/IF |
| **种属反应性** | **与反应:** Human, African green monkey<br>**预测可用于:** Mouse, Rat |
| **免疫原** | Synthetic peptide corresponding to Human Histone H4 (acetyl K5). A synthetic peptide from human Histone H4 around the acetylated site of Lys5.<br>Database link: P62805 |
| **阳性对照** | Human prostate, COS7 cells, HeLa cells. |
| **常规说明** | The Life Science industry has been in the grips of a reproducibility crisis for a number of years. Abcam is leading the way in addressing this with our range of recombinant monoclonal antibodies and knockout edited cell lines for gold-standard validation. Please check that this product meets your needs before purchasing.<br>If you have any questions, special requirements or concerns, please send us an inquiry and/or contact our Support team ahead of purchase. Recommended alternatives for this product can be found below, along with publications, customer reviews and Q&As |

## 性能

| | |
|---|---|
| **形式** | Liquid |
| **存放说明** | Shipped at 4°C. Store at -20°C. Stable for 12 months at -20°C. |
| **存储溶液** | pH: 7.40<br>Preservative: 0.02% Sodium azide<br>Constituents: 49% PBS, 0.9% Sodium chloride, 50% Glycerol (glycerin, glycerine)<br><br>without Mg2+, Ca2+ |
| **纯度** | Immunogen affinity purified |
| **克隆** | 多克隆 |

**同种型** IgG

## 应用

**The Abpromise guarantee** Abpromise™承诺保证使用ab124636于以下的经测试应用

“应用说明”部分 下显示的仅为推荐的起始稀释度；实际最佳的稀释度/浓度应由使用者检定。

| 应用 | Ab评论 | 说明 |
|---|---|---|
| **WB** | | 1/500 - 1/1000. Predicted molecular weight: 11 kDa. |
| **IHC-P** | | Use a concentration of 5 µg/ml. |
| **ICC/IF** | | 1/100 - 1/500. |

## 靶标

**功能** Core component of nucleosome. Nucleosomes wrap and compact DNA into chromatin, limiting DNA accessibility to the cellular machineries which require DNA as a template. Histones thereby play a central role in transcription regulation, DNA repair, DNA replication and chromosomal stability. DNA accessibility is regulated via a complex set of post-translational modifications of histones, also called histone code, and nucleosome remodeling.

**序列相似性** Belongs to the histone H4 family.

**翻译后修饰** Acetylation at Lys-6 (H4K5ac), Lys-9 (H4K8ac), Lys-13 (H4K12ac) and Lys-17 (H4K16ac) occurs in coding regions of the genome but not in heterochromatin.
Citrullination at Arg-4 (H4R3ci) by PADI4 impairs methylation.
Monomethylation and asymmetric dimethylation at Arg-4 (H4R3me1 and H4R3me2a, respectively) by PRMT1 favors acetylation at Lys-9 (H4K8ac) and Lys-13 (H4K12ac). Demethylation is performed by JMJD6. Symmetric dimethylation on Arg-4 (H4R3me2s) by the PRDM1/PRMT5 complex may play a crucial role in the germ-cell lineage.
Monomethylated, dimethylated or trimethylated at Lys-21 (H4K20me1, H4K20me2, H4K20me3). Monomethylation is performed by SET8. Trimethylation is performed by SUV420H1 and SUV420H2 and induces gene silencing.
Ubiquitinated by the CUL4-DDB-RBX1 complex in response to ultraviolet irradiation. This may weaken the interaction between histones and DNA and facilitate DNA accessibility to repair proteins. Monoubiquitinated at Lys-92 of histone H4 (H4K91ub1) in response to DNA damage. The exact role of H4K91ub1 in DNA damage response is still unclear but it may function as a licensing signal for additional histone H4 post-translational modifications such as H4 Lys-21 methylation (H4K20me).
Sumoylated, which is associated with transcriptional repression.

**细胞定位** Nucleus. Chromosome.

## 图片

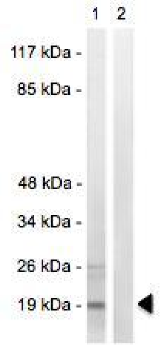


Western blot - Anti-Histone H4 (acetyl K5) antibody (ab124636)

**All lanes :** Anti-Histone H4 (acetyl K5) antibody (ab124636) at 1/500 dilution

**Lane 1 :** COS7 cell extract, treated with TSA 400nM 24h.
**Lane 2 :** COS7 cell extract, treated with TSA 400nM 24h. with synthesized peptide

**Predicted band size:** 11 kDa

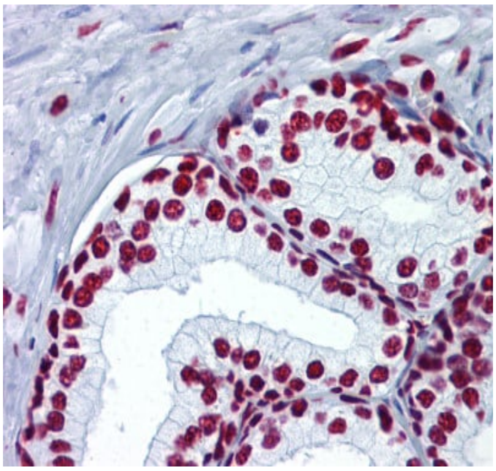

Immunohistochemistry (Formalin/PFA-fixed paraffin-embedded sections) - Anti-Histone H4 (acetyl K5) antibody (ab124636)

ab124636, at 5µg/mL, staining Human prostate by immunohistochemistry of paraffin embedded tissues (IHC-P).

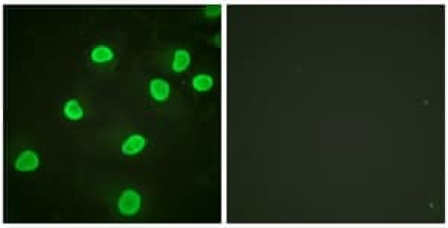

Immunocytochemistry/ Immunofluorescence - Anti-Histone H4 (acetyl K5) antibody (ab124636)

ab124636, at 1/100 dilution, staining HeLa cells, by immunofluorescence. The picture on the right is treated with synthesized peptide.

**Please note:** All products are "FOR RESEARCH USE ONLY. NOT FOR USE IN DIAGNOSTIC PROCEDURES"

- Replacement or refund for products not performing as stated on the datasheet
- Valid for 12 months from date of delivery
- Response to your inquiry within 24 hours

- We provide support in Chinese, English, French, German, Japanese and Spanish
- Extensive multi-media technical resources to help you
- We investigate all quality concerns to ensure our products perform to the highest standards

If the product does not perform as described on this datasheet, we will offer a refund or replacement. For full details of the Abpromise, please visit https://www.abcam.cn/abpromise or contact our technical team.

## Terms and conditions

- Guarantee only valid for products bought direct from Abcam or one of our authorized distributors

abcam

## Product datasheet

# Anti-Histone H4 (tri methyl K20) antibody [EPR17001(2)] - ChIP Grade ab177190

重组 RabMAb

★★★★★ 4 Abreviews 1 References 7 图像

### 概述

| | |
|---|---|
| **产品名称** | Anti-Histone H4 (tri methyl K20)抗体[EPR17001(2)] - ChIP Grade |
| **描述** | 兔单克隆抗体[EPR17001(2)] to Histone H4 (tri methyl K20) - ChIP Grade |
| **宿主** | Rabbit |
| **经测试应用** | **适用于:** IHC-P, WB, Dot blot, ChIP |
| **种属反应性** | **与反应:** Mouse, Rat, Human |
| **免疫原** | Synthetic peptide. This information is proprietary to Abcam and/or its suppliers. |
| **阳性对照** | WB: HeLa and NIH/3T3 cell lysates. IHC-P: Human, mouse and rat colon tissues. ICC/IF: HeLa cells. ChIP: Chromatin prepared from HeLa cells. |
| **常规说明** | This product is a recombinant monoclonal antibody, which offers several advantages including:<br>- High batch-to-batch consistency and reproducibility<br>- Improved sensitivity and specificity<br>- Long-term security of supply<br>- Animal-free production<br>For more information see here.<br>Our RabMAb® technology is a patented hybridoma-based technology for making rabbit monoclonal antibodies. For details on our patents, please refer to RabMAb® patents. |

### 性能

| | |
|---|---|
| **形式** | Liquid |
| **存放说明** | Shipped at 4°C. Store at +4°C short term (1-2 weeks). Upon delivery aliquot. Store at -20°C long term. Avoid freeze / thaw cycle. |
| **存储溶液** | Preservative: 0.01% Sodium azide<br>Constituents: PBS, 40% Glycerol (glycerin, glycerine), 0.05% BSA |
| **纯度** | Protein A purified |
| **克隆** | 单克隆 |
| **克隆编号** | EPR17001(2) |
| **同种型** | IgG |

## 应用

**The Abpromise guarantee** Abpromise™承诺保证使用ab177190于以下的经测试应用

“应用说明”部分 下显示的仅为推荐的起始稀释度;实际最佳的稀释度/浓度应由使用者检定。

| 应用 | Ab评论 | 说明 |
|---|---|---|
| **IHC-P** | ★★★★★ (1) | 1/8000. Perform heat mediated antigen retrieval with Tris/EDTA buffer pH 9.0 before commencing with IHC staining protocol. |
| **WB** | | 1/1000. Detects a band of approximately 11 kDa (predicted molecular weight: 11 kDa). |
| **Dot blot** | | 1/1000. |
| **ChIP** | ★★★★★ (2) | Use 2 µg for 25 µg of chromatin. |

## 靶标

**功能** Core component of nucleosome. Nucleosomes wrap and compact DNA into chromatin, limiting DNA accessibility to the cellular machineries which require DNA as a template. Histones thereby play a central role in transcription regulation, DNA repair, DNA replication and chromosomal stability. DNA accessibility is regulated via a complex set of post-translational modifications of histones, also called histone code, and nucleosome remodeling.

**序列相似性** Belongs to the histone H4 family.

**翻译后修饰** Acetylation at Lys-6 (H4K5ac), Lys-9 (H4K8ac), Lys-13 (H4K12ac) and Lys-17 (H4K16ac) occurs in coding regions of the genome but not in heterochromatin.
Citrullination at Arg-4 (H4R3ci) by PADI4 impairs methylation.
Monomethylation and asymmetric dimethylation at Arg-4 (H4R3me1 and H4R3me2a, respectively) by PRMT1 favors acetylation at Lys-9 (H4K8ac) and Lys-13 (H4K12ac).
Demethylation is performed by JMJD6. Symmetric dimethylation on Arg-4 (H4R3me2s) by the PRDM1/PRMT5 complex may play a crucial role in the germ-cell lineage.
Monomethylated, dimethylated or trimethylated at Lys-21 (H4K20me1, H4K20me2, H4K20me3).
Monomethylation is performed by SET8. Trimethylation is performed by SUV420H1 and SUV420H2 and induces gene silencing.
Ubiquitinated by the CUL4-DDB-RBX1 complex in response to ultraviolet irradiation. This may weaken the interaction between histones and DNA and facilitate DNA accessibility to repair proteins. Monoubiquitinated at Lys-92 of histone H4 (H4K91ub1) in response to DNA damage. The exact role of H4K91ub1 in DNA damage response is still unclear but it may function as a licensing signal for additional histone H4 post-translational modifications such as H4 Lys-21 methylation (H4K20me).
Sumoylated, which is associated with transcriptional repression.

**细胞定位** Nucleus. Chromosome.

## 图片

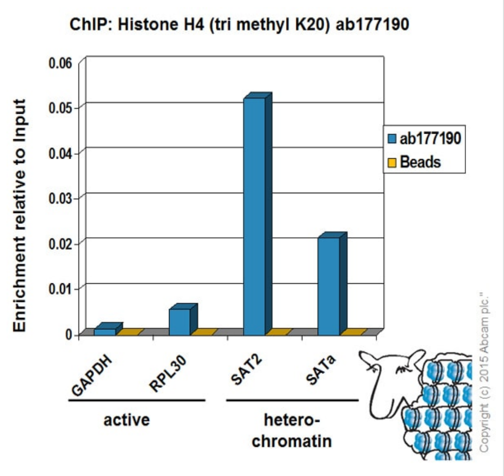


ChIP - Anti-Histone H4 (tri methyl K20) antibody [EPR17001(2)] - ChIP Grade (ab177190)

Chromatin was prepared from HeLa cells according to the Abcam X-ChIP protocol. Cells were fixed with formaldehyde for 10 minutes. The ChIP was performed with 25µg of chromatin, 2µg of ab177190 (blue), and 20µl of Protein A/G sepharose beads. No antibody was added to the beads control (yellow). The immunoprecipitated DNA was quantified by real time PCR (Taqman approach for active loci and Sybr green approach for heterochromatic loci). Primers and probes are located in the first kb of the transcribed region.

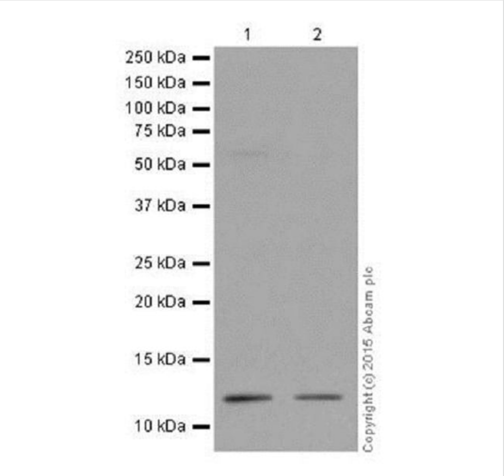


Western blot - Anti-Histone H4 (tri methyl K20) antibody [EPR17001(2)] - ChIP Grade (ab177190)

**All lanes :** Anti-Histone H4 (tri methyl K20) antibody [EPR17001(2)] - ChIP Grade (ab177190) at 1/1000 dilution

**Lane 1 :** HeLa (Human epithelial cells from cervix adenocarcinoma) cell lysate
**Lane 2 :** NIH 3T3 (Mouse embyro fibroblast cells) cell lysate

Lysates/proteins at 10 µg per lane.

**Secondary**
**All lanes :** Goat Anti-Rabbit IgG, (H+L), Peroxidase conjugated at 1/1000 dilution

**Predicted band size:** 11 kDa
**Observed band size:** 11 kDa

**Exposure time:** 1 minute

5% NFDM/TBST: Blocking and diluting buffer.

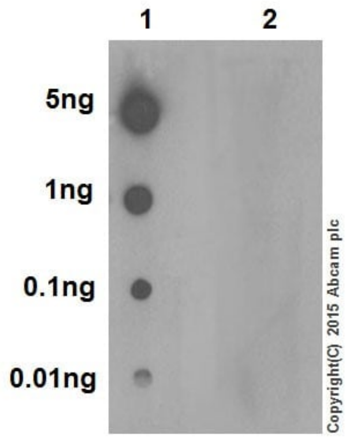


Dot Blot - Anti-Histone H4 (tri methyl K20) antibody [EPR17001(2)] - ChIP Grade (ab177190)

Dot blot analysis of Histone H4 (tri methyl K20) peptide(aa16-25) (Lane 1) and unmodified Histone H4 peptide (aa 16-25) (Lane 2) labeled using ab177190 at 1/1000 dilution, followed by Goat Anti-Rabbit IgG, (H+L), Peroxidase conjugated secondary antibody at 1/1000 dilution.

Blocking/Dilution buffer: 5% NFDM/TBST.

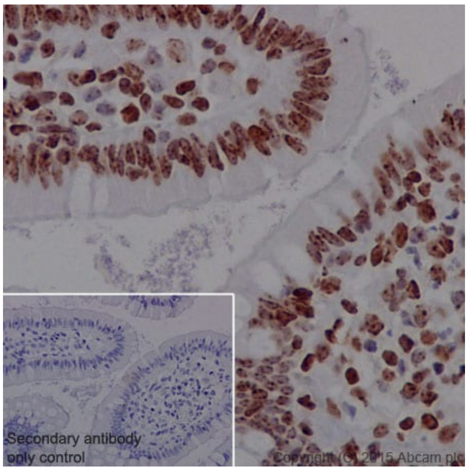


Immunohistochemistry (Formalin/PFA-fixed paraffin-embedded sections) - Anti-Histone H4 (tri methyl K20) antibody [EPR17001(2)] - ChIP Grade (ab177190)

Immunohistochemical analysis of paraffin-embedded Human colon tissue labeling Histone H4 (tri methyl K20) using ab177190 at 1/8000 dilution. A Goat Anti-Rabbit IgG H&L (HRP) (ab97051) was used as secondary at 1/500 dilution. Nucleus staining on Human colon was observed. Counterstained with Hematoxylin.
Negative control: Used PBS instead of primary antibody, secondary antibody is Goat Anti-Rabbit IgG H&L (HRP) (ab97051) at 1/500 dilution.

Perform heat mediated antigen retrieval with Tris/EDTA buffer pH 9.0 before commencing with IHC staining protocol.

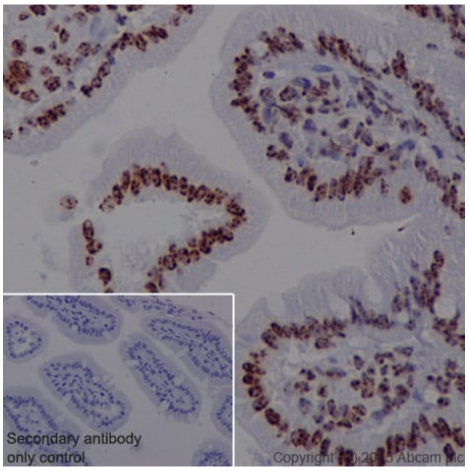


Immunohistochemistry (Formalin/PFA-fixed paraffin-embedded sections) - Anti-Histone H4 (tri methyl K20) antibody [EPR17001(2)] - ChIP Grade (ab177190)

Immunohistochemical analysis of paraffin-embedded mouse colon tissue labeling Histone H4 (tri methyl K20) using ab177190 at 1/8000 dilution. A Goat Anti-Rabbit IgG H&L (HRP) (ab97051) was used as secondary at 1/500 dilution. Nucleus staining on mouse colon was observed. Counterstained with Hematoxylin.
Negative control: Used PBS instead of primary antibody, secondary antibody is Goat Anti-Rabbit IgG H&L (HRP) (ab97051) at 1/500 dilution.

Perform heat mediated antigen retrieval with Tris/EDTA buffer pH 9.0 before commencing with IHC staining protocol.

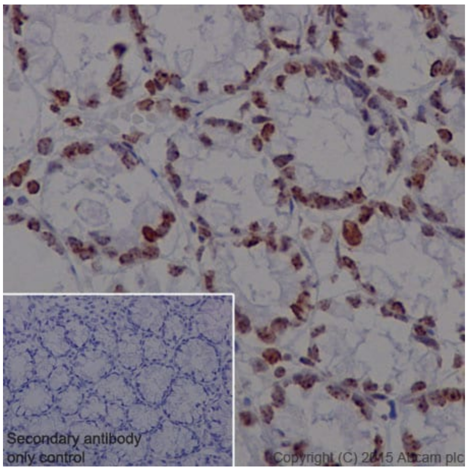


Immunohistochemistry (Formalin/PFA-fixed paraffin-embedded sections) - Anti-Histone H4 (tri methyl K20) antibody [EPR17001(2)] - ChIP Grade (ab177190)

Immunohistochemical analysis of paraffin-embedded rat colon tissue labeling Histone H4 (tri methyl K20) using ab177190 at 1/8000 dilution. A Goat Anti-Rabbit IgG H&L (HRP) (ab97051) was used as secondary at 1/500 dilution. Nucleus staining on rat colon was observed. Counterstained with Hematoxylin.
Negative control: Used PBS instead of primary antibody, secondary antibody is Goat Anti-Rabbit IgG H&L (HRP) (ab97051) at 1/500 dilution.

Perform heat mediated antigen retrieval with Tris/EDTA buffer pH 9.0 before commencing with IHC staining protocol.

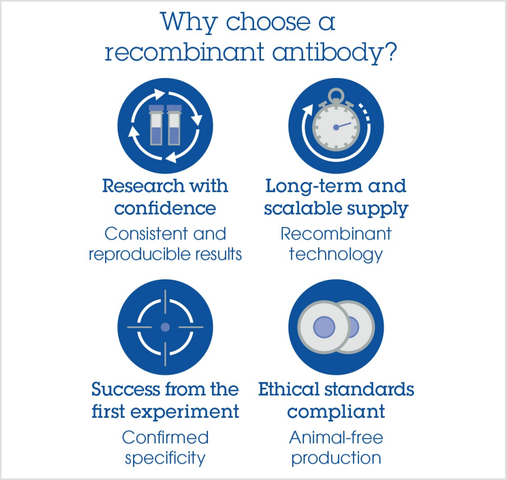


Anti-Histone H4 (tri methyl K20) antibody [EPR17001(2)] - ChIP Grade (ab177190)

**Please note:** All products are "FOR RESEARCH USE ONLY. NOT FOR USE IN DIAGNOSTIC PROCEDURES"

## Our Abpromise to you: Quality guaranteed and expert technical support

- Replacement or refund for products not performing as stated on the datasheet
- Valid for 12 months from date of delivery
- Response to your inquiry within 24 hours

- We provide support in Chinese, English, French, German, Japanese and Spanish
- Extensive multi-media technical resources to help you
- We investigate all quality concerns to ensure our products perform to the highest standards

If the product does not perform as described on this datasheet, we will offer a refund or replacement. For full details of the Abpromise, please visit https://www.abcam.cn/abpromise or contact our technical team.

## Terms and conditions

- Guarantee only valid for products bought direct from Abcam or one of our authorized distributors

abcam

## Product datasheet

# Anti-FOXM1 antibody ab180710

★★★★★ 1 Abreviews 22 References 1 图像

### 概述

| | |
|---|---|
| **产品名称** | Anti-FOXM1抗体 |
| **描述** | 兔多克隆抗体to FOXM1 |
| **宿主** | Rabbit |
| **经测试应用** | **适用于:** WB |
| **种属反应性** | **与反应:** Mouse, Rat, Human |
| **免疫原** | Recombinant fragment corresponding to Human FOXM1 aa 1-350 (N terminal).<br>Database link: Q08050 |
| **阳性对照** | WB: SW620, SKOV3, A-549, A-431 and HeLa cell lysates; Mouse heart, spleen and thymus tissue lysates; Rat thymus tissue lysate. |
| **常规说明** | The Life Science industry has been in the grips of a reproducibility crisis for a number of years. Abcam is leading the way in addressing this with our range of recombinant monoclonal antibodies and knockout edited cell lines for gold-standard validation. Please check that this product meets your needs before purchasing.<br>If you have any questions, special requirements or concerns, please send us an inquiry and/or contact our Support team ahead of purchase. Recommended alternatives for this product can be found below, along with publications, customer reviews and Q&As |

### 性能

| | |
|---|---|
| **形式** | Liquid |
| **存放说明** | Shipped at 4°C. Store at +4°C short term (1-2 weeks). Upon delivery aliquot. Store at -20°C long term. Avoid freeze / thaw cycle. |
| **存储溶液** | pH: 7.30<br>Preservative: 0.02% Sodium azide<br>Constituents: 50% Glycerol, 49% PBS |
| **纯度** | Immunogen affinity purified |
| **克隆** | 多克隆 |
| **同种型** | IgG |

### 应用

**The Abpromise guarantee** Abpromise™承诺保证使用ab180710于以下的经测试应用

“应用说明”部分 下显示的仅为推荐的起始稀释度；实际最佳的稀释度/浓度应由使用者检定。

| 应用 | Ab评论 | 说明 |
| --- | --- | --- |
| WB | ★★★★★ (1) | 1/500 - 1/2000. Predicted molecular weight: 84 kDa. |

## 靶标

**功能** Transcriptional activatory factor. May play a role in the control of cell proliferation.

**组织特异性** Expressed in thymus, testis, small intestine, colon followed by ovary. Appears to be expressed only in adult organs containing proliferating/cycling cells or in response to growth factors. Also expressed in epithelial cell lines derived from tumors. Not expressed in resting cells. Isoform 2 is highly expressed in testis.

**序列相似性** Contains 1 fork-head DNA-binding domain.

**发展阶段** Embryonic expression pattern: liver, lung, intestine, kidney, urinary tract; adult expression pattern: intestine, colon, testis and thymus.

**结构域** Within the protein there is a domain which acts as a transcriptional activator. Insertion of a splicing sequence within it inactivates this transcriptional activity, as it is the case for isoform 4.

**翻译后修饰** Phosphorylated in M (mitotic) phase.

**细胞定位** Nucleus.

## 图片

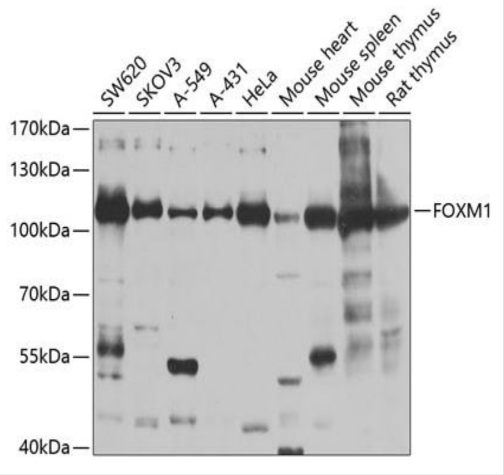


Western blot - Anti-FOXM1 antibody (ab180710)

**All lanes :** Anti-FOXM1 antibody (ab180710) at 1/1000 dilution

**Lane 1 :** SW620 cell lysate
**Lane 2 :** SKOV3 cell lysate
**Lane 3 :** A-549 cell lysate
**Lane 4 :** A-431 cell lysate
**Lane 5 :** HeLa cell lysate
**Lane 6 :** Mouse heart tissue lysate
**Lane 7 :** Mouse spleen tissue lysate
**Lane 8 :** Mouse thymus tissue lysate
**Lane 9 :** Rat thymus tissue lysate

Lysates/proteins at 25 µg per lane.

**Secondary**
**All lanes :** HRP Goat Anti-Rabbit IgG (H+L)

Developed using the ECL technique.

**Predicted band size:** 84 kDa

**Exposure time:** 90 seconds

**Please note:** All products are "FOR RESEARCH USE ONLY. NOT FOR USE IN DIAGNOSTIC PROCEDURES"

## Our Abpromise to you: Quality guaranteed and expert technical support

- Replacement or refund for products not performing as stated on the datasheet
- Valid for 12 months from date of delivery
- Response to your inquiry within 24 hours

- We provide support in Chinese, English, French, German, Japanese and Spanish
- Extensive multi-media technical resources to help you
- We investigate all quality concerns to ensure our products perform to the highest standards

If the product does not perform as described on this datasheet, we will offer a refund or replacement. For full details of the Abpromise, please visit https://www.abcam.cn/abpromise or contact our technical team.

## Terms and conditions

- Guarantee only valid for products bought direct from Abcam or one of our authorized distributors

abcam

# Product datasheet

# Anti-Cdc20 antibody ab183479

7 References 3 图像

## 概述

| | |
|---|---|
| **产品名称** | Anti-Cdc20抗体 |
| **描述** | 兔多克隆抗体to Cdc20 |
| **宿主** | Rabbit |
| **经测试应用** | **适用于:** WB, IP, ICC/IF |
| **种属反应性** | **与反应:** Mouse, Rat, Human |
| **免疫原** | Recombinant fragment corresponding to Human Cdc20 aa 1-300.<br>Database link: Q12834<br>Run BLAST with Run BLAST with |
| **阳性对照** | NIH 3T3 and INS1 cell lysates; U2OS cells transfected with Cdc20. |
| **常规说明** | The Life Science industry has been in the grips of a reproducibility crisis for a number of years. Abcam is leading the way in addressing this with our range of recombinant monoclonal antibodies and knockout edited cell lines for gold-standard validation. Please check that this product meets your needs before purchasing.<br>If you have any questions, special requirements or concerns, please send us an inquiry and/or contact our Support team ahead of purchase. Recommended alternatives for this product can be found below, along with publications, customer reviews and Q&As |

## 性能

| | |
|---|---|
| **形式** | Liquid |
| **存放说明** | Shipped at 4°C. Store at +4°C short term (1-2 weeks). Upon delivery aliquot. Store at -20°C long term. Avoid freeze / thaw cycle. |
| **存储溶液** | Preservative: 0.05% Sodium azide |
| **纯度** | Whole antiserum |
| **克隆** | 多克隆 |
| **同种型** | IgG |

## 应用

**The Abpromise guarantee** Abpromise™承诺保证使用ab183479于以下的经测试应用

“应用说明”部分 下显示的仅为推荐的起始稀释度；实际最佳的稀释度/浓度应由使用者检定。

| 应用 | Ab评论 | 说明 |
|---|---|---|
| WB | | 1/2000. Predicted molecular weight: 55 kDa. |
| IP | | Use at an assay dependent concentration. |
| ICC/IF | | 1/1000. |

## 靶标

**功能** Required for full ubiquitin ligase activity of the anaphase promoting complex/cyclosome (APC/C) and may confer substrate specificity upon the complex. Is regulated by MAD2L1. In metaphase the MAD2L1-CDC20-APC/C ternary complex is inactive and in anaphase the CDC20-APC/C binary complex is active in degrading substrates.

**通路** Protein modification; protein ubiquitination.

**序列相似性** Belongs to the WD repeat CDC20/Fizzy family.
Contains 7 WD repeats.

**发展阶段** Synthesis is initiated at G1/S, protein level peaks in M phase and protein is abruptly degraded at M/G1 transition.

**翻译后修饰** Phosphorylated during mitosis, probably by maturation promoting factor (MPF). Phosphorylated by BUB1 at Ser-41; Ser-72; Ser-92; Ser-153; Thr-157 and Ser-161.
Ubiquitinated and degraded by the proteasome during spindle assembly checkpoint.
Deubiquitinated by USP44, leading to stabilize the MAD2L1-CDC20-APC/C ternary complex, thereby preventing premature activation of the APC/C.

## 图片

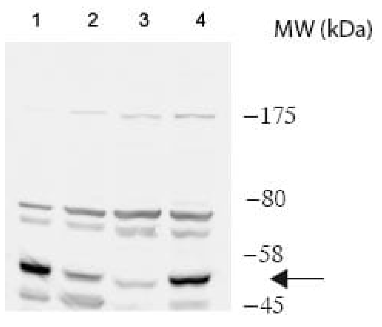


Western blot - Anti-Cdc20 antibody (ab183479)

**All lanes :** Anti-Cdc20 antibody (ab183479) at 1/2000 dilution

**Lane 1 :** U2OS cells transfected with control si RNA
**Lane 2 :** U2OS cells transfected with Cdc20 si RNA
**Lane 3 :** Rat INS1 cell lysate
**Lane 4 :** Mouse NIH 3T3 cell lysate

Lysates/proteins at 40 µg per lane.

**Predicted band size:** 55 kDa

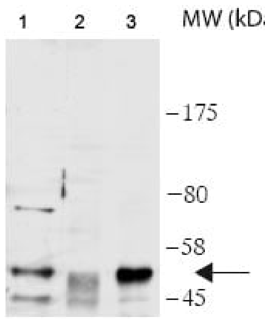


Immunoprecipitation - Anti-Cdc20 antibody (ab183479)

Western blot analysis of Immunoprecipitation using U2OS cells

Lane 1: Input.

Lane 2: control serum.

Lane 3: ab183479.

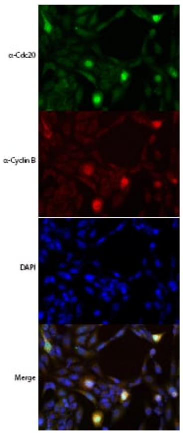


Immunocytochemistry/ Immunofluorescence - Anti-Cdc20 antibody (ab183479)

Immunofluorescent analysis of U2OS cells showing labeling of Cdc20 with ab183479 at 1/1000 dilution. Anti-Cyclin B labeling is seen in red and DAPI counterstain in blue. Lower image is merged.

**Please note:** All products are "FOR RESEARCH USE ONLY. NOT FOR USE IN DIAGNOSTIC PROCEDURES"

## Our Abpromise to you: Quality guaranteed and expert technical support

- Replacement or refund for products not performing as stated on the datasheet
- Valid for 12 months from date of delivery
- Response to your inquiry within 24 hours

- We provide support in Chinese, English, French, German, Japanese and Spanish
- Extensive multi-media technical resources to help you
- We investigate all quality concerns to ensure our products perform to the highest standards

If the product does not perform as described on this datasheet, we will offer a refund or replacement. For full details of the Abpromise, please visit https://www.abcam.cn/abpromise or contact our technical team.

## Terms and conditions

- Guarantee only valid for products bought direct from Abcam or one of our authorized distributors

9F, No. 108, Jhouzih St.,Taipei, Taiwan
Tel: + 886-2-8751-1888 Fax: + 886-2-6602-1218
E-mail: sales@abnova.com

# Datasheet

## MYST3 polyclonal antibody

**Catalog Number:** PAB8745

**Regulatory Status:** For research use only (RUO)

**Product Description:** Rabbit polyclonal antibody raised against synthetic peptide of MYST3.

**Immunogen:** A synthetic peptide corresponding to C-terminus of human MYST3.

**Host:** Rabbit

**Theoretical MW (kDa):** 220

**Reactivity:** Human,Mouse,Rat

**Applications:** WB-Ce
(See our web site product page for detailed applications information)

**Protocols:** See our web site at http://www.abnova.com/support/protocols.asp or product page for detailed protocols

**Specificity:** This antibody recognizes 220 KDa human HAT-3. A 50 KDa unknown band can also be detected.

**Form:** Liquid

**Recommend Usage:** Western Blot (2-6 ug/mL)
The optimal working dilution should be determined by the end user.

**Storage Buffer:** In PBS (30% glycerol, 0.5% BSA, 0.01% thimerosal)

**Storage Instruction:** Store at -20°C. For long term storage store at -80°C.
Aliquot to avoid repeated freezing and thawing.

**Entrez GeneID:** 7994

**Gene Symbol:** MYST3

**Gene Alias:** KAT6A, MGC167033, MOZ, RUNXBP2, ZNF220
