# Supplementary material for: Inhibition of DYRK1A, via histone modification, promotes cardiomyocyte cell cycle activation and cardiac repair after myocardial infarction
Source: eBioMedicine. 2022 Jul 8;82:104139. doi: 10.1016/j.ebiom.2022.104139 (PMC9278077; doi:10.1016/j.ebiom.2022.104139)
Supplement: Supplementary file 6 [file mmc6.pdf]

CELLAPY

首页 细胞治疗 心肌药筛 赛贝产品 赛贝服务 客户支持

赛贝产品

干细胞培养

PSCeasy®II人多潜能干细胞培养基

PSCeasy®人多潜能干细胞消化液

PSCeasy® 人多潜能干细胞复苏培养基

PSCeasy® 人多潜能干细胞铺底工作液

MSCeasy人间充质干细胞培养基

PGM1人多潜能干细胞培养基

细胞产品

人诱导多能干细胞/hiPSC

体细胞分离培养试剂

UrinEasy®尿液细胞分离试剂盒

UrinEasy®尿液细胞扩增试剂盒

体细胞重编程试剂

ReproEasy®人体细胞重编程培养基

Reproeasy hiPSC重编程试剂盒

心肌细胞培养试剂

CardioEasy®人心肌细胞

CardioEasy®人心肌细胞消化液

CardioEasy®人心肌细胞分化试剂盒

CardioEasy®人心肌细胞纯化培养基

CardioEasy®人心肌细胞维持培养基

基因修饰工具

AAVS1-Easy基因修饰试剂盒

支原体检测试剂

MycoEasy支原体检测试剂盒

NeuroEasy人神经干细胞

NeuroEasy人神经元分化试剂盒

NeuroEasy人神经干细胞（贴壁）培养试剂盒

NeuroEasy人神经干细胞诱导培养试剂盒

其他产品

细胞沉淀

细胞因子

CardioEasy®人心肌细胞

CardioEasy®人心肌细胞是一种即用型的高纯度心肌产品。该产品采用赛贝生物（Cellapy®）的心肌定向分化和纯化方法产生。同时，包括hiPSC在内的所有细胞培养分化过程采用化学成分确定的培养试剂。

CardioEasy® 人心肌细胞主要由具备自主电生理活动的心室肌样细胞组成，同时也包含少量心房肌和窦房结样细胞。这些细胞表达常规的心肌特异性基因，包括多种收缩蛋白和离子通道，同时具备经典的心肌细胞电生理活性，能够对电生理和生物化学刺激做出心肌细胞的常规反应。以电生理和钙转导信号记录为例，赛贝心肌细胞可以作为医疗科研，新药筛选，毒理学测定和其他生理学研究的理想工具。

产品介绍 | 细胞功能鉴定 | 疑难解答 | 心肌视频

货号： CA2201106

规格： 1×106

储存条件：

活细胞储存于25℃ ~ 37℃，收到后请立即放入CO2培养箱。 冻存细胞暂存于-80℃冰箱，2周有效期；液氮储存，
期。

产品简介：
CardioEasy®人心肌细胞产品是一种即用型的高纯度心肌产品。该产品采用赛贝生物（Cellapy®）的高效心肌定
方法产生。同时，包括人多潜能干细胞（hESC/hiPSC）在内的所有细胞培养分化过程采用化学成分确定的培养试剂。
人心肌细胞主要由具备自主电生理活动的心室肌样细胞组成，同时也包含少量心房肌和窦房结样细胞。这些细胞表达常
性基因，包括多种收缩蛋白和离子通道。同时，这些细胞具备经典的心肌细胞电生理活性，能够对电生理和生物化学刺
胞的常规反应。以电生理和钙转导信号记录为例，这些细胞可以作为新药筛选，毒理学测定和其他生理学研究的理想工

产品内容：

组份代码	名称	规格	数量
CA2201106	CardioEasy®人心肌细胞-药筛级	1×106	1支

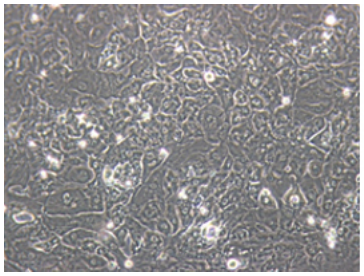

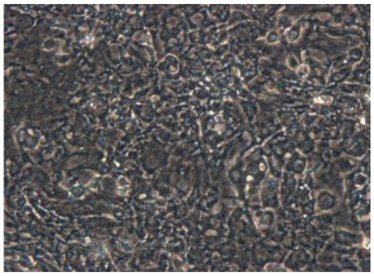

CardioEasy®心肌细胞

CardioEasy®人心肌细胞优势

**人源细胞**	人心肌细胞与常用实验动物的心肌细胞相比，存在较大生理学差异。例如，人心脏生理条件下每分钟搏动

	500-700次，大鼠为300-400次。赛贝人源心肌细胞从hiPS诱导分化而成，具备动物细胞无法比
**纯度高**	细胞纯度可达90%以上，可由搏动和表达GFP的细胞比例证实。
**均一性好**	可以提供大量组成稳定的心肌细胞群体，产品经过严格的质检，保证后续实验良好的重复
**完全的心肌生理学活性**	具备大多数正常人类心脏中心肌细胞的标准电生理学和生物化学特性，可以受到电兴奋刺激而产生搏动，不<br>以传导电兴奋信号。可以用于多种生物化学和电生理学检测。
**培养简易**	可以冻存细胞的形式运输和保存，并提供配套的接种培养基和维持培养基。仅需常规细胞培养设施B
**稳定性强可长期培养**	可以被长期体外培养。在理想条件下，可维持存活达到3个月以上，并且保持节律性的搏动，是长期药物毒<br>的理想模型工具。

电话：010—69737398 业务咨询邮箱：info@cellapybio.com 技术支持邮箱：support@cellapybio.com 地址:北京市昌平区生命科学
版权所有：北京赛贝生物技术有限公司 京ICP备13027174号 技术支持： 云梦网络

阿里云 aliyun.com 本网站由阿里云提供云计算及安全服务

赛贝产品

干细胞培养

PSCeasy®II人多潜能干细胞培养基

PSCeasy®人多潜能干细胞消化液

PSCeasy® 人多潜能干细胞复苏培养基

PSCeasy® 人多潜能干细胞铺底工作液

MSCeasy人间充质干细胞培养基

PGM1人多潜能干细胞培养基

细胞产品

人诱导多能干细胞/hiPSC

体细胞分离培养试剂

UrinEasy®尿液细胞分离试剂盒

UrinEasy®尿液细胞扩增试剂盒

体细胞重编程试剂

ReproEasy®人体细胞重编程培养基

Reproeasy hiPSC重编程试剂盒

心肌细胞培养试剂

CardioEasy®人心肌细胞

CardioEasy®人心肌细胞消化液

CardioEasy®人心肌细胞分化试剂盒

CardioEasy®人心肌细胞纯化培养基

CardioEasy®人心肌细胞维持培养基

基因修饰工具

AAVS1-Easy基因修饰试剂盒

支原体检测试剂

MycoEasy支原体检测试剂盒

NeuroEasy人神经干细胞

NeuroEasy人神经元分化试剂盒

NeuroEasy人神经干细胞（贴壁）培养试剂盒

NeuroEasy人神经干细胞诱导培养试剂盒

其他产品

细胞沉淀

细胞因子

CardioEasy®人心肌细胞

CardioEasy®人心肌细胞是一种即用型的高纯度心肌产品。该产品采用赛贝生物（Cellapy®）的心肌定向分化和纯化方法产生。同时，包括hiPSC在内的所有细胞培养分化过程采用化学成分确定的培养试剂。

CardioEasy® 人心肌细胞主要由具备自主电生理活动的心室肌样细胞组成，同时也包含少量心房肌和窦房结样细胞。这些细胞表达常规的心肌特异性基因，包括多种收缩蛋白和离子通道，同时具备经典的心肌细胞电生理活性，能够对电生理和生物化学刺激做出心肌细胞的常规反应。以电生理和钙转导信号记录为例，赛贝心肌细胞可以作为医疗科研，新药筛选，毒理学测定和其他生理学研究的理想工具。

产品介绍	细胞功能鉴定	疑难解答	心肌视频

CardioEasy®人心肌细胞胞特异标记物荧光染色图

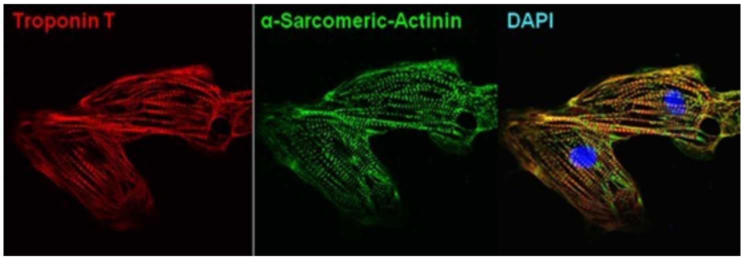

CardioEasy®人心肌细胞表达主要的心肌特异性收缩蛋白Cardiac Troponin T和α-Sarcomeric-Actinin，并且形成非

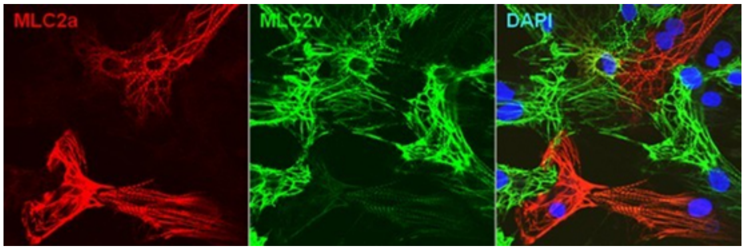

CardioEasy®人心肌细胞主要由心室肌样细胞组成，表达心室特异性轻链肌球蛋白MLC2v，少量其他心肌细胞表达心
MLC2a。

RT-PCR 检测图

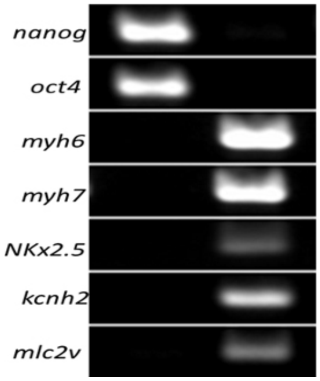

CardioEasy®人心肌细胞纯度鉴定图

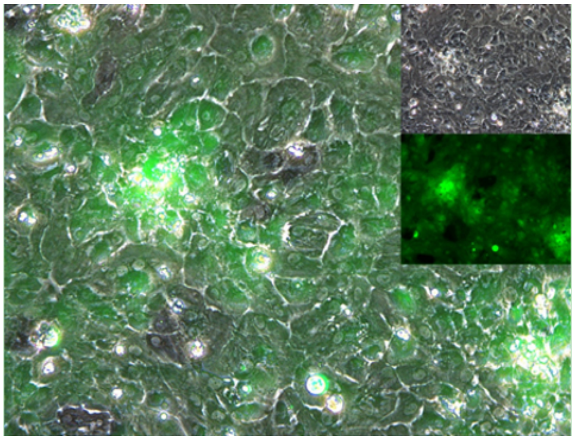

CardioEasy®人心肌细胞NKX2.5启动子调控下特异性的表达绿色荧光蛋白（GFP）

CardioEasy®人心肌细胞电生理检测（RTCA）

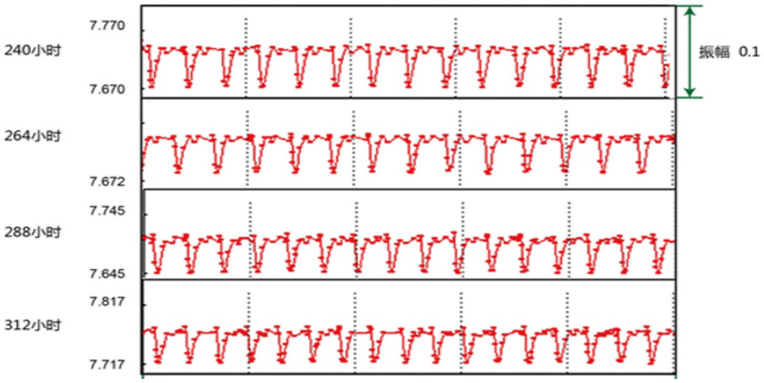

CardioEasy®人心肌细胞转染效率测试

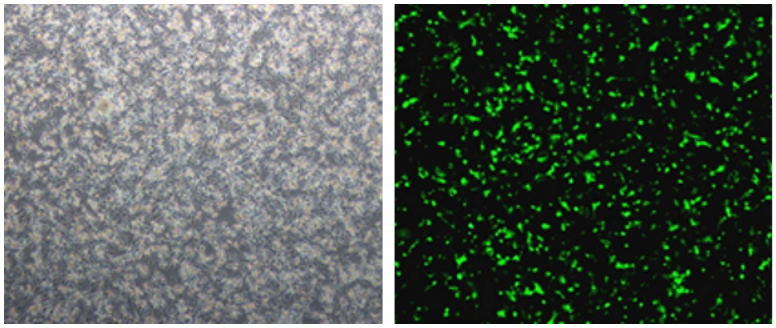

CardioEasy®人心肌细胞转染GFP质粒（Lipofectamine LTX and PLUS Reagents），48小时后观察，GFP荧光[illegible]

CardioEasy®人心肌细胞膜片钳检测

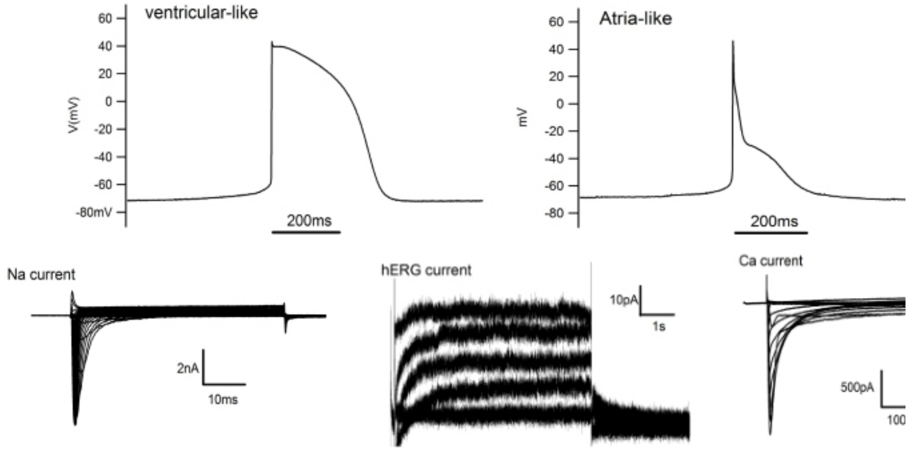

图注： CardioEasy®人心肌细胞自发Na，hERG，Ca离子通道

电话：010—69737398 业务咨询邮箱：info@cellapybio.com 技术支持邮箱：support@cellapybio.com 地址:北京市昌平区生命科学
版权所有：北京赛贝生物技术有限公司 京ICP备13027174号 技术支持： 云梦网络

本网站由阿里云提供云计算及安全服务
